# One-pot divergent synthesis of 5-arylmethyl oxazoles and polyacenes from propargylic amides and arynes

**DOI:** 10.1039/d6qo01223j

**Published:** 2026-09-14

**Authors:** A. Yannic R. Werling, Yun Yang, Christopher R. Jones

**Affiliations:** a Department of Chemistry, Queen Mary University of London Mile End Road London E1 4NS UK c.jones@qmul.ac.uk

## Abstract

We report a sequential one-pot gold-catalyzed cyclization/aryne cycloaddition strategy that affords structurally diverse 5-arylmethyl oxazoles and substituted polyacenes from simple propargylic amides. Arynes are shown to engage the oxazoline products from gold catalysis *via* an ene reaction, with the resulting oxazoles trapping excess aryne in a cascade cycloaddition process to furnish epoxyacenes. Telescoping a subsequent deoxygenation reaction provides direct access to a range of polyaromatic hydrocarbon frameworks *via* a one-pot, three-stage transformation. This protocol is simple to perform, operates under mild reaction conditions and affords high levels of product selectivity through straightforward control of aryne stoichiometry.

## Abstract 2

A tandem Au(i) cyclization/aryne cycloaddition process enables selective access to 5-arylmethyl oxazoles or substituted polyacene derivatives directly from simple propargylic amides.

## Graphical abstract

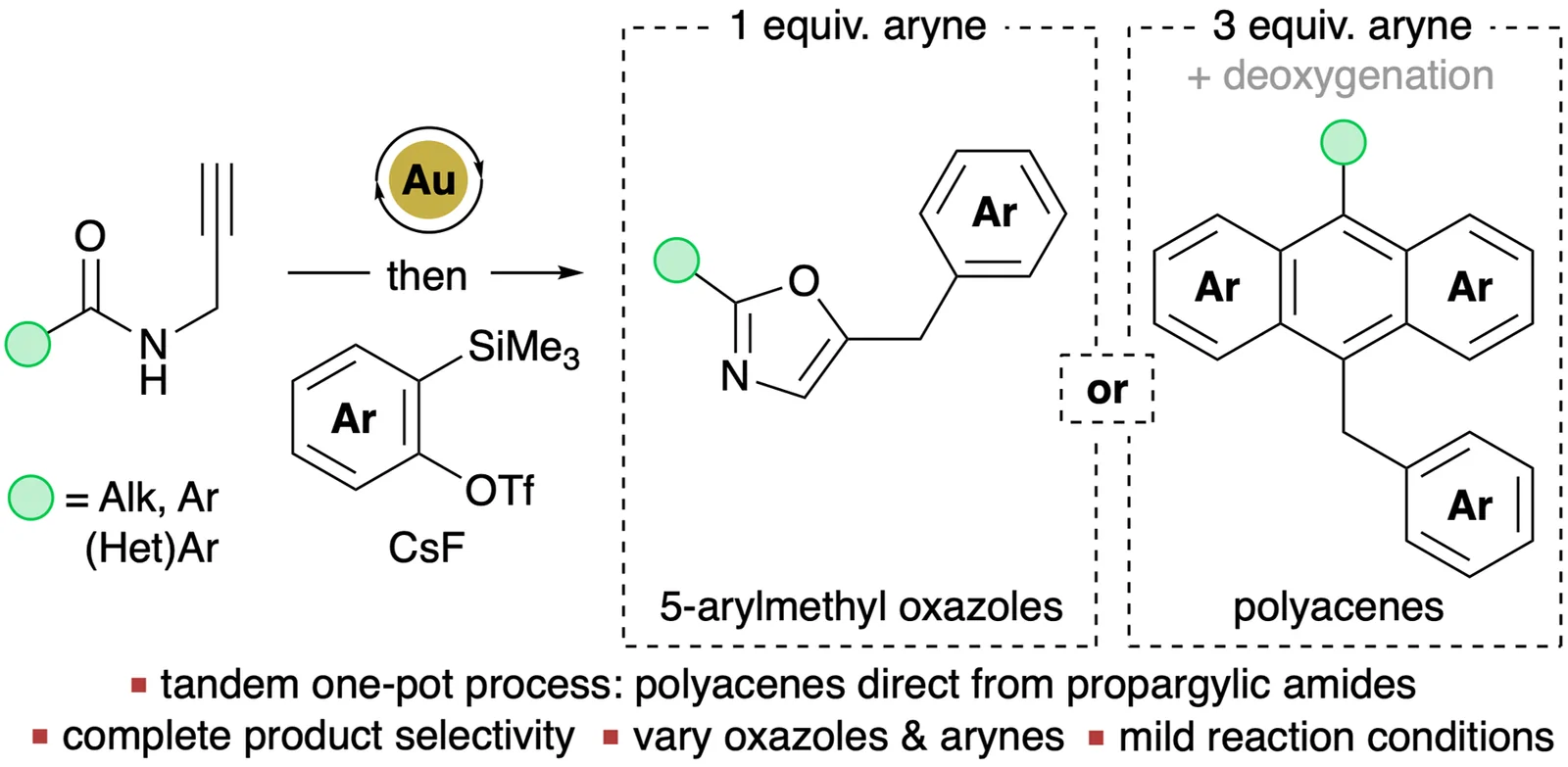

## Introduction

Arynes are versatile reactive intermediates that rapidly afford valuable benzenoid and heterocyclic frameworks through the formation of multiple C–C or C–X bonds in a single operation.^1^ Their utility extends from complex natural product synthesis^1*b*,*k*^ to the preparation of polycyclic aromatic hydrocarbons (PAHs)^2^ for application in organic semiconducting devices.^3^ Renewed interest in aryne chemistry has been driven by the development of precursors that act under mild conditions, such as *o*-trimethylsilylaryl triflates (*o*SATs),^4^ the hexadehydro-Diels–Alder reaction of polyalkynes^5^ and aryl onium salts,^6^ which continue to enable the discovery of new modes of reactivity.^1*j*,*l*^

**Scheme 1:**
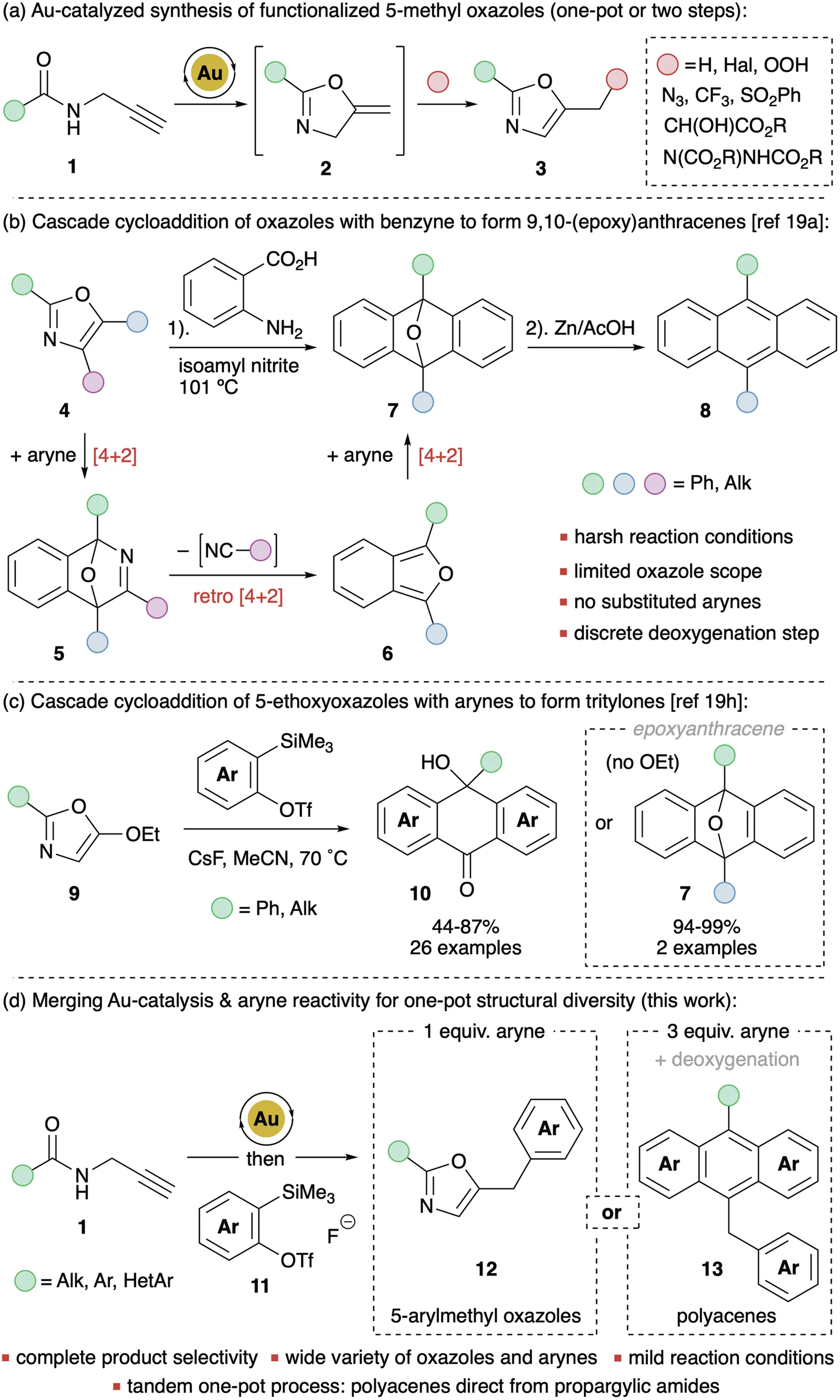
Oxazole synthesis *via* gold-catalyzed cyclizations of *N*-propargylamides and cascade cycloadditions of arynes with oxazoles.

Oxazoles are privileged scaffolds in medicinal chemistry that demonstrate high-affinity binding across diverse biological targets.^7^ They feature in several FDA-approved drugs (*e.g.* oxacilin and oxaprozin)^8^ and many natural products (*e.g.* telomestatin and bengazole A),^9^ as well as in polymers for photophysical applications.^10^ One widely studied method for the *de novo* preparation of diverse oxazole derivatives (3) is the intramolecular cyclization of propargyl amides 1, pioneered by Hashmi and co-workers, which proceeds *via* oxazoline intermediate 2 (Scheme 1a).^11^ Whilst the cycloisomerization of 1 can also be achieved using alternative activation modes,^12^ the mild Au-mediated conditions render this an attractive option.

Considering potential oxazole structural diversification, gold-catalyzed cyclization of non-terminal alkynes would introduce C5-functionality beyond the methyl group; however, many limitations concerning substrate sterics and electronics are encountered. This is due to hindered cyclization – typically necessitating alkynes bearing electron-withdrawing substituents – and the increasingly competitive 6-*endo-dig* cyclization that leads to poorer regioselectivity.^11*d*,13^ Alternatively, the intermediate oxazoline 2 offers opportunities for functionalization after cyclization. Hashmi and co-workers observed that Au(i) catalysts preferentially form the oxazoline species,^11*c*^ which has been functionalized *via* ene or radical addition reactions to furnish a range of different C-5 substituted oxazoles (3) (see Scheme 1a);^14^ however, no analogous method exists for aryl incorporation. Given the prevalence of the oxazole scaffold in pharmaceuticals, natural products and functional materials,^7–10^ methods enabling access to new 5-arylmethyl derivatives remain highly desirable.

Arynes are well established as good enophiles, although the majority of examples feature hydrocarbon-based ene donors with limited functionality.^15^ However, encouraged by our previous reports of arynes engaging ene donors bearing multiple heteratoms,^16^ we envisaged an opportunity for oxazoline 2 to participate in an aryne ene reaction that would afford 5-arylmethyl oxazoles 12 (Scheme 1d). Importantly, several studies have demonstrated successful incorporation of gold-catalysis within aryne reactions,^17^ suggesting that oxazoline 2 might similarly engage in aryne trapping and avoid potentially deleterious Au-mediated cyclotrimerization.^18^ Compared to the cyclization of internal alkynes, the proposed post-cyclization functionalization strategy should enable much broader 5-arylmethyl oxazole scope (electron-rich, electron-poor and bulky aryl groups) and obviate the regioselectivity issues associated with competing 6-ring formation.

Interestingly, arynes have been reported to engage oxazoles in Diels–Alder cycloadditions (Scheme 1b).^19^ Early studies using harsh benzyne-forming conditions with a small number of oxazoles established the formation of cycloadducts (5) that, upon nitrile extrusion, generate isobenzofurans (6) capable of further aryne trapping to furnish epoxyacenes 7.^19*a*–*d*^ More recently, the mild conditions enabled by *o*SAT precursors have expanded the oxazole–benzyne cycloaddition to afford diverse isoquinoline derivatives, accessed *via* cycloadduct 5,^19*e*–*g*^ as well as tritylones (10) produced by ring-opening of the corresponding epoxyanthracene intermediates (Scheme 1c).^19*h*^ Raminelli and co-workers also reported three examples of epoxyacene products; however, only benzyne was examined.^19*e*^

Given our interest in harnessing the reactivity of arynes,^20^ we reasoned that the proposed 5-arylmethyl oxazole products (12) might themselves participate in aryne cycloaddition cascades, providing rapid access to new 9,10-epoxyacene and polyacene architectures, including pentacene, relevant to organic materials chemistry (Scheme 1d).^3^ As such, herein we report a divergent strategy that exploits sequential aryne trapping of oxazolines 2, oxazoles 12 and transient intermediate isobenzofurans 6. Control of aryne stoichiometry enables selective access to diverse and valuable (hetero)aromatic compounds: either 5-arylmethyl oxazoles 12 or polyacene derivatives 13. Furthermore, telescoping the sequence with a final deoxygenation step enables efficient access to polyacenes in high overall yields directly from simple propargyl amides.

## Results and discussion

Initial efforts focused on testing the feasibility of the proposed aryne ene reaction of methylene oxazoline 2 for the formation of 5-arylmethyl oxazoles 12. To this end, *N*-(prop-2-ynyl)pivalamide 14 could be fully converted into the intermediate oxazoline using 1.0 mol% of PPh_3_AuOTf in a range of different solvents (dichloromethane, chloroform, THF, acetonitrile, toluene). To probe the aryne ene reaction in isolation, the Au(i) catalyst was removed by filtration prior to addition of 2-trimethylsilylphenyl triflate 11a and KF/18-crown-6 (Table 1). After 24 hours at room temperature, we were pleased to observe the formation of the desired 2-*tert*-butyl-5-benzyl oxazole 15a in 69% isolated yield (entry 1). With a view to developing a one-pot procedure for the preparation of 5-arylmethyl oxazoles (12) from propargyl amide 1, attention was then turned to finding conditions that were suitable for both the cyclization and ene reaction. Further preliminary studies revealed that the Au(i)-cyclization was tolerant of precursor 11a and that the gold catalyst was compatible with the aryne ene reaction. However, fluoride was found to inhibit the catalysis and would therefore have to be added to the vessel once cyclization was complete. For simplicity, it was decided that the precursor would also be introduced at this stage. With these observations in mind, we embarked on developing a one-pot, two-stage tandem cyclization/aryne ene process; selected optimization studies are presented in Table 1.

**Table 1:** Selected optimization studies for the tandem gold-catalyzed cyclization/aryne ene reaction of propargylic amide 14^a^

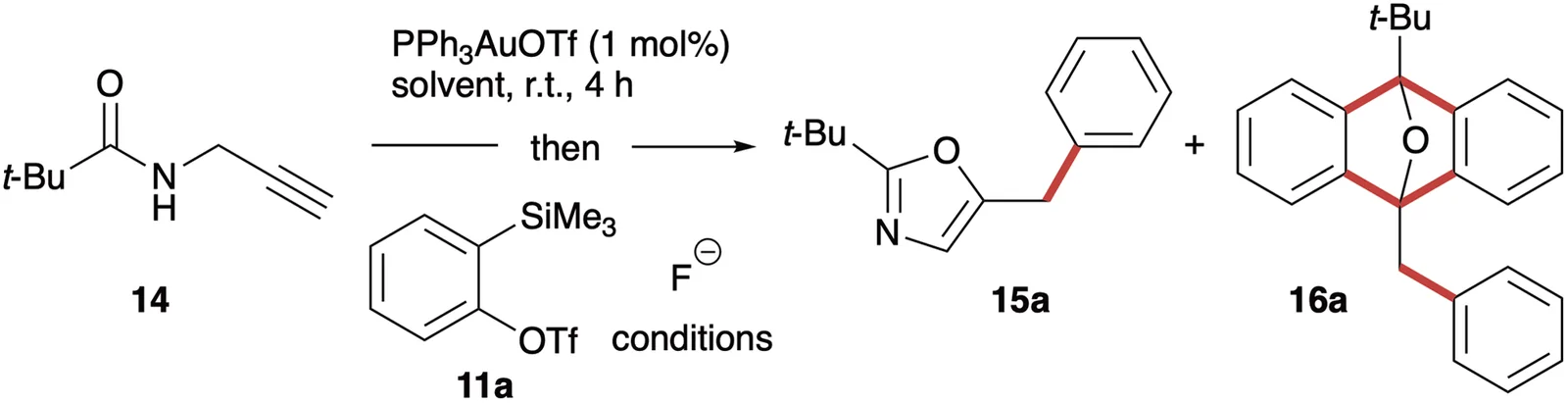								
Entry	Fluoride source	Additive^b^	Aryne (equiv.)	Solvent	Temp. (°C)	Time (h)	Yield^c^ (%)	
							15a	16a
1^d^	KF	18-Crown-6	1.1	THF	r.t.	24	69	Trace
2	KF	18-Crown-6	1.1	THF	r.t.	24	71	Trace
3	TBAF^e^	—	1.1	THF	r.t.	24	55	—
4	TBAT	—	1.1	THF	r.t.	24	66	—
5	CsF	—	1.1	MeCN	r.t.	24	67	13
6	CsF	18-Crown-6	1.1	MeCN	r.t.	24	66	13
7	CsF	—	1.1	CH_2_Cl_2_	r.t.	24	—	—
8	CsF	—	1.0	MeCN	r.t.	24	62	Trace
9	CsF	—	1.3	MeCN	r.t.	24	57	23
10	CsF	—	1.1	MeCN	50	16	68	12
11	CsF^f^	—	4.0	MeCN	50	16	—	85

a Reaction conditions: propargyl amide 14 (1.0 equiv.), PPh_3_AuOTf (1 mol%), solvent [0.01 M], r.t., 4 h, then aryne precursor 11a, fluoride source (3.0 equiv.), additive, temp. and time.

b 2.5 equiv. of additive relative to aryne.

c Isolated yields throughout.

d Reaction mixture passed through a silica plug prior to the 2^nd^ step.

e 1.0 M in THF.

f 10 equiv. of CsF.

Evaluation of some common conditions for aryne generation from *o*SATs revealed that the overall transformation was tolerant of a range of additives and solvents (entries 2–6). Considering reagent economy and ease of purification, CsF in acetonitrile was identified as the most promising combination (entry 5). For example, cyclization of propargyl amide 14 using 1 mol% of PPh_3_AuOTf in acetonitrile was complete after 4 h at room temperature (r.t.), at which point 11a and CsF were added to the flask and left for a further 24 h to afford 5-benzyl oxazole 15a in 67% yield. However, attempts to employ dichloromethane, one of the most widely used solvents in Au(i)-catalyzed cyclizations of propargyl amides,^12^ proved incompatible with CsF-mediated aryne formation (entry 7), presumably due to poor fluoride solubility. Elsewhere, altering the aryne stoichiometry resulted in erosion of oxazole yield. For example, a reduction in *o*SAT loading (1.0 equivalent, entry 8) led to the recovery of trace amounts of oxazoline, whereas increasing to 1.3 equivalents afforded more significant amounts of 9-benzyl-10-(*tert*-butyl)-9,10-dihydro-9,10-epoxyanthracene 16a (entry 9), which results from oxazole 15a engaging excess benzyne in cascade [4 + 2] cycloadditions.^19^ Increasing the temperature of the aryne ene reaction to 50 °C led to a faster process (16 h, entry 10), whilst maintaining the overall yield. This is particularly interesting as benzyne precursor 11a has been reported to undergo efficient cyclotrimerization under similar reaction conditions (PPh_3_AuCl or PPh_3_AuSbF_6_ in MeCN at 60 °C).^18^ In contrast, no triphenylene was observed during our studies, which provided good support for the compatibility of Au-catalysis with the desired aryne reactivity. Finally, considering our overall aim of achieving structural diversity through aryne stoichiometry (see Scheme 1d), it was gratifying that the use of 4.0 equivalents of benzyne precursor 11a selectively furnished epoxyanthracene 16a in 85% yield at 50 °C with no trace of oxazole 15a (entry 11). Notably, this combination of the Au-cyclization and cascade aryne cycloaddition would prove to be amenable to a wide range of propargylic amides and arynes (*vide infra*).

**Scheme 2:**
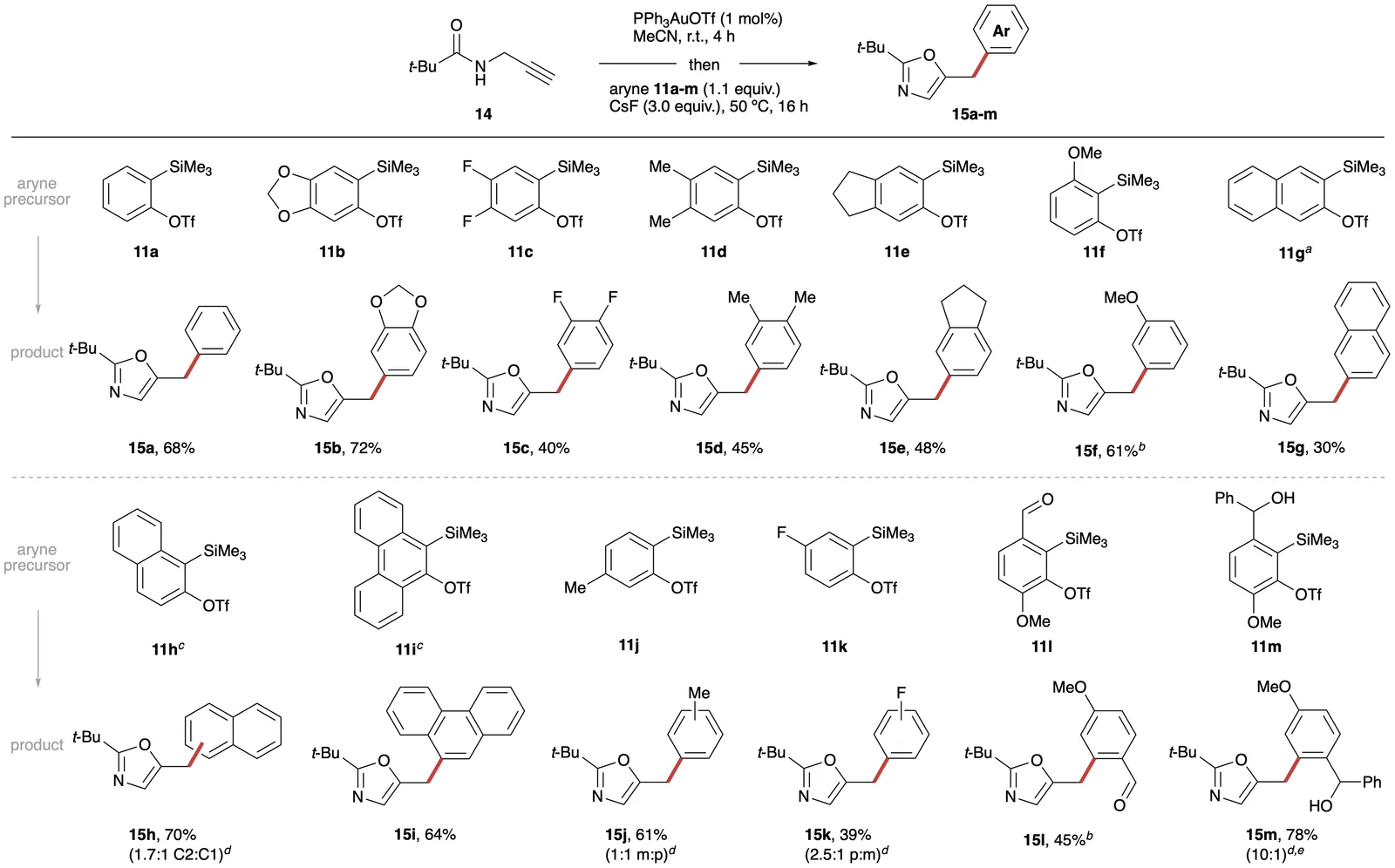
Evaluation of the aryne scope in the tandem Au(i) cyclization/aryne ene reaction of propargyl amide 14. Yields of isolated products throughout. ^*a*^ Reaction conducted at r.t. ^*b*^ Single regioisomer by ^1^H NMR spectroscopy. ^*c*^ 2.0 equivalents of aryne precursor and 5.0 equivalents of CsF added. ^*d*^ Ratio determined by ^1^H NMR spectroscopy. ^*e*^ Major regioisomer drawn.

With an initial focus on oxazole synthesis, the transformation was first shown to be reproducible on a larger scale; 2.0 g of pivalamide 14 produced 2-*tert*-butyl-5-benzyl oxazole 15a in 60% isolated yield under the optimized conditions (see Table 1, entry 10). Next, we began to explore the scope of the tandem Au(i)-cyclization/aryne ene reaction with respect to the nature of the resulting 5-arylmethyl substituent. Pleasingly, 2,5-disubstituted oxazoles 15a–m were generally afforded in good yields when pivalamide 14 was exposed to a wide range of *o*SAT aryne precursors 11a–m (Scheme 2). Importantly, the reaction was found to be equally tolerant of arynes substituted with electron-donating and electron-withdrawing groups (*e.g.* methylenedioxy 11b, 3,4-difluoro 11c, 3,4-dimethyl 11d, cyclopentyl 11e, 3-methoxy 11f, 5-methyl 11j and 4-fluoro 11k). Aromatic polycyclic aryne precursors were also amenable to the transformation, as 2,3-naphthyne (11g), 1,2-naphthyne (11h) and 9,10-phenanthryne (11i) all furnished the corresponding 2,5-disubstituted oxazoles 15g–i. It is noteworthy that under these reaction conditions, the bulkier 5-arylmethyl oxazoles 15h and 15i did not engage excess aryne to form the corresponding epoxyacenes 16, in contrast to the other oxazole derivatives investigated. As a result, propargyl amide 14 was treated with two equivalents of the bulkier precursors 11h and 11i to ensure complete consumption of the starting material. In comparison, lower yields of naphthalen-2-ylmethyl oxazole 15g were obtained, firstly due to the less hindered oxazole that does enter the cascade cycloaddition process, even at room temperature, and secondly as a result of the competitive and deleterious thia-Fries rearrangement of precursor 11g.^21^

Elsewhere, it was interesting to discover that the tandem Au(i)/aryne transformation was tolerant of reactive functional groups such as unprotected aldehydes and alcohols. For example, when 14 was exposed to aryne precursors 11l and 11m, the corresponding benzaldehyde- and benzylalcohol-containing oxazoles 15l and 15m were afforded in 45% and 78% yields, respectively. Finally, it is noteworthy that excellent regioselectivity was observed with several unsymmetrical arynes. For example, the 3-methoxy precursor 11f and 4-methoxybenzaldehyde derivative 11l both afforded products as a single regioisomer (>19 : 1 by ^1^H NMR spectroscopy), while 4-methoxybenzylalcohol 11m furnished the corresponding 5-arylmethyl oxazole 15m as a 10 : 1 product mixture. The major regioisomers of 15f, 15l and 15m were all identified with the methoxy substituent in a *meta*-relationship to the new oxazole methylene, which is consistent with the regioisomeric preference reported in ene reactions of similarly polarized arynes.^15^ Furthermore, deuterium incorporation was not observed when the reaction was conducted in acetonitrile-*d*_3_, which tentatively provides further support for a concerted process. As expected, the unsymmetrical arynes arising from precursors 11j and 11k, less electronically and sterically biased than the *ortho*-methoxy arynes, gave rise to more evenly distributed regioisomeric mixtures of products. For example, oxazole 15j was afforded as a 1 : 1 mixture of *meta*- and *para*-tolyl isomers, whereas 1,2-naphthyne 11h furnished oxazole 15h with a slight preference (1.7 : 1) for the less hindered naphthalen-2-ylmethyl isomer.

**Scheme 3:**
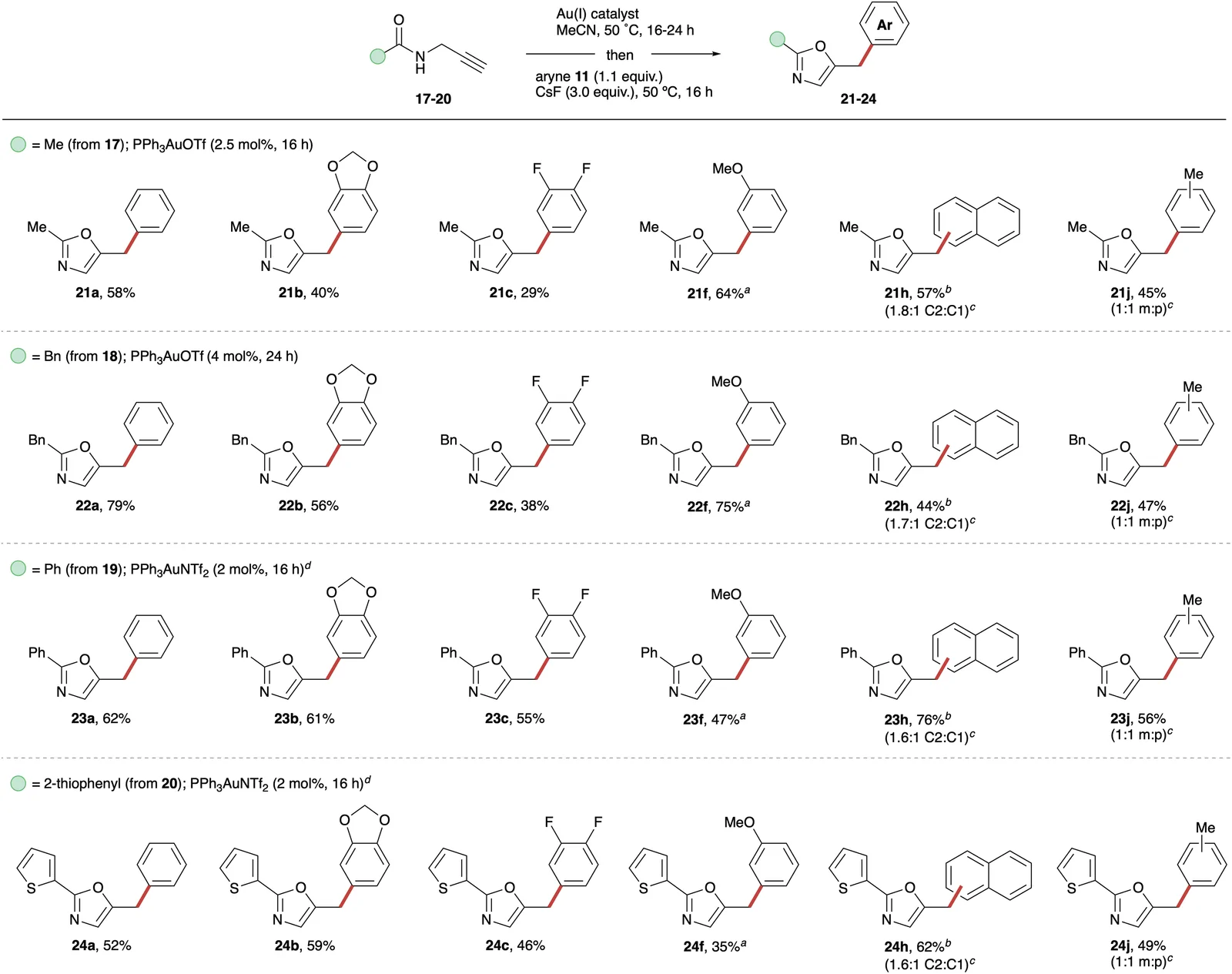
Evaluation of the substrate scope in the tandem Au(i) cyclization/aryne ene reaction with respect to propargyl amides and selected aryne derivatives. Yields of isolated products throughout. ^*a*^ Single regioisomer by ^1^H NMR spectroscopy. ^*b*^ 2.0 equivalents of aryne 11h and 5.0 equivalents of CsF added. ^*c*^ Ratio determined by ^1^H NMR spectroscopy. ^*d*^ CH_2_Cl_2_ used as a solvent for Au(i) cyclization, MeCN then added with aryne precursor and CsF.

Attention then turned towards varying the propargylic amide starting material. We were pleased to observe that the general transformation proceeded smoothly when different aliphatic (methyl, 17, benzyl, 18), aromatic (phenyl, 19) and heteroaromatic (2-thiophenyl, 20) propargylic amides were treated with a Au(i) catalyst and the *o*SAT precursor 11a, furnishing the corresponding 2,5-disubstituted oxazoles 17a–20a in good yields (Scheme 3). In agreement with previous reports,^11,14*b*^ it was found that the precise conditions for Au(i)-cyclization required modification depending on the nature of the amide group. For example, to effect complete cyclization, both the methyl (17) and benzyl (18) derivatives needed a higher catalyst loading and longer reaction times (2 mol% of PPh_3_AuOTf for 16 h and 4 mol% for 24 h, respectively) compared to pivalamide 14 (1 mol% for 4 h). Elsewhere, the more active PPh_3_AuNTf_2_ catalyst (Gagosz's catalyst)^22^ was required to promote cyclization of the aryl amides 19 and 20. Due to the instability of PPh_3_AuNTf_2_ in acetonitrile, a solvent switch to dichloromethane was also necessary to generate the corresponding methylene oxazoline intermediates. Acetonitrile was then added in combination with the aryne precursor and CsF to facilitate the subsequent aryne ene reactions, which proceeded smoothly in the acetonitrile–dichloromethane solvent mixture (*c.f.* no aryne ene reaction in neat dichloromethane; see Table 1, entry 7). It should be noted that the thiophene ring in the methylene oxazoline intermediate derived from amide 20 could potentially undergo an unwanted Diels–Alder reaction upon introduction of aryne; however, no evidence of the corresponding cycloadduct was detected. Next, we explored the substrate scope with respect to each of the propargyl amides 17–20, using a selection of substituted symmetrical and unsymmetrical *o*SAT precursors 11a–c, 11f, 11h and 11j. Pleasingly, good yields of the corresponding 5-arylmethyl oxazoles 21–24 were obtained for most of the combinations (see Scheme 3). The highest yields were generally afforded using benzyne (11a) – unpolarized and sterically unencumbered – whereas the strongly electron-withdrawing difluoro aryne derivative 11c typically gave lower yields, perhaps due to added instability of the aryne.

The diversity of 5-arylmethyl groups that have been incorporated into the product oxazoles 21–24 – varying electronics, sterics and functionality – provides good support for our cyclization/functionalization strategy. This compares favourably to previous approaches to synthesize 5-arylmethyl oxazoles *via* cyclization of non-terminal aryl-substituted propargylic amides (*i.e.* the reverse ‘functionalization/cyclization’ strategy) that report narrower substrate tolerance (only phenyl or electron-deficient aryl groups) and increased oxazine by-product formation (*i.e.* competing *6-endo-dig* cyclization).^11*d*^ Furthermore, as different propargylic amide substrates typically require modified conditions for the Au-cyclization, the more divergent method reduces the volume of optimization required for the overall process.

**Scheme 4:**
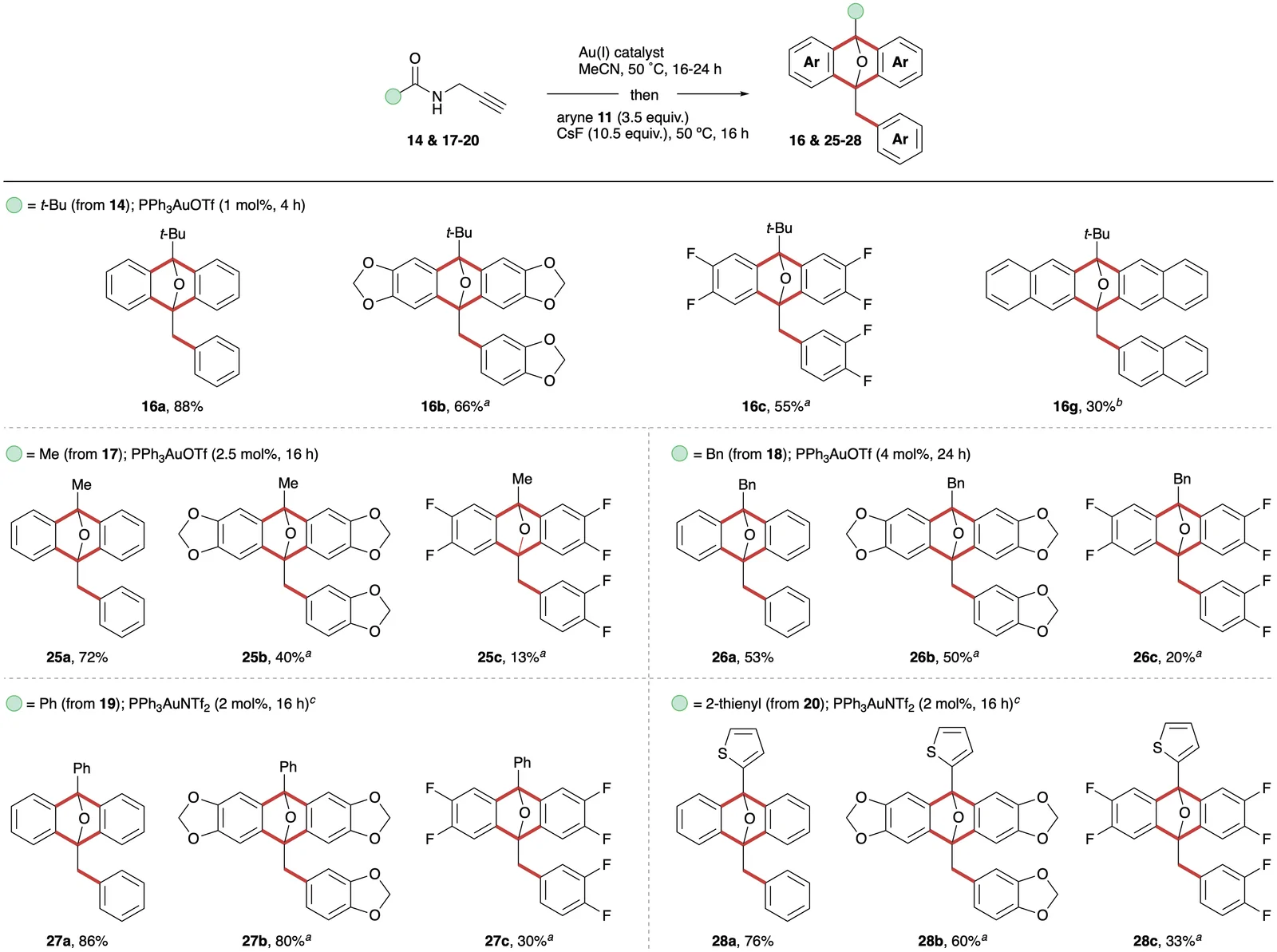
Evaluation of the substrate scope in the tandem Au(i) cyclization/aryne ene reaction to form epoxyacenes. Yields of isolated products throughout. ^*a*^ 5 equivalents of aryne and 15 equivalents of CsF added. ^*b*^ Reaction conducted at 80 °C. ^*c*^ CH_2_Cl_2_ used as a solvent for Au(i) cyclization, MeCN then added with aryne precursor 11 and CsF.

With a one-pot Au(i) cyclization/aryne ene reaction for the synthesis of a wide range of 2,5-disubstituted oxazoles in hand, we turned our attention toward our second goal of accessing substituted epoxyacene derivatives from propargylic amides *via* extension to incorporate the cascade aryne cycloaddition sequence. Building on our promising initial finding with pivalamide 14 (see Table 1, entry 11), it was found that a slight reduction in the amount of benzyne precursor 11a (from 4.0 to 3.5 equivalents) still afforded 9-benzyl-10-(*tert*-butyl)-9,10-dihydro-9,10-epoxyanthracene 16a in an excellent 88% yield *via* the analogous one-pot methodology (Scheme 4). Pleasingly, alkoxy- and fluoro-substituted epoxyanthracenes (16b and 16c, respectively) were accessed using methylenedioxy (11b) and 3,4-difluoro (11c) aryne precursors. Here, a small increase in precursor was required (5.0 equivalents), presumably due to the heightened promiscuity of the electron-deficient arynes. It was particularly encouraging to observe that the 2,3-naphthyne precursor 11g furnished the corresponding 6,13-epoxypentacene 16g in 30% yield. Pentacene derivatives have found widespread application in organic electronics, such as organic light-emitting diodes (OLEDs), field emission organic light-emitting diodes (FE-OLEDs) and organic field-emitting transistors (OFETs),^23^ so this provides an intriguing new approach to access such materials. Here, a slight increase in temperature (80 °C) was required to drive the cycloaddition cascade to completion but also led to a reduction in the amount of the competing thia-Fries rearrangement, presumably by increasing the rate of triflate elimination. Elsewhere, the transformation was tolerant of a range of different propargylic amides, as treatment of 17–20 with aryne precursors 11a–c under analogous reaction conditions to those used for pivalamide 14 furnished the corresponding epoxyanthracenes 25–28 in generally good yields. Once again, the use of 3,4-difluoro aryne 11c led to lower product yields, with the methyl (25c) and thiophenyl (28c) derivatives proving difficult to isolate from by-products at this stage.^24^

**Scheme 5:**
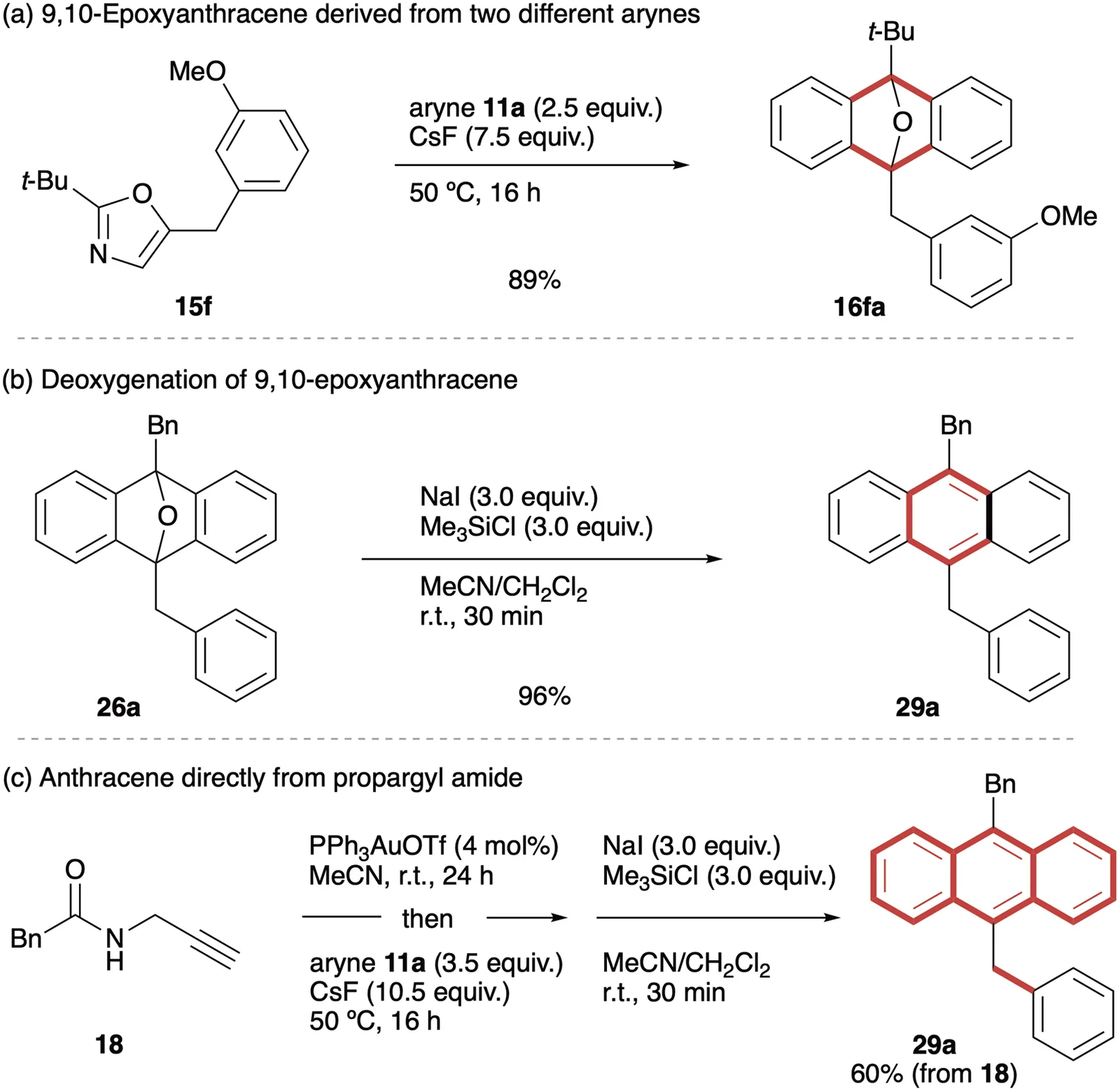
Extending the tandem Au(i) cyclization/aryne cycloaddition reactions: (a) mixed 9,10-epoxyanthracene 16da; (b) 9,10-disubstituted anthracene 29a*via* deoxygenation; and (c) telescoping deoxygenation to access anthracene 29a in one pot directly from propargyl amide 18.

Having successfully demonstrated the tandem Au(i)-cyclization/aryne cycloaddition process for the preparation of epoxyacenes directly from simple propargylic amides, we were interested to explore this transformation further. Firstly, a mixed 9,10-epoxyanthracene derivative 16fa was synthesized in excellent yield *via* the treatment of 2-benzyl-5-(3-methoxybenzyl)oxazole 15f with 2.5 equivalents of a second aryne precursor (11a) (Scheme 5a). Note that the use of oxazole 15f as the starting material in this reaction provided additional evidence for the intermediacy of oxazoles in the cascade process. Next, to access valuable PAH derivatives, we investigated procedures for the deoxygenation of the epoxyacenes.^25^ The original report by Bhatt and Reddy showed that zinc in refluxing acetic acid effected the desired transformation;^19*a*^ however, with a view to developing conditions that would be tolerant of a wider range of functionality, we focused on using *in situ* generated trimethylsilyl iodide, as described by Sygula and co-workers.^25*b*^ For example, when 9,10-dibenzyl-9,10-epoxyanthracene 26a was exposed to NaI and trimethylsilyl chloride in a 1 : 1 mixture of acetonitrile and dichloromethane, we were pleased to observe that the corresponding 9,10-disubstituted anthracene 29a was produced in near-quantitative yield (Scheme 5b). Finally, we looked at telescoping the whole process from propargylic amide through to the acene. Initial attempts to add NaI and TMSCl directly to the reaction mixture following epoxyanthracene formation were unsuccessful, due to the incompatibility of TMSCl and residual CsF. However, following a simple filtration to remove fluoride, the filtrate was exposed to the deoxygenation conditions to furnish anthracene 29a in 60% overall yield from benzyl amide 18 (Scheme 5c). This provides direct access to PAHs under mild conditions from readily available propargylic amide starting materials.

## Conclusions

We have described the development of a tandem Au(i) cyclization/aryne cycloaddition process for the synthesis of 5-arylmethyl oxazoles and substituted polyacene derivatives directly from simple propargylic amides. Access to these valuable and structurally diverse frameworks is achieved in a selective manner through straightforward control of aryne stoichiometry. Also translatable to the gram-scale, this approach is distinct from existing methods for the preparation of 5-arylmethyl oxazoles *via* cyclization of propargylic amides, as arylation occurs after ring-closure, which enables the introduction of electron-rich, electron-deficient and sterically demanding aryl groups. In addition, this divergent strategy reduces the number of structurally distinct substrates entering the Au(i)-cyclization step, which results in significant savings with respect to continued reaction optimization at this stage. The use of contemporary *o*SAT aryne precursors has also facilitated the development of mild conditions for the synthesis of a wide variety of 9,10-epoxyacenes. In contrast to previous reports of a cascade cycloaddition process between oxazoles and arynes, the incorporation of tandem Au-catalysis with *o*SAT precursors has enabled a wide range of PAHs to be prepared from readily available linear starting materials. Straightforward variation of the aryne enables tuning of the electronic and steric properties of the epoxyacenes and by extension the corresponding polyacenes resulting from deoxygenation, offering potential applications in organic materials chemistry. Finally, this aromatization step has been successfully telescoped to provide an operationally simple one-pot, three-step method for the synthesis of substituted polyacenes directly from propargylic amides.

## Author contributions

A. Y. R. W. and C. R. J. conceived and designed the experiments. A. Y. R. W. and Y. Y. performed the experiments and analysed the data. A. Y. R. W. and C. R. J. wrote the manuscript.

## Conflicts of interest

There are no conflicts to declare.

## Supplementary Material

QO-013-D6QO01223J-s001

## Data Availability

Details of the procedures and the corresponding datasets reported in this study are provided in the paper and its supplementary information (SI), and are available from the corresponding author on request. Supplementary information: all preparative procedures, characterisation data, and NMR spectra. See DOI: https://doi.org/10.1039/d6qo01223j.
