# Supplementary material for: One-pot divergent synthesis of 5-arylmethyl oxazoles and polyacenes from propargylic amides and arynes
Source: Org Chem Front. 2026 Sep 14;13(21):7615–24. doi: 10.1039/d6qo01223j (PMC13588082; doi:10.1039/d6qo01223j)

Supplementary Information (SI) for Organic Chemistry Frontiers.
This journal is © the Partner Organisations 2026

SUPPORTING INFORMATION

One-pot divergent synthesis of 5-arylmethyl oxazoles and polyacenes from propargylic amides and arynes

A. Yannic R. Werling, Yun Yang, Christopher R. Jones*

Department of Chemistry, Queen Mary University of London, Mile End Road, London, E1 4NS, UK.

Table of contents

1. **General information** 2
2. **Experimental procedures and compound characterisation** 3
3. **^{1}H and ^{13}C NMR spectra of isolated compounds** 41

1. General information

General: All chemicals were used directly as obtained from commercial sources without prior purification. Acetonitrile (MeCN) and dichloromethane were obtained from the MB SPS-80 solvent purification machine. Reactions requiring anhydrous conditions were carried out in oven-dried apparatus under nitrogen. 2-(Trimethylsilyl)phenyl trifluoromethanesulfonate **1** was purchased from commercial suppliers and used without additional purification.

Chromatography: Flash column chromatography was carried out using 40-63 µm silica gel or Biotage Isolera One flash purification system using KP-Sil or KP-NH (amine modified) Snap cartridges. Thin-layer chromatography (TLC) was performed on aluminium backed plates pre-coated with Silica Gel 60 F_{254} and visualized using a UV lamp (254 nm) or Dragendorff reagent stain.

Nuclear Magnetic Resonance Spectroscopy: ^{1}H, ^{13}C and ^{19}F NMR spectra were recorded on a Bruker Avance UltraShield AV400 or AV(III)400 (400 MHz, 376 MHz and 101 MHz). ^{1}H and ^{13}C NMR spectra were recorded in $CDCl_3$ and referenced to the residual solvent peak at 7.26 ppm and solvent peak at 77.2 ppm. ^{19}F NMR spectra were recorded in C_6F_6 referenced to the solvent peak at –164.9 ppm. Chemical shifts are quoted in parts per million (ppm) to 2 dp for ^{1}H NMR spectra and 1 dp for the ^{13}C and ^{19}F NMR spectra. Coupling constants (*J*) were measured in Hertz (Hz) to 1 dp. Spectral data are reported as follows: chemical shift, multiplicity (s = singlet, d = doublet, t = triplet, q = quartet, quint = quintet, sep = septet, dd = doublet of doublets, ddd = doublet of doublets of doublets, m = multiplet, br = broad), coupling constant (*J*) and integration.

Infrared (IR) spectroscopy: IR spectra were recorded as solids or neat liquids on the PerkinElmer Spectrum 65 FT-IR spectrometer fitted with a Universal ATR sampling accessory and are reported in wavenumbers (cm^{-1}) to the nearest integer.

Mass Spectrometry: High resolution mass spectra were acquired by nanospray ionization (NSI), time-of-flight (TOF) or atmospheric pressure chemical ionization (APCI) at the EPSRC UK National Mass Spectrometry Facility at Swansea University. Low resolution mass spectra were recorded on the Agilent 1100 series LC-MS (comprising a 6310 ion trap) under electrospray ionization (ESI) or on the Agilent GC-MS (comprising a 6890 GC and 5973 MSD) under electron ionization (EI).

Melting points: Melting points determined in open glass capillaries and are uncorrected.

2. Experimental Data

2.1. Synthesis of starting materials

Propargyl amides **14** and **17-20** were prepared according to literature procedures and data obtained were consistent with those reported.[1]

Aryne precursors **11b-l** were prepared according to literature procedures and data obtained were consistent with those reported.[2,3]

3-(Hydroxy(phenyl)methyl)-6-methoxy-2-(trimethylsilyl)phenyl trifluoromethanesulfonate, 11m

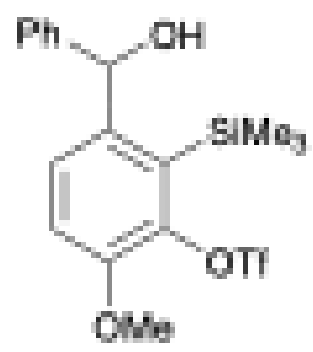

Magnesium (401 mg, 16.8 mmol) and bromobenzene (0.770 mL, 7.3 mmol) were dissolved in diethyl ether (24 mL) and stirred for 16 h. The solution was cooled to 0 ˚C and 3-formyl-6-methoxy-2-(trimethylsilyl)phenyl trifluoromethanesulfonate[3] **11l** (2.00 g, 5.61 mmol) was added and the reaction stirred for 10 minutes. The reaction mixture was washed with ammonium chloride (sat. aq., 25 mL) and brine (25 mL), dried over $MgSO_4$, filtered and concentrated *in vacuo*. The resulting residue was purified by silica gel flash column chromatography (10:1, PE:EA) to yield the desired product as an off-white solid (2.18 g, 5.03 mmol, 90 %).

^{1}H NMR (400 MHz, CDCl$_3$): δ_H 7.37-7.27 (m, 5H), 7.19 (d, *J* = 8.6, 1H), 6.96 (d, *J* = 8.6, 1H), 6.15 (d, *J* = 3.9, 1H), 3.83 (s, 3H), 2.13 (d, *J* = 3.9, 1H), 0.52 (s, 9H).

^{13}C NMR (101 MHz, CDCl$_3$): δ_C 150.0, 143.8, 142.7, 142.5, 134.6, 129.6, 128.4, 127.4, 126.5, 119.0 (q, *J* = 321), 114.2, 77.3, 55.7, 2.5.

^{19}F NMR (376 MHz, CDCl$_3$): δ_F –71.4.

IR (neat): ν_{max}/cm^{-1} 605, 620, 657, 678, 699, 729, 778, 835, 873, 908, 936, 1023, 1044, 1119, 1164, 1201, 1256, 1289, 1407, 1438, 1451, 1468, 1494, 1562, 1603, 2251, 2844, 2948, 3363.

HRMS (ASAP (solid)) [M-OH]+ calcd. for $C_{18}H_{20}F_3O_4SSi^+$ 417.0798, found: 417.0791.

---

[1] J. P. Weyrauch, A. S. K. Hashmi, A. Schuster, T. Hengst, S. Schetter, A. Littmann, M. Rudolph, M. Hamzic, J. Visus, F. Rominger, W. Frey, J. W. Bats, *Chem. Eur. J.* **2010**, *16*, 956.
[2] D. Peña, A. Cobas, D. Pérez, E. Guitián, *Synthesis*, **2002**, 1454.
[3] W. Sun, M. Uttendorfer, F. I. M. Idiris, A. Y. R. Werling, K. Siddiq, C. R Jones, *Chem. Commun*. **2023**, *59*, 11823.

Preparation of catalysts PPh_3AuOTf or PPh_3AuNTf_2

AgOTf or $AgNTf_2$ (18 mg, 0.07 mmol) and PPh_3AuCl (35 mg, 0.07 mmol) were dissolved in dry dichloromethane (1.4 mL). After stirring at rt for 10 min, filtration over celite to remove the silver chloride salts resulted in a pale solution. Quantitative recovery of PPh_3AuOTf or PPh_3AuNTf_2 was achieved upon removal of the reaction solvent and subsequent drying *in vacuo*.

2.2. Synthesis and characterization of 2,5-disubstituted oxazoles

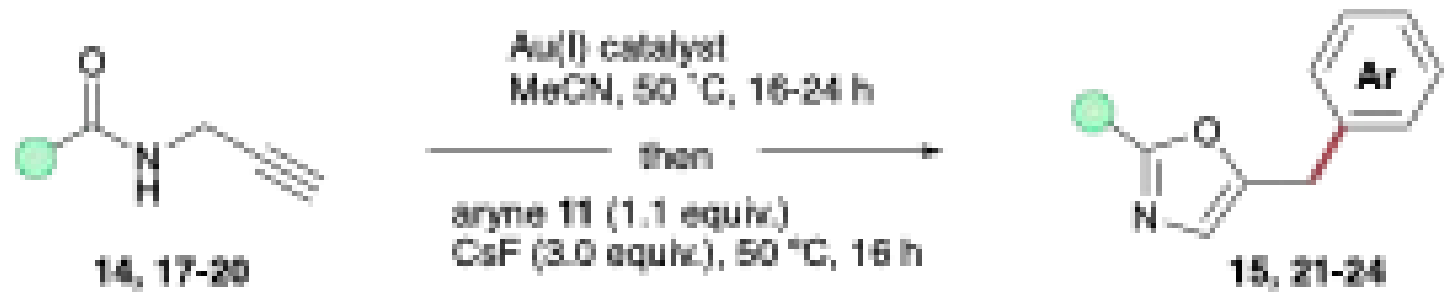

General procedure 1 for the synthesis of 2,5-disubstituted oxazoles (R = *t*-Bu, Me, Bn):

Propargylamide (1 eq) and triphenylphosphine gold(I) trifluoromethanesulfonate were dissolved in acetonitrile (0.15 M). The reaction mixture was stirred at the given temperature for between 4 to 24 h. Aryne precursor (1.1 equiv.) and cesium fluoride were then added and the reaction was stirred at 50 °C for 16 h. The solvent was removed *in vacuo* and the product was purified using silica gel flash column chromatography (*n*-hexane:ethyl acetate).

R group	Catalyst loading	Temp. (˚C)	Time (h)
t-Bu	1.0 mol%	20	4
Bn	4.0 mol%	50	24
Me	2.5 mol%	50	16

General procedure 2 for the synthesis of 2,5-disubstituted oxazoles (R = Ph, 2-thienyl):

Propargylamide (1 eq) and triphenylphosphine gold(I) bis[(trifluoromethyl)sulfonyl]amide (2.0 mol%) were dissolved in dichloromethane (0.30 M). The reaction mixture was stirred at 50 ˚C for 16 h. Aryne precursor (1.1 equiv.), cesium fluoride and acetonitrile (0.15 M) were then added and the reaction was stirred at 50 °C for 16 h. The solvent was removed *in vacuo* and the product was purified using silica gel flash column chromatography (*n*-hexane:ethyl acetate).

5-Benzyl-2-(*tert*-butyl)oxazole, 15a

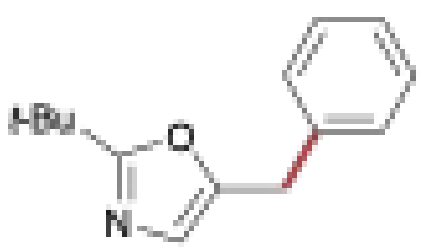

Prepared according to general procedure 1 for the synthesis of 2,5-disubstituted oxazoles (R = *t*-Bu): using *N*-propargylpivalamide **14** (44.7 mg, 0.321 mmol), triphenylphosphine gold(I) trifluoromethanesulfonate (1.93 mg, 3.21 µmol, 1.0 mol %), 2-(trimethylsilyl)phenyl trifluoromethanesulfonate **11a** (105 mg, 0.352 mmol) and cesium fluoride (160 mg, 1.06 mmol). The crude reaction mixture was purified by silica gel flash chromatography to afford the title compound (46.8 mg, 0.217 mmol, 68%) as a colourless oil; R_f 0.48 (2:1 *n*-hexane:ethyl acetate).

^{1}H NMR (400 MHz, CDCl$_3$): δ_H 7.34-7.28 (m, 2H), 7.26-7.21 (m, 3H), 6.57 (s, 1H), 3.96 (s, 2H), 1.35 (s, 9H).

^{13}C NMR (101 MHz, CDCl$_3$): δ_C 170.8, 150.6, 137.1, 128.7, 126.9, 126.9, 123.0, 33.8, 32.2, 28.7.

IR (neat): ν_{max}/cm^{-1} 2971, 1561, 1496, 1455, 1365, 1219, 1139, 1109, 1030, 986, 828, 750, 708, 695, 649.

HRMS (NSI) [M+H]+ calcd. for $C_{14}H_{18}NO$ 216.1383, found: 216.1383.

5-(Benzo[d][1,3]dioxol-5-ylmethyl)-2-(*tert*-butyl)oxazole, 15b

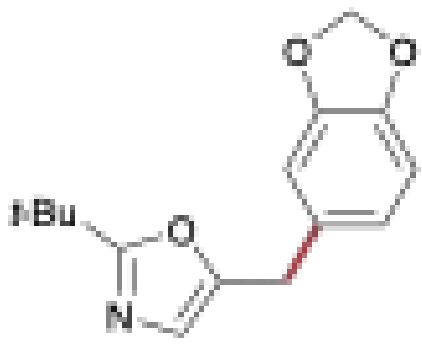

Prepared according to general procedure 1 for the synthesis of 2,5-disubstituted oxazoles (R = *t*-Bu): using *N*-propargylpivalamide **14** (43.7 mg, 0.314 mmol), triphenylphosphine gold(I) trifluoromethanesulfonate (1.91 mg, 3.14 µmol, 1.0 mol %), 6-(trimethylsilyl)benzo[d][1,3]dioxol-5-yl trifluoromethanesulfonate **11b** (118 mg, 0.345 mmol) and cesium fluoride (156 mg, 1.04 mmol). The crude reaction mixture was purified by silica gel flash chromatography to afford the title compound (48.6 mg, 0.225 mmol, 72%) as a yellow oil; R_f 0.38 (2:1 *n*-hexane:ethyl acetate).

^{1}H NMR (400 MHz, CDCl$_3$): δ_H 6.74 (d, J = 7.9, 1H), 6.71 (d, J = 1.5, 1H), 6.68 (dd, J = 7.9, 1.5, 1H), 6.57 (s, 1H), 5.93 (s, 2H), 3.96 (s, 2H), 1.45 (s, 9H).

^{13}C NMR (101 MHz, CDCl$_3$): δ_C 170.8, 150.8, 148.0, 156.5, 130.8, 122.8, 121.7, 109.2, 108.4, 101.1, 33.8, 31.9, 28.7.

IR (neat): ν_{max}/cm^{-1} 1503, 1489, 1443, 1243, 1185, 1139, 1109, 1098, 1037, 986, 940, 925, 810, 772, 739.

HRMS (NSI) [M+H]+ calcd. for $C_{15}H_{18}NO_3$ 260.1281, found: 260.1282.

2-(*tert*-Butyl)-5-(3,4-difluorobenzyl)oxazole, 15c

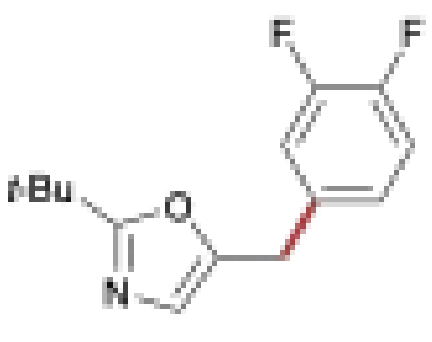

Prepared according to general procedure 1 for the synthesis of 2,5-disubstituted oxazoles (R = *t*-Bu): using *N*-propargylpivalamide **14** (20.9 mg, 0.150 mmol), triphenylphosphine gold(I) trifluoromethanesulfonate (0.91 mg, 1.50 µmol, 1.0 mol %), 4,5-difluoro-2-(trimethylsilyl)phenyl

trifluoromethanesulfonate **11c** (75.2 mg, 0.225 mmol) and cesium fluoride (85.4 mg, 0.563 mmol). The crude reaction mixture was purified by silica gel flash chromatography to afford the title compound (15.2 mg, 0.0605 mmol, 40%) as a yellow oil; R_f 0.37 (2:1 *n*-hexane:ethyl acetate).

^{1}H NMR (400 MHz, $CDCl_3$): δ_H 7.10 (m, 1H), 7.03 (m, 1H), 6.94 (m, 1H), 6.62 (s, 1H), 3.92 (s, 2H), 1.35 (s, 9H).

^{13}C NMR (101 MHz, $CDCl_3$): δ_C 171.2, 150.8 (dd, $J_{C\text{-}F}$ 142, 12.6), 149.6, 149.2 (dd, $J_{C\text{-}F}$ 141, 12.4), 134.1 (dd, $J_{C\text{-}F}$ 5.1, 3.2), 124.6 (dd, $J_{C\text{-}F}$ 6.3, 3.4), 123.3, 117.6 (d, $J_{C\text{-}F}$ 17.5), 117.5 (d, $J_{C\text{-}F}$ 17.1), 33.8, 31.3, 28.7.

^{19}F NMR (376 MHz, $CDCl_3$): δ_F –137.6 (d, $J_{F\text{-}F}$ 21.2, 1F), –140.7 (d, $J_{F\text{-}F}$ 21.2, 1F).

IR (neat): ν_{max}/cm^{-1} 1610, 1561, 1518, 1436, 1283, 1210, 1139, 1113, 988, 820, 781, 764, 746, 734, 640.

HRMS (NSI) [M+H]+ calcd. for $C_{14}H_{16}F_2NO$ 252.1195, found: 252.1195.

2-(*tert*-Butyl)-5-(3,4-dimethylbenzyl)oxazole, 15d

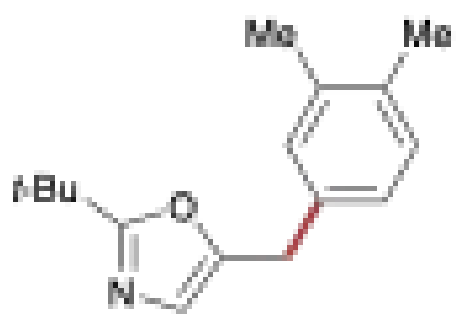

Prepared according to general procedure 1 for the synthesis of 2,5-disubstituted oxazoles (R = *t*-Bu): using *N*-propargylpivalamide **14** (20.9 mg, 0.150 mmol), triphenylphosphine gold(I) trifluoromethanesulfonate (0.91 mg, 1.50 μmol, 1.0 mol %), %), 4,5-dimethyl-2-(trimethylsilyl)phenyl trifluoromethanesulfonate **11d** (58.6 mg, 0.180 mmol) and cesium fluoride (62.7 mg, 0.413 mmol). The crude reaction mixture was purified by silica gel flash chromatography to afford the title compound (16.6 mg, 0.0682 mmol, 45%) as a colourless oil; R_f 0.51 (2:1 *n*-hexane:ethyl acetate).

^{1}H NMR (400 MHz, $CDCl_3$): δ_H 7.07 (d, J = 7.6, 1H), 7.00 (d, J = 1.2 , 1H), 6.96 (dd, J = 7.6 1.2, 1H), 6.56 (s, 1H), 3.89 (s, 2H), 2.24 (s, 6H), 1.36 (s, 9H).

^{13}C NMR (101 MHz, $CDCl_3$): δ_C 170.6, 151.0, 136.9, 135.0, 134.5, 130.1, 129.9, 126.1, 122.8, 33.8, 31.7, 28.7, 19.8, 19.4.

IR (neat): ν_{max}/cm^{-1} 2971, 1561, 1505, 1457, 1365, 1138, 1109, 1023, 986, 821, 765, 746, 730, 640, 624.

HRMS (ESI) [M+H]+ calcd. for $C_{16}H_{21}NO$ 244.1701, found: 244.1703.

2-(*tert*-Butyl)-5-((2,3-dihydro-1H-inden-5-yl)methyl)oxazole, 15e

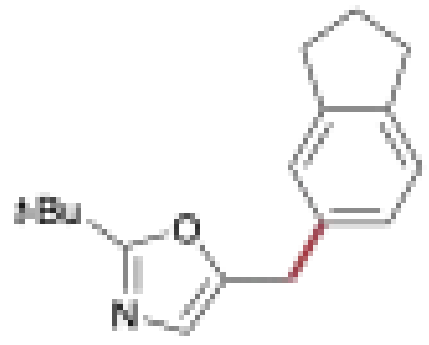

Prepared according to general procedure 1 for the synthesis of 2,5-disubstituted oxazoles (R = *t*-Bu): using *N*-propargylpivalamide **14** (20.9 mg, 0.150 mmol), triphenylphosphine gold(I) trifluoromethanesulfonate (0.91 mg, 1.50 µmol, 1.0 mol %), 6-(trimethylsilyl)-2,3-dihydro-1H-inden-5-yl trifluoromethanesulfonate **11e** (60.9 mg, 0.180 mmol) and cesium fluoride (62.7 mg, 0.413 mmol). The crude reaction mixture was purified by silica gel flash chromatography to afford the title compound (18.2 mg, 0.0713 mmol, 48%) as a pale yellow oil; Rf 0.51 (2:1 *n*-hexane:ethyl acetate).

^{1}H NMR (400 MHz, $CDCl_3$): δ_H 7.16 (d, J = 7.7, 1H), 7.09 (s, 1H), 7.00 (d, J = 7.7, 1H), 6.57 (s, 1H), 3.92 (s, 2H), 2.89 (t, J = 7.4, 4H), 2.07 (quin, J = 7.4, 2H), 1.36 (s, 9H).

^{13}C NMR (101 MHz, $CDCl_3$): δ_C 170.6, 151.1, 144.8, 142.8, 134.9, 126.5, 124.8, 124.5, 122.8, 33.7, 32.9, 32.6, 32.0, 28.7, 25.6.

IR (neat): ν_{max}/cm^{-1} 2968, 2906, 1561, 1492, 1460, 1438, 1364, 1217, 1207, 1139, 1109, 986, 822, 767, 740.

HRMS (ESI) [M+H]+calcd. for $C_{17}H_{21}NO$ 256.1701, found: 256.1703.

2-(*tert*-Butyl)-5-(3-methoxybenzyl)oxazole, 15f

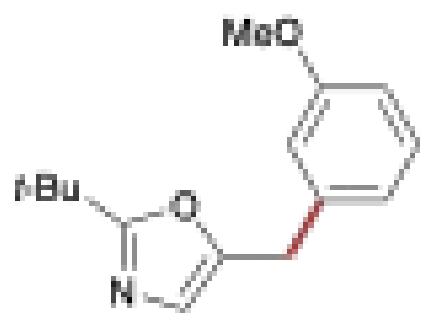

Prepared according to general procedure 1 for the synthesis of 2,5-disubstituted oxazoles (R = *t*-Bu): using *N*-propargylpivalamide **14** (20.9 mg, 0.150 mmol), triphenylphosphine gold(I) trifluoromethanesulfonate (0.91 mg, 1.50 µmol, 1.0 mol %), 3-methoxy-2-(trimethylsilyl)phenyl trifluoromethanesulfonate **11f** (59.1 mg, 0.180 mmol) and cesium fluoride (62.7 mg, 0.413 mmol). The crude reaction mixture was purified by silica gel flash chromatography to afford the title compound (22.4 mg, 0.0913 mmol, 61%) as a colourless oil; R_f 0.45 (2:1 *n*-hexane:ethyl acetate).

^{1}H NMR (400 MHz, $CDCl_3$): δ_H 7.22 (t, J = 7.8, 1H), 6.83-6.76 (m, 3H), 6.60 (s, 1H), 3.93 (s, 2H), 3.78 (s, 3H), 1.37 (s, 9H).

^{13}C NMR (101 MHz, $CDCl_3$): δ_C 170.8, 160.0, 150.4, 138.7, 129.7, 123.0, 121.1, 114.4, 112.4, 55.3, 33.8, 32.1, 28.7.

IR (neat): ν_{max}/cm^{-1} 1600, 1586, 1561, 1490, 1455, 1436, 1261, 1140, 1109, 1046, 987, 760, 744, 730, 690.

HRMS (ESI) [M+H]+ calcd. for $C_{15}H_{19}NO_2$ 246.1489, found: 246.1526.

2-(*tert*-Butyl)-5-(naphthalen-2-ylmethyl)oxazole, 15g

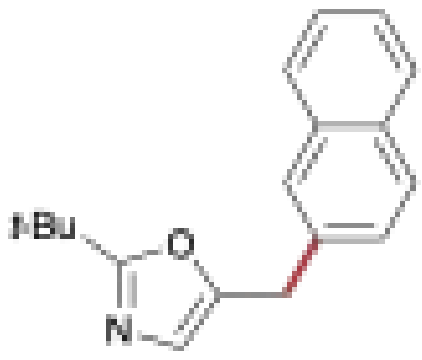

Prepared according to general procedure 1 for the synthesis of 2,5-disubstituted oxazoles (R = *t*-Bu): using *N*-propargylpivalamide **14** (22.3 mg, 0.160 mmol), triphenylphosphine gold(I) trifluoromethanesulfonate (0.97 mg, 1.60 µmol, 1.0 mol %), 3-(trimethylsilyl)naphthalen-2-yl triflate **11g** (62.7 mg, 0.180 mmol) and cesium fluoride (60.8 mg, 0.400 mmol). *Note: aryne reaction conducted at room temperature*. The crude reaction mixture was purified by silica gel flash chromatography to afford the title compound (12.7 mg, 0.048 mmol, 30%) as a colourless solid; R_f 0.45 (2:1 *n*-hexane:ethyl acetate).

^{1}H NMR (400 MHz, $CDCl_3$): δ_H 7.83-7.77 (m, 3H), 7.67 (bs, 1H), 7.48-7.45 (m, 2H), 7.36 (dd, *J* = 8.4, 1.7, 1H), 6.62 (s, 1H), 4.12 (s, 2H), 1.36 (s, 9H).

^{13}C NMR (101 MHz, $CDCl_3$): δ_C 170.9, 150.6, 134.6, 133.7, 132.5, 128.4, 127.8, 127.7, 127.3, 127.1, 126.3, 125.8, 123.2, 33.8, 32.4, 28.7.

IR (neat): ν_{max}/cm^{-1} 3030, 2921, 1727, 1684, 1495, 1450, 1250, 1064, 985, 709, 691.

HRMS (NSI) [M+H]+ calcd. for $C_{18}H_{20}NO$ 266.1539, found: 266.1440.

2-(*tert*-Butyl)-5-(naphthalen-2-ylmethyl)oxazole, 15h & 2-(*tert*-butyl)-5-(naphthalen-1-ylmethyl)oxazole, 15h'

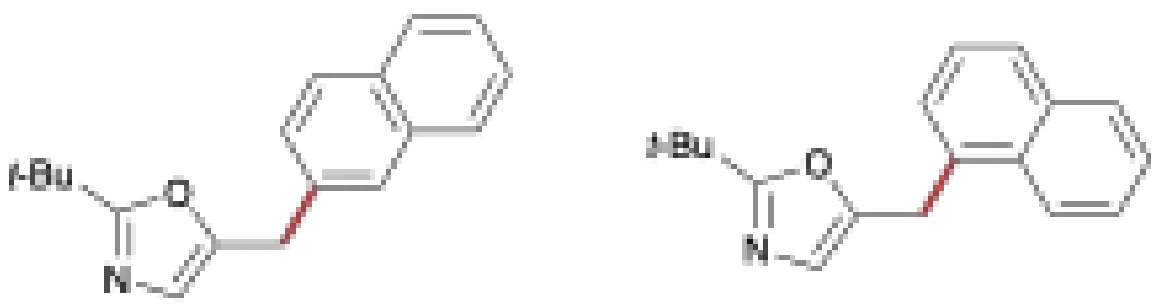

Prepared according to general procedure 1 for the synthesis of 2,5-disubstituted oxazoles (R = *t*-Bu): using *N*-propargylpivalamide **14** (20.9 mg, 0.150 mmol), triphenylphosphine gold(I) trifluoromethanesulfonate (0.91 mg, 1.50 µmol, 1.0 mol %), 1-(trimethylsilyl)naphthalen-2-yl trifluoromethanesulfonate **11h** (104 mg, 0.300 mmol, 2.0 equiv.) and cesium fluoride (120 mg, 0.750 mmol, 5.0 equiv.). The crude reaction mixture was purified by silica gel flash chromatography to afford the title compound as an inseparable 1.7:1 mixture **15h:15h'** (28.0 mg, 0.106 mmol, 70%) as a pale yellow oil; R_f 0.44 (2:1 *n*-hexane:ethyl acetate).

Data provided for a 1.7:1 regioisomeric mixture of **15h to 15h'**:

^{1}H NMR (400 MHz, CDCl$_3$): δ_H 8.04-8.00 (m, 0.4H), 7.88-7.85 (m, 0.4H), 7.83-7.76 (m, 2.2H), 7.66 (bs, 0.6H), 7.54-7.39 (m, 2.4H), 7.36 (dd, *J* = 8.4, 1.7, 0.6H), 7.31 (d, *J* = 6.9, 0.4H), 6.62 (s, 0.6H), 6.50 (s, 0.4H), 4.39 (s, 0.8H), 4.11 (s, 1.2H), 1.35 (s, 9H).

^{13}C NMR (101 MHz, CDCl$_3$): δ_C 170.8, 170.5, 150.4, 150.1, 134.4, 133.8, 133.5, 133.0, 132.3, 131.8, 128.7, 128.2, 127.7 (2xC), 127.6, 127.1, 126.9, 126.8, 126.2, 125.7, 125.7, 125.5, 123.6, 123.3, 123.0, 33.7, 33.6, 32.2 (2xC), 29.5, 28.6.

IR (neat): ν_{max}/cm^{-1} 2971, 1556, 1509, 1365, 1219, 1139, 1107, 986, 860, 821, 800, 792, 779, 763, 752.

HRMS (NSI) [M+H]+ calcd. for $C_{18}H_{20}NO$ 266.1539, found: 266.1539.

2-(*tert*-Butyl)-5-(phenanthren-9-ylmethyl)oxazole, 15i

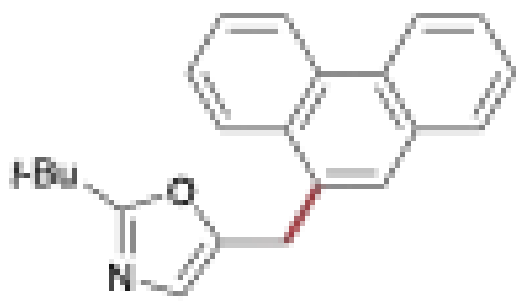

Prepared according to general procedure 1 for the synthesis of 2,5-disubstituted oxazoles (R = *t*-Bu): using *N*-propargylpivalamide **14** (20.9 mg, 0.150 mmol), triphenylphosphine gold(I) trifluoromethanesulfonate (0.91 mg, 1.50 µmol, 1.0 mol %), 10-(trimethylsilyl)phenanthren-9-yl trifluoromethanesulfonate **11i** (119 mg, 0.300 mmol, 2.0 equiv.) and cesium fluoride (120 mg, 0.750 mmol, 5.0 equiv.). The crude reaction mixture was purified by silica gel flash chromatography to afford the title compound (30.5 mg, 0.0967 mmol, 64%) as a yellow solid; R_f 0.41 (2:1 *n*-hexane:ethyl acetate).

^{1}H NMR (400 MHz, CDCl$_3$): δ_H 8.73 (dd, *J* = 7.6 1.3, 1H), 8.65 (d, *J* = 8.2, 1H), 8.05 (dd, *J* = 8.0, 1.3, 1H) 7.80 (dd, *J* = 7.8 1.3, 1H), 7.68-7.54 (m, 5H), 6.57 (s, 1H), 4.43 (s, 2H), 1.37 (s, 9H).

^{13}C NMR (101 MHz, CDCl$_3$): δ_C 170.7, 150.0, 131.7, 131.4, 131.0, 130.9, 130.2, 128.4, 127.6, 126.9 (2xC), 126.7, 126.6, 124.5, 123.7, 123.3, 122.6, 33.8, 30.1, 28.7.

IR (neat): ν_{max}/cm^{-1} 2971, 1560, 1453, 1139, 1109, 1040, 986, 826, 792, 774, 747, 731, 722, 626, 616.

HRMS (NSI) [M+H]+ calcd. for $C_{22}H_{21}NO$ 316.1701, found: 316.1705.

2-(*tert*-Butyl)-5-(3-methylbenzyl)oxazole 15j & 2-(*tert*-butyl)-5-(4-methylbenzyl)oxazole, 15j’

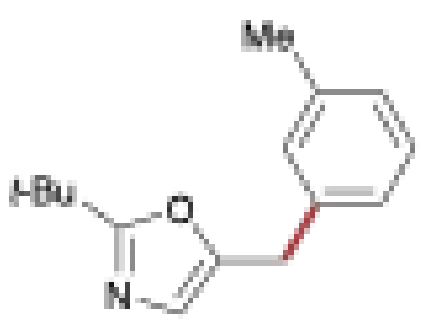

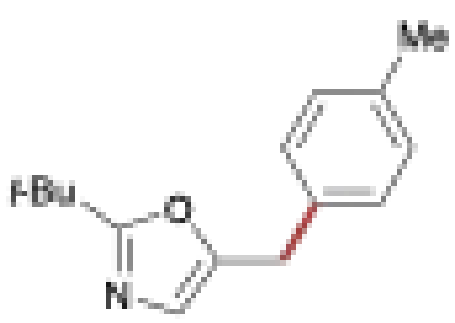

Prepared according to general procedure 1 for the synthesis of 2,5-disubstituted oxazoles (R = *t*-Bu): using *N*-propargylpivalamide **14** (41.6 mg, 0.299 mmol), triphenylphosphine gold(I) trifluoromethanesulfonate (1.81 mg, 2.99 µmol, 1.0 mol %), 4-methyl-2-(trimethylsilyl)phenyl trifluoromethanesulfonate **11j** (103 mg, 0.329 mmol) and cesium fluoride (150 mg, 0.986 mmol). The crude reaction mixture was purified by silica gel flash chromatography to afford the title compounds as an inseparable 1:1 mixture (42.0 mg, 0.183 mmol, 61%) as a yellow oil; R_f 0.49 (2:1 *n*-hexane:ethyl acetate).

Data provided for a 1:1 regioisomeric mixture of **15j**:**15j'**

^{1}H NMR (400 MHz, CDCl$_3$): δ_H 7.22-7.18 (m, 0.5H), 7.12 (s, 2H) 7.07-7.01 (m, 1.5H), 6.57 (s, 0.5H), 6.56 (s, 0.5H), 3.92 (s, 1H), 3.91 (s, 1H), 2.33 (s, 3H), 1.35 (s, 9H).

^{13}C NMR (101 MHz, CDCl$_3$): δ_C 170.8, 170.7, 150.9, 150.8, 138.4, 137.0, 136.4, 134.0, 129.5, 129.4 (2xC), 128.6 (2xC), 128.6, 127.6, 125.8, 122.9, 122.8, 33.8 (2xC), 32.1, 31.8, 28.7 (2xC), 21.5, 21.2.

IR (neat): ν_{max}/cm^{-1} 2971, 2931, 1561, 1516, 1460, 1365, 1218, 1139, 1110, 986, 822, 794, 759, 741, 690.

HRMS (ESI) [M+H]+ calcd. for $C_{15}H_{20}NO$ 230.1539, found: 230.1532.

2-(*tert*-butyl)-5-(4-fluorobenzyl)oxazole 15k & 2-(*tert*-butyl)-5-(3-fluorobenzyl)oxazole, 15k'

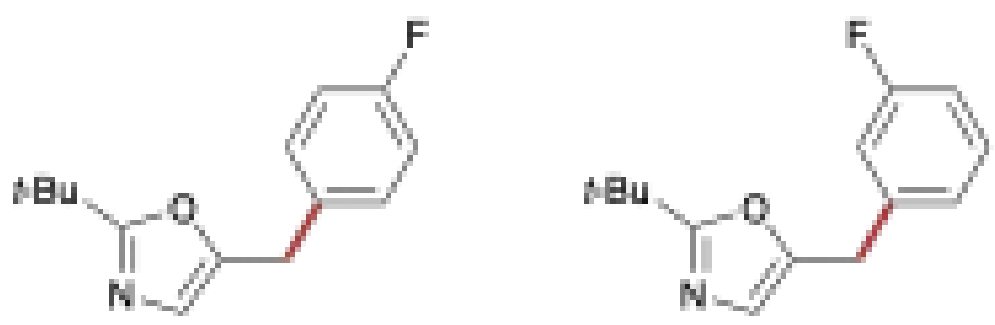

Prepared according to general procedure 1 for the synthesis of 2,5-disubstituted oxazoles (R = *t*-Bu): using *N*-propargylpivalamide **14** (20.9 mg, 0.150 mmol), triphenylphosphine gold(I) trifluoromethanesulfonate (0.91 mg, 1.50 µmol, 1.0 mol %), 4-fluoro-2-(trimethylsilyl)phenyl trifluoromethanesulfonate **11k** (52.2 mg, 0.165 mmol) and cesium fluoride (60.0 mg, 0.375 mmol). The crude reaction mixture was purified by silica gel flash chromatography to afford the title compounds as an inseparable 2.5:1 mixture (13.8 mg, 0.0592 mmol, 39%) as a yellow oil; R_f 0.42 (2:1 *n*-hexane:ethyl acetate).

Data provided for a 2.5:1 regioisomeric mixture of **15k**:**15k'**

NMR spectroscopic data for major isomer **15k**:

^{1}H NMR (400 MHz, CDCl$_3$): δ_H 7.19 (m, 2H), 7.00 (m, 2H), 6.57 (s, 1H), 3.93 (s, 2H), 1.35 (s, 9H).

^{13}C NMR (101 MHz, CDCl$_3$): δ_C 170.9, 161.9 (d, J_{C-F} 245), 150.4, 132.7 (d, J_{C-F} 3.0), 130.2 (d, J_{C-F} 7.7), 123.0, 115.6 (d, J_{C-F} 21.7), 33.8, 31.4, 28.7.

^{19}F NMR (376 MHz, CDCl$_3$): δ_F –116.2.

NMR spectroscopic data for minor isomer **15k'**:

^{1}H NMR (400 MHz, CDCl$_3$): δ_H 7.29-7.25 (m, 1H), 7.0 (m, 1H), 6.95-6.93 (m, 2H), 6.62 (s, 1H), 3.95 (s, 2H), 1.35 (s, 9H).

^{13}C NMR (101 MHz, CDCl$_3$): δ_C 171.1, 163.1 (d, $J_{C\text{-}F}$ 246), 149.8, 139.6 (d, $J_{C\text{-}F}$ 7.6), 130.2 (d, $J_{C\text{-}F}$ 8.4), 124.3 (d, $J_{C\text{-}F}$ 2.9),123.3, 115.8 (d, $J_{C\text{-}F}$ 19.4), 113.9 (d, $J_{C\text{-}F}$ 20.9), 33.8, 31.8, 28.7.

^{19}F NMR (376 MHz, CDCl$_3$): δ_F –113.1.

IR (neat): ν_{max}/cm^{-1} 2973, 1604, 1561, 1510, 1488, 1461, 1366, 1223, 1158, 1140, 1111, 987, 824, 770, 745.

HRMS (ESI) [M+H]+ calcd. for $C_{14}H_{17}FNO$ 234.1294, found: 234.1291.

2-((2-(*tert*-Butyl)oxazol-5-yl)methyl)-4-methoxybenzaldehyde, 15l

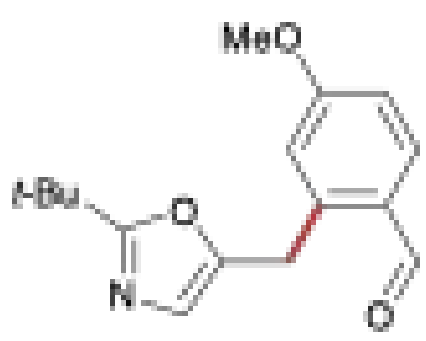

Prepared according to general procedure 1 for the synthesis of 2,5-disubstituted oxazoles (R = *t*-Bu): using *N*-propargylpivalamide **14** (105 mg, 0.750 mmol), triphenylphosphine gold(I) trifluoromethanesulfonate (4.50 mg, 7.40 µmol, 1.0 mol %), 3-formyl-6-methoxy-2-(trimethylsilyl)phenyl trifluoromethanesulfonate **11l** (401 mg, 1.13 mmol) and cesium fluoride (427 mg, 2.82 mmol). The crude reaction mixture was purified by silica gel flash chromatography to afford the title compound (91.5 mg, 0.335 mmol, 45%) as a pale yellow solid; R_f 0.22 (2:1 *n*-hexane:ethyl acetate).

^{1}H NMR (400 MHz, CDCl$_3$): δ_H 10.04 (s, 1H), 7.76 (d, J = 8.5, 1H), 6.90 (dd, J = 8.5, 1.4, 1H), 6. 73 (d, J = 1.4, 1H), 6.58 (s, 1H), 4.40 (s, 2H), 3.82 (s, 3H), 1.32 (s, 9H).

^{13}C NMR (101 MHz, CDCl$_3$): δ_C 191.0, 170.8, 163.9, 149.5, 141.7, 136.3, 127.4, 123.4, 116.6, 112.4, 55.6, 33.7, 28.9, 28.6.

IR (neat): ν_{max}/cm^{-1} 2950, 2898, 1695, 1589, 1552, 1469, 1407, 1377, 1300, 1201, 976, 834, 740.

HRMS (ESI) [M+H]+ calcd. for $C_{16}H_{19}NO_3$ 274.1438, found: 274.1493.

(2-((2-(*tert*-Butyl)oxazol-5-yl)methyl)-4-methoxyphenyl)(phenyl)methanol, 15m & (3-((2-(*tert*-butyl)oxazol-5-yl)methyl)-4-methoxyphenyl)(phenyl)methanol, 15m'

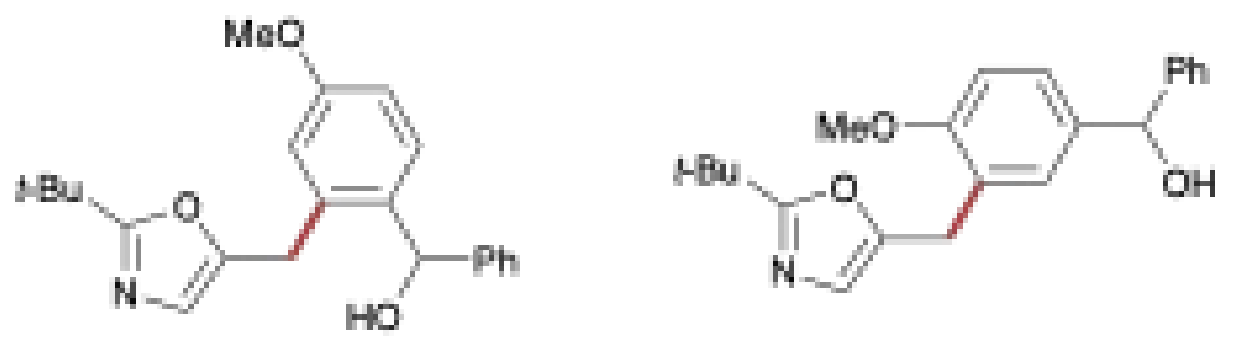

Prepared according to general procedure 1 for the synthesis of 2,5-disubstituted oxazoles (R = *t*-Bu): using *N*-propargylpivalamide **14** (20.9 mg, 0.150 mmol), triphenylphosphine gold(I) trifluoromethanesulfonate (0.91 mg, 1.50 µmol, 1.0 mol %), 3-(hydroxy(phenyl)methyl)-6-methoxy-2-(trimethylsilyl)phenyl trifluoromethanesulfonate **11m** (97.8 mg, 0.225 mmol) and cesium fluoride (85.4 mg, 0.563 mmol). The crude reaction mixture was purified by silica gel flash chromatography to afford the title compound as an inseparable 10:1 mixture **15m:15m'** (41.1 mg, 0.117 mmol, 78%) as a yellow oil; R_f 0.22 (2:1 *n*-hexane:ethyl acetate).

Data provided for a 10:1 regioisomeric mixture of **15m to 15m'**:

^{1}H NMR (400 MHz, CDCl$_3$): δ_H 7.29-7.18 (m, 5H), 7.08 (d, *J* = 2.0, 0.1H), 6.78 (d, *J* = 8.4, 0.1H), 6.76 (dd, *J* = 8.6 2.6, 0.9H), 6.66 (d, *J* = 2.6, 0.9H), 6.40 (s, 0.1H), 6.32 (s, 0.9H), 5.98 (s, 0.9H), 5.71 (s, 0.1H), 3.93 (s, 0.9H), 3.90 (d, *J* = 4.7, 1.8H), 3.87 (s, 0.2H), 3.76 (s, 0.3H), 3.72 (s, 2.7H), 2.62 (bs, 0.9H), 1.29 (s, 8.1H), 1.28 (s, 0.9H)

^{13}C NMR (101 MHz, CDCl$_3$): δ_C 170.7 (2xC), 159.3, 156.8, 150.3, 150.1, 144.3, 143.4, 136.3, 134.0, 129.2, 128.5, 128.5, 128.3, 127.5, 127.5, 126.8, 126.5, 126.4, 125.7, 123.1, 122.7, 115.8, 112.5, 110.5, 75.8, 72.8, 55.6, 55.3, 33.7, 33.7, 29.1, 29.0, 28.7, 28.5.

IR (neat): ν_{max}/cm^{-1} 3345, 2948, 2845, 1601, 1556, 1467, 1437, 1292, 1257, 1201, 1041, 835, 730.

HRMS (ASAP) [M+H]+ calcd. for $C_{22}H_{26}NO_3$ 352.1913, found: 352.1912.

5-Benzyl-2-methyloxazole, 21a

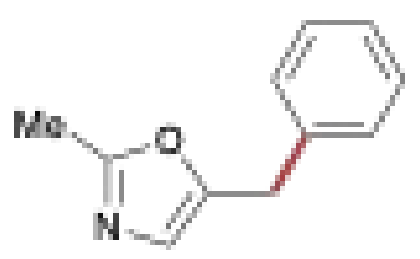

Prepared according to general procedure 1 for the synthesis of 2,5-disubstituted oxazoles (R = Me): using *N*-(prop-2-yn-1-yl)acetamide **17** (15.5 mg, 0.160 mmol), triphenylphosphine gold(I) trifluoromethanesulfonate (2.43 mg, 4.00 µmol, 2.5 mol %), 2-(trimethylsilyl)phenyl trifluoromethanesulfonate **11a** (52.4 mg, 0.176 mmol) and cesium fluoride (66.7 mg, 0.439 mmol). The crude reaction mixture was purified by silica gel flash chromatography to afford the title compound (16.1 mg, 0.093 mmol, 58%) as a colourless oil; R_f 0.22 (2:1 *n*-hexane:ethyl acetate).

^{1}H NMR (400 MHz, CDCl$_3$): δ_H 7.33-7.30 (m, 2H), 7.27-7.22 (m, 3H), 6.61 (s, 1H), 3.93 (s, 2H), 2.39 (s, 3H).

^{13}C NMR (101 MHz, CDCl$_3$): δ_C 161.0, 151.0, 137.1, 128.8 (2xC), 127.0, 123.4, 32.2, 14.1.

IR (neat): ν_{max}**/cm**$^{-1}$ 3030, 1603, 1577, 1496, 1455, 1386, 1333, 1220, 1119, 1011, 966, 831, 723, 699, 669.

HRMS (NSI) [M+H]+ calcd. for $C_{11}H_{12}NO$ 174.0913, found: 174.0912.

5-(Benzo[d][1,3]dioxol-5-ylmethyl)-2-methyloxazole, 21b

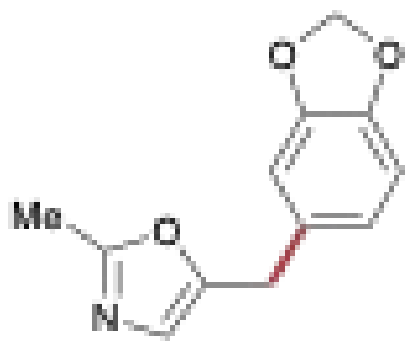

Prepared according to general procedure 1 for the synthesis of 2,5-disubstituted oxazoles (R = Me): using *N*-(prop-2-yn-1-yl)acetamide **17** (14.5 mg, 0.149 mmol), triphenylphosphine gold(I) trifluoromethanesulfonate (2.28 mg, 3.75 µmol, 2.5 mol %), 6-(trimethylsilyl)benzo[d][1,3]dioxol-5-yl trifluoromethanesulfonate **11b** (79.0 mg, 0.231 mmol) and cesium fluoride (107 mg, 0.704 mmol). The crude reaction mixture was purified by silica gel flash chromatography to afford the title compound (13.7 mg, 0.0631 mmol, 42%) as a colourless solid; R_f 0.18 (2:1 *n*-hexane:ethyl acetate).

^{1}**H NMR (400 MHz, CDCl$_3$):** δ_H 6.75 (d, *J* = 7.9, 1H), 6.70 (d, *J* = 1.2, 1H), 6.68 (dd, *J* = 7.9, 1.2, 1H), 6.61 (s, 1H) 5.93 (s, 2H), 3.84 (s, 2H), 2.39 (s, 3H).

^{13}**C NMR (101 MHz, CDCl$_3$):** δ_C 161.0, 151.1, 148.0, 146.6, 130.7, 123.3, 121.8, 109.2, 108.5, 101.1, 31.8, 14.1.

IR (neat): ν_{max}**/cm**$^{-1}$ 1577, 1503, 1489, 1444, 1246, 1220, 1187, 1119, 1038, 968, 939, 926, 811, 772, 757.

HRMS (NSI) [M+H]+ calcd. for $C_{12}H_{12}NO_3$ 218.0812, found: 218.0812.

5-(3,4-Difluorobenzyl)-2-methyloxazole, 21c

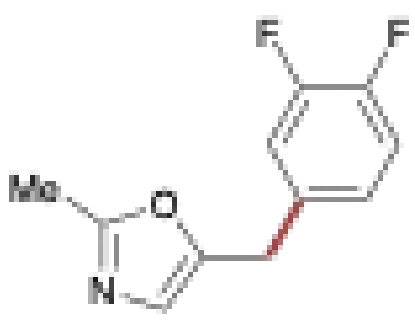

Prepared according to general procedure 1 for the synthesis of 2,5-disubstituted oxazoles (R = Me): using *N*-(prop-2-yn-1-yl)acetamide **17** (14.8 mg, 0.152 mmol), triphenylphosphine gold(I) trifluoromethanesulfonate (2.28 mg, 3.75 µmol, 2.5 mol %), 4,5-difluoro-2-(trimethylsilyl)phenyl trifluoromethanesulfonate **11c** (78.0 mg, 0.233 mmol) and cesium fluoride (105 mg, 0.691 mmol). The crude reaction mixture was purified by silica gel flash chromatography to afford the title compound (9.3 mg, 0.0445 mmol, 29%) as a pale yellow oil; R_f 0.16 (2:1 *n*-hexane:ethyl acetate).

^{1}H NMR (400 MHz, CDCl$_3$): δ_H 7.10 (m, 1H), 7.03 (m, 1H), 6.94 (m, 1H), 6.65 (s, 1H), 3.89 (s, 2H), 2.40 (s, 3H).

^{13}C NMR (101 MHz, CDCl$_3$): δ_C 171.2, 150.8 (dd, $J_{C\text{-}F}$ 142, 12.6), 149.6, 149.2 (dd, $J_{C\text{-}F}$ 141, 12.4), 134.0 (t, $J_{C\text{-}F}$ 4.5), 124.6 (dd, $J_{C\text{-}F}$ 6.3, 3.5), 123.7, 117.7 (d, $J_{C\text{-}F}$ 17.6), 117.5 (d, $J_{C\text{-}F}$ 17.3), 31.3, 14.1.

^{19}F NMR (376 MHz, CDCl$_3$): δ_F –137.6 (d, $J_{F\text{-}F}$ 21.2, 1F), –140.7 (d, $J_{F\text{-}F}$ 21.2, 1F).

IR (neat): ν_{max}/cm^{-1} 1736, 1610, 1578, 1518, 1438, 1388, 1282, 1212, 1116, 970, 821, 781, 762, 741, 670.

HRMS (NSI) [M+H]+ calcd. for $C_{11}H_{10}F_2NO$ 210.0725, found: 210.0726.

5-(3-Methoxybenzyl)-2-methyloxazole, 21f

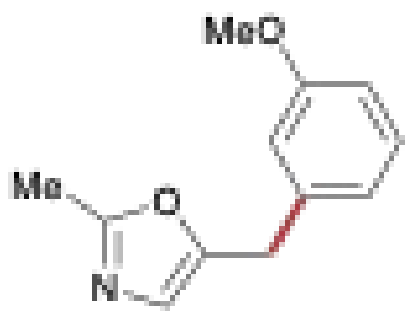

Prepared according to general procedure 1 for the synthesis of 2,5-disubstituted oxazoles (R = Me): using *N*-(prop-2-yn-1-yl)acetamide **17** (14.6 mg, 0.150 mmol), triphenylphosphine gold(I) trifluoromethanesulfonate (2.28 mg, 3.75 µmol, 2.5 mol %), 3-methoxy-2-(trimethylsilyl)phenyl trifluoromethanesulfonate **11f** (79.0 mg, 0.231 mmol) and cesium fluoride (107 mg, 0.704 mmol). The crude reaction mixture was purified by silica gel flash chromatography to afford the title compound (20.8 mg, 0.0958 mmol, 64%) as a pale yellow oil; R_f 0.17 (2:1 *n*-hexane:ethyl acetate).

^{1}H NMR (400 MHz, CDCl$_3$): δ_H 7.23 (t, J = 7.9, 1H), 6.83-6.76 (m, 3H), 6.63 (s, 1H), 3.91 (s, 2H), 3.79 (s, 3H), 2.39 (s, 3H).

^{13}C NMR (101 MHz, CDCl$_3$): δ_C 160.9, 159.9, 150.7, 138.5, 129.6, 123.3, 121.0, 114.5, 112.1, 55.2, 32.0, 14.0.

IR (neat): ν_{max}/cm^{-1} 1600, 1578, 1490, 1466, 1452, 1438, 1285, 1260, 1219, 1163, 1150, 1119, 1046, 757, 737.

HRMS (ESI) [M+H]+ calcd. for $C_{12}H_{14}NO_2$ 204.1019, found: 204.1038.

2-Methyl-5-(naphthalen-2-ylmethyl)oxazole, 21h & 2-methyl-5-(naphthalen-1-ylmethyl)oxazole, 21h'

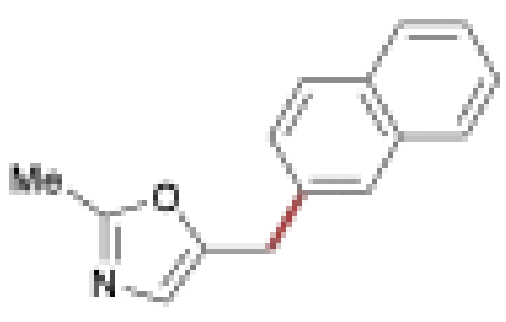

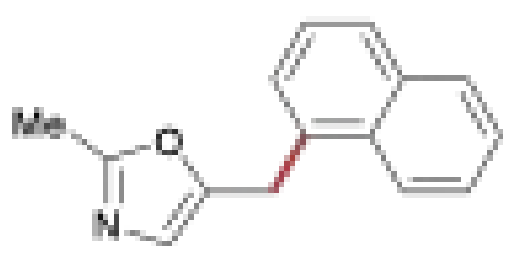

Prepared according to general procedure 1 for the synthesis of 2,5-disubstituted oxazoles (R = Me): using *N*-(prop-2-yn-1-yl)acetamide **17** (28.9 mg, 0.300 mmol), triphenylphosphine gold(I) trifluoromethanesulfonate (4.50 mg, 7.50 µmol, 2.5 mol %), 1-(trimethylsilyl)naphthalen-2-yl trifluoromethanesulfonate **11h** (114 mg, 0.328 mmol) and cesium fluoride (150 mg, 0.988 mmol). The crude reaction mixture was purified by silica gel flash chromatography to afford the title compound as an inseparable 1.8:1 mixture **21h:21h'** (37.8 mg, 0.169 mmol, 57%) as an amber solid; R_f 0.44 (2:1 *n*-hexane:ethyl acetate).

Data provided for a 1.8:1 regioisomeric mixture of **21h:21h'**:

^{1}H NMR (400 MHz, CDCl$_3$): δ_H 8.01-7.99 (s, 0.3H), 7.88-7.85 (s, 0.3H), 7.82-7.77 (m, 2.4H), 7.67 (s, 0.6H), 7.51-7.40 (m, 2.4H), 7.36-7.33 (m, 1H), 6.66 (s, 0.6H), 6.54 (s, 0.3H), 4.38 (s, 0.7H), 4.09 (s, 1.3H), 2.39 (s, 1.9H), 2.39 (s, 1.1H).

^{13}C NMR (101 MHz, CDCl$_3$): δ_C 161.0, 160.7, 150.8, 150.6, 134.4, 133.9, 133.5, 132.8, 132.4, 131.8, 128.8, 128.3, 127.8, 127.7, 127.6, 127.2, 127.0, 126.9, 126.2, 125.8, 125.7, 125.5, 123.6, 123.6, 123.4, 32.2 (2xC), 14.0 (2xC).

IR (neat): ν_{max}/cm^{-1} 1601, 1576, 1510, 1440, 1385, 1367, 1221, 1119, 1012, 967, 858, 819, 780, 760, 670.

HRMS (NSI) [M+H]+ calcd. for $C_{15}H_{14}NO$ 224.107, found: 224.107.

2-Methyl-5-(3-methylbenzyl)oxazole, 21j & 2-methyl-5-(4-methylbenzyl)oxazole, 21j'

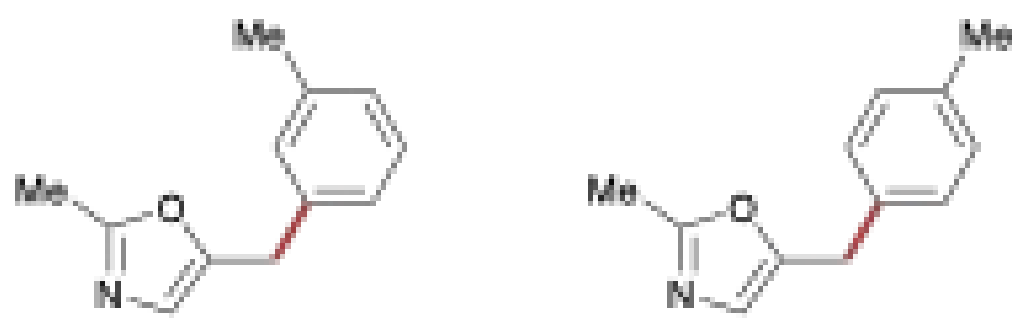

Prepared according to general procedure 1 for the synthesis of 2,5-disubstituted oxazoles (R = Me): using *N*-(prop-2-yn-1-yl)acetamide **17** (29.1 mg, 0.300 mmol), triphenylphosphine gold(I) trifluoromethanesulfonate (4.6 mg, 7.50 µmol, 2.5 mol %), 4-methyl-2-(trimethylsilyl)phenyl trifluoromethanesulfonate **11j** (103 mg, 0.330 mmol) and cesium fluoride (150 mg, 0.986 mmol). The crude reaction mixture was purified by silica gel flash chromatography to afford the title compounds as an inseparable 1:1 mixture (25.4 mg, 0.136 mmol, 45%) as a yellow oil; R_f 0.24 (2:1 *n*-hexane:ethyl acetate).

Data provided for a 1:1 regioisomeric mixture of **21j:21j'**:

^{1}H NMR (400 MHz, CDCl$_3$): δ_H 7.20 (m, 0.5H), 7.12 (s, 2H), 7.07-7.02 (m, 1.5H), 6.61 (s, 0.5H), 6.60 (s, 0.5H), 3.89 (s, 2H), 2.38 (s, 1.5H), 2.33 (s, 1.5H).

^{13}C NMR (101 MHz, CDCl$_3$): δ_C 161.0, 160.9, 151.3, 151.2, 138.4, 137.0, 136.6, 134.0, 129.5 (2xC), 129.4, 128.7 (2xC), 128.7, 127.7, 125.8, 123.3, 123.2, 32.0, 31.7, 21.5, 21.2, 14.1 (2xC).

IR (neat): ν_{max}/cm^{-1} 1607, 1577, 1515, 1492, 1439, 1386, 1220, 1119, 1012, 966, 822, 796, 757, 749, 670.

HRMS (ASAP) [M+H]+ calcd. for $C_{12}H_{14}NO$ 188.1075, found: 188.1072.

2,5-Dibenzyloxazole, 22a

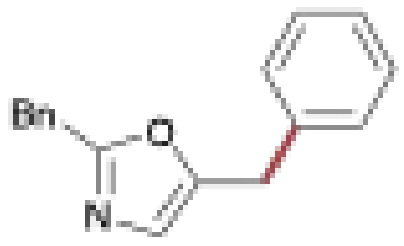

Prepared according to general procedure 1 for the synthesis of 2,5-disubstituted oxazoles (R = Bn): using 2-phenyl-*N*-(prop-2-yn-1-yl)acetamide **18** (50.8 mg, 0.293 mmol), triphenylphosphine gold(I) trifluoromethanesulfonate (7.13 mg, 11.7 µmol, 4.0 mol %), 2-(trimethylsilyl)phenyl trifluoromethanesulfonate **11a** (96.2 mg, 0.323 mmol) and cesium fluoride (123 mg, 0.806 mmol). The crude reaction mixture was purified by silica gel flash chromatography to afford the title compound (57.6 mg, 0.231 mmol, 79%) as a yellow oil; R_f 0.39 (2:1 *n*-hexane:ethyl acetate).

^{1}H NMR (400 MHz, CDCl$_3$): δ_H 7.31-7.20 (m, 10H), 6.63 (s, 1H), 4.05 (s, 2H), 3.93 (s, 2H).

^{13}C NMR (101 MHz, CDCl$_3$): δ_C 161.0, 151.0, 137.1, 128.8 (4xC), 127.0, 123.4, 32.2, 14.1.

IR (neat): ν_{max}/cm^{-1} 1721, 1601, 1564, 1496, 1455, 1243, 1219, 1172, 1106, 1030, 992, 755, 727, 711, 696.

HRMS (NSI) [M+H]+ calcd. for $C_{17}H_{16}NO$ 250.1226, found: 250.1229.

5-(Benzo[d][1,3]dioxol-5-ylmethyl)-2-benzyloxazole, 22b

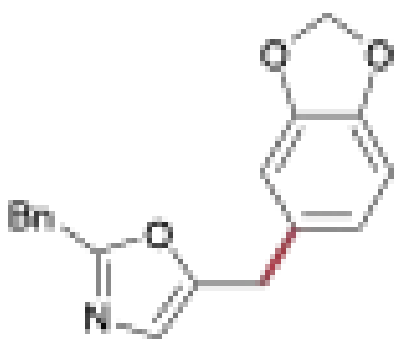

Prepared according to general procedure 1 for the synthesis of 2,5-disubstituted oxazoles (R = Bn): using 2-phenyl-*N*-(prop-2-yn-1-yl)acetamide **18** (50.7 mg, 0.293 mmol), triphenylphosphine gold(I) trifluoromethanesulfonate (7.13 mg, 11.7 µmol, 4.0 mol %), 6-(trimethylsilyl)benzo[d][1,3]dioxol-5-yl trifluoromethanesulfonate **11b** (110 mg, 0.322 mmol) and cesium fluoride (146 mg, 0.966 mmol). The crude reaction mixture was purified by silica gel flash chromatography to afford the title compound (47.7 mg, 0.163 mmol, 56%) as a yellow oil; R_f 0.29 (2:1 *n*-hexane:ethyl acetate).

^{1}H NMR (400 MHz, CDCl$_3$): δ_H 7.33-7.23 (m, 5H), 6.73 (d, *J* = 7.9, 1H), 6.69 (d, *J* = 1.2, 1H), 6.66 (dd, *J* = 7.9, 1.2, 1H), 6.62 (s, 1H), 5.92 (s, 2H), 4.05 (s, 2H), 3.83 (s, 2H).

^{13}C NMR (101 MHz, CDCl$_3$): δ_C 162.6, 151.7, 148.0, 146.6, 135.8, 130.5, 128.9, 128.8, 127.1, 123.5, 121.8, 109.3, 108.5, 101.1, 34.8, 31.8.

IR (neat): ν_{max}**/cm^{-1}** 1566, 1502, 1489, 1455, 1444, 1247, 1216, 1186, 1101, 1038, 992, 927, 811, 726, 696.

HRMS (NSI) [M+H]+ calcd. for $C_{18}H_{16}NO_3$ 294.1125, found: 294.1126.

2-Benzyl-5-(3,4-diuorobenzyl)oxazole, 22c

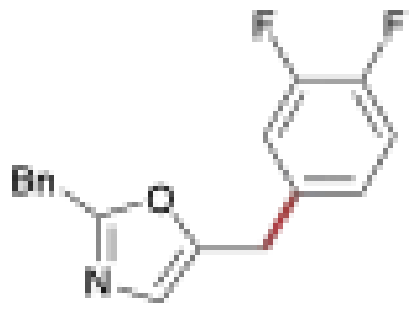

Prepared according to general procedure 1 for the synthesis of 2,5-disubstituted oxazoles (R = Bn): using 2-phenyl-*N*-(prop-2-yn-1-yl)acetamide **18** (45.0 mg, 0.260 mmol), triphenylphosphine gold(I) trifluoromethanesulfonate (6.33 mg, 10.4 µmol, 4.0 mol %), 4,5-difluoro-2-(trimethylsilyl)phenyl trifluoromethanesulfonate **11c** (95.5 mg, 0.286 mmol) and cesium fluoride (130 mg, 0.858 mmol). The crude reaction mixture was purified by silica gel flash chromatography to afford the title compound (28.1 mg, 0.0985 mmol, 38%) as a pale yellow oil; R_f 0.28 (2:1 *n*-hexane:ethyl acetate).

^{1}H NMR (400 MHz, CDCl$_3$): 7.34-7.25 (m, 5H), 7.08 (m, 1H), 7.01 (s, 1H), 6.91 (m, 1H), 6.67 (s, 1H), 4.06 (s, 2H), 3.89 (s, 2H).

^{13}C NMR (101 MHz, CDCl$_3$): δ_C 161.4, 150.8 (dd, $J_{C\text{-}F}$ 137, 12.7), 150.0, 149.2 (dd, $J_{C\text{-}F}$ 136, 13.0), 135.7, 134.8 (t, $J_{C\text{-}F}$ 4.6), 128.9, 128.9, 127.2, 124.7 (dd, $J_{C\text{-}F}$ 6.1, 3.4, 1C), 123.9, 117.7 (d, $J_{C\text{-}F}$ 17.8), 117.5 (d, $J_{C\text{-}F}$ 17.3), 34.9, 31.3.

^{19}F NMR (376 MHz, CDCl$_3$): δ_F –137.5 (d, $J_{F\text{-}F}$ 21.2, 1F), –140.5 (d, $J_{F\text{-}F}$ 21.2, 1F).

IR (neat): ν_{max}**/cm^{-1}** 1565, 1517, 1496, 1437, 1281, 1210, 1114, 1105, 993, 820, 780, 753, 734, 725, 695.

HRMS (NSI) [M+H]+ calcd. for $C_{17}H_{14}F_2NO$ 286.1038, found: 286.1039.

2-Benzyl-5-(3-methoxybenzyl)oxazole, 22f

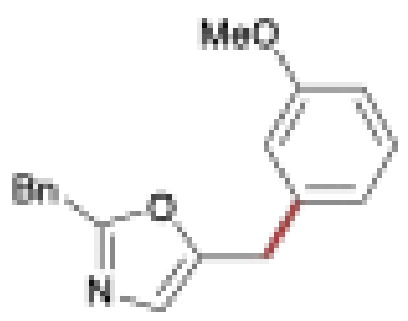

Prepared according to general procedure 1 for the synthesis of 2,5-disubstituted oxazoles (R = Bn): using 2-phenyl-*N*-(prop-2-yn-1-yl)acetamide **18** (27.7 mg, 0.160 mmol), triphenylphosphine gold(I) trifluoromethanesulfonate (4.0 mg, 6.40 µmol, 4.0 mol %), 3-methoxy-2-(trimethylsilyl)phenyl trifluoromethanesulfonate **11f** (75.0 mg, 0.228 mmol) and cesium fluoride (106 mg, 0.698 mmol). The

crude reaction mixture was purified by silica gel flash chromatography to afford the title compound (35.3 mg, 0.120 mmol, 75%) as a yellow oil; R_f 0.29 (2:1 *n*-hexane:ethyl acetate).

^{1}H NMR (400 MHz, CDCl$_3$): δ_H 7.32-7.20 (m, 6H), 6.81-6.77 (m, 2H), 6.75-6.73 (m, 1H), 6.65 (s, 1H), 4.05 (s, 2H), 3.90 (s, 2H), 3.76 (s, 3H).

^{13}C NMR (101 MHz, CDCl$_3$): δ_C 162.5, 159.9, 151.3, 138.3, 135.7, 129.6, 128.8, 128.7, 127.0, 123.6, 121.0, 114.3, 112.3, 55.2, 34.7, 32.0.

IR (neat): ν_{max}/cm^{-1} 1600, 1585, 1566, 1492, 1455, 1436, 1260, 1163, 1150, 1103, 1046, 992, 751, 732, 695.

HRMS (NSI) [M+H]+ calcd. for $C_{18}H_{18}NO_2$ 280.1334, found: 280.1332.

2-Benzyl-5-(naphthalen-2-ylmethyl)oxazole, 22h & 2-benzyl-5-(naphthalen-1-ylmethyl)oxazole, 22h'

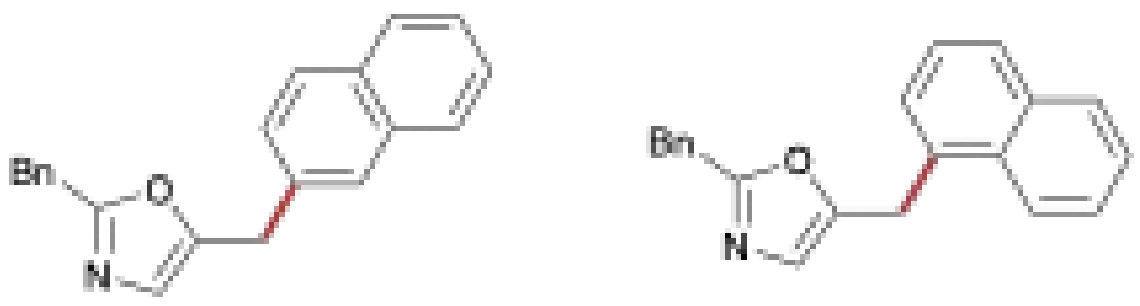

Prepared according to general procedure 1 for the synthesis of 2,5-disubstituted oxazoles (R = Bn): using 2-phenyl-*N*-(prop-2-yn-1-yl)acetamide **18** (44.6 mg, 0.257 mmol), triphenylphosphine gold(I) trifluoromethanesulfonate (5.4 mg, 8.88 μmol, 4.0 mol %), 1-(trimethylsilyl)naphthalen-2-yl trifluoromethanesulfonate **11h** (126 mg, 0.362 mmol) and cesium fluoride (130 mg, 0.850 mmol). The crude reaction mixture was purified by silica gel flash chromatography to afford the title compound as an inseparable 1.7:1 mixture **22h:22h'** (34.1 mg, 0.114 mmol, 44%) as an amber solid; R_f 0.33 (2:1 *n*-hexane:ethyl acetate).

Data provided for a 1.7:1 regioisomeric mixture of **22h:22h'**:

^{1}H NMR (400 MHz, CDCl$_3$): δ_H 7.99-7.95 (m, 0.4H), 7.88-7.84 (m, 0.4H), 8.82-7.74 (m, 2.6H), 7.63 (s, 0.6H), 7.50-7.42 (m, 1.6H), 7.40 (dd, *J* = 8.1, 7.1, 0.4H), 7.34-7.22 (m, 6H), 6.67 (s, 0.6H), 6.55 (s, 0.4H), 4.37 (s, 0.8H), 4.08 (s, 1.2H), 4.05(s, 1.2H), 4.04 (s, 0.8H).

^{13}C NMR (101 MHz, CDCl$_3$): δ_C 162.5, 162.2, 151.3, 151.1, 135.7, 134.2, 133.9, 133.5, 132.7, 132.4, 131.7, 128.8, 128.8, 128.8, 128.7, 128.7, 128.3, 127.8, 127.7, 127.6, 127.2, 127.0, 126.9, 126.2, 126.2, 125.8, 125.7, 125.5, 123.9, 123.7, 123.6, 34.7 (2xC), 32.2, 29.5.

IR (neat): ν_{max}/cm^{-1} 1565, 1496, 1166, 1102, 1074, 1030, 992, 963, 897, 817, 792, 781, 757, 728, 695.

HRMS (NSI) [M+H]+ calcd. for $C_{21}H_{17}NO$ 299.1305, found: 299.1301.

2-Benzyl-5-(3-methylbenzyl)oxazole, 22j & 2-benzyl-5-(4-methylbenzyl)oxazole, 22j'

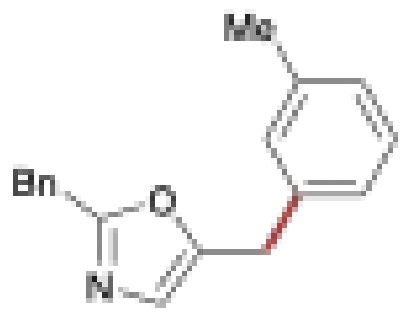

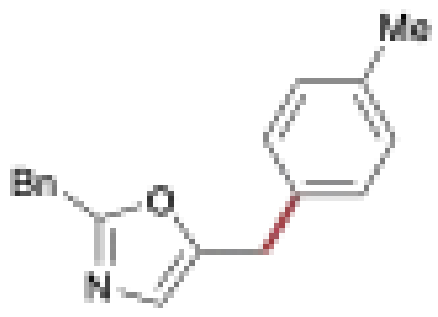

Prepared according to general procedure 1 for the synthesis of 2,5-disubstituted oxazoles (R = Bn): using 2-phenyl-*N*-(prop-2-yn-1-yl)acetamide **18** (26.0 mg, 0.150 mmol), triphenylphosphine gold(I) trifluoromethanesulfonate (3.65 mg, 6.00 µmol, 4.0 mol %), 4-methyl-2-(trimethylsilyl)phenyl trifluoromethanesulfonate **11j** (56.2 mg, 0.180 mmol) and cesium fluoride (62.7 mg, 0.413 mmol). The crude reaction mixture was purified by silica gel flash chromatography to afford the title compounds as an inseparable 1:1 mixture (18.7 mg, 0.071 mmol, 47%) as a yellow oil; R_f 0.40 (2:1 *n*-hexane:ethyl acetate).

Data provided for a 1:1 regioisomeric mixture of **22j:22j'**:

^{1}H NMR (400 MHz, CDCl$_3$): δ_H 7.33-7.23 (m, 5H), 7.21-7.17 (m, 0.5H), 7.12-7.08 (m, 2.5H), 7.02-6.99 (m, 1H), 6.66 (s, 0.5H), 6.64 (s, 0.5H), 4.07 (s, 1H), 4.07 (s, 1H), 3.89 (s, 1H), 3.89 (s, 1H), 2.33 (s, 1.5H), 2.32 (s, 1.5H).

^{13}C NMR (101 MHz, CDCl$_3$): 162.6, 152.0, 151.8, 138.3, 136.5, 136.4, 135.4, 133.4, 129.4, 129.4, 128.8, 128.7, 128.6, 127.6, 127.1, 125.7, 123.0, 122.9, 34.6, 34.6, 31.9, 31.6, 21.3, 21.0.

IR (neat): ν_{max}/cm^{-1} 1564, 1495, 1455, 1424, 1102, 991, 961, 822, 792, 744, 724, 694, 670, 638, 624.

HRMS (NSI) [M+H]+ calcd. for $C_{18}H_{18}NO$ 264.1383, found: 264.1385.

5-Benzyl-2-phenyloxazole, 23a

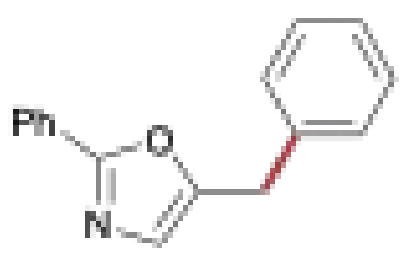

Prepared according to general procedure 2 for the synthesis of 2,5-disubstituted oxazoles (R = Ph): using *N*-(prop-2-yn-1-yl)benzamide **19** (25.5 mg, 0.160 mmol), triphenylphosphine gold(I) bis[(trifluoromethyl)sulfonyl]amide (1.95 mg, 3.20 µmol, 2.0 mol %), 2-(trimethylsilyl)phenyl trifluoromethanesulfonate **11a** (52.6 mg, 0.176 mmol) and cesium fluoride (66.9 mg, 0.441 mmol). The crude reaction mixture was purified by silica gel flash chromatography to afford the title compound (23.3 mg, 0.0990 mmol, 62%) as a yellow oil; R_f 0.35 (2:1 *n*-hexane:ethyl acetate).

^{1}H NMR (400 MHz, CDCl$_3$): δ_H 7.84 (m, 2H), 7.28-7.10 (m, 8H), 6.70 (s, 1H), 3.91 (s, 2H).

^{13}C NMR (101 MHz, CDCl$_3$): δ_C 161.4, 151.5, 136.8, 130.2, 128.8 (2xC), 127.8, 127.1, 126.2, 125.0, 32.3.

IR (neat): ν_{max}/cm^{-1} 3030, 2921, 1727, 1684, 1495, 1450, 1250, 1064, 985, 709, 691.

HRMS (NSI) [M+H]+ calcd. for $C_{16}H_{14}NO$ 236.1070, found: 236.1071.

5-(Benzo[d][1,3]dioxol-5-ylmethyl)-2-phenyloxazole, 23b

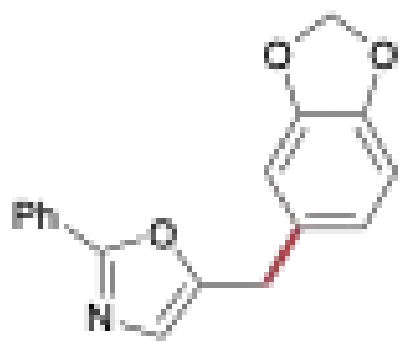

Prepared according to general procedure 2 for the synthesis of 2,5-disubstituted oxazoles (R = Ph): using *N*-(prop-2-yn-1-yl)benzamide **19** (25.5 mg, 0.160 mmol), triphenylphosphine gold(I) bis[(trifluoromethyl)sulfonyl]amide (1.95 mg, 3.20 µmol, 2.0 mol %), 6-(trimethylsilyl)benzo[d][1,3]dioxol-5-yl trifluoromethanesulfonate **11b** (61.6 mg, 0.180 mmol) and cesium fluoride (60.8 mg, 0.400 mmol). The crude reaction mixture was purified by silica gel flash chromatography to afford the title compound (27.3 mg, 0.0977 mmol, 61%) as a yellow oil; R_f 0.27 (2:1 *n*-hexane:ethyl acetate).

^{1}H NMR (400 MHz, CDCl$_3$): δ_H 7.99 (dd, J = 7.4, 2.2, 2H), 7.45-7.41 (m, 3H), 6.86 (s, 1H), 6.79-6.75 (m, 3H), 5.95 (s, 2H), 3.98 (s, 2H).

^{13}C NMR (101 MHz, CDCl$_3$): δ_C 161.4, 151.6, 148.0, 146.7, 130.5, 130.2, 128.8, 127.8, 126.2, 124.9, 121.8, 109.3, 108.5, 101.2, 32.0.

IR (neat): ν_{max}/cm^{-1} 2896, 1727, 1683, 1550, 1501, 1488, 1443, 1243, 1120, 1098, 986, 711.

HRMS (NSI) [M+H]+ calcd. for $C_{17}H_{14}NO_3$ 280.0968, found: 280.0969.

5-(3,4-Diuorobenzyl)-2-phenyloxazole, 23c

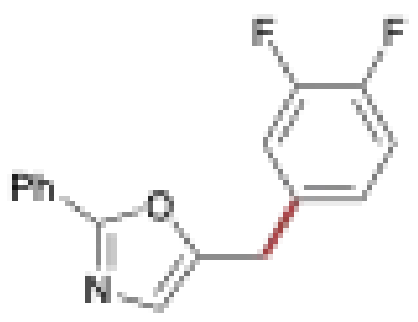

Prepared according to general procedure 2 for the synthesis of 2,5-disubstituted oxazoles (R = Ph): using *N*-(prop-2-yn-1-yl)benzamide **19** (25.5 mg, 0.160 mmol), triphenylphosphine gold(I) bis[(trifluoromethyl)sulfonyl]amide (1.95 mg, 3.20 µmol, 2.0 mol %), 4,5-difluoro-2-(trimethylsilyl)phenyl trifluoromethanesulfonate **11c** (60.2 mg, 0.180 mmol) and cesium fluoride (60.8 mg, 0.400 mmol). The crude reaction mixture was purified by silica gel flash chromatography to afford the title compound (23.9 mg, 0.0881 mmol, 55%) as a yellow oil; R_f 0.30 (2:1 *n*-hexane:ethyl acetate).

^{1}H NMR (400 MHz, CDCl$_3$): 8.00-7.97 (m, 2H), 7.47-7.41 (m, 3H), 7.16-7.07 (m, 2H), 7.02-6.98 (m, 1H), 6.89 (s, 1H), 4.03 (s, 2H).

^{13}C NMR (101 MHz, CDCl$_3$): δ_C 161.7, 150.5 (dd, J_{C-F} 249, 12.9), 150.4, 149.6 (dd, J_{C-F} 247, 12.6), 133.7 (dd, J_{C-F} 5.5, 4.0), 130.4, 128.9, 127.6, 126.3, 125.3, 124.7 (dd, J_{C-F} 6.2, 3.6), 117.8 (d, J_{C-F} 15.2), 117.6 (d, J_{C-F} 14.9), 31.4.

^{19}F NMR (376 MHz, CDCl$_3$): δ_F –137.3 (d, J_{F-F} 21.2, 1F), –140.3 (d, J_{F-F} 21.2, 1F).

IR (neat): ν_{max}/cm^{-1} 3065, 2919, 1728, 1610, 1517, 1450, 1282, 1210, 1117, 988, 712.

HRMS (NSI) [M+H]+ calcd. for $C_{16}H_{12}F_2NO$ 272.0881, found: 272.0883.

5-(3-Methoxybenzyl)-2-phenyloxazole, 23f

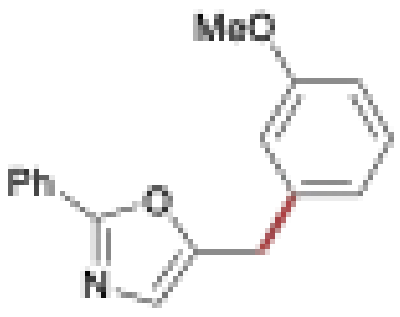

Prepared according to general procedure 2 for the synthesis of 2,5-disubstituted oxazoles (R = Ph): using *N*-(prop-2-yn-1-yl)benzamide **19** (25.5 mg, 0.160 mmol), triphenylphosphine gold(I) bis[(trifluoromethyl)sulfonyl]amide (1.95 mg, 3.20 µmol, 2.0 mol %), 3-methoxy-2-(trimethylsilyl)phenyl trifluoromethanesulfonate **11f** (59.1 mg, 0.180 mmol) and cesium fluoride (60.8 mg, 0.400 mmol). The crude reaction mixture was purified by silica gel flash chromatography to afford the title compound (19.9 mg, 0.0750 mmol, 47%) as a yellow oil; R_f 0.27 (2:1 *n*-hexane:ethyl acetate).

^{1}H NMR (400 MHz, CDCl$_3$): δ_H 7.87 (m, 2H), 7.31-7.28 (m, 3H), 7.13 (t, J = 7.8, 1H), 6.76-6.67 (m, 4H), 3.92 (s, 2H), 3.67 (s, 3H).

^{13}C NMR (101 MHz, CDCl$_3$): δ_C 161.4, 160.0, 151.3, 138.3, 130.2, 129.8, 128.8, 127.8, 126.2, 125.0, 121.2, 114.6, 112.4, 55.3, 32.3.

IR (neat): ν_{max}/cm^{-1} 3024, 2919, 1706, 1696, 1515, 1483, 1449, 1120, 1064, 986, 711.

HRMS (NSI) [M+H]+ calcd. for $C_{17}H_{16}NO_2$ 266.1176, found: 266.1177.

5-(Naphthalen-2-ylmethyl)-2-phenyloxazole, 23h & 5-(Naphthalen-1-ylmethyl)-2-phenyloxazole, 23h'

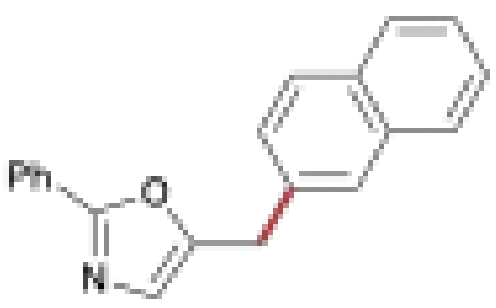

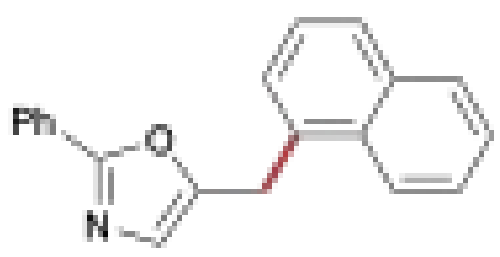

Prepared according to general procedure 2 for the synthesis of 2,5-disubstituted oxazoles (R = Ph): using *N*-(prop-2-yn-1-yl)benzamide **19** (25.5 mg, 0.160 mmol), triphenylphosphine gold(I) bis[(trifluoromethyl)sulfonyl]amide (1.95 mg, 3.20 µmol, 2.0 mol %), 1-(trimethylsilyl)naphthalen-2-yl trifluoromethanesulfonate **11h** (112 mg, 0.320 mmol) and cesium fluoride (122 mg, 0.800 mmol). The crude reaction mixture was purified by silica gel flash chromatography to afford the title compound as an inseparable 1.6:1 mixture **23h:23h'** (34.7 mg, 0.122 mmol, 76%) as a yellow solid; R_f 0.33 (2:1 *n*-hexane:ethyl acetate).

Data provided for a 1.6:1 regioisomeric mixture of **23h:23h'**:

^{1}H NMR (400 MHz, $CDCl_3$): δ_H 8.08 (m, 0.4H), 8.04-7.97 (m, 2H), 7.90 (m, 0.4H), 7.86-7.79 (m, 2.2H), 7.74 (s, 0.6H), 7.57-7.42 (m, 6.4H), 6.92 (s, 0.6H), 6.80 (s, 0.4H), 4.52 (s, 0.8H), 4.24 (s, 1.2H)

^{13}C NMR (101 MHz, $CDCl_3$): δ_C 161.5, 161.2, 151.4, 151.2, 134.2, 134.0, 133.7, 132.7, 132.6, 131.9, 130.2, 130.1, 128.9, 128.8, 128.5, 128.1, 127.8, 127.8, 127.7, 127.3, 127.2, 127.0, 126.4, 126.4, 126.2, 126.2, 126.0, 125.9, 125.7, 125.4, 125.2, 123.7, 32.5, 29.8

IR (neat): ν_{max}/cm^{-1} 3051, 1727, 1685, 1596, 1549, 1510, 1483, 1365, 1120, 987, 779, 710, 690.

HRMS (NSI) [M+H]+ calcd. for $C_{20}H_{16}NO$ 286.1225, found: 286.1226.

5-(3-Methylbenzyl)-2-phenyloxazole, 23j & 5-(4-methylbenzyl)-2-phenyloxazole, 23j'

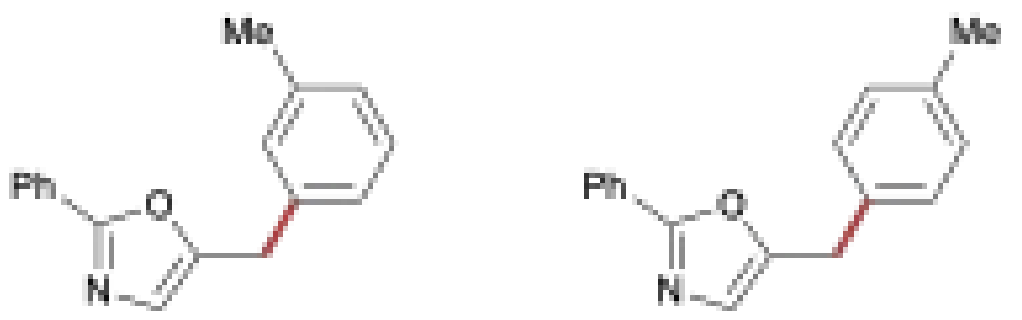

Prepared according to general procedure 2 for the synthesis of 2,5-disubstituted oxazoles (R = Ph): using *N*-(prop-2-yn-1-yl)benzamide **19** (25.5 mg, 0.160 mmol), triphenylphosphine gold(I) bis[(trifluoromethyl)sulfonyl]amide (1.95 mg, 3.20 µmol, 2.0 mol %), 4-methyl-2-(trimethylsilyl)phenyl trifluoromethanesulfonate **11j** (56.2 mg, 0.180 mmol) and cesium fluoride (62.7 mg, 0.413 mmol). The crude reaction mixture was purified by silica gel flash chromatography to afford the title compounds as an inseparable 1:1 mixture (22.3 mg, 0.0895 mmol, 56%) as a yellow oil; R_f 0.41 (2:1 *n*-hexane:ethyl acetate).

Data provided for a 1:1 regioisomeric mixture of **23j:23j'**:

^{1}H NMR (400 MHz, $CDCl_3$): δ_H 8.04-7.95 (m, 2H), 7.48-7.38 (m, 3H), 7.25-7.08 (m, 4H), 6.87 (s, 0.5H), 6.85 (s, 0.5H), 4.03 (s, 2H), 2.35 (s, 3H).

^{13}C NMR (101 MHz, $CDCl_3$): 161.4, 161.3, 151.8, 151.7, 138.5, 136.7, 136.7, 133.7, 130.1, 130.1, 129.6, 129.5, 128.8, 128.7, 127.9, 127.8, 126.2, 125.8, 125.0, 124.9, 32.2, 31.9, 21.5, 21.2.

IR (neat): ν_{max}/cm^{-1} 3024, 2919, 1706, 1696, 1515, 1483, 1449, 1120, 1064, 986, 711.

HRMS (NSI) [M+H]+ calcd. for $C_{17}H_{16}NO$ 250.1226, found: 250.1228.

5-Benzyl-2-(thiophen-2-yl)oxazole, 24a

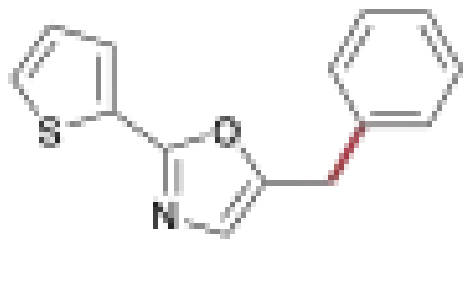

Prepared according to general procedure 2 for the synthesis of 2,5-disubstituted oxazoles (R = 2-thienyl): using *N*-propargylthiophene-2-carboxamide **20** (26.4 mg, 0.160 mmol), triphenylphosphine gold(I) bis[(trifluoromethyl)sulfonyl]amide (1.95 mg, 3.20 µmol, 2.0 mol %), 2-(trimethylsilyl)phenyl trifluoromethanesulfonate **11a** (52.6 mg, 0.176 mmol) and cesium fluoride (66.9 mg, 0.441 mmol). The crude reaction mixture was purified by silica gel flash chromatography to afford the title compound (20.1 mg, 0.0833 mmol, 52%) as a yellow oil; R_f 0.30 (2:1 *n*-hexane:ethyl acetate).

^{1}H NMR (400 MHz, CDCl$_3$): δ_H 7.57 (dd, *J* = 3.7, 1.0, 1H), 7.35-7.22 (m, 6H), 7.05 (dd, *J* = 5.0, 3.7, 1H), 6.77 (s, 1H), 4.01 (s, 2H).

^{13}C NMR (101 MHz, CDCl$_3$): δ_C 157.6, 151.1, 136.6, 130.4, 128.8, 128.8, 127.9 (2xC), 127.3, 127.1, 124.9, 32.2.

IR (neat): ν_{max}/cm^{-1} 3107, 3029, 1723, 1597, 1495, 1424, 1113, 981, 852, 707.

HRMS (NSI) [M+H]+ calcd. for $C_{14}H_{12}N_2OS$ 242.0634, found: 242.0635.

5-(Benzo[d][1,3]dioxol-5-ylmethyl)-2-(thiophen-2-yl)oxazole, 24b

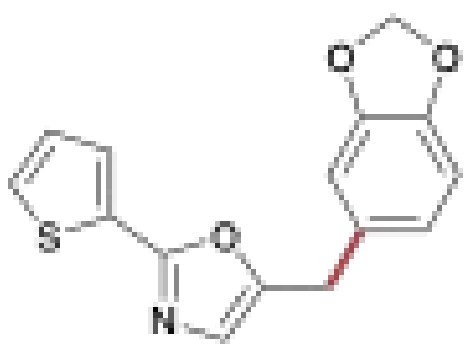

Prepared according to general procedure 2 for the synthesis of 2,5-disubstituted oxazoles (R = 2-thienyl): using *N*-propargylthiophene-2-carboxamide **20** (26.4 mg, 0.160 mmol), triphenylphosphine gold(I) bis[(trifluoromethyl)sulfonyl]amide (1.95 mg, 3.20 µmol, 2.0 mol %), 6-(trimethylsilyl)benzo[d][1,3]dioxol-5-yl trifluoromethanesulfonate **11b** (61.6 mg, 0.180 mmol) and cesium fluoride (60.8 mg, 0.400 mmol). The crude reaction mixture was purified by silica gel flash chromatography to afford the title compound (26.9 mg, 0.0943 mmol, 59%) as a yellow oil; R_f 0.25 (2:1 *n*-hexane:ethyl acetate).

^{1}H NMR (400 MHz, CDCl$_3$): δ_H 7.60 (dd, *J* = 3.7, 1.0,1H), 7.38 (dd, *J* = 5.0, 1.0, 1H), 7.08 (dd, *J* = 5.0, 3.7, 1H), 6.79-6.72 (m, 4H), 5.94 (s, 2H), 3.94 (s, 2H).

^{13}C NMR (101 MHz, CDCl$_3$): δ_C 157.6, 151.2, 148.0, 146.7, 130.4, 130.3, 128.0, 127.9, 127.4, 124.7, 121.8, 109.3, 108.5, 101.2, 31.9.

IR (neat): ν_{max}/cm^{-1} 3109, 2897, 1721, 1599, 1488, 1443, 1244, 1185, 1114, 1037, 926, 851, 723.

HRMS (NSI) [M+H]+ calcd. for $C_{15}H_{12}NO_3S$ 286.0532, found: 286.0534.

5-(3,4-Diuorobenzyl)-2-(thiophen-2-yl)oxazole, 24c

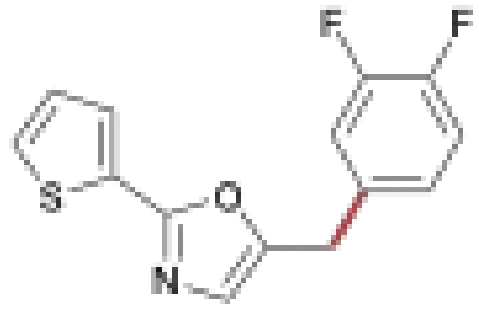

Prepared according to general procedure 2 for the synthesis of 2,5-disubstituted oxazoles (R = 2-thienyl): using *N*-propargylthiophene-2-carboxamide **20** (26.4 mg, 0.160 mmol), triphenylphosphine gold(I) bis[(trifluoromethyl)sulfonyl]amide (1.95 mg, 3.20 µmol, 2.0 mol %), 4,5-difluoro-2-(trimethylsilyl)phenyl trifluoromethanesulfonate **11c** (60.2 mg, 0.180 mmol) and cesium fluoride (60.8 mg, 0.400 mmol). The crude reaction mixture was purified by silica gel flash chromatography to afford the title compound (20.4 mg, 0.0736 mmol, 46%) as a yellow oil; R_f 0.35 (2:1 *n*-hexane:ethyl acetate).

^{1}H NMR (400 MHz, $CDCl_3$): 7.60 (dd, *J* = 3.7 1.0, 1H), 7.40 (d, *J* = 5.0 1.0 Hz, 1H), 7.15-7.06 (m, 3H), 7.01-6.97 (m, 1H), 6.83 (s, 1H), 4.02 (s, 2H).

^{13}C NMR (101 MHz, $CDCl_3$): δ_C 157.9, 150.5 (dd, J_{C-F} 249, 12.8), 150.0, 149.6 (dd, J_{C-F} 248, 12.6), 133.6 (dd, J_{C-F} 5.5, 4.0), 130.2, 128.2, 128.0, 127.6, 125.1, 124.7 (dd, J_{C-F} 6.2, 3.6), 117.8 (d, J_{C-F} 14.1), 117.6 (d, J_{C-F} 13.8), 31.3.

^{19}F NMR (376 MHz, $CDCl_3$): δ_F –137.3 (d, J_{F-F} 21.2, 1F), –140.3 (d, J_{F-F} 21.2, 1F).

IR (neat): ν_{max}/cm^{-1} 3111, 1722, 1609, 1518, 1424, 1281, 1115, 983, 723.

HRMS (NSI) [M+H]+ calcd. for $C_{14}H_{10}F_2NOS$ 278.0446, found: 278.0445.

5-(3-Methoxybenzyl)-2-(thiophen-2-yl)oxazole, 24f

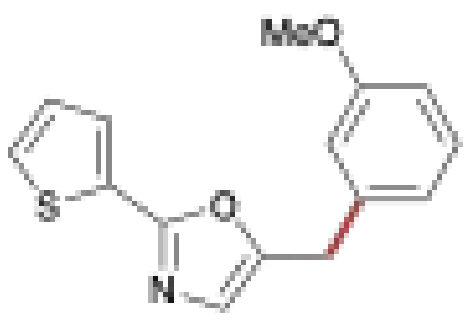

Prepared according to general procedure 2 for the synthesis of 2,5-disubstituted oxazoles (R = 2-thienyl): using *N*-propargylthiophene-2-carboxamide **20** (26.4 mg, 0.160 mmol), triphenylphosphine gold(I) bis[(trifluoromethyl)sulfonyl]amide (1.95 mg, 3.20 µmol, 2.0 mol %), 3-methoxy-2-(trimethylsilyl)phenyl trifluoromethanesulfonate **11f** (59.1 mg, 0.180 mmol) and cesium fluoride (60.8 mg, 0.400 mmol). The crude reaction mixture was purified by silica gel flash chromatography to afford the title compound (15.2 mg, 0.0561 mmol, 35%) as a yellow oil; R_f 0.30 (2:1 *n*-hexane:ethyl acetate).

^{1}H NMR (400 MHz, $CDCl_3$): δ_H 7.60 (dd, *J* = 3.7, 1.0, 1H), 7.38 (dd, *J* = 5.0, 1.0, 1H), 7.27-7.23 (m, 1H), 7.08 (dd, *J* = 5.0, 3.7, 1H), 6.87 (d, *J* = 7.7, 1H), 6.82-6.80 (m, 3H), 4.02 (s, 2H), 3.8 (s, 3H).

^{13}C NMR (101 MHz, $CDCl_3$): δ_C 160.0, 157.6, 150.9, 138.2, 130.4, 129.8, 128.0 (2xC), 127.4, 124.9, 121.2, 114.6, 112.4, 55.4, 32.2.

IR (neat): ν_{max}/cm^{-1} 3106, 2936, 2834, 1722, 1597, 1490, 1424, 1259, 1113, 1048, 981, 851, 722.

HRMS (NSI) [M+H]+ calcd. for $C_{15}H_{14}NO_2S$ 272.0740, found: 272.0742.

5-(Naphthalen-2-ylmethyl)-2-(thiophen-2-yl)oxazole, 24h & 5-(naphthalen-1-ylmethyl)-2-(thiophen-2-yl)oxazole, 24h’

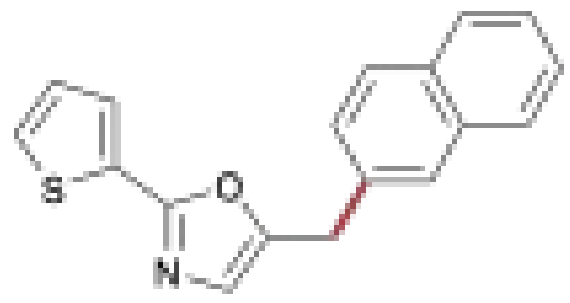
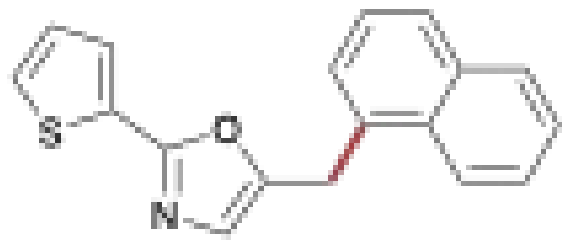

Prepared according to general procedure 2 for the synthesis of 2,5-disubstituted oxazoles (R = 2-thienyl): using *N*-propargylthiophene-2-carboxamide **20** (26.4 mg, 0.160 mmol), triphenylphosphine gold(I) bis[(trifluoromethyl)sulfonyl]amide (1.95 mg, 3.20 µmol, 2.0 mol %), 1-(trimethylsilyl)naphthalen-2-yl trifluoromethanesulfonate **11h** (112 mg, 0.320 mmol) and cesium fluoride (122 mg, 0.800 mmol). The crude reaction mixture was purified by silica gel flash chromatography to afford the title compound as an inseparable 1.6:1 mixture **24h:24h’** (28.9 mg, 0.0992 mmol, 62%) as a yellow solid; R_f 0.36 (2:1 *n*-hexane:ethyl acetate).

Data provided for a 1.6:1 regioisomeric mixture of **24h:24h’**:

^{1}H NMR (400 MHz, $CDCl_3$): δ_H 8.06-8.04 (m, 0.4H), 7.91-7.88 (m, 0.4H), 7.85-7.80 (m, 2H), 7.73 (m, 0.6H), 7.61-7.57 (m, 1H), 7.51-7.37 (m, 4.6H), 7.09-7.07 (m, 1H), 6.85 (s, 0.6H), 6.71 (s, 0.4H), 4.49 (s, 0.8H), 4.21 (s, 1.2H)

^{13}C NMR (101 MHz, $CDCl_3$): δ_C 157.7, 157.4, 151.0, 150.8, 134.1, 134.0, 133.7, 132.6, 132.5, 131.8, 130.4, 128.9, 128.6, 128.1, 128.0, 127.9, 127.8, 127.7, 127.4, 127.4, 127.3, 127.2, 127.0, 126.4, 126.4, 126.0, 125.9, 125.7, 125.3, 125.1, 123.7, 32.4, 29.7

IR (neat): ν_{max}/cm^{-1} 3052, 1721, 1599, 1494, 1424, 1257, 1114, 982, 722.

HRMS (NSI) [M+H]+ calcd. for $C_{18}H_{14}NOS$ 292.0791, found: 292.0791.

5-(3-Methylbenzyl)-2-(thiophen-2-yl)oxazole, 24j & 5-(4-methylbenzyl)-2-(thiophen-2-yl)oxazole, 24j’

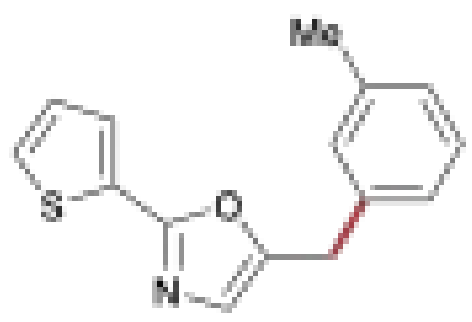
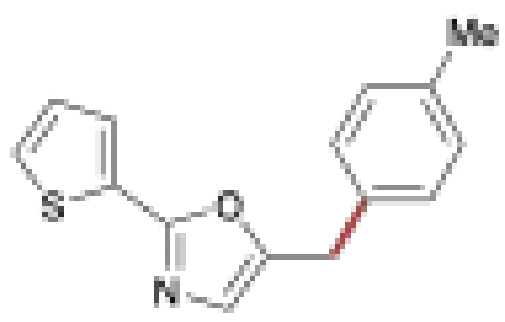

Prepared according to general procedure 2 for the synthesis of 2,5-disubstituted oxazoles (R = 2-thienyl): using *N*-propargylthiophene-2-carboxamide **20** (26.4 mg, 0.160 mmol), triphenylphosphine gold(I) bis[(trifluoromethyl)sulfonyl]amide (1.95 mg, 3.20 µmol, 2.0 mol %), 4-methyl-2-(trimethylsilyl)phenyl trifluoromethanesulfonate **11j** (56.2 mg, 0.180 mmol) and cesium fluoride (60.8 mg, 0.400 mmol). The crude reaction mixture was purified by silica gel flash chromatography to afford

the title compounds as an inseparable 1:1 mixture (20.0 mg, 0.0784 mmol, 49%) as a yellow oil; R_f 0.44 (2:1 *n*-hexane:ethyl acetate).

Data provided for a 1:1 regioisomeric mixture of **24j:24j'**:

^{1}H NMR (400 MHz, $CDCl_3$): δ_H 7.62-7.58 (m, 1H), 7.39-7.37 (m, 1H), 7.25-7.07 (m, 5H), 6.80 (s, 0.5H), 6.78 (s, 0.5H), 4.00 (s, 2H), 2.34 (s, 3H).

^{13}C NMR (101 MHz, $CDCl_3$): 157.5, 151.4, 151.2, 138.5, 136.7, 136.6, 133.6, 130.5, 129.6, 129.5, 128.7, 128.7, 127.9, 127.9, 127.9, 127.8, 127.3, 127.3, 125.8, 124.8, 124.7, 32.1, 31.8, 21.5, 21.2.

IR (neat): ν_{max}/cm^{-1} 3022, 2919, 1724, 1599, 1494, 1424, 1113, 981, 851, 722.

HRMS (NSI) [M+H]+ calcd. for $C_{15}H_{14}NOS$ 256.0791, found: 256.0793.

2.3 Synthesis and characterization of epoxyacenes

General procedure for the synthesis of substituted epoxacenes:

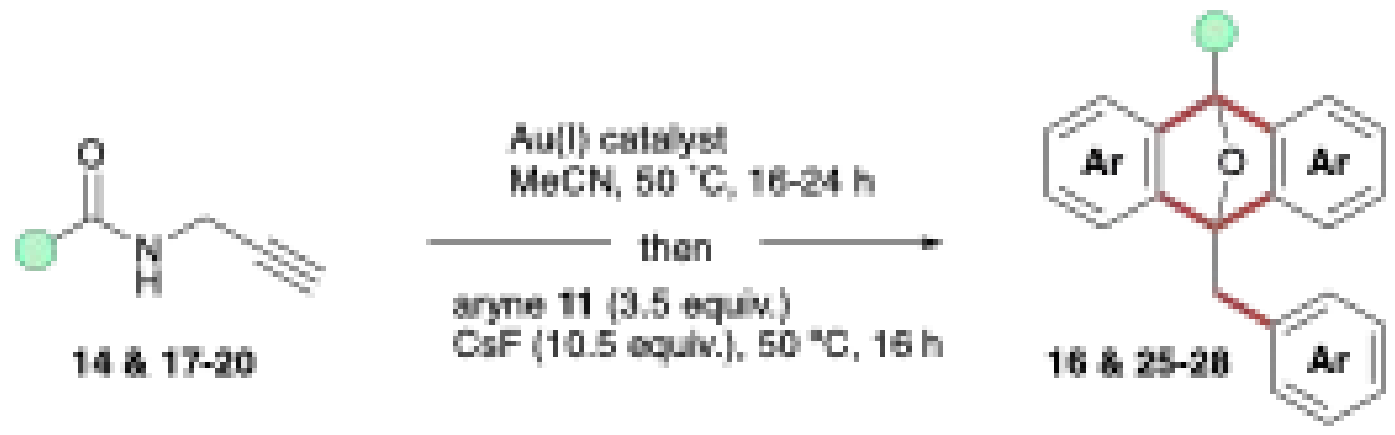

General procedure 1 for the synthesis of substituted epoxacenes (R = *t*-Bu, Me, Bn):

Propargylamide (1 eq) and triphenylphosphine gold(I) trifluoromethanesulfonate were dissolved in acetonitrile (0.15 M). The reaction mixture was stirred at the given temperature for between 4 to 24 h. Aryne precursor (3.5 equiv.) and cesium fluoride (10.5 equiv.) were then added and the reaction was stirred at 50 °C for 16 h. The solvent was removed *in vacuo* and the product was purified using silica gel flash column chromatography (*n*-hexane:ethyl acetate).

R group	Catalyst loading	Temp. (˚C)	Time (h)
t-Bu	1.0 mol%	20	4
Bn	4.0 mol%	50	24
Me	2.5 mol%	50	16

General procedure 2 for the synthesis of substituted epoxacenes (R = Ph, 2-thienyl):

Propargylamide (1 eq) and triphenylphosphine gold(I) bis[(trifluoromethyl)sulfonyl]amide (2.0 mol%) were dissolved in dichloromethane (0.30 M). The reaction mixture was stirred at 50 ˚C for 16 h. Aryne precursor (3.5 equiv.), cesium fluoride (10.5 equiv.) and acetonitrile (0.15 M) were then added and the reaction was stirred at 50 °C for 16 h. The solvent was removed *in vacuo* and the product was purified using silica gel flash column chromatography (*n*-hexane:ethyl acetate).

9-Benzyl-10-(tert-butyl)-9,10-dihydro-9,10-epoxyanthracene, 16a

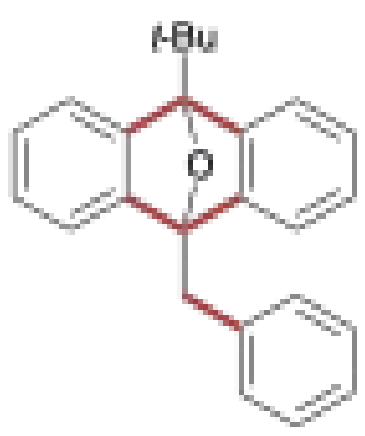

Prepared according to general procedure 1 for the synthesis of substituted epoxyacenes (R = *t*-Bu): using *N*-propargylpivalamide **14** (44.7 mg, 0.321 mmol), triphenylphosphine gold(I)

trifluoromethanesulfonate (1.93 mg, 3.21 µmol, 1.0 mol %), 2-(trimethylsilyl)phenyl trifluoromethanesulfonate **11a** (334 mg, 1.12 mmol) and cesium fluoride (539 mg, 3.37 mmol). The crude reaction mixture was purified by silica gel flash chromatography to afford the title compound (96.2 mg, 0.282 mmol, 88%) as a colourless oil; R_f 0.65 (2:1 *n*-hexane:ethyl acetate).

^{1}H NMR (400 MHz, CDCl$_3$): δ_H 7.50 (d, *J* = 7.5, 2H), 7.46-7.43 (m, 2H), 7.24 (t, *J* = 7.5, 2H), 7.16 (t, *J* = 7.5, 1H), 7.13-7.09 (m, 2H), 6.93-6.90 (m, 4H), 3.87 (s, 2H), 1.47 (s, 9H).

^{13}C NMR (101 MHz, CDCl$_3$): δ_C 152.4, 150.5, 136.7, 130.7, 128.1, 126.3, 125.3, 125.2, 121.5, 119.5, 96.3, 87.8, 34.1, 34.1, 27.2.

IR (neat): ν_{max}/cm^{-1} 2971, 1497, 1455, 1395, 1366, 1169, 1031, 925, 718, 669.

HRMS (NSI) [M+H]+ calcd. for $C_{25}H_{25}O$ 341.1900, found: 341.1904.

5-(Benzo[d][1,3]dioxol-5-ylmethyl)-11-(tert-butyl)-5,11-dihydro-5,11-epoxyanthra[2,3-d:6,7-d']bis([1,3]dioxole), 16b

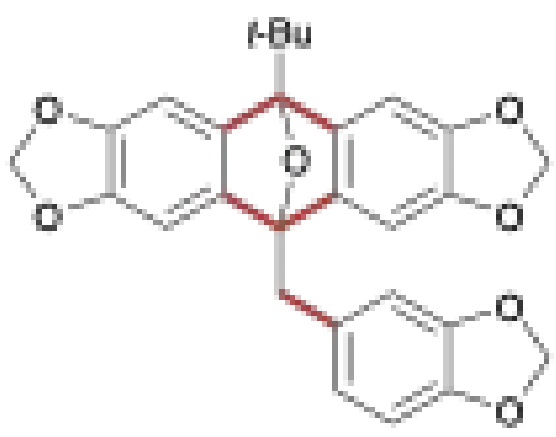

Prepared according to general procedure 1 for the synthesis of substituted epoxyacenes (R = *t*-Bu): using *N*-propargylpivalamide **14** (22.3 mg, 0.160 mmol), triphenylphosphine gold(I) trifluoromethanesulfonate (0.97 mg, 1.60 µmol, 1.0 mol %), 6-(trimethylsilyl)benzo[d][1,3]dioxol-5-yl trifluoromethanesulfonate **11b** (274 mg, 0.800 mmol, 5.0 equiv.) and cesium fluoride (365 mg, 2.40 mmol, 15 equiv.). The crude reaction mixture was purified by silica gel flash chromatography to afford the title compound (49.9 mg, 0.106 mmol, 66%) as a pale yellow solid; R_f 0.65 (2:1 *n*-hexane:ethyl acetate).

^{1}H NMR (400 MHz, CDCl$_3$): δ_H 7.01 (d, *J* = 1.5, 1H), 6.96 (s, 2H), 6.91 (dd, *J* = 8.0, 1.5, 1H), 6.71-6.69 (m, 1H), 6.60 (s, 2H), 5.89 (s, 2H), 5.88 (d, *J* = 1.5, 2H), 5.82 (d, *J* = 1.5 , 2H), 3.65 (s, 2H), 1.41 (s, 9H).

^{13}C NMR (101 MHz, CDCl$_3$): δ_C 147.5, 147.1, 146.2, 144.9, 144.6, 144.6, 130.2, 123.4, 111.1, 107.9, 104.3, 102.3, 101.4, 100.9, 96.5, 88.0, 34.0, 33.8, 27.2.

IR (neat): ν_{max}/cm^{-1} 3377, 2976, 2904, 2228, 1734, 1606, 1488, 1443, 1248, 920, 807, 728, 648.

HRMS (ASAP SOLID) [M+H]+ calcd. for $C_{28}H_{25}O_3$ 473.1595, found: 473.1598.

9-(*tert*-Butyl)-10-(3,4-difluorobenzyl)-2,3,6,7-tetrafluoro-9,10-dihydro-9,10-epoxyanthracene, 16c

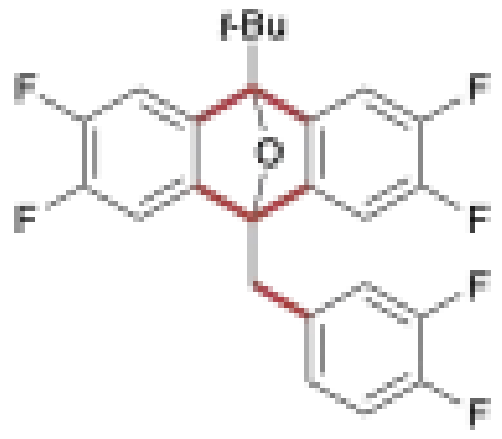

Prepared according to general procedure 1 for the synthesis of substituted epoxyacenes (R = *t*-Bu): using *N*-propargylpivalamide **14** (22.3 mg, 0.160 mmol), triphenylphosphine gold(I) trifluoromethanesulfonate (0.93 mg, 1.60 µmol, 1.0 mol %), 4,5-difluoro-2-(trimethylsilyl)phenyl trifluoromethanesulfonate **11c** (268 mg, 0.800 mmol, 5.0 equiv.) and cesium fluoride (365 mg, 2.40 mmol, 15 equiv.). The crude reaction mixture was purified by silica gel flash chromatography to afford the title compound (39.5 mg, 0.0881 mmol, 55%) as a yellow solid; R_f 0.55 (2:1 *n*-hexane:ethyl acetate).

^{1}H NMR (400 MHz, CDCl$_3$): δ_H 7.31-7.25 (m, 1H), 7.26 (dd, J = 9.3, 7.0, 2H), 7.17-7.11 (m, 1H), 7.06 (dt, J = 10.2, 8.2, 1H), 6.89 (dd, J = 8.3, 7.1, 2H), 3.71 (s, 2H), 1.40 (s, 9H).

^{13}C NMR (101 MHz, CDCl$_3$): δ_C 150.2 (dd, $J_{C\text{-}F}$ 247, 12.6), 149.6 (dd, $J_{C\text{-}F}$ 247, 12.7), 147.9 (dd, $J_{C\text{-}F}$ 251, 15.1), 147.6 (dd, $J_{C\text{-}F}$ 249, 14.8 Hz), 147.5 (t, $J_{C\text{-}F}$ 4.4), 145.8 (t, $J_{C\text{-}F}$ 4.4), 132.3 (dd, $J_{C\text{-}F}$ 6.0, 4.1), 126.3 (dd, $J_{C\text{-}F}$ 6.1, 3.6), 119.3 (d, $J_{C\text{-}F}$ 17.7), 117.1 (d, $J_{C\text{-}F}$ 17.1), 112.2 (dd, $J_{C\text{-}F}$ 18.6, 2.9), 109.8 (dd, $J_{C\text{-}F}$ 14.9, 6.0), 96.4, 87.4, 33.9, 33.0, 26.9 ppm

^{19}F NMR (376 MHz, CDCl$_3$): δ_F –(137.6-137.7) (m, 1F), –(139.8-139.9) (m, 4F), –(140.4-140.5) (m, 1F).

IR (neat): ν_{max}/cm^{-1} 2977, 1733, 1519, 1459, 1334, 1283, 1245, 1129, 877,773.

HRMS (ASAP) [M+H]+ calcd. for $C_{25}H_{19}F_6O$ 449.1335, found: 449.1334.

6-(*tert*-Butyl)-13-(naphthalen-2-ylmethyl)-6,13-dihydro-6,13-epoxypentacene, 16g

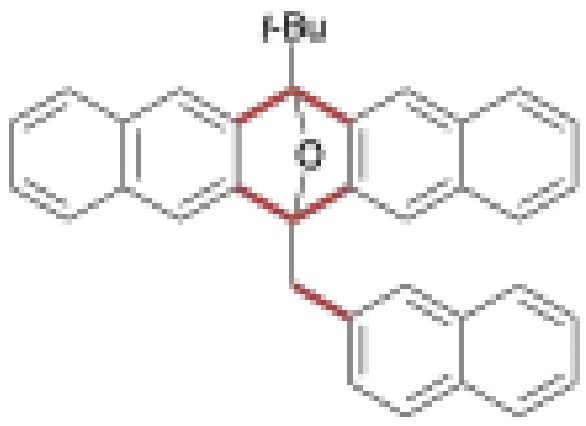

Prepared according to general procedure 1 for the synthesis of substituted epoxyacenes (R = *t*-Bu): using *N*-propargylpivalamide **14** (22.3 mg, 0.160 mmol), triphenylphosphine gold(I) trifluoromethanesulfonate (0.97 mg, 1.60 µmol, 1.0 mol %), 3-(trimethylsilyl)naphthalen-2-yl triflate **11g** (195 mg, 0.560 mmol) and cesium fluoride (367 mg, 0.1.68 mmol). *Note: following addition of the aryne precursor and CsF, reaction mixture heated at 80 ˚C for 16h.* The crude reaction mixture was purified by silica gel flash chromatography to afford the title compound (23.7 mg, 0.0483 mmol, 30%) as a yellow crystalline solid; R_f 0.65 (2:1 *n*-hexane:ethyl acetate).

^{1}H NMR (400 MHz, CDCl$_3$): δ_H 8.06 (s, 1H), 7.87 (s, 2H), 7.78 (d, *J* = 7.3, 1H), 7.76-7.71 (m, 3H), 7.56 (s, 2H), 7.41 (td, *J* = 7.4, 1.4, 1H), 7.70-7.67 (m, 2H), 7.66-7.63 (m, 2H), 7.39 (dd, *J* = 7.4, 1.4, 1H), 7.37-7.33 (m, 4H), 4.26 (s, 2H), 1.64 (s, 9H).

^{13}C NMR (101 MHz, CDCl$_3$): δ_C 148.1, 146.0, 134.3, 132.3, 132.2, 132.1, 129.2, 129.2, 128.4, 128.0, 127.8, 127.7, 127.6, 126.1, 126.0, 125.8, 125.4, 123.9, 120.1, 117.8, 95.4, 87.1, 34.6, 34.6, 27.4.

IR (neat): ν_{max}/cm^{-1} 3030, 2921, 1727, 1684, 1495, 1450, 1250, 1064, 985, 709, 691.

HRMS (ASAP) [M+H]+ calcd. for $C_{37}H_{31}O$ 491.2369, found: 491.2371.

Melting point: 120-122 ˚C (EtOH)

9-Benzyl-10-methyl-9,10-dihydro-9,10-epoxyanthracene, 25a

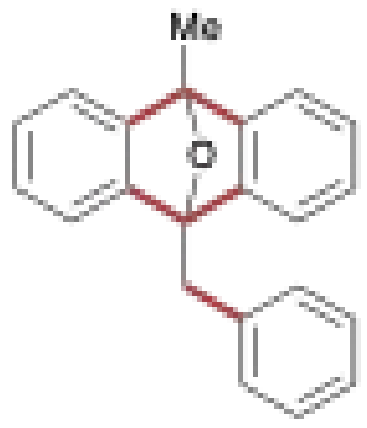

Prepared according to general procedure 1 for the synthesis of substituted epoxyacenes (R = Me): using *N*-(prop-2-yn-1-yl)acetamide **17** (14.9 mg, 0.153 mmol), triphenylphosphine gold(I) trifluoromethanesulfonate (2.43 mg, 4.00 µmol, 2.5 mol %), 2-(trimethylsilyl)phenyl trifluoromethanesulfonate **11a** (162 mg, 0.543 mmol) and cesium fluoride (257 mg, 1.61 mmol). The crude reaction mixture was purified by silica gel flash chromatography to afford the title compound (32.7 mg, 0.110 mmol, 72%) as a pale yellow solid; R_f 0.35 (2:1 *n*-hexane:ethyl acetate).

^{1}H NMR (400 MHz, CDCl$_3$): δ_H 7.49 (d, *J* = 7.3, 2H), 7.26 (t, *J* = 7.6, 2H), 7.20-7.14 (m, 5H), 6.99-6.94 (m, 4H), 3.91 (s, 2H), 2.11 (s, 3H).

^{13}C NMR (101 MHz, CDCl$_3$): δ_C 152.0, 150.8, 136.4, 130.7, 128.2, 126.5, 125.7, 125.6, 119.7, 118.7, 89.6, 87.0, 34.5, 14.1.

IR (neat): ν_{max}/cm^{-1} 3069, 3031, 2930, 1737, 1595, 1493, 1366, 1272, 1214, 908, 730.

HRMS (NSI) [M+H]+ calcd. for $C_{22}H_{19}O$ 299.143, found: 299.1433.

5-(Benzo[d][1,3]dioxol-5-ylmethyl)-11-methyl-5,11-dihydro-5,11-epoxyanthra[2,3-d:6,7-d']bis([1,3]dioxole), 25b

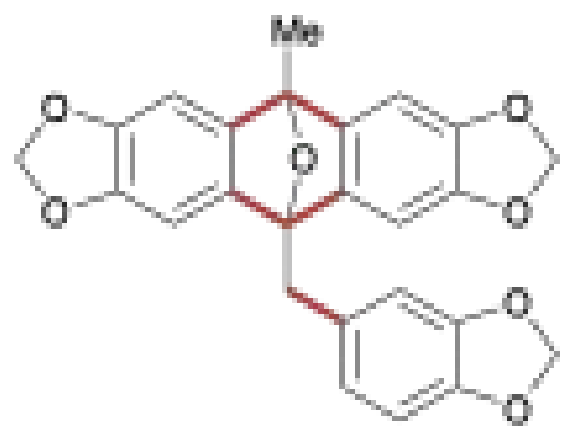

Prepared according to general procedure 1 for the synthesis of substituted epoxyacenes (R = Me): using *N*-(prop-2-yn-1-yl)acetamide **17** (15.5 mg, 0.160 mmol), triphenylphosphine gold(I) trifluoromethanesulfonate (2.43 mg, 4.00 µmol, 2.5 mol %), 6-(trimethylsilyl)benzo[d][1,3]dioxol-5-yl trifluoromethanesulfonate **11b** (274 mg, 0.800 mmol, 5.0 equiv.) and cesium fluoride (365 mg, 2.40 mmol, 15 equiv.). The crude reaction mixture was purified by silica gel flash chromatography to afford the title compound (27.5 mg, 0.0639 mmol, 40%) as a colourless solid; R_f 0.28 (2:1 *n*-hexane:ethyl acetate).

^{1}H NMR (400 MHz, $CDCl_3$): δ_H 7.00 (d, *J* = 1.5, 1H), 6.90 (dd, *J* = 7.9, 1.5, 1H), 6.72 (d, *J* = 7.8, 1H), 6.66 (s, 2H), 6.66 (s, 2H), 5.90 (s, 2H), 5.89 (d, *J* = 1.4, 2H), 5.83 (d, *J* = 1.4, 2H), 3.69 (s, 2H), 2.00 (s, 3H).

^{13}C NMR (101 MHz, $CDCl_3$): δ_C 147.5, 146.8, 146.3, 145.4, 145.1, 145.0, 129.8, 123.5, 111.1, 108.0, 102.6, 101.8, 101.4, 100.9, 89.7, 87.2, 34.2, 14.2.

IR (neat): ν_{max}/cm^{-1} 3041, 2989, 1729, 1587, 1510, 1488, 1444, 1248, 920, 815, 723, 658.

HRMS (ASAP (SOLID)) [M+H]+ calcd. for $C_{25}H_{19}O_7$ 431.1131, found: 431.1125.

9-(3,4-Diuorobenzyl)-2,3,6,7-tetrauoro-10-methyl-9,10-dihydro-9,10-epoxyanthracene, 25c

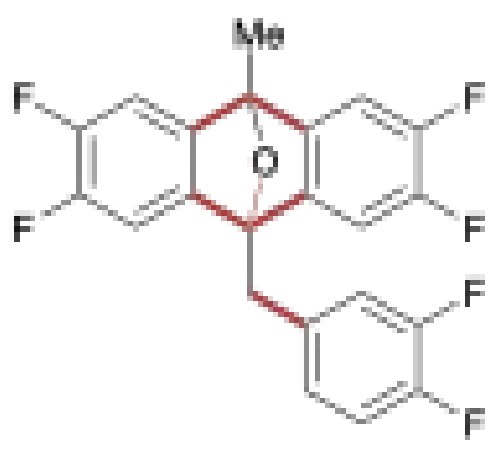

Prepared according to general procedure 1 for the synthesis of substituted epoxyacenes (R = Me): using *N*-(prop-2-yn-1-yl)acetamide **17** (15.5 mg, 0.160 mmol), triphenylphosphine gold(I) trifluoromethanesulfonate (2.43 mg, 4.00 µmol, 2.5 mol %), 4,5-difluoro-2-(trimethylsilyl)phenyl trifluoromethanesulfonate **11c** (262 mg, 0.800 mmol, 5.0 equiv.) and cesium fluoride (384 mg, 2.40 mmol, 15 equiv.). The crude reaction mixture was purified by silica gel flash chromatography to afford the title compound (8.4 mg, 0.0208 mmol, 13%) as a pale yellow oil; R_f 0.16 (2:1 *n*-hexane:ethyl acetate).

^{1}H NMR (400 MHz, $CDCl_3$): δ_H 7.27 (d, *J* = 1.7, 1H), 7.14 (dd, *J* = 8.5 1.7, 1H), 7.07 (d, *J* = 8.5, 1H), 6.98 (s, 2H), 6.92 (s, 2H), 3.73 (s, 2H), 2.05 (s, 3H).

^{13}C NMR (101 MHz, CDCl$_3$): δ_C 150.2 (dd, $J_{C\text{-}F}$ 248, 13.1), 149.7 (dd, $J_{C\text{-}F}$ 249, 13.1), 148.4 (dd, $J_{C\text{-}F}$ 252, 16.6), 148.2 (dd, $J_{C\text{-}F}$ 247, 11.2), 147.6 (dd, $J_{C\text{-}F}$ 5.5, 3.3), 145.9 (dd, $J_{C\text{-}F}$ 5.5, 3.3), 132.0 (dd, $J_{C\text{-}F}$ 6.5, 4.4), 126.3 (dd, $J_{C\text{-}F}$ 5.9, 3.3), 119.4 (d, $J_{C\text{-}F}$ 17.6), 117.2 (d, $J_{C\text{-}F}$ 17.1), 110.2 (d, $J_{C\text{-}F}$ 20.3), 109.7 (d, $J_{C\text{-}F}$ 20.1), 89.0, 87.0, 33.3, 13.9.

^{19}F NMR (376 MHz, CDCl$_3$): δ_F –137.5 (d, $J_{F\text{-}F}$ 21.4, 1F), –139.5 (d, $J_{F\text{-}F}$ 18.2, 2F), –139.7 (d, $J_{F\text{-}F}$ 18.2, 2F), –140.2 (d, $J_{F\text{-}F}$ 21.4, 1F).

IR (neat): ν_{max}/cm^{-1} 3056, 1609, 1514, 1489, 1250, 1191, 960, 729, 630.

HRMS (NSI) [M+H]+ calcd. for $C_{22}H_{12}F_6O$ 407.0871, found: 407.0867.

9,10-Dibenzyl-9,10-dihydro-9,10-epoxyanthracene, 26a

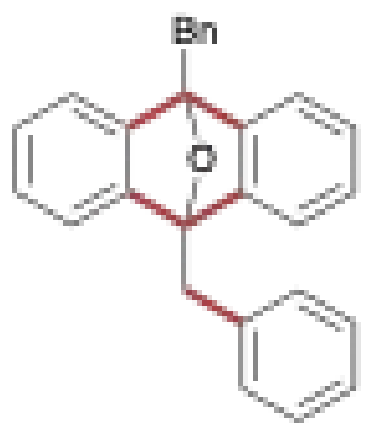

Prepared according to general procedure 1 for the synthesis of substituted epoxyacenes (R = Bn): using 2-phenyl-*N*-(prop-2-yn-1-yl)acetamide **18** (50.8 mg, 0.293 mmol), triphenylphosphine gold(I) trifluoromethanesulfonate (7.13 mg, 11.7 µmol, 4.0 mol %), 2-(trimethylsilyl)phenyl trifluoromethanesulfonate **11a** (306 mg, 1.03 mmol) and cesium fluoride (492 mg, 3.08 mmol). The crude reaction mixture was purified by silica gel flash chromatography to afford the title compound (58.1 mg, 0.155 mmol, 53%) as a yellow solid; R_f 0.59 (2:1 *n*-hexane:ethyl acetate).

^{1}H NMR (400 MHz, CDCl$_3$): δ_H 7.50 (d, J = 7.2, 4H), 7.24 (t, J = 7.2, 4H), 7.17 (m, 2H), 7.16-7.10 (m, 4H), 6.93-6.88 (m, 4H), 3.91 (s, 4H).

^{13}C NMR (101 MHz, CDCl$_3$): δ_C 151.0, 136.4, 130.7, 128.1, 126.4, 125.6, 119.6, 89.5, 34.3.

IR (neat): ν_{max}/cm^{-1} 3375, 2991, 2904, 2238, 1734, 1490, 1443, 1248, 920, 728, 648.

HRMS (ASAP (SOLID)) [M+H]+ calcd. for $C_{28}H_{23}O$ 375.1749, found: 375.1747.

5-(Benzo[d][1,3]dioxol-5-ylmethyl)-11-benzyl-5,11-dihydro-5,11-epoxyanthra[2,3-d:6,7-d']bis([1,3]dioxole), 26b

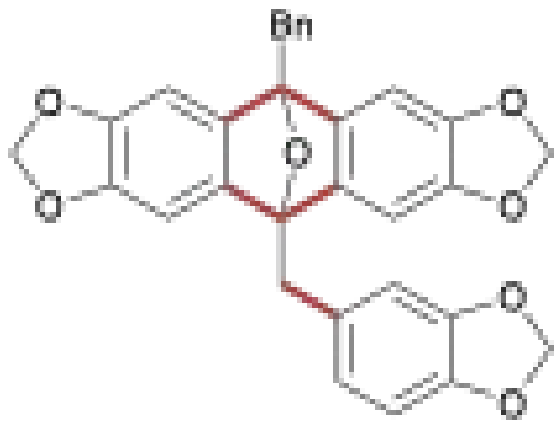

Prepared according to general procedure 1 for the synthesis of substituted epoxyacenes (R = Bn): using 2-phenyl-*N*-(prop-2-yn-1-yl)acetamide **18** (27.7 mg, 0.160 mmol), triphenylphosphine gold(I) trifluoromethanesulfonate (0.97 mg, 1.60 µmol, 4.0 mol %), 6-(trimethylsilyl)benzo[d][1,3]dioxol-5-yl trifluoromethanesulfonate **11b** (274 mg, 0.800 mmol, 5.0 equiv.) and cesium fluoride (365 mg, 2.40 mmol, 15 equiv.). The crude reaction mixture was purified by silica gel flash chromatography to afford the title compound (40.5 mg, 0.080 mmol, 50%) as a yellow solid; R_f 0.52 (2:1 *n*-hexane:ethyl acetate).

^{1}H NMR (400 MHz, CDCl$_3$): δ_H 7.48 (d, *J* = 7.2, 2H), 7.27 (t, *J* = 7.2, 2H), 7.19 (t, *J* = 7.2, 1H), 7.02 (s, 1H), 6.90 (d, *J* = 7.7, 1H), 6.70 (d, *J* = 7.9, 1H), 6.65 (s, 2H), 6.62 (s, 2H), 5.90 (s, 2H), 5.85 (s, 2H), 5.81 (s, 2H), 3.77 (s, 2H), 3.68 (s, 2H).

^{13}C NMR (101 MHz, CDCl$_3$): δ_C 147.6, 146.3, 145.8, 145.7, 144.9, 144.9, 136.2, 130.6, 129.9, 128.2, 126.6, 123.5, 111.2, 107.9, 102.6, 102.5, 101.4, 100.9, 89.7, 89.7, 34.3, 34.0.

IR (neat): ν_{max}/cm^{-1} 2908, 1738, 1608, 1455, 1250, 1037, 932.

HRMS (ASAP) [M+H]+ calcd. for $C_{31}H_{23}O_7$ 507.1444, found: 507.1444.

9-Benzyl-10-(3,4-diuorobenzyl)-2,3,6,7-tetrauoro-9,10-dihydro-9,10-epoxyanthracene, 26c

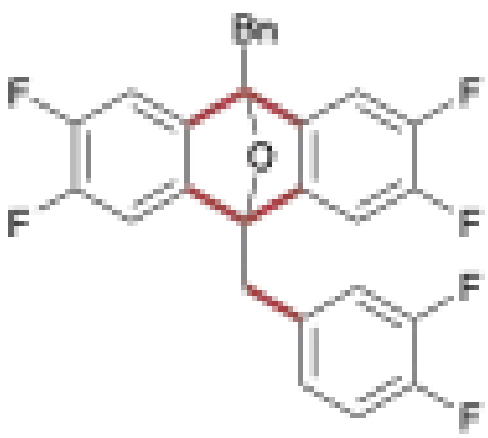

Prepared according to general procedure 1 for the synthesis of substituted epoxyacenes (R = Bn): using 2-phenyl-*N*-(prop-2-yn-1-yl)acetamide **18** (27.7 mg, 0.160 mmol), triphenylphosphine gold(I) trifluoromethanesulfonate (3.89 mg, 6.40 µmol, 4.0 mol %), 4,5-difluoro-2-(trimethylsilyl)phenyl trifluoromethanesulfonate **11c** (268 mg, 0.800 mmol) and cesium fluoride (365 mg, 2.40 mmol). The crude reaction mixture was purified by silica gel flash chromatography to afford the title compound (15.4 mg, 0.0319 mmol, 20%) as a pale yellow solid; R_f 0.45 (2:1 *n*-hexane:ethyl acetate).

^{1}H NMR (400 MHz, CDCl$_3$): 7.42 (d, *J* = 7.4, 2H), 7.31-7.27 (m, 1H), 7.30 (t, *J* = 7.4, 2H), 7.23 (m, 1H), 7.14-7.11 (m, 1H), 7.05 (m, 1H), 6.96 (dd, *J* = 8.7, 6.6, 2H), 6.89 (dd, *J* = 8.6, 6.5, 2H), 3.80 (s, 2H), 3.74 (s, 2H).

^{13}C NMR (101 MHz, CDCl$_3$): δ_C 150.6 (dd, $J_{C\text{-}F}$ 247.5, 12.7), 150.1 (dd, $J_{C\text{-}F}$ 247.4, 12.7), 148.2 (dd, $J_{C\text{-}F}$ 251, 14.7), 148.2 (dd, $J_{C\text{-}F}$ 254, 17.6), 146.5 (dd, $J_{C\text{-}F}$ 5.4, 3.3), 146.2 (dd, $J_{C\text{-}F}$ 5.4, 3.3), 134.9,

132.1 (dd, J_{C-F} 6.0, 4.1), 130.4, 128.5, 127.2, 126.3 (dd, J_{C-F} 5.4, 3.3), 119.5 (d, J_{C-F} 18.2), 117.1 (d, J_{C-F} 16.7), 110.5 (d, J_{C-F} 20.3), 110.1 (d, J_{C-F} 20.4), 89.6, 89.0, 34.1, 33.1.

^{19}F NMR (376 MHz, $CDCl_3$): δ_F –(137.6-137.7) (m, 1F), –(139.3-139.4) (m, 2F), –(139.5-139.6) (m, 2F), –(140.2-140.3) (m, 1F).

IR (neat): ν_{max}/cm^{-1} 3111, 1620, 1518, 1461, 1434, 1358, 1119, 877, 765.

HRMS (ASAP) [M+H]+ calcd. for $C_{28}H_{17}F_6O$ 483.1178, found: 483.1179.

9-Benzyl-10-phenyl-9,10-dihydro-9,10-epoxyanthracene, 27a

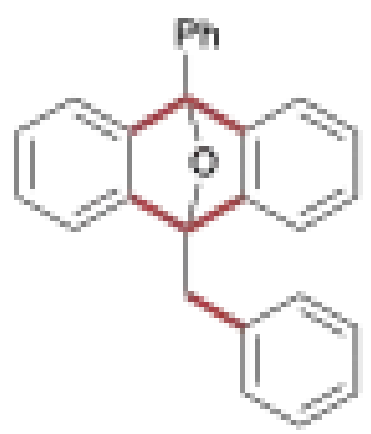

Prepared according to general procedure 2 for the synthesis of substituted epoxyacenes (R = Ph): using *N*-(prop-2-yn-1-yl)benzamide **19** (25.5 mg, 0.160 mmol), triphenylphosphine gold(I) bis[(trifluoromethyl)sulfonyl]amide (1.95 mg, 3.20 µmol, 2.0 mol %), 2-(trimethylsilyl)phenyl trifluoromethanesulfonate **11a** (167 mg, 0.560 mmol) and cesium fluoride (255 mg, 1.68 mmol). The crude reaction mixture was purified by silica gel flash chromatography to afford the title compound (49.6 mg, 0.138 mmol, 86%) as a yellow solid; R_f 0.47 (2:1 *n*-hexane:ethyl acetate).

^{1}H NMR (400 MHz, $CDCl_3$): δ_H 7.87 (d, *J* = 7.4, 2H), 7.58 (t, *J* = 7.5, 4H), 7.48 (t, *J* = 7.4, 1H), 7.30-7.23 (m, 6H), 7.20 (t, *J* = 7.4, 1H), 7.02-6.94 (m, 4H), 4.03 (s, 2H).

^{13}C NMR (101 MHz, $CDCl_3$): δ_C 151.0, 150.4, 136.3, 135.4, 130.7, 128.8, 128.2, 128.2, 126.6, 126.5, 125.9, 125.6, 120.1, 119.9, 90.4, 89.7, 34.4.

IR (neat): ν_{max}/cm^{-1} 3452, 3029, 1734, 1601, 1496, 1447, 1322, 1243, 1032, 1001, 980, 761.

HRMS (ASAP) [M+H]+ calcd. for $C_{27}H_{21}O$ 361.1592, found: 361.1590.

5-(Benzo[d][1,3]dioxol-5-ylmethyl)-11-phenyl-5,11-dihydro-5,11-epoxyanthra[2,3-d:6,7-d']bis([1,3]dioxole), 27b

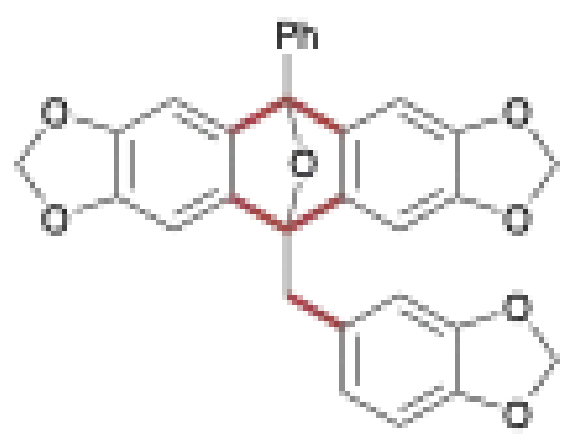

Prepared according to general procedure 2 for the synthesis of substituted epoxyacenes (R = Ph): using *N*-(prop-2-yn-1-yl)benzamide **19** (25.5 mg, 0.160 mmol), triphenylphosphine gold(I) bis[(trifluoromethyl)sulfonyl]amide (1.95 mg, 3.20 µmol, 2.0 mol %), 6-(trimethylsilyl)benzo[d][1,3]dioxol-5-yl trifluoromethanesulfonate **11b** (274 mg, 0.800 mmol, 5.0 equiv.) and cesium fluoride (365 mg, 2.40 mmol, 15 equiv.). The crude reaction mixture was purified by silica gel flash chromatography to afford the title compound (63.0 mg, 0.128 mmol, 80%) as a yellow oil; R_f 0.47 (2:1 *n*-hexane:ethyl acetate).

^{1}H NMR (400 MHz, CDCl$_3$): δ_H 7.72 (d, *J* = 7.2, 2H), 7.55 (t, *J* = 7.6, 2H), 7.46 (m, 1H), 7.08 (d, *J* = 1.4 Hz, 1H), 6.97 (dd, *J* = 7.9, 1.4, 1H), 6.77 (s, 2H), 6.73 (d, *J* = 7.9, 1H), 6.72 (s, 2H), 5.90 (s, 2H), 5.87 (d, *J* = 1.3, 2H), 5.84 (d, *J* = 1.3, 2H), 3.79 (s, 2H).

^{13}C NMR (101 MHz, CDCl$_3$): δ_C 147.6, 146.3, 145.8, 145.2, 145.2, 145.0, 135.1, 129.7, 128.9, 128.4, 126.5, 123.6, 111.2, 108.1, 103.2, 102.7, 101.5, 100.9, 90.6, 89.8, 34.1.

IR (neat): ν_{max}/cm^{-1} 2902, 1724, 1648, 1610, 1502, 1475, 1444, 1273, 1249, 1212, 1037, 931, 731.

HRMS (ASAP) [M+H]+ calcd. for $C_{30}H_{21}O_7$ 493.1287, found: 493.1294.

9-(3,4-Diuorobenzyl)-2,3,6,7-tetrauoro-10-phenyl-9,10-dihydro-9,10-epoxyanthracene, 27c

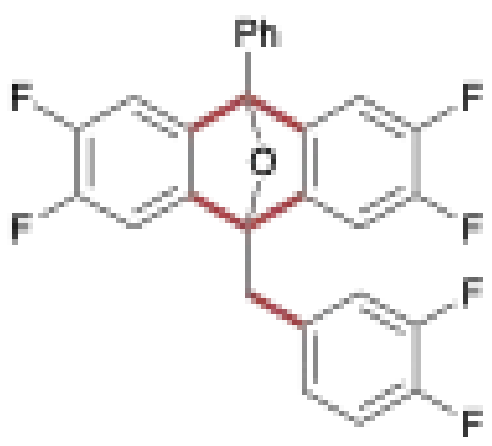

Prepared according to general procedure 2 for the synthesis of substituted epoxyacenes (R = Ph): using *N*-(prop-2-yn-1-yl)benzamide **19** (25.5 mg, 0.160 mmol), triphenylphosphine gold(I) bis[(trifluoromethyl)sulfonyl]amide (1.95 mg, 3.20 µmol, 2.0 mol %), 4,5-difluoro-2-(trimethylsilyl)phenyl trifluoromethanesulfonate **11c** (275 mg, 0.800 mmol, 5.0 equiv.) and cesium fluoride (365 mg, 2.40 mmol, 15 equiv.). The crude reaction mixture was purified by silica gel flash chromatography to afford the title compound (22.5 mg, 0.0480 mmol, 30%) as a yellow solid; R_f 0.52 (2:1 *n*-hexane:ethyl acetate).

^{1}H NMR (400 MHz, CDCl$_3$): 7.73-7.71 (m, 2H), 7.60 (t, *J* = 7.5, 2H), 7.52 (m, 1H), 7.35 (m, 1H), 7.09 (m, 1H), 7.09 (dd, *J* = 8.5, 7.0, 3H), 7.00 (dd, *J* = 8.2, 6.9, 2H), 3.84 (s, 2H).

^{13}C NMR (101 MHz, CDCl$_3$): δ_C 150.3 (dd, $J_{C\text{-}F}$ 247, 12.0), 149.6 (dd, $J_{C\text{-}F}$ 247, 9.8), 148.4 (dd, $J_{C\text{-}F}$ 251, 15.4), 148.2 (dd, $J_{C\text{-}F}$ 251, 14.9), 146.8 (dd, $J_{C\text{-}F}$ 5.5, 3.6), 145.7 (dd, $J_{C\text{-}F}$ 5.5, 3.4), 133.5, 132.0 (dd, $J_{C\text{-}F}$ 5.7, 3.0), 129.3, 129.2, 126.4 (dd, $J_{C\text{-}F}$ 5.9, 3.6), 126.2, 119.4 (d, $J_{C\text{-}F}$ 17.7), 117.3 (d, $J_{C\text{-}F}$ 17.2), 111.2 (dd, $J_{C\text{-}F}$ 17.7 3.5), 110.3 (d, $J_{C\text{-}F}$ 19.8), 90.4, 89.2, 33.4.

^{19}F NMR (376 MHz, CDCl$_3$): δ_F –(137.3-137.4) (m, 1F), –(139.1-139.3) (m, 4F), –(140.0-140.2) (m, 1F).

IR (neat): ν_{max}**/cm^{-1}** 3030, 1620, 1519, 1461, 1382, 1284, 1213, 1172, 1016, 879, 774.

HRMS (ASAP) [M+H]+ calcd. for $C_{27}H_{15}F_6O$ 469.1027, found: 469.1025.

9-Benzyl-10-(thiophen-2-yl)-9,10-dihydro-9,10-epoxyanthracene, 28a

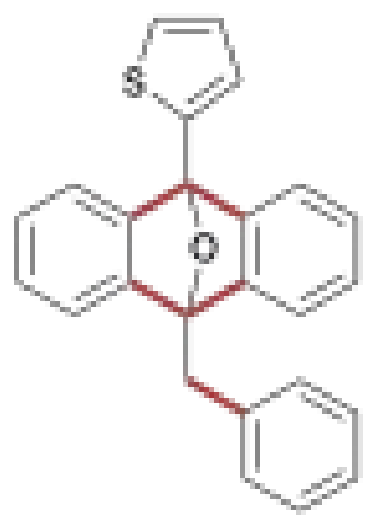

Prepared according to general procedure 2 for the synthesis of substituted epoxyacenes (R = 2-thienyl): using *N*-propargylthiophene-2-carboxamide **20** (26.4 mg, 0.160 mmol), triphenylphosphine gold(I) bis[(trifluoromethyl)sulfonyl]amide (1.95 mg, 3.20 μmol, 2.0 mol %), 2-(trimethylsilyl)phenyl trifluoromethanesulfonate **11a** (167 mg, 0.560 mmol) and cesium fluoride (255 mg, 1.68 mmol). The crude reaction mixture was purified by silica gel flash chromatography to afford the title compound (44.6 mg, 0.122 mmol, 76%) as a yellow solid; R_f 0.50 (2:1 *n*-hexane:ethyl acetate).

^{1}H NMR (400 MHz, CDCl$_3$): δ_H 7.63-7.59 (m, 1H), 7.57-7.51 (m, 3H), 7.41-7.36 (m, 2H), 7.29-7.19 (m, 6H), 7.04-6.99 (m, 4H), 3.99 (s, 2H).

^{13}C NMR (101 MHz, CDCl$_3$): δ_C 150.7, 150.7, 137.2, 136.2, 130.7, 128.2, 128.1, 127.5, 127.1, 126.5, 126.1, 125.8, 120.7, 119.9, 89.7, 88.4, 34.3.

IR (neat): ν_{max}**/cm^{-1}** 3499, 3065, 3029, 1733, 1666, 1599, 1454, 1320, 1275, 1250, 931, 689.

HRMS (ASAP) [M+H]+ calcd. for $C_{25}H_{10}OS$ 367.1157, found: 367.1154.

5-(Benzo[d][1,3]dioxol-5-ylmethyl)-11-(thiophen-2-yl)-5,11-dihydro-5,11-epoxyanthra[2,3-d:6,7-d']bis([1,3]dioxole), 28b

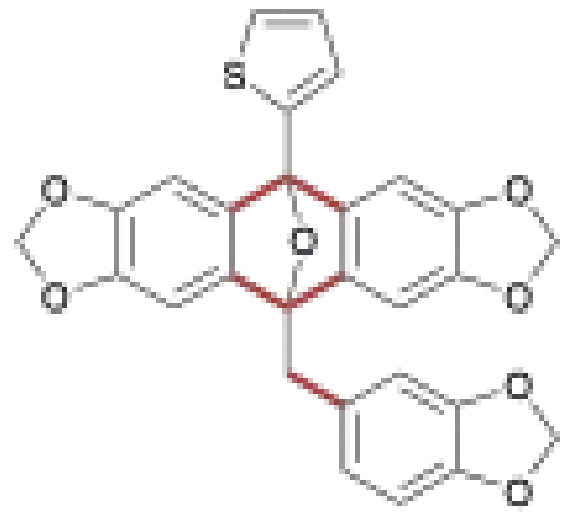

Prepared according to general procedure 2 for the synthesis of substituted epoxyacenes (R = 2-thienyl): using *N*-propargylthiophene-2-carboxamide **20** (26.4 mg, 0.160 mmol), triphenylphosphine gold(I) bis[(trifluoromethyl)sulfonyl]amide (1.95 mg, 3.20 μmol, 2.0 mol %), 6-(trimethylsilyl)benzo[d][1,3]dioxol-5-yl trifluoromethanesulfonate **11b** (274 mg, 0.800 mmol, 5.0

equiv.) and cesium fluoride (365 mg, 2.40 mmol, 15 equiv.). The crude reaction mixture was purified by silica gel flash chromatography to afford the title compound (47.9mg, 0.0961 mmol, 60%) as a yellow oil; R_f 0.43 (2:1 *n*-hexane:ethyl acetate).

^{1}H NMR (400 MHz, CDCl$_3$): δ_H 7.51 (s, 2H), 7.30 (dd, *J* = 5.1, 0.8, 1H), 7.26 (dd, *J* = 3.6, 0.8, 1H), 7.04 (dd, *J* = 5.1, 3.6, 1H), 6.72 (s, 2H), 6.31 (d, *J* = 7.9, 1H), 6.08 (d, *J* = 1.0, 2H), 6.01 (d, *J* = 1.0, 2H), 5.77 (s, 2H), 5.58 (dd, *J* = 8.0, 1.6, 1H), 5.54 (d, *J* = 1.5, 1H), 3.61 (s, 2H).

^{13}C NMR (101 MHz, CDCl$_3$): δ_C 180.2, 152.0, 151.8, 147.5, 146.6, 146.4, 143.6, 128.2, 127.1, 126.4, 126.0, 125.5, 123.2, 110.0, 107.9, 107.4, 105.3, 101.9, 100.8, 52.2, 51.1.

IR (neat): ν_{max}/cm^{-1} 2908, 1732, 1649, 1610, 1502, 1478, 1287, 1245, 1037, 933, 729.

HRMS (ASAP) [M+H]+ calcd. for $C_{28}H_{19}O_7S$ 499.0851, found: 499.0847.

9-(3,4-Difluorobenzyl)-2,3,6,7-tetrafluoro-10-(thiophen-2-yl)-9,10-dihydro-9,10-epoxyanthracene, 28c

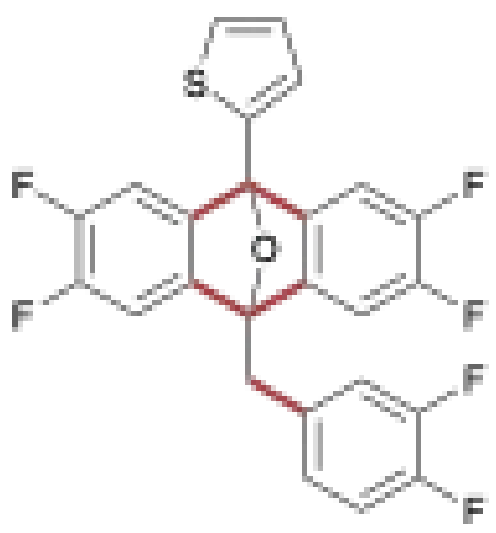

Prepared according to general procedure 2 for the synthesis of substituted epoxyacenes (R = 2-thienyl): using *N*-propargylthiophene-2-carboxamide **20** (26.4 mg, 0.160 mmol), triphenylphosphine gold(I) bis[(trifluoromethyl)sulfonyl]amide (1.95 mg, 3.20 µmol, 2.0 mol %), 4,5-difluoro-2-(trimethylsilyl)phenyl trifluoromethanesulfonate **11c** (275 mg, 0.800 mmol, 5.0 equiv.) and cesium fluoride (365 mg, 2.40 mmol, 15 equiv.). The crude reaction mixture was purified by silica gel flash chromatography to afford the title compound (25.0 mg, 0.0527 mmol, 33%) as a yellow solid; R_f 0.58 (2:1 *n*-hexane:ethyl acetate).

^{1}H NMR (400 MHz, CDCl$_3$): 7.80 (dd, *J* = 12.3, 7.9, 2H), 7.66 (dd, *J* = 5.2, 0.8, 1H), 7.55 (dd, *J* = 12.1, 8.4, 2H), 7.33 (dd, *J* = 5.1, 3.5, 1H), 7.19 (dd, *J* = 3.4, 1.0, 1H), 7.07 (dt, *J* = 9.7, 8.7, 1H), 6.86-6.80 (m, 2H), 4.80 (s, 2H).

^{13}C NMR (101 MHz, CDCl$_3$): δ_C 150.2 (dd, $J_{C\text{-}F}$ 248, 12.7), 149.6 (dd, $J_{C\text{-}F}$ 247, 12.7), 147.8 (dd, $J_{C\text{-}F}$ 250, 22.9), 147.7 (dd, $J_{C\text{-}F}$ 250, 22.7), 147.5 (dd, $J_{C\text{-}F}$ 6.0, 4.1), 145.8 (dd, $J_{C\text{-}F}$ 6.1, 3.6), 137.6, 132.3 (dd, $J_{C\text{-}F}$ 6.0, 4.1), 130.0, 127.9, 127.8, 126.3 (dd, $J_{C\text{-}F}$ 6.1, 3.6), 117.9 (d, $J_{C\text{-}F}$ 17.7), 116.9 (d, $J_{C\text{-}F}$ 17.1), 112.9 (dd, $J_{C\text{-}F}$ 18.6 2.9), 109.8 (dd, $J_{C\text{-}F}$ 14.9 6.0), 90.4, 89.2, 34.0.

^{19}F NMR (376 MHz, CDCl$_3$): δ_F –(133.2-133.3) (m, 2F), –(133.8-139.9) (m, 2F), –(136.8-137.0) (m, 1F), –(140.4-140.5) (m, 1F).

IR (neat): ν_{max}/cm^{-1} 3111, 1722, 1609, 1518, 1424, 1281, 1250, 1115, 983, 723, 691.

HRMS (ASAP) [M+H]+ calcd. for $C_{25}H_{13}F_6OS$ 475.0591, found: 475.0594.

9-(*tert*-Butyl)-10-(3-methoxybenzyl)-9,10-dihydro-9,10-epoxyanthracene, 16fa

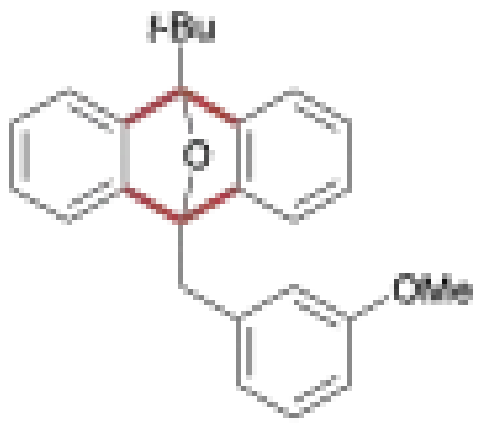

2-(*tert*-Butyl)-5-(3-methoxybenzyl)oxazole **16f** (18.8 mg, 0.0766 mmol) was dissolved in acetonitrile (0.51 mL). 2-(Trimethylsilyl)phenyl trifluoromethanesulfonate **11a** (57.3 mg, 0.192 mmol, 2:5 eq) and cesium fluoride (72.8 mg, 0.479 mmol, 6:25 equiv.) were added and the reaction was heated at 50 °C for 16 h. The solvent was removed *in vacuo* and the product was purified using flash column chromatography (*n*-hexane:ethyl acetate) to afford the title compound (25.3 mg, 0.0683 mmol, 89%) as a yellow solid.

^{1}H NMR (400 MHz, CDCl$_3$): δ_H 7.48-7.44 (m, 2H), 7.17 (t, *J* = 7.9, 1H), 7.14-7.08 (m, 4H), 6.94-6.91 (m, 4H), 6.73 (dd, *J* = 8.1, 2.1, 1H), 3.87 (s, 2H), 3.72 (s, 3H), 1.49 (s, 9H).

^{13}C NMR (101 MHz, CDCl$_3$): δ_C 159.4, 152.3, 150.4, 138.3, 128.9, 125.4, 125.2, 123.0, 121.5, 119.5, 115.9, 112.4, 96.3, 87.8, 55.1, 34.0, 27.2.

IR (neat): ν_{max}/cm^{-1} 3301, 3003, 2976, 1732, 1676, 1593, 1454, 1265, 1119, 971, 845, 689.

HRMS (ESI) [M+H]+ calcd. for $C_{26}H_{27}O_2$ 371.2006, found: 371.2033.

2.4 Synthesis and characterization of 9,10-dibenzylanthracene, 29a

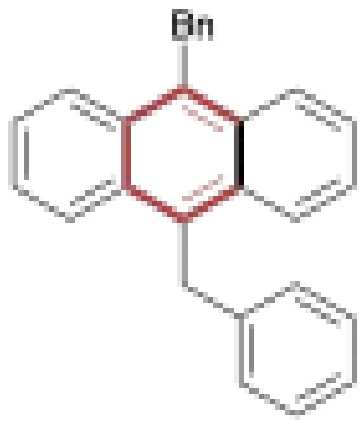

From 9,10-epoxyanthracene:

9,10-Dibenzyl-9,10-dihydro-9,10-epoxyanthracene **26a** (59.8 mg, 0.160 mmol) was dissolved in a mixture of acetonitrile (1.8 mL) and dichloromethane (0.2 mL). Sodium iodide (72.0 mg, 0.480 mmol) and chlorotrimethylsilane (61 µL, 0.480 mmol) were added and the reaction was stirred for 30 min at room temperature. The solvent was removed *in vacuo* and the product was purified using flash column chromatography (*n*-hexane:dichloromethane) to afford the title compound (55.0 mg, 0.154 mmol, 96%) as a pale yellow solid; R_f 0.53 (2:1 Petrol 40-60:ethyl acetate).

One-pot process from propargyl amide:

2-Phenyl-*N*-(prop-2-yn-1-yl)acetamide **18** (27.7 mg, 0.160 mmol) was dissolved in acetonitrile (1.0 mL). Triphenylphosphine gold(I) trifluoromethanesulfonate (3.89 mg, 6.40 µmol, 4.0 mol %) was added and the reaction mixture heated at 50 ˚C for 24 h. 2-(Trimethylsilyl)phenyl trifluoromethanesulfonate **11a** (57.3 mg, 0.192 mmol) and cesium fluoride (72.8 mg, 0.479 mmol) were then added and the resulting mixture heated at 50 ˚C for 16 h. The reaction mixture was filtered through a plug of silica, the solvent removed in vacuo and resulting residue redissolved in a mixture of acetonitrile (1.8 mL) and dichloromethane (0.2 mL). Sodium iodide (72.0 mg, 0.480 mmol) and chlorotrimethylsilane (61 µL, 0.480 mmol) were added and the reaction was stirred for 30 min at room temperature. The solvent was removed *in vacuo* and the product was purified using flash column chromatography (*n*-hexane:dichloromethane) to afford the title compound (34.4 mg, 0.0960 mmol, 60%) as a pale yellow solid; R_f 0.53 (2:1 Petrol 40-60:ethyl acetate).

^{1}H NMR (400 MHz, $CDCl_3$): δ_H 8.29 (dd, *J* = 6.9, 3.2, 4H), 7.46 (dd, *J* = 6.9, 3.2, 4H), 7.26-7.21 (m, 4H), 7.20-7.15 (m, 6H), 5.07 (s, 4H).

^{13}C NMR (101 MHz, $CDCl_3$): δ_C 141.1, 131.6, 130.7, 128.7, 128.3, 126.1, 125.7, 125.6, 34.0.

Data is consistent with previous reported literature.[4]

[4] E. Yagodkin, C. J. Douglas, *Tetrahedron Lett.* **2010**, *51*, 3037.

3. ^{1}H and ^{13}C NMR spectra of isolated compounds

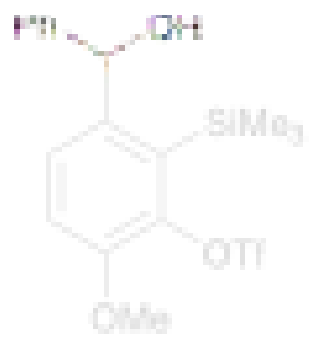

^{1}H NMR (400 MHz, $CDCl_3$), **11m**

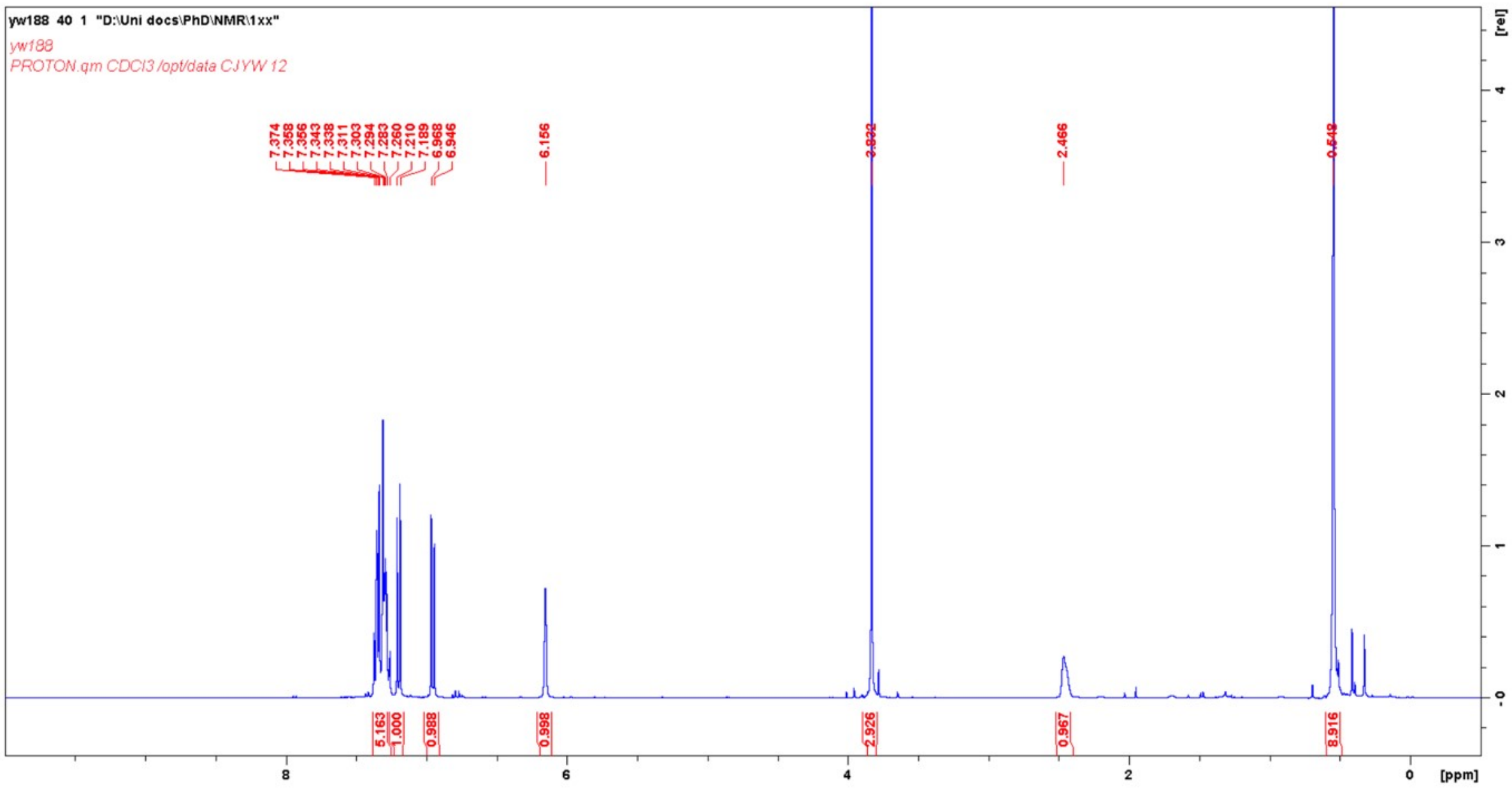

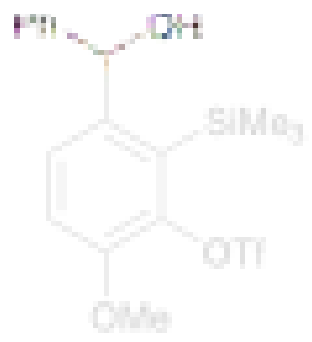

^{13}C NMR (101 MHz, $CDCl_3$), **11m**

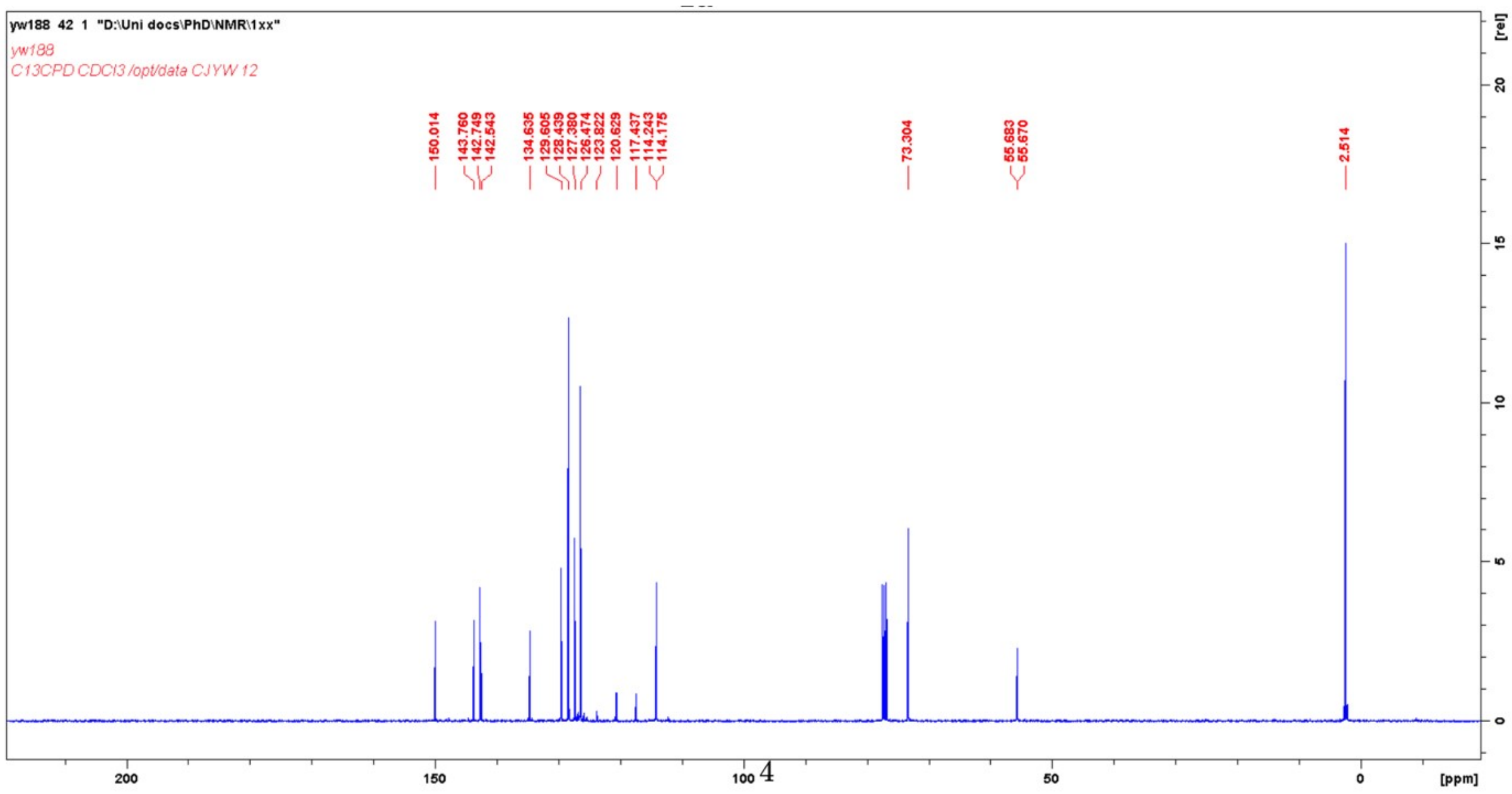

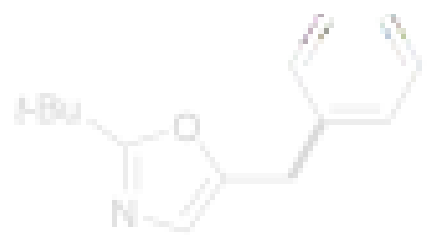

^1H NMR (400 MHz, $CDCl_3$), **15a**

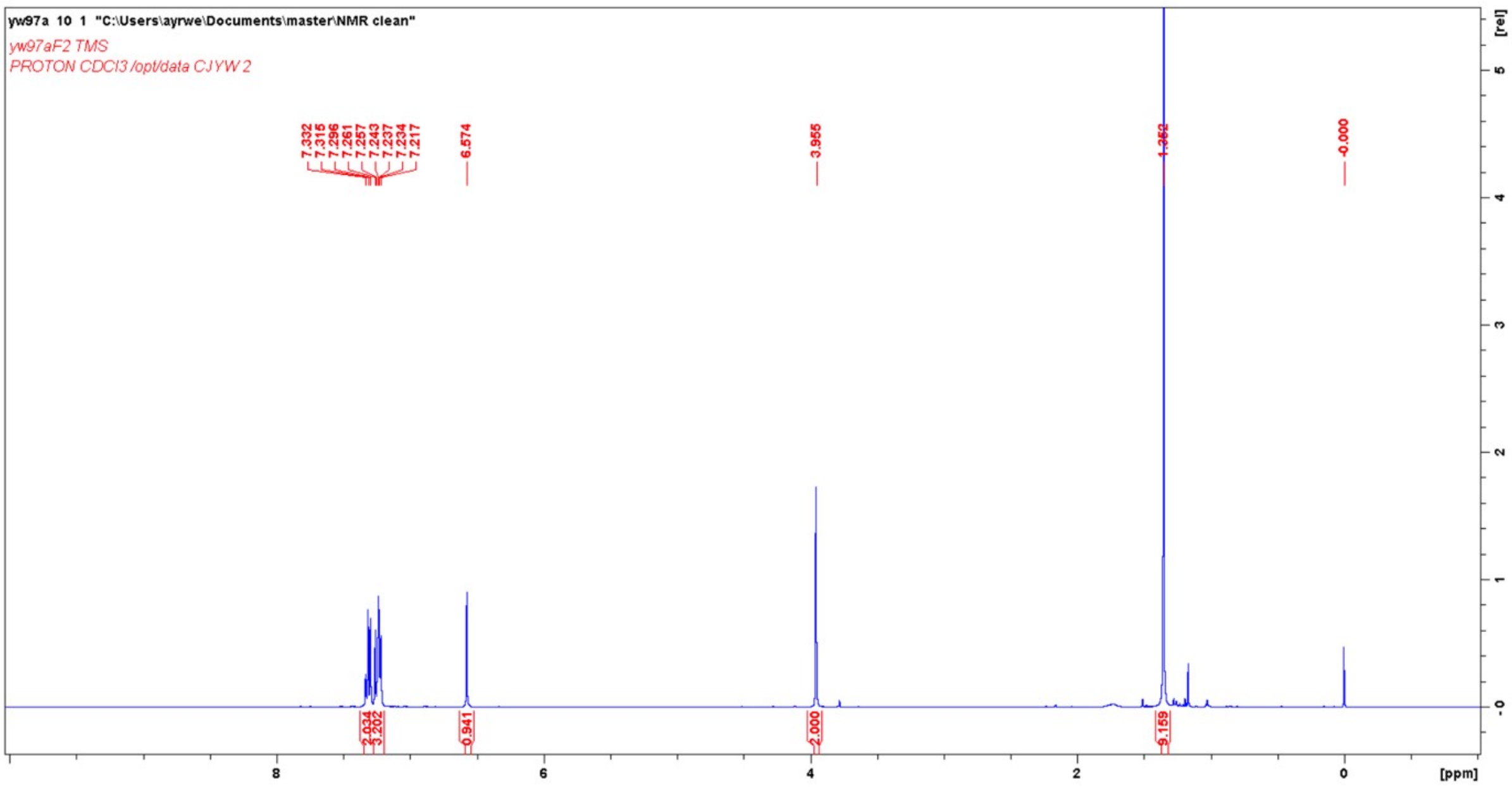

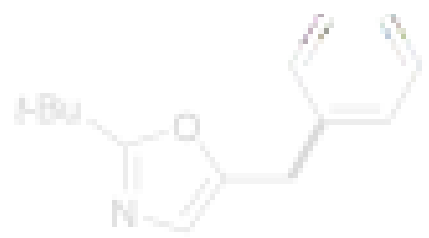

^{13}C NMR (101 MHz, $CDCl_3$), **15a**

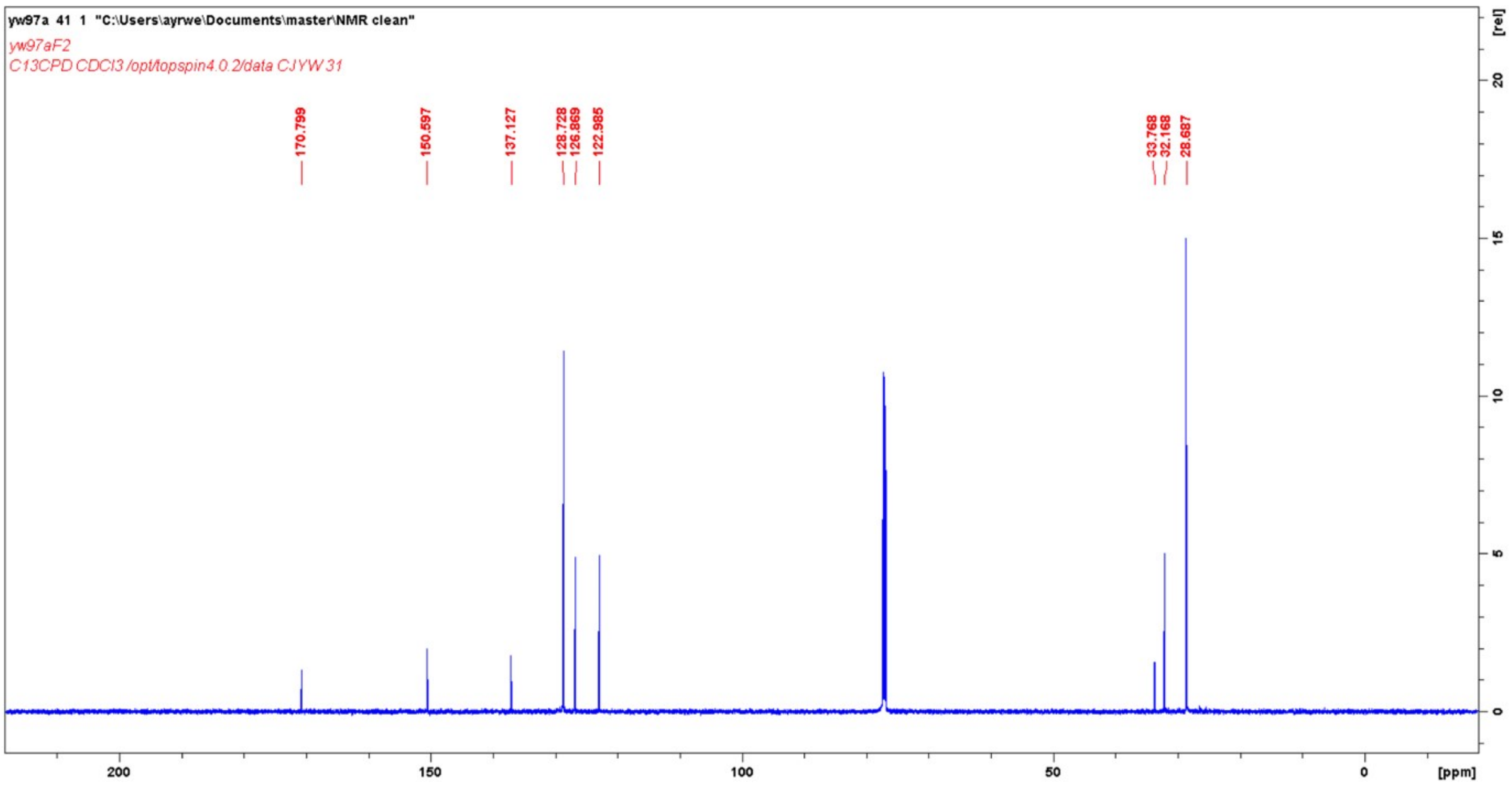

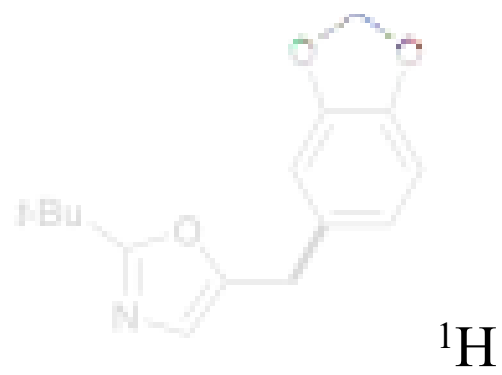

^{1}H NMR (400 MHz, $CDCl_3$), **15b**

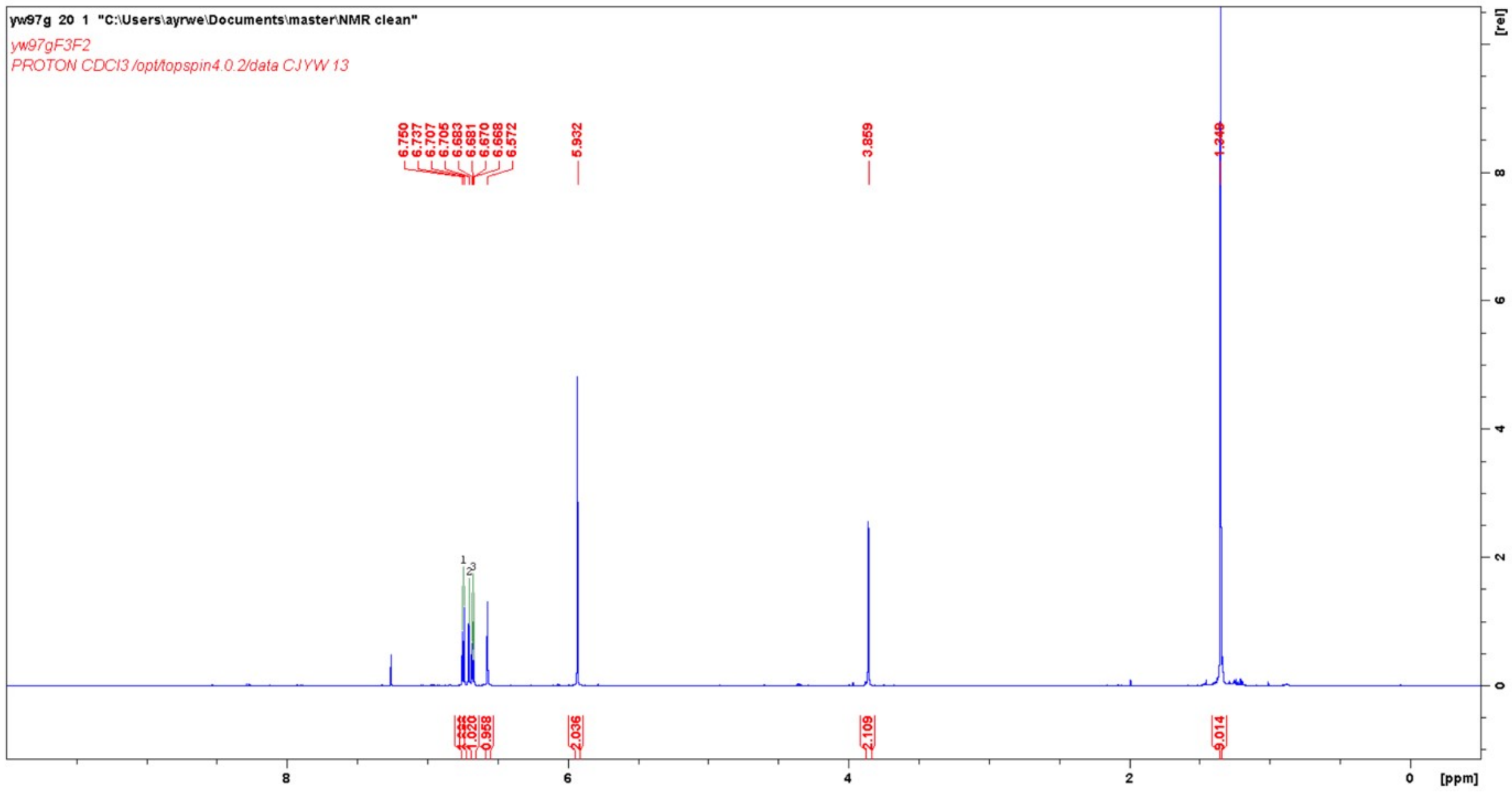

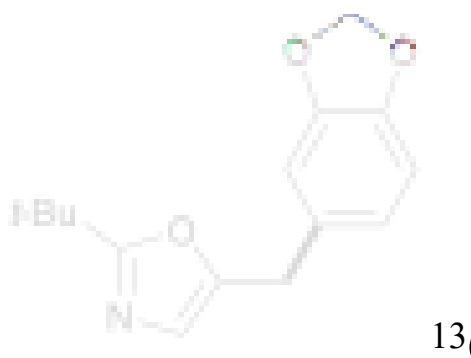

^{13}C NMR (101 MHz, $CDCl_3$), **15b**

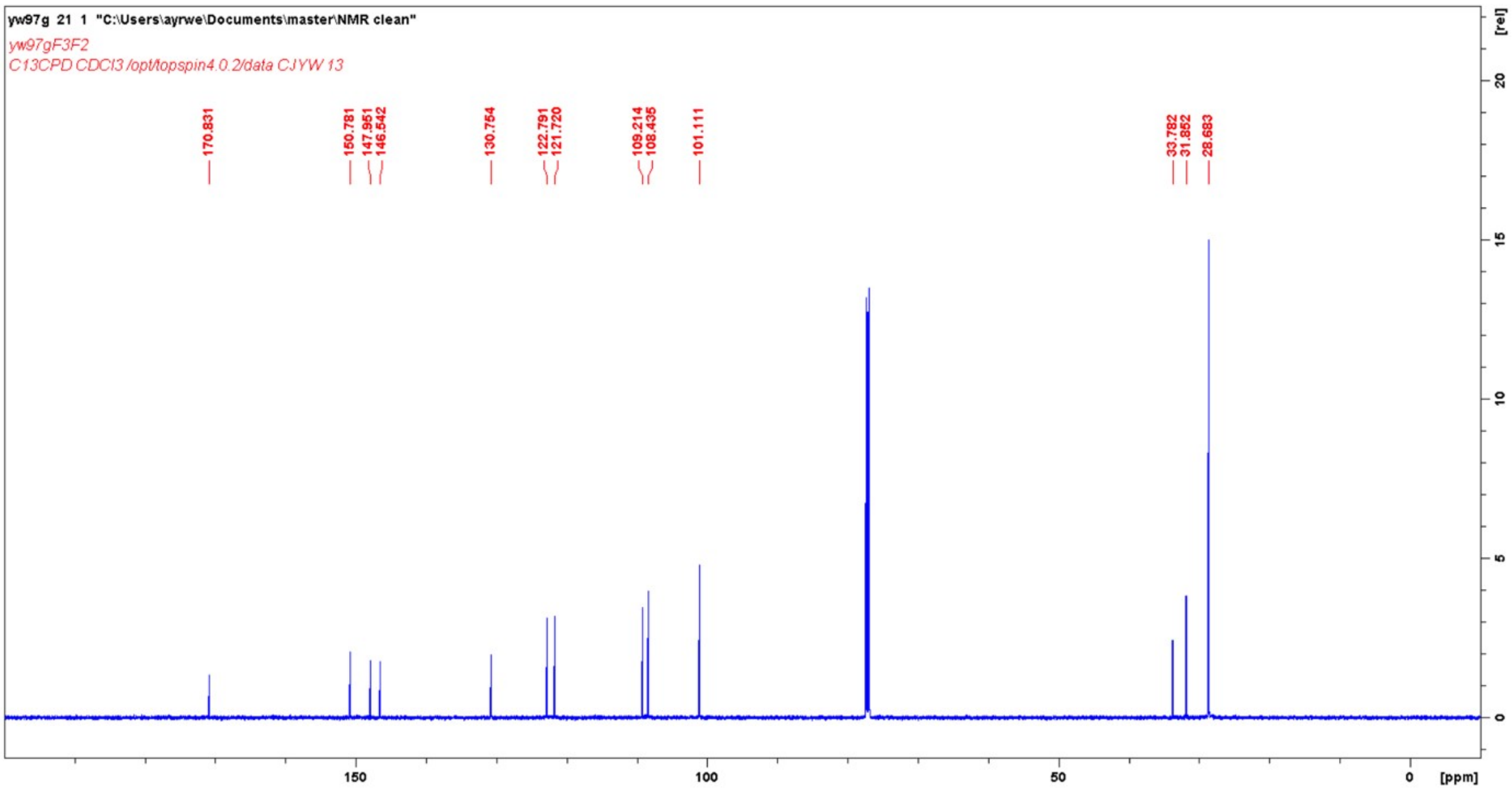

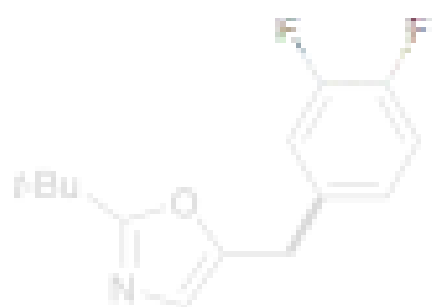

^{1}H NMR (400 MHz, $CDCl_3$), **15c**

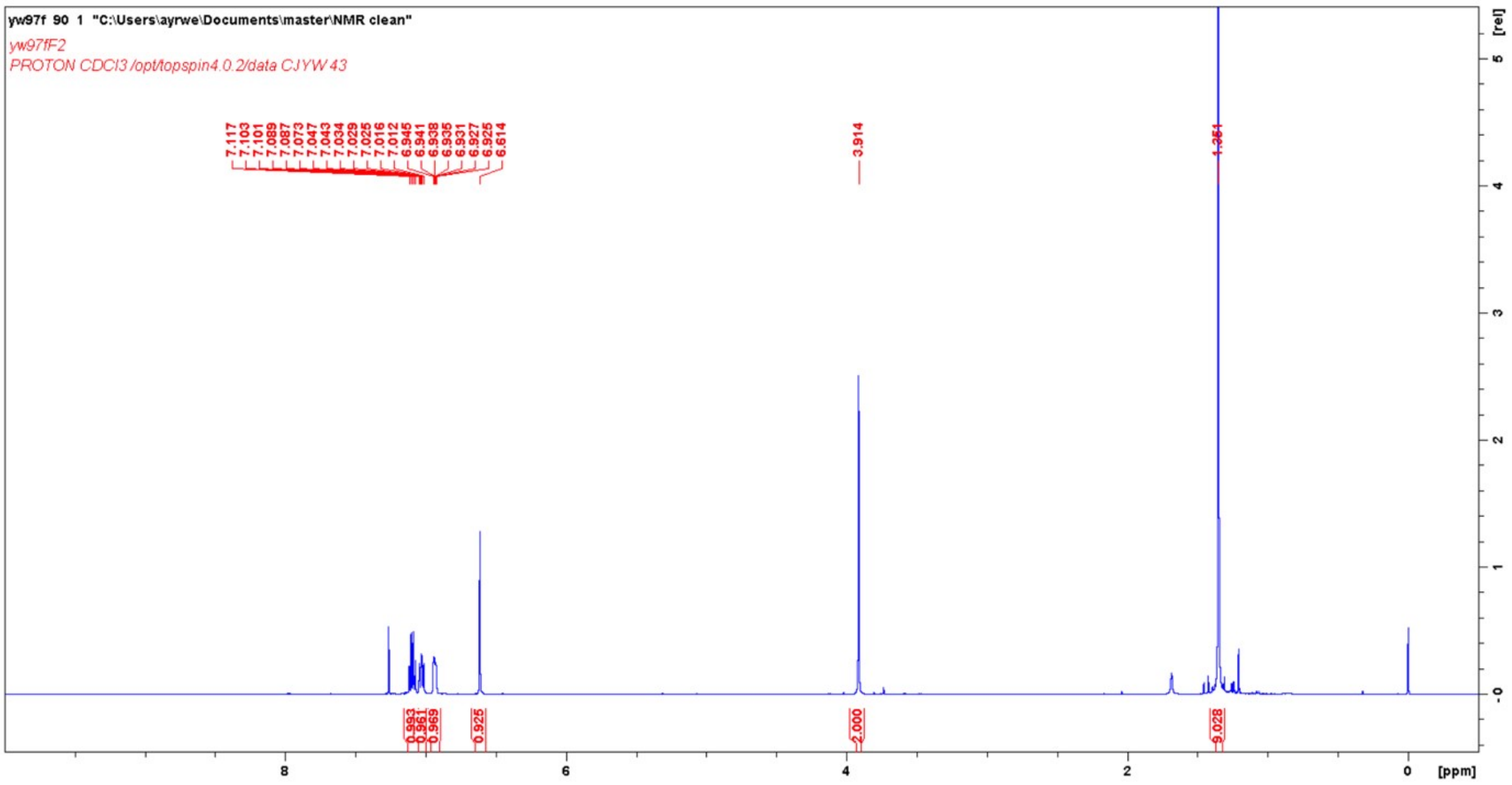

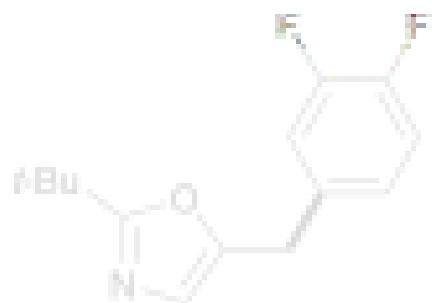

^{13}C NMR (101 MHz, $CDCl_3$), **15c**

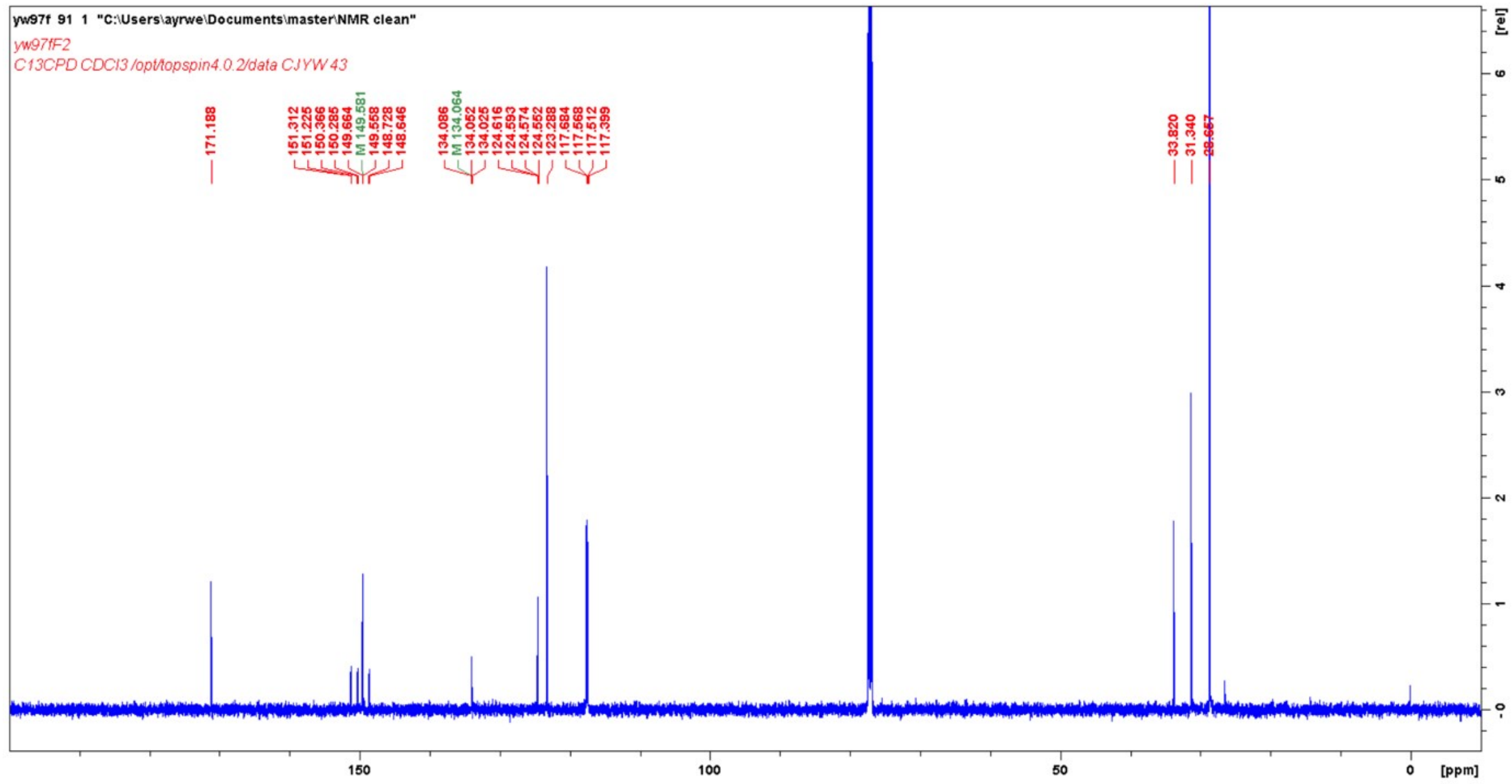

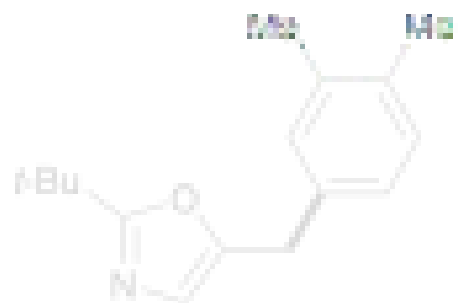

^{1}H NMR (400 MHz, $CDCl_3$), **15d**

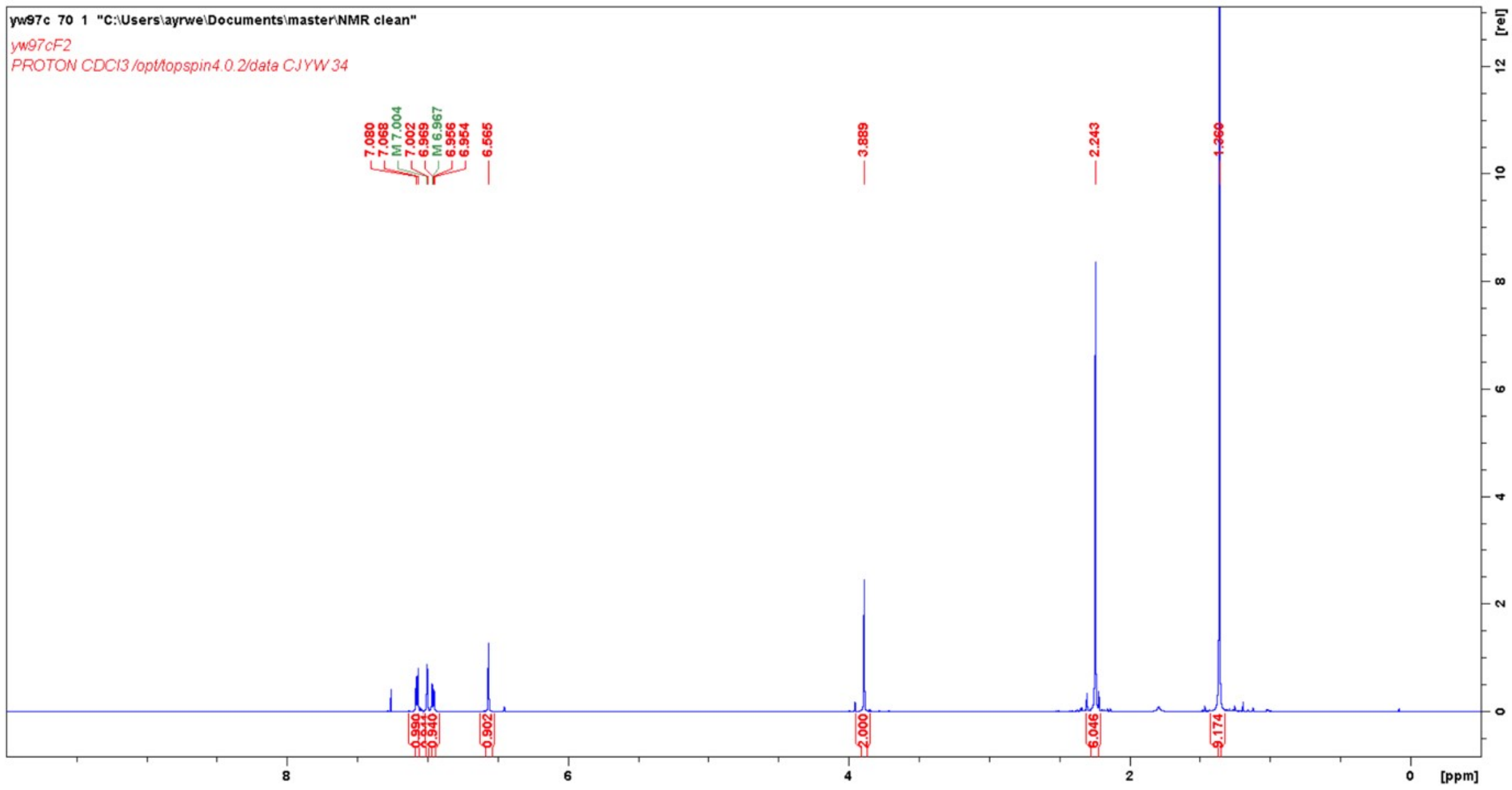

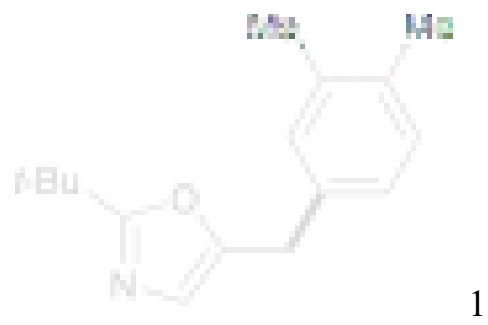

^{13}C NMR (101 MHz, $CDCl_3$), **15d**

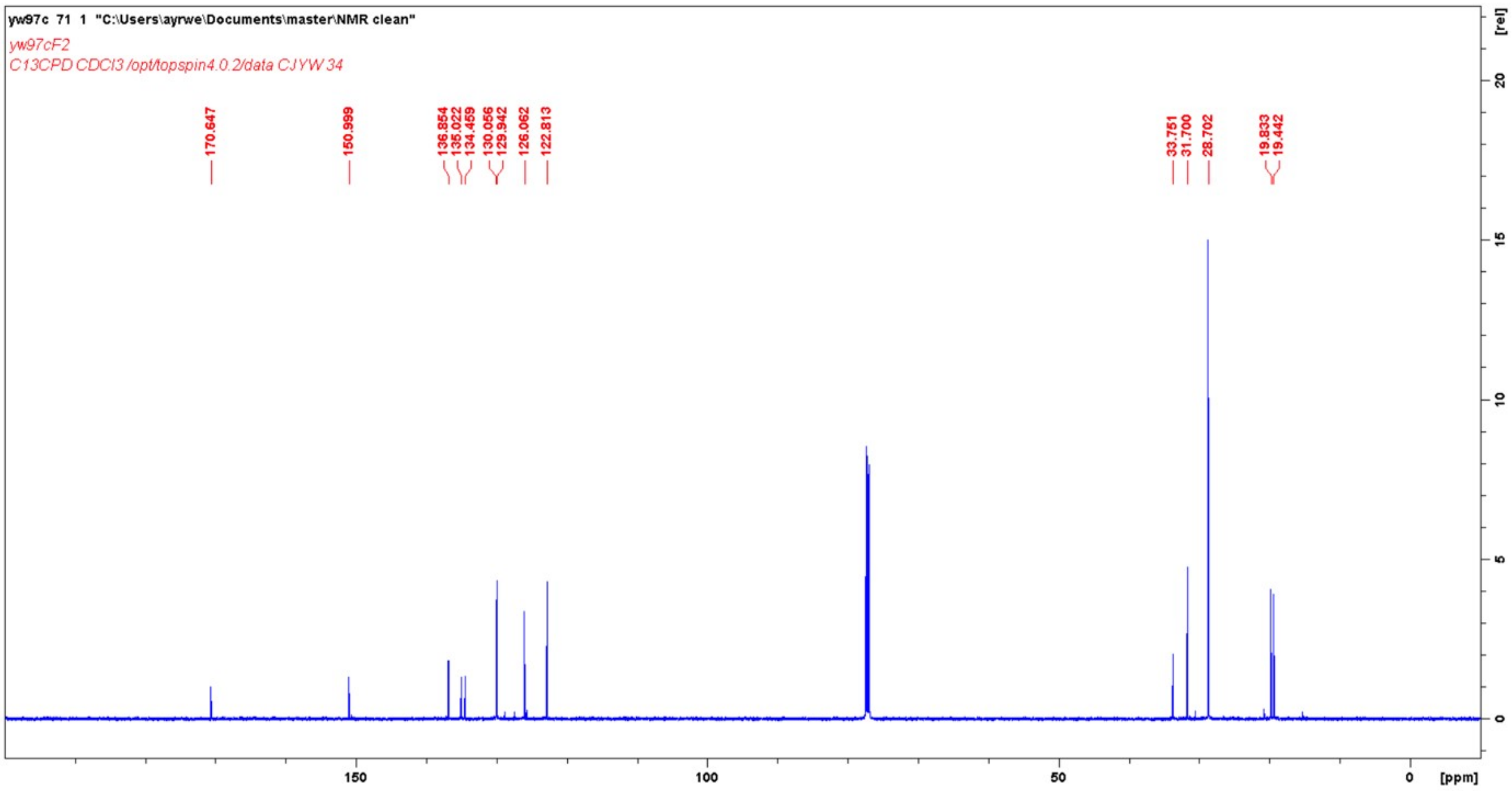

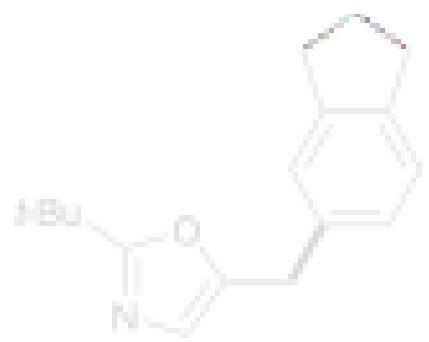

^{1}H NMR (400 MHz, $CDCl_3$), **15e**

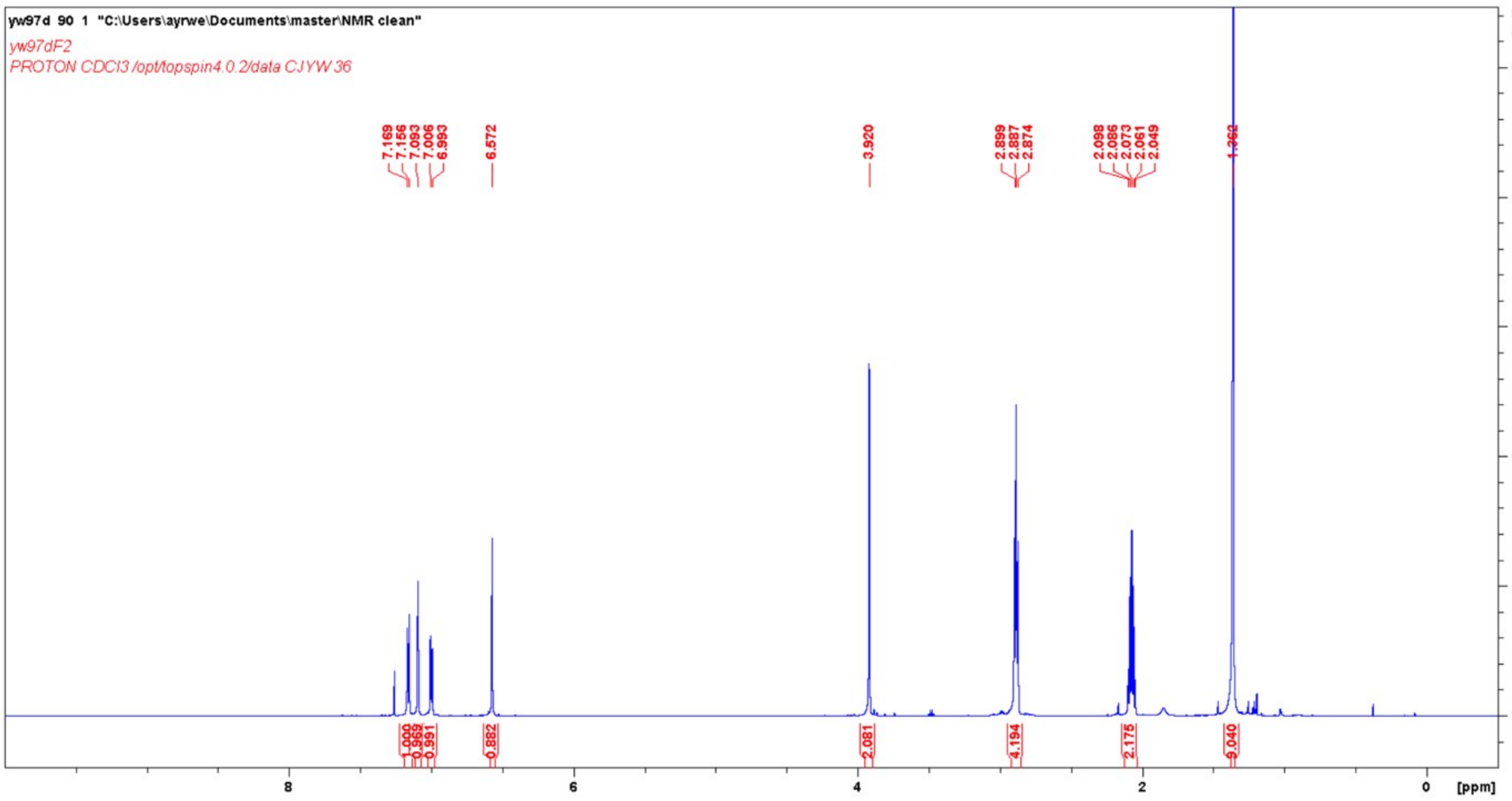

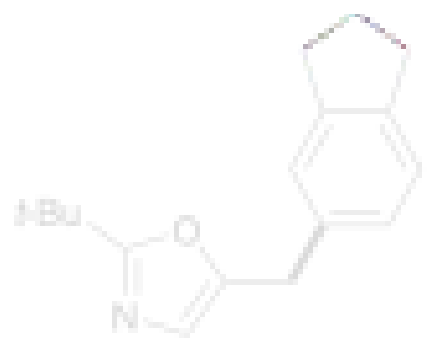

^{13}C NMR (101 MHz, $CDCl_3$), **15e**

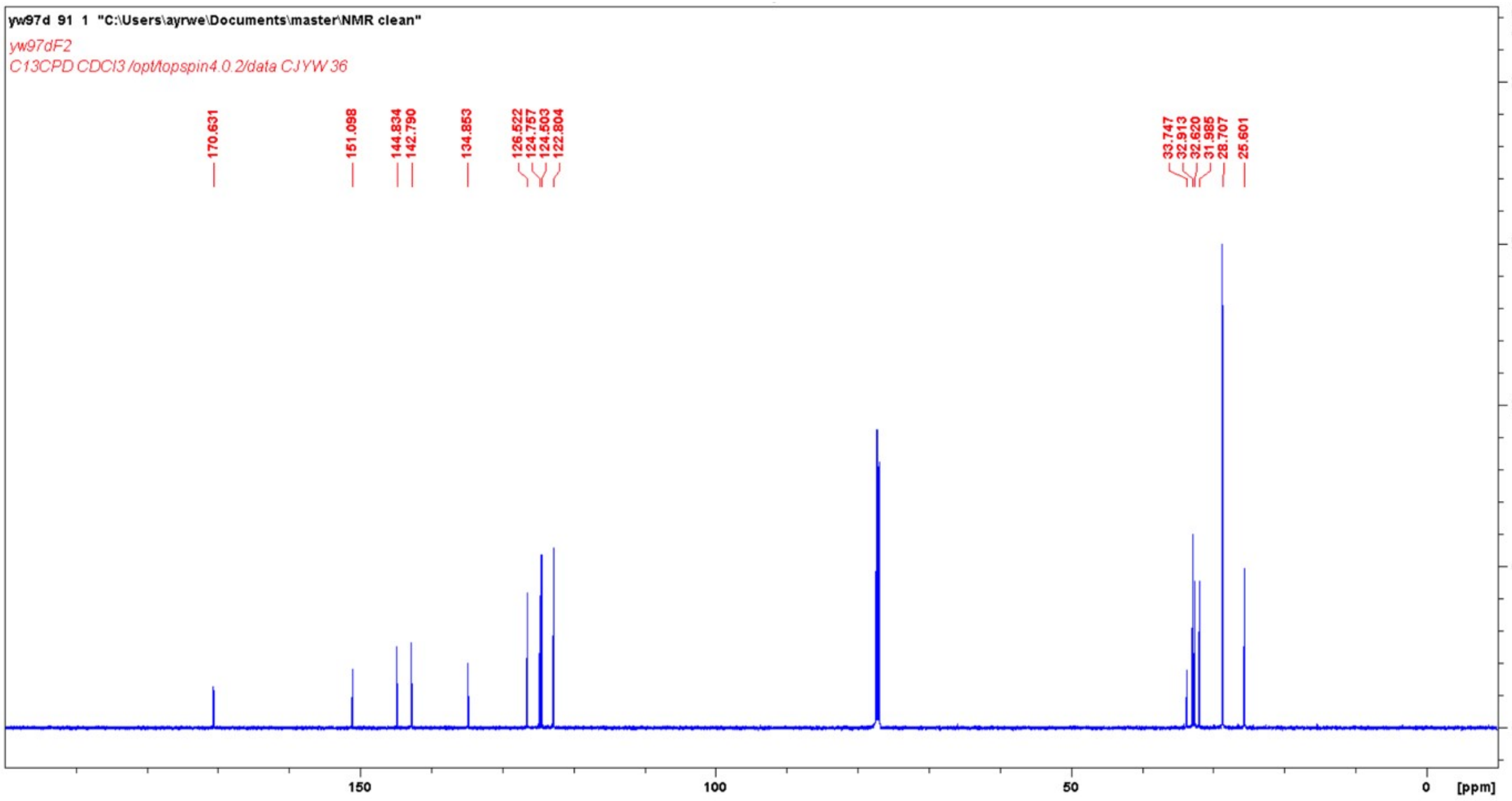

^{1}H NMR (400 MHz, $CDCl_3$), **15f**

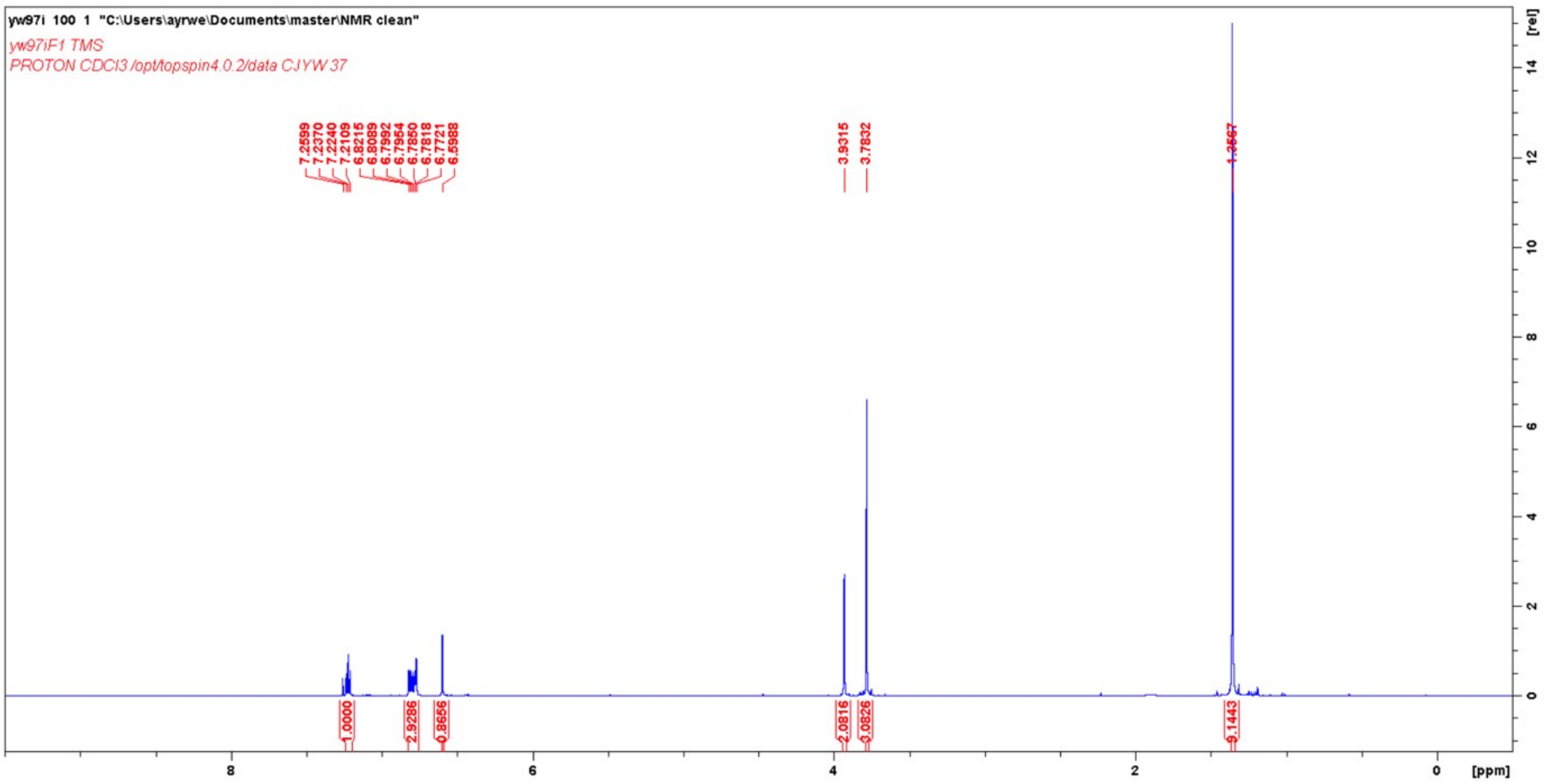

^{13}C NMR (101 MHz, $CDCl_3$), **15f**

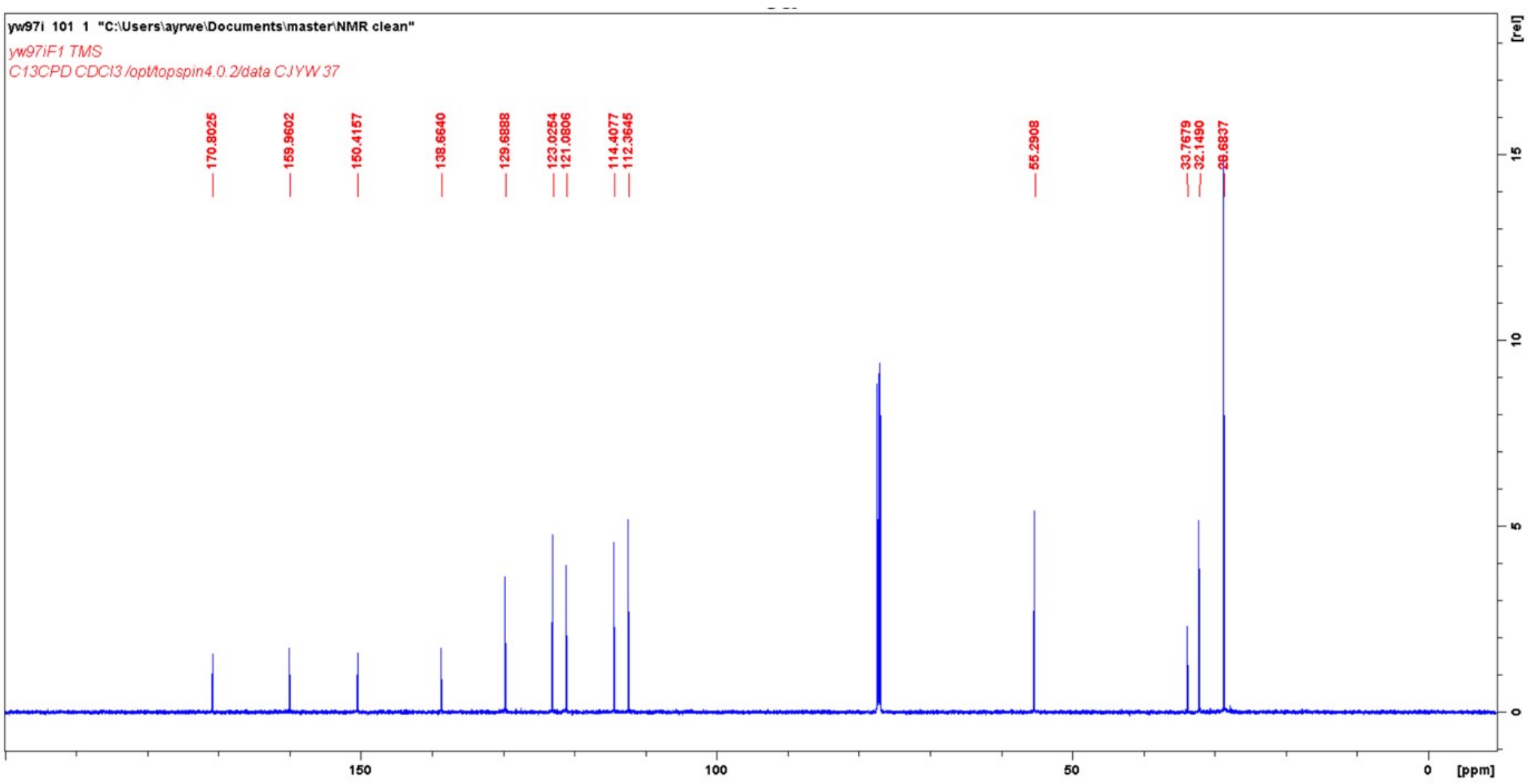

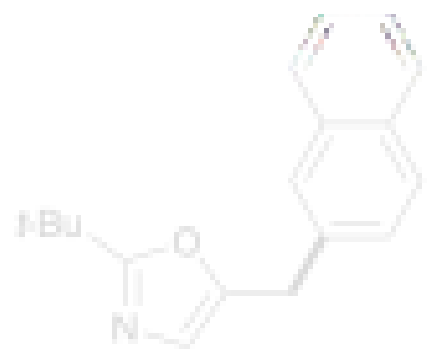

^1H NMR (400 MHz, $CDCl_3$), **15g**

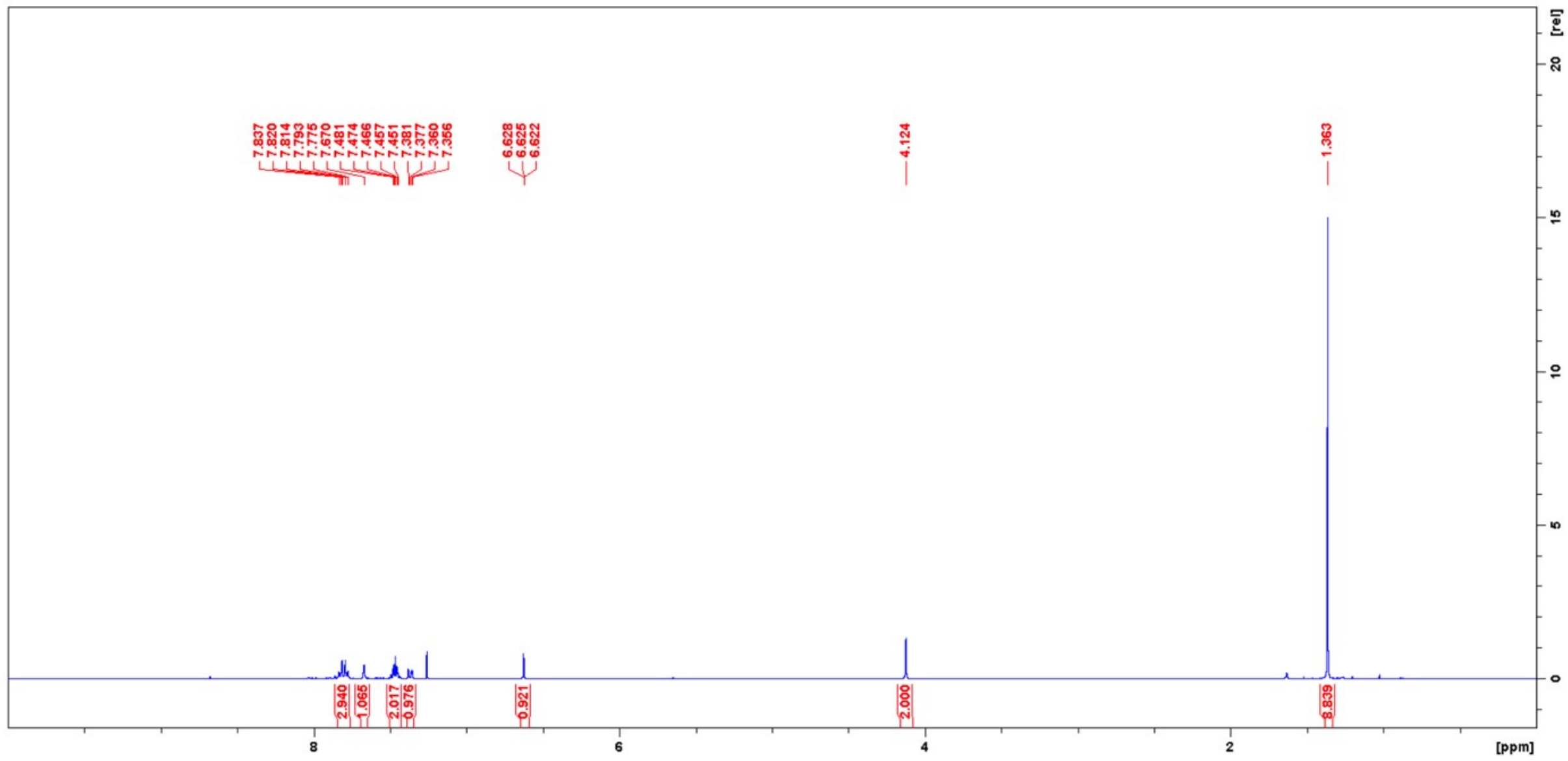

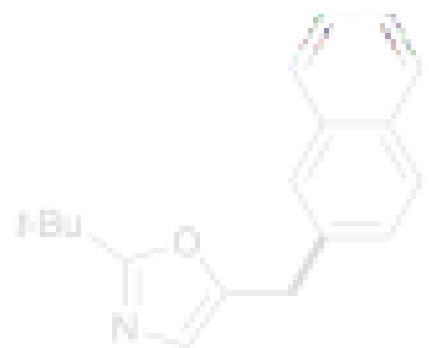

^{13}C NMR (101 MHz, $CDCl_3$), **15g**

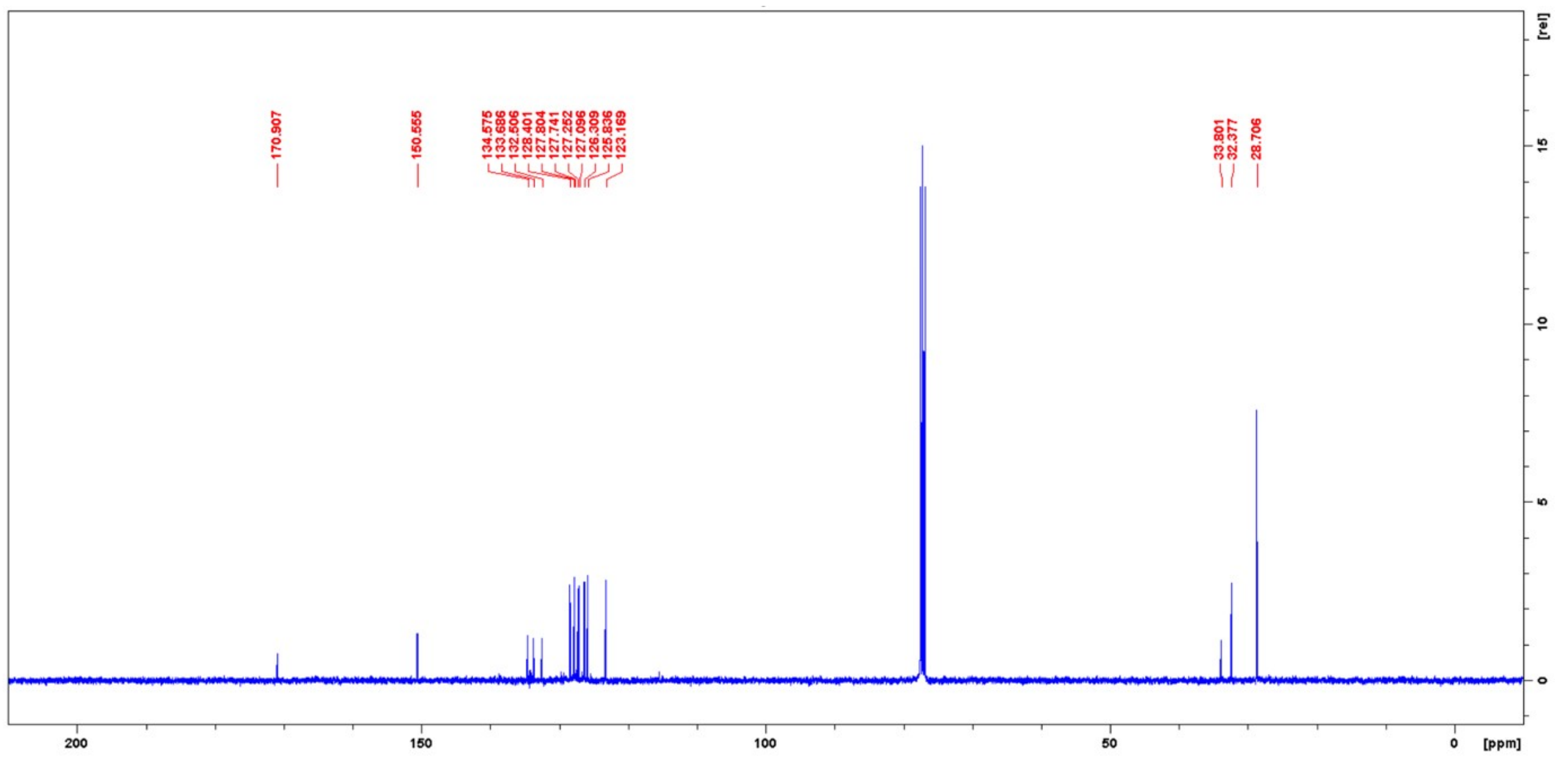

^{1}H NMR (400 MHz, $CDCl_3$), **15h & 15h'**

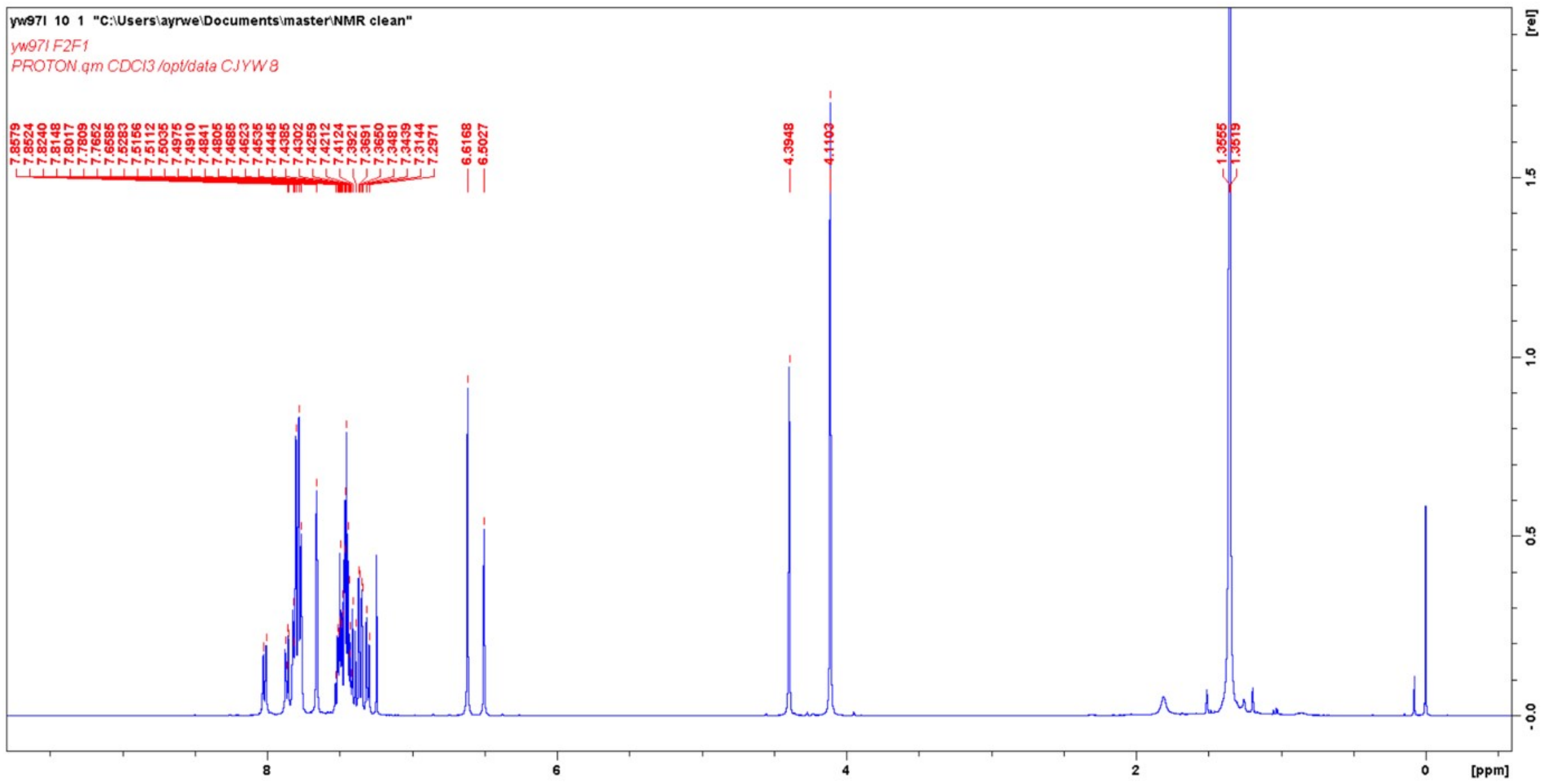

^{13}C NMR (101 MHz, $CDCl_3$), **15h & 15h'**

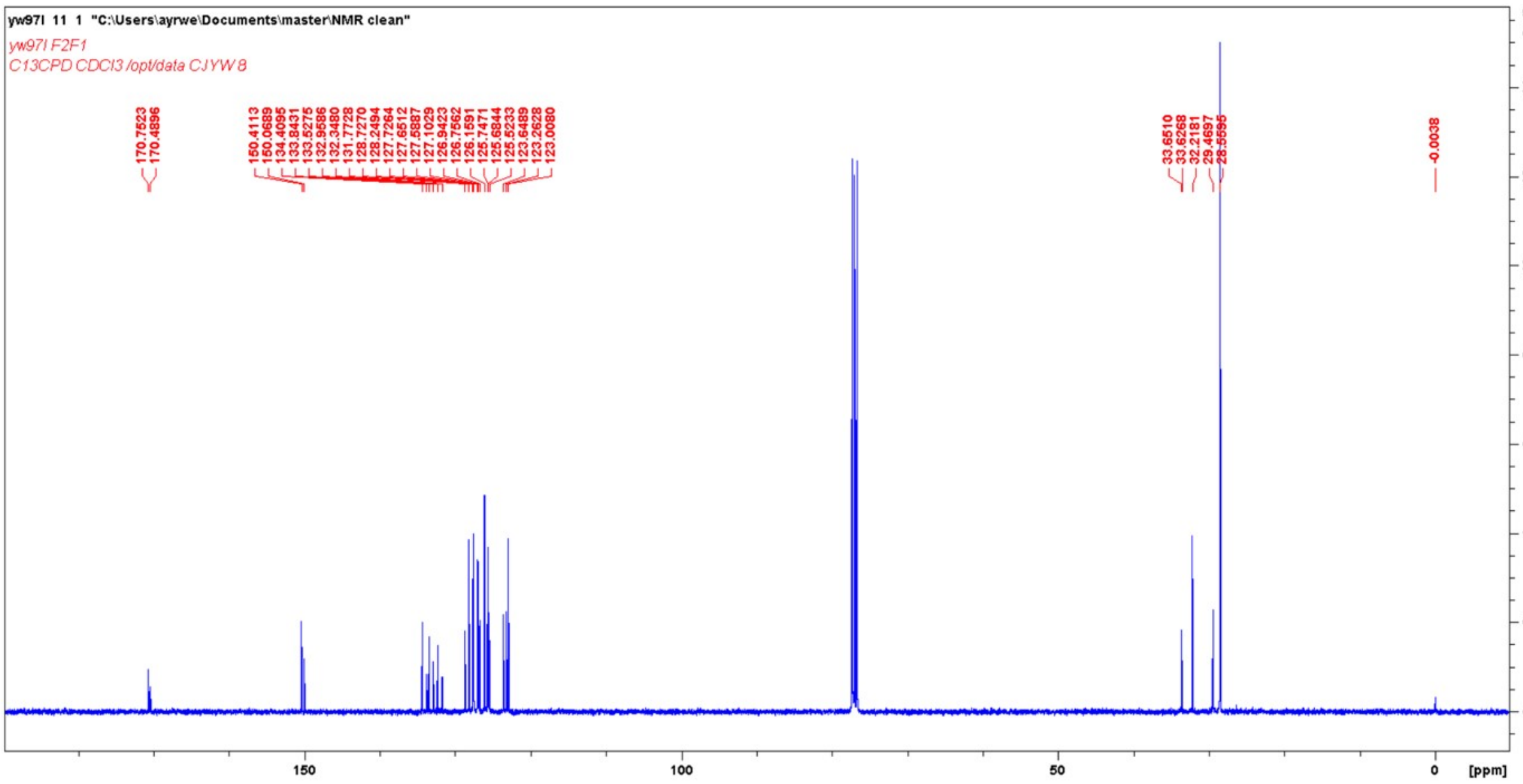

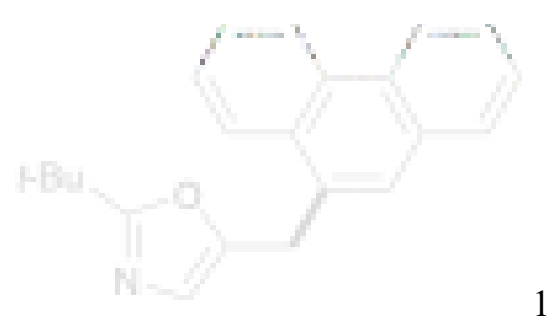

^{1}H NMR (400 MHz, $CDCl_3$), **15i**

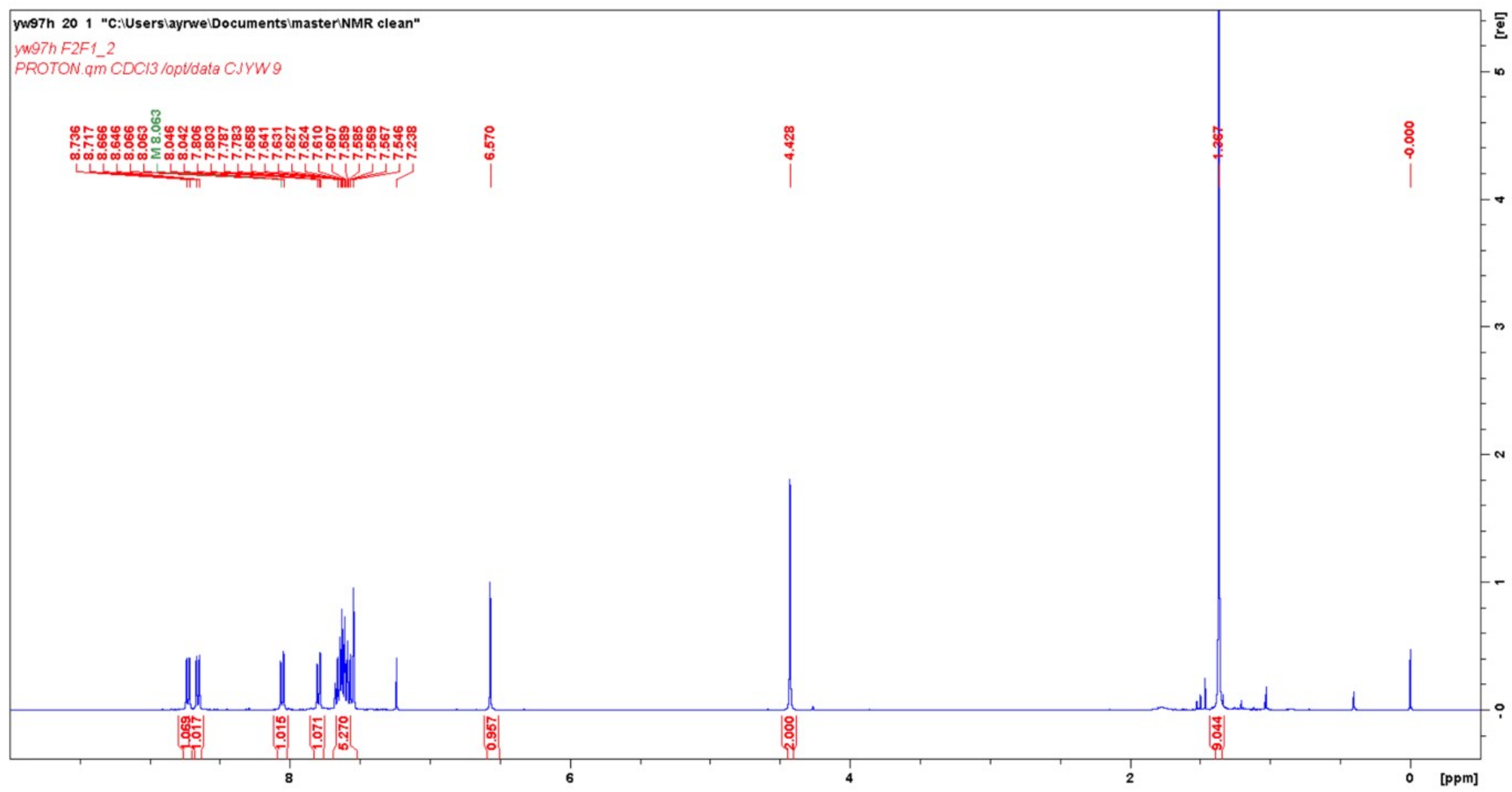

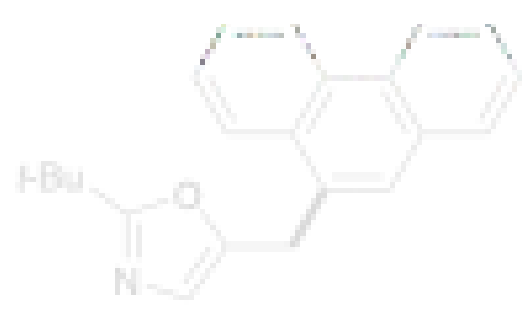

^{13}C NMR (101 MHz, $CDCl_3$), **15i**

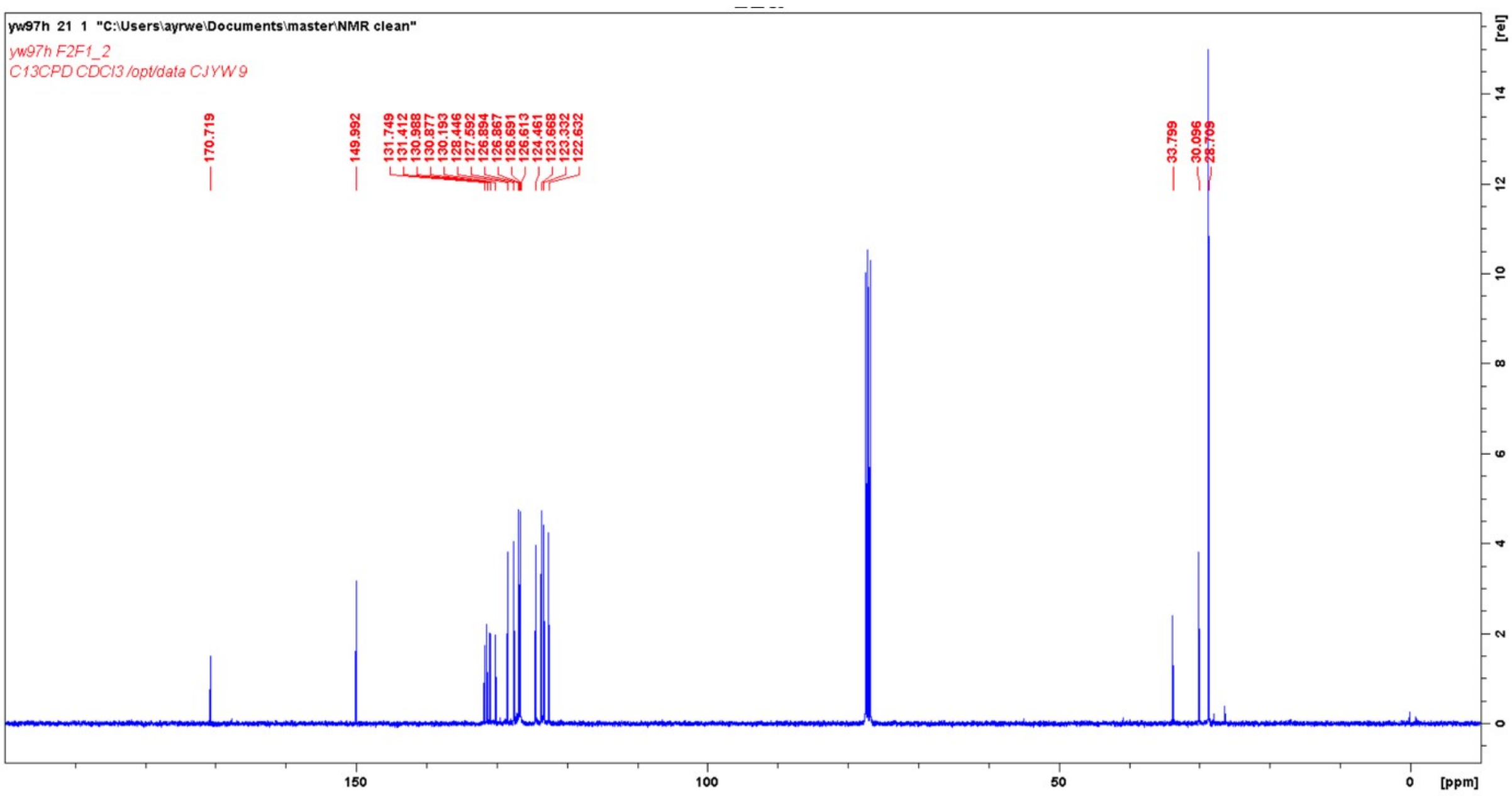

^{1}H NMR (400 MHz, $CDCl_3$), **15j & 15j'**

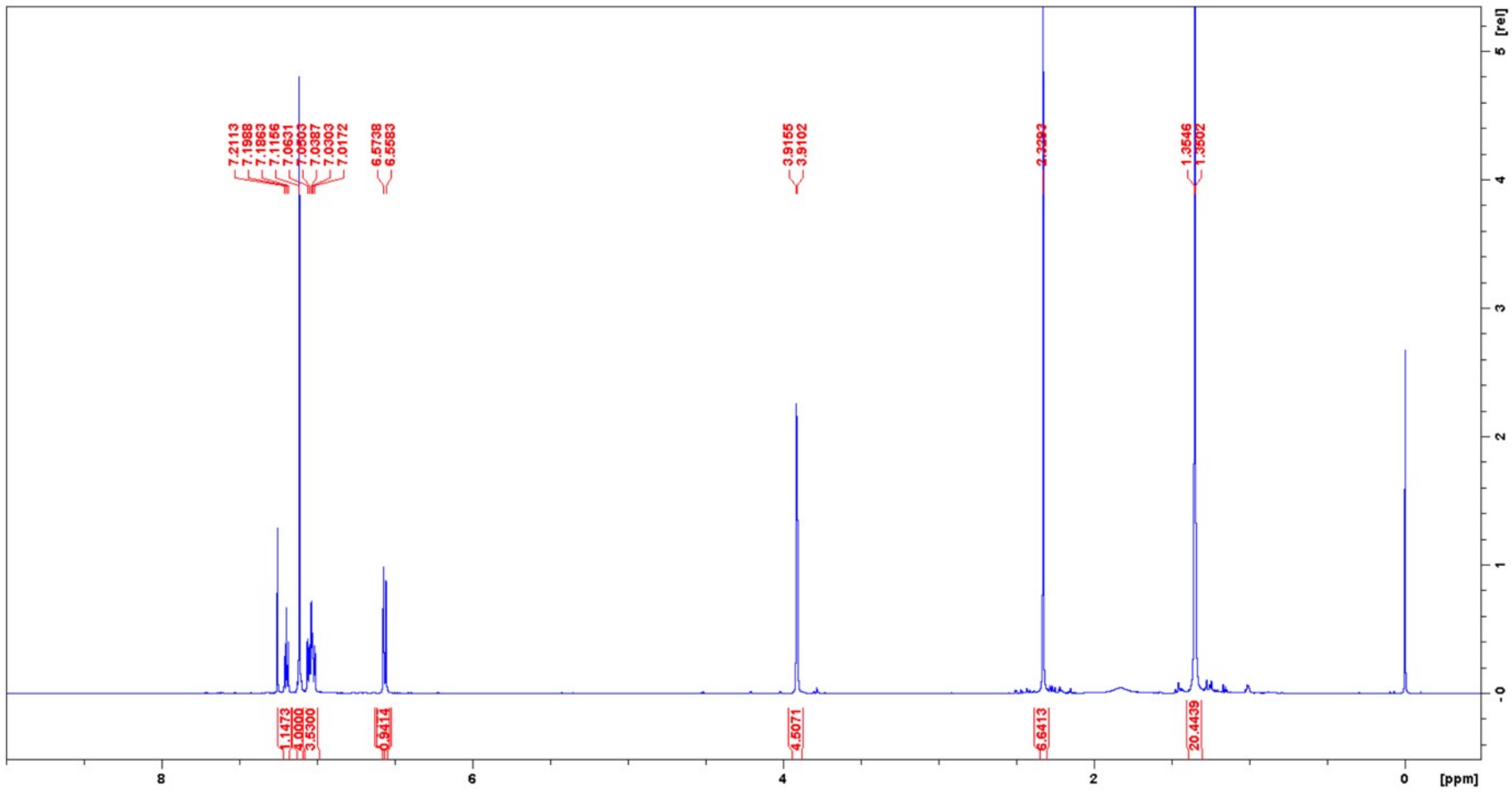

^{13}C NMR (101 MHz, $CDCl_3$), **15j & 15j'**

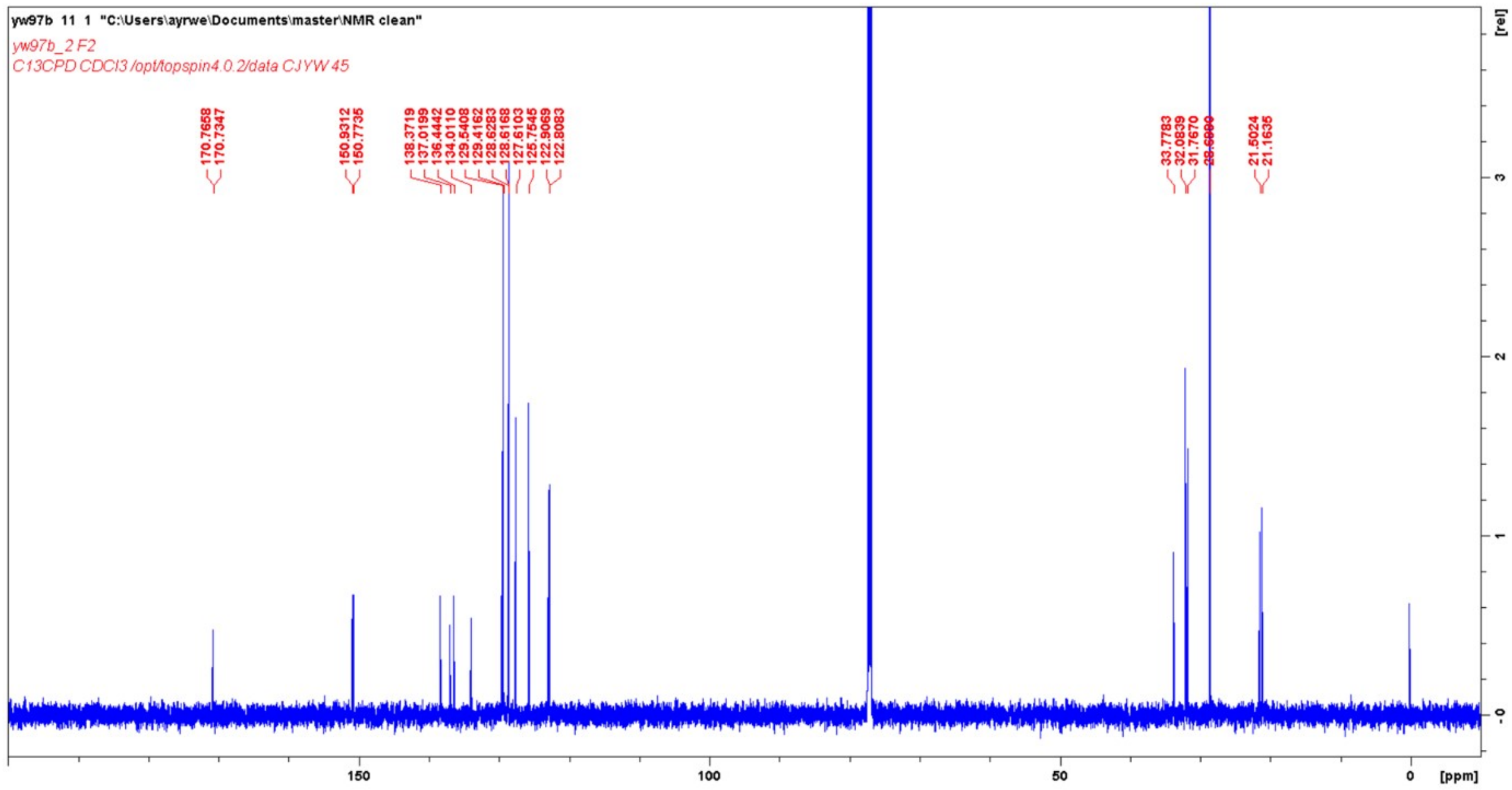

^{1}H NMR (400 MHz, $CDCl_3$), **15k & 15k’**

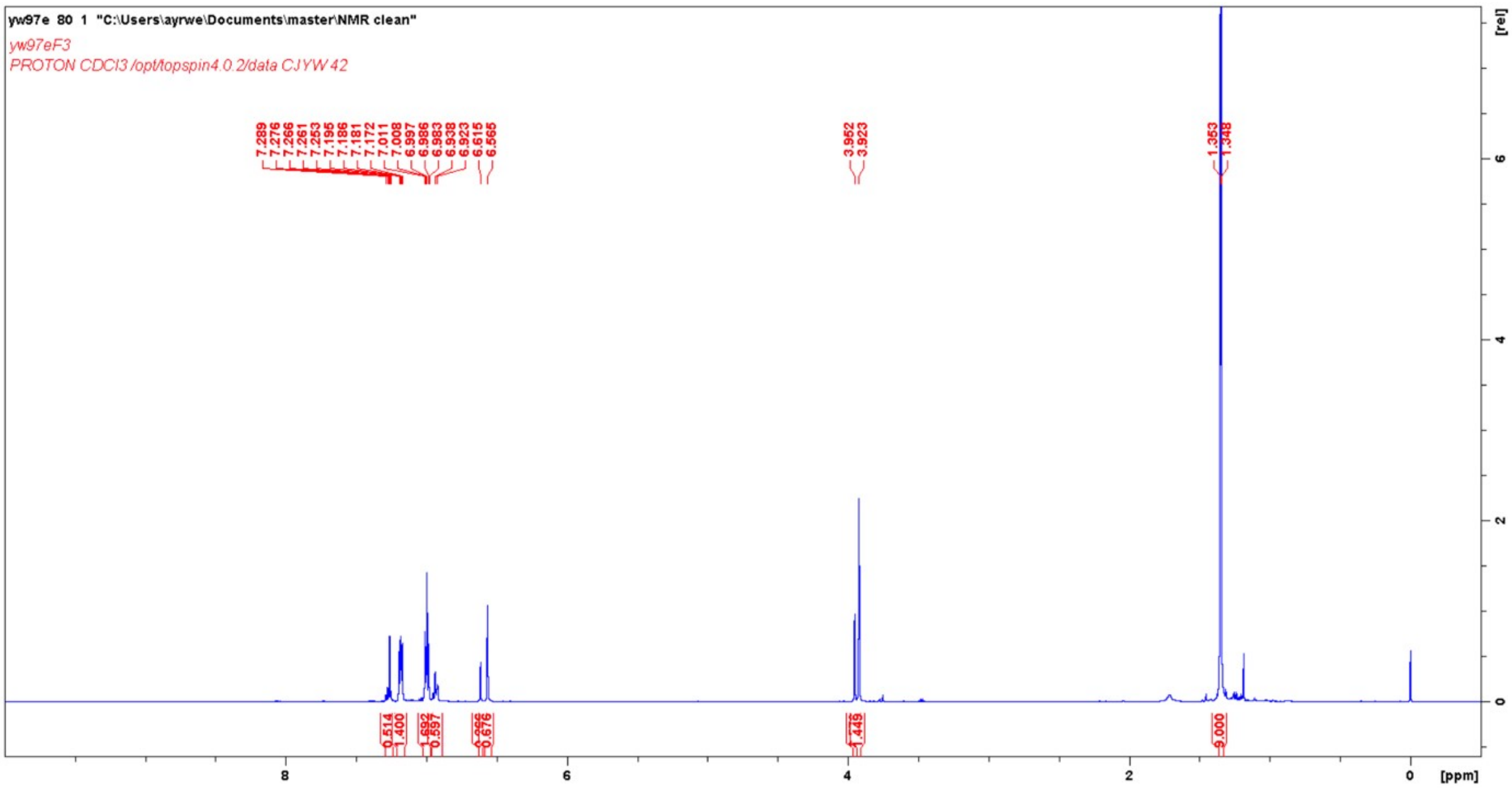

^{13}C NMR (101 MHz, $CDCl_3$), **15k & 15k’**

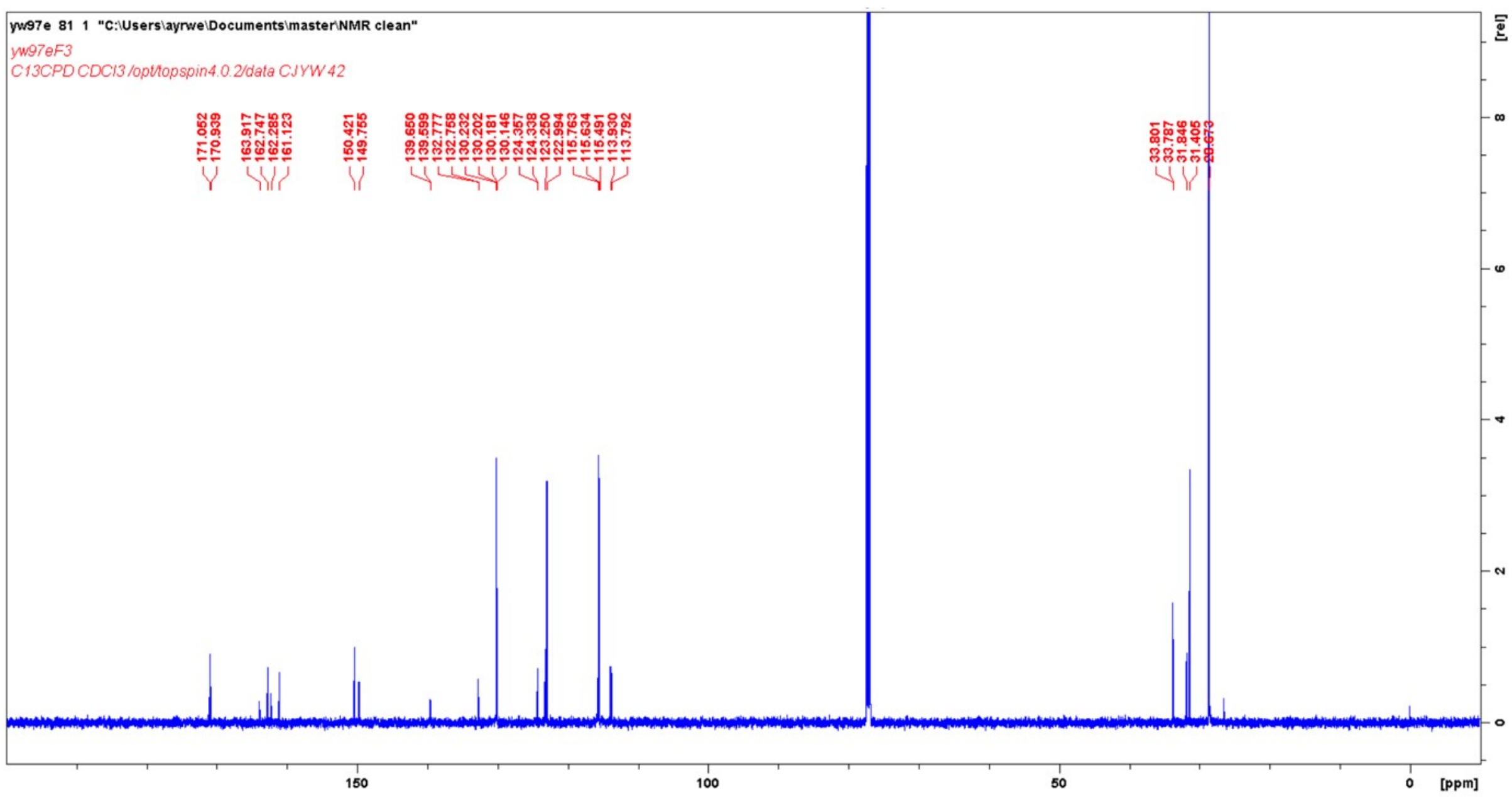

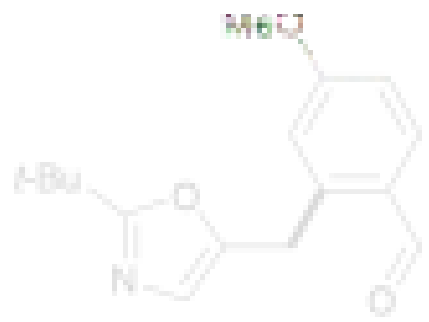

^{1}H NMR (400 MHz, $CDCl_3$), **15l**

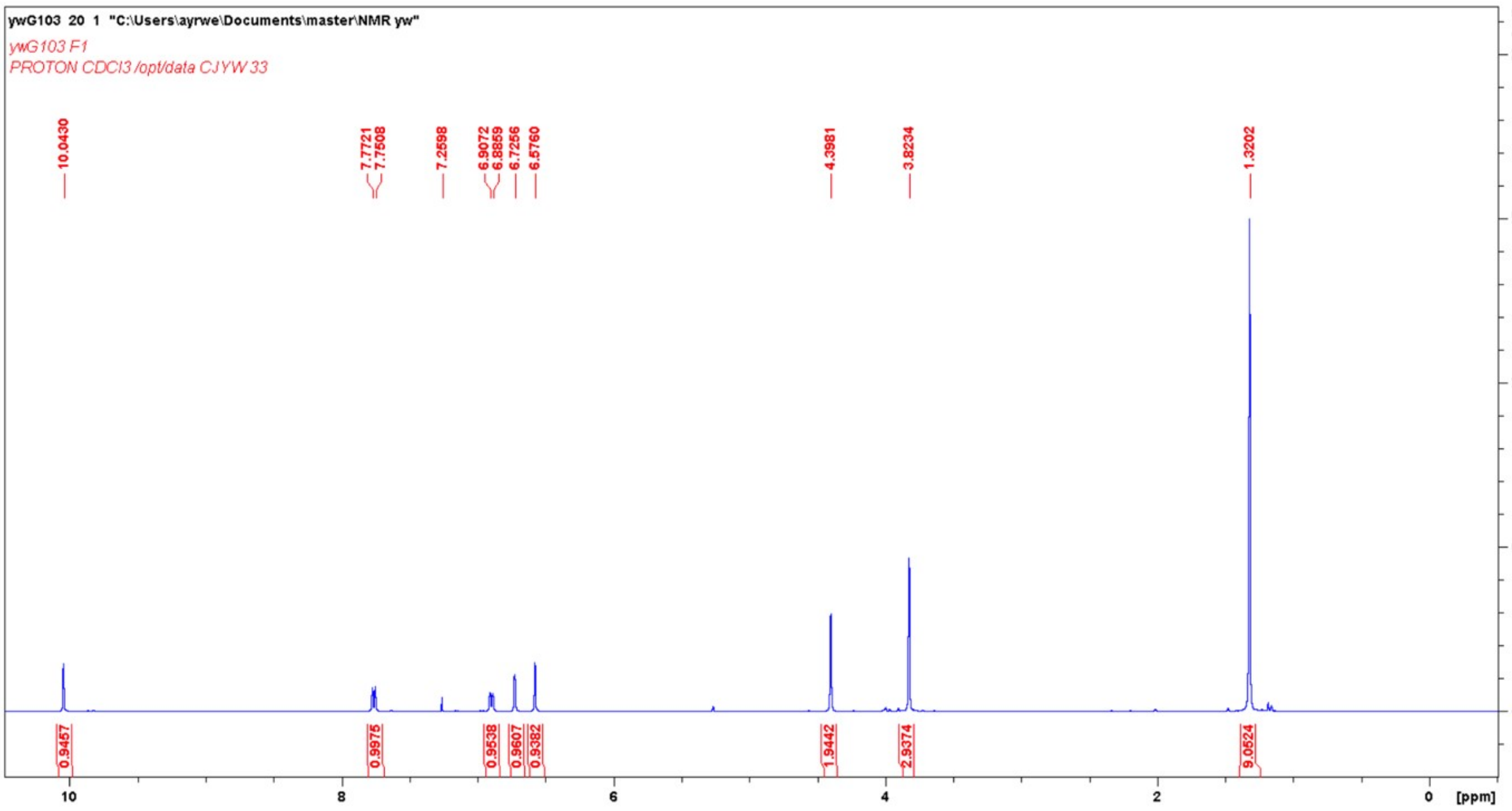

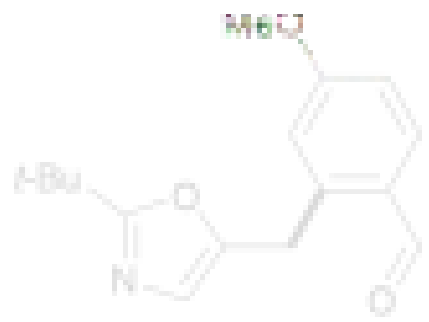

^{13}C NMR (101 MHz, $CDCl_3$), **15l**

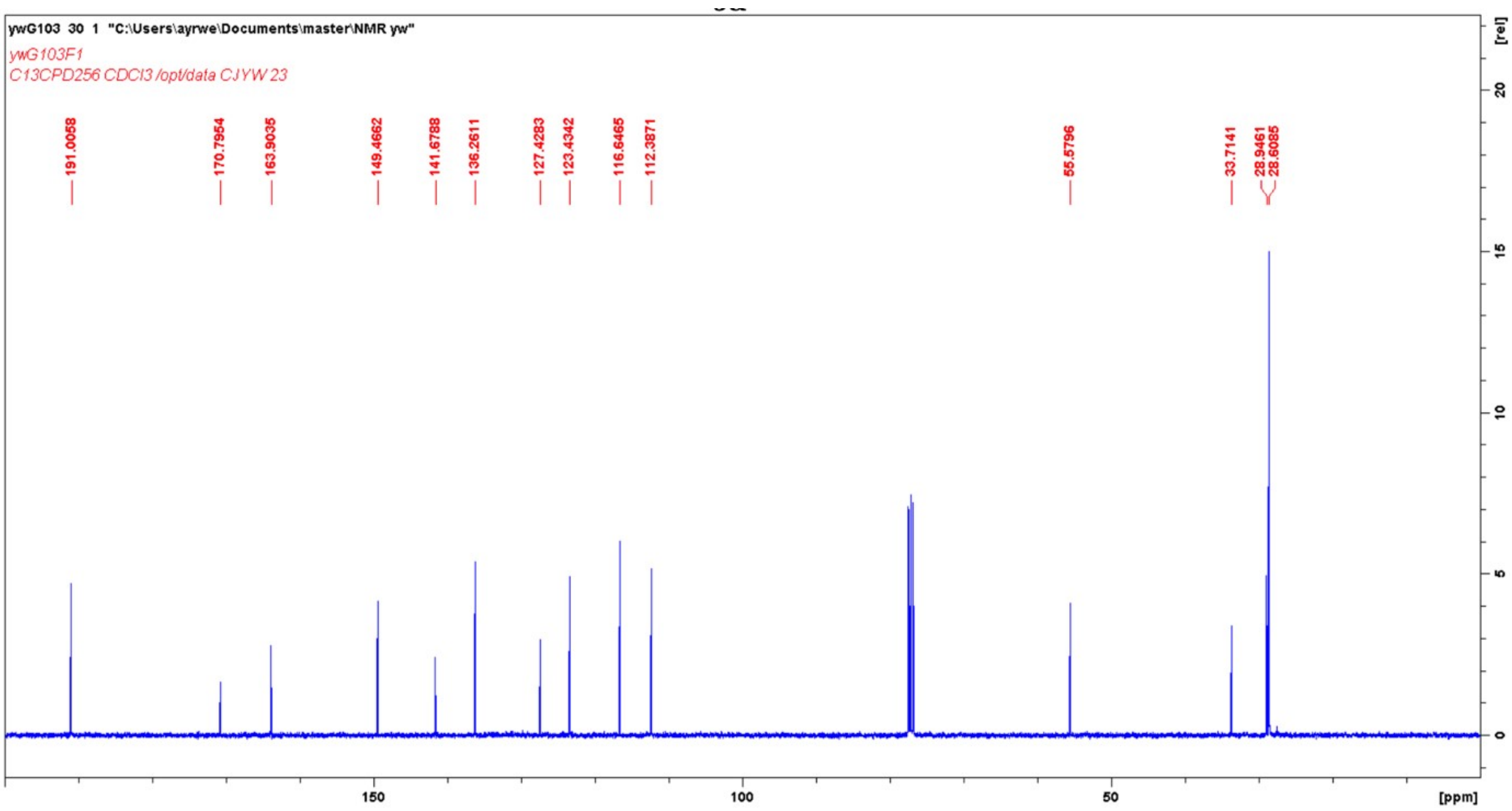

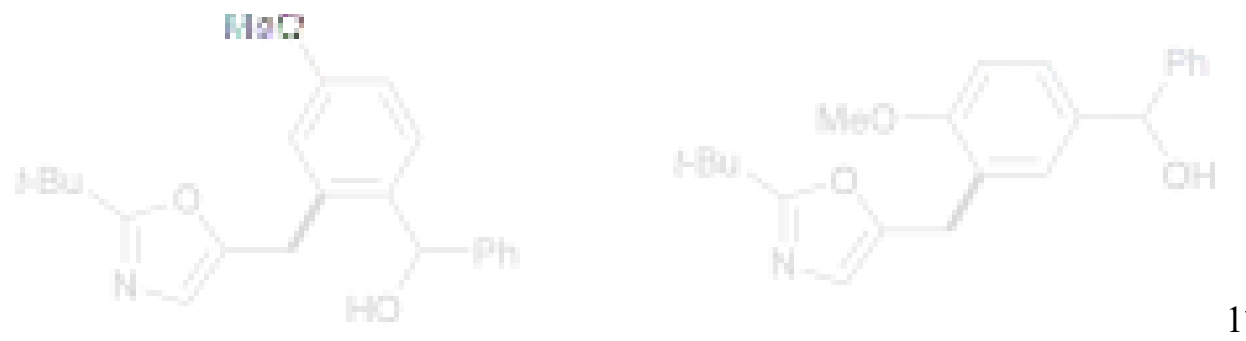

^1H NMR (400 MHz, $CDCl_3$), **15m & 15m'**

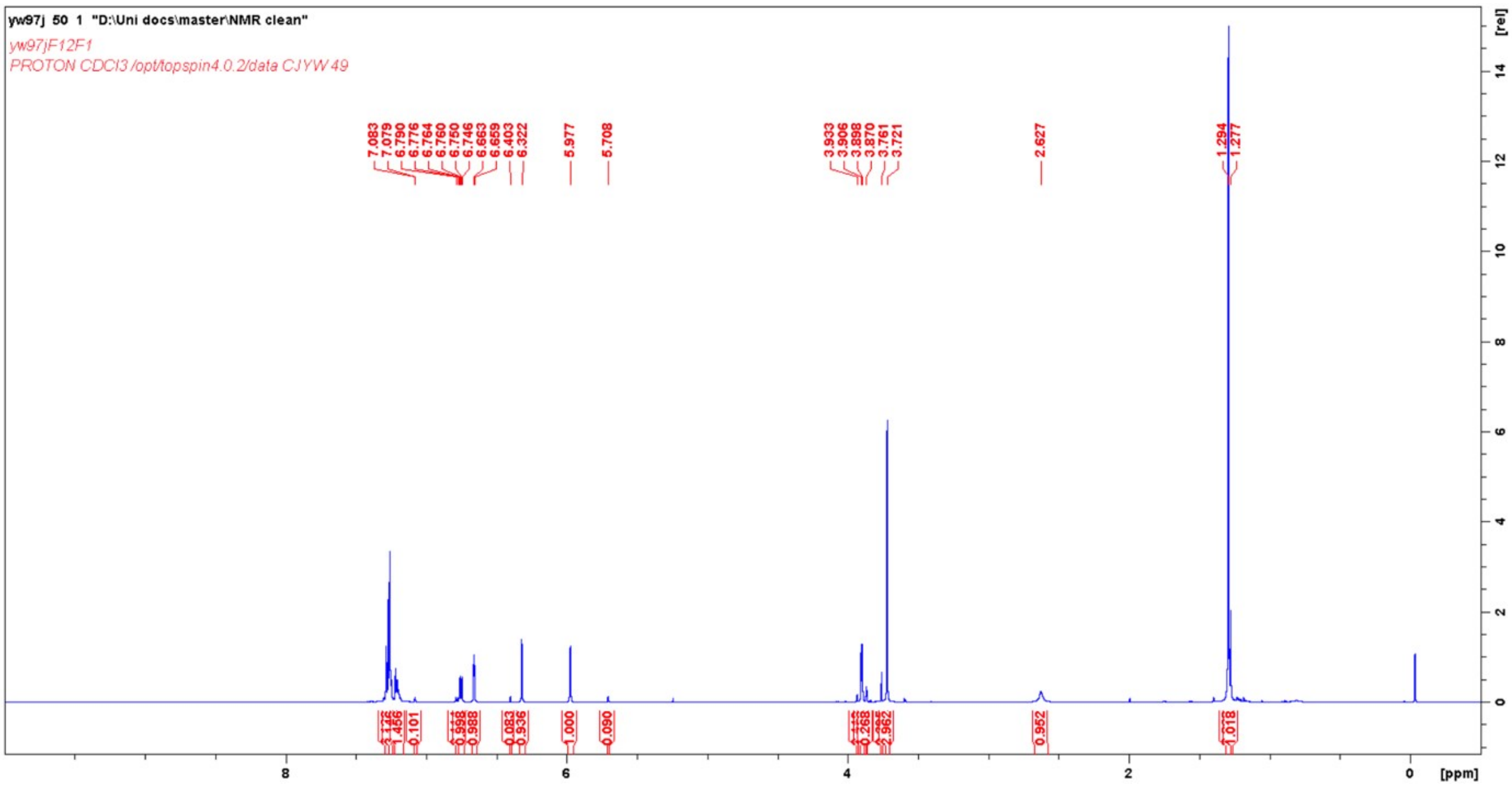

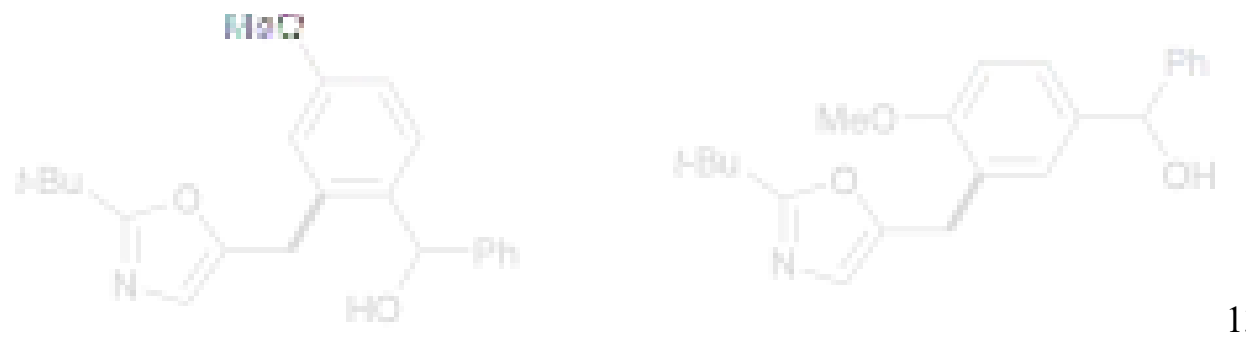

^{13}C NMR (101 MHz, $CDCl_3$), **15m & 15m'**

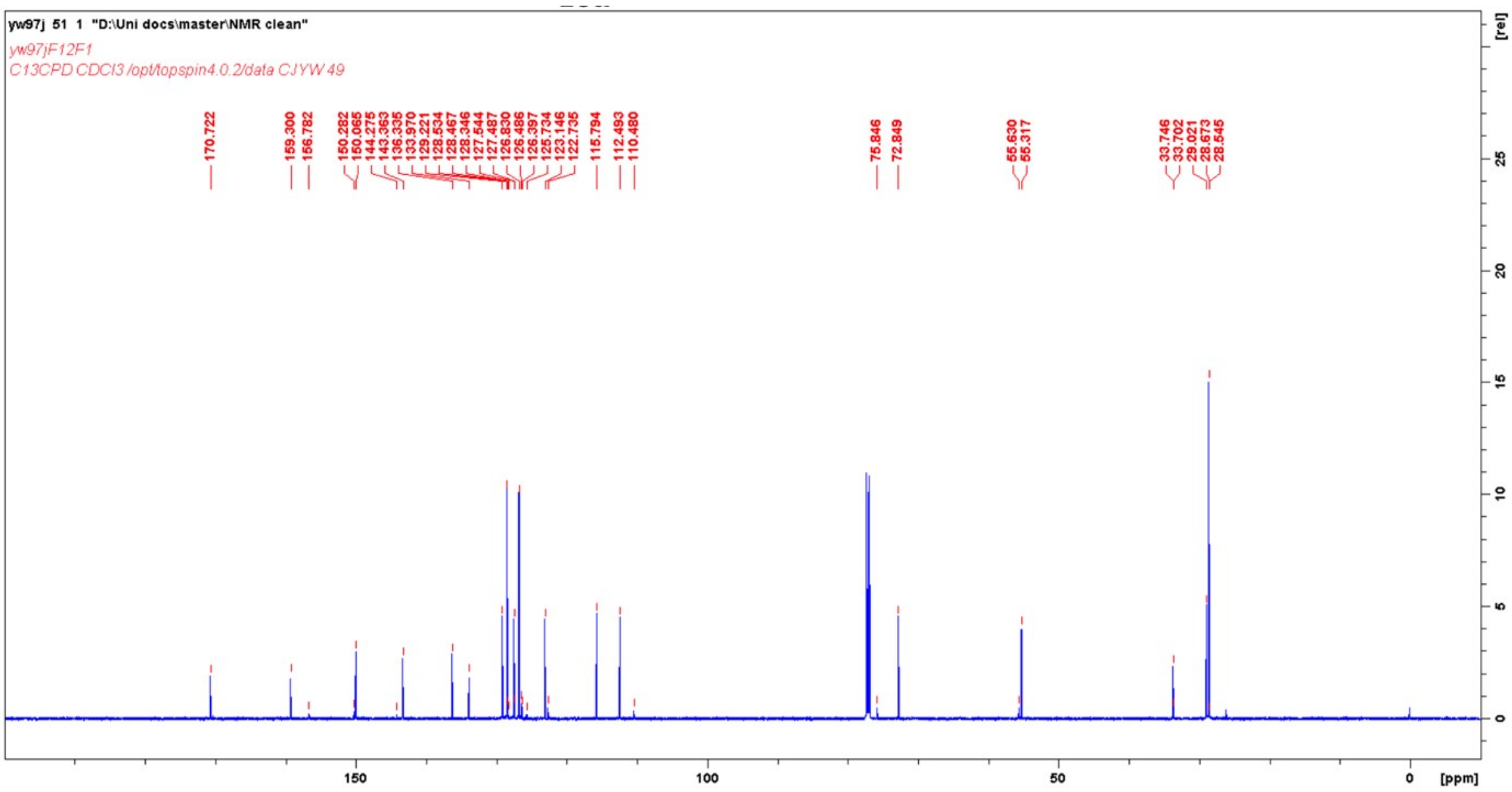

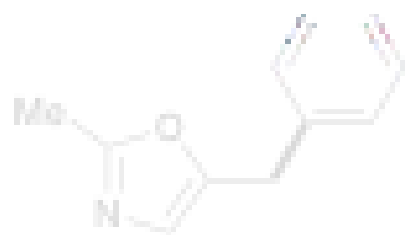

^{1}H NMR (400 MHz, $CDCl_3$), **21a**

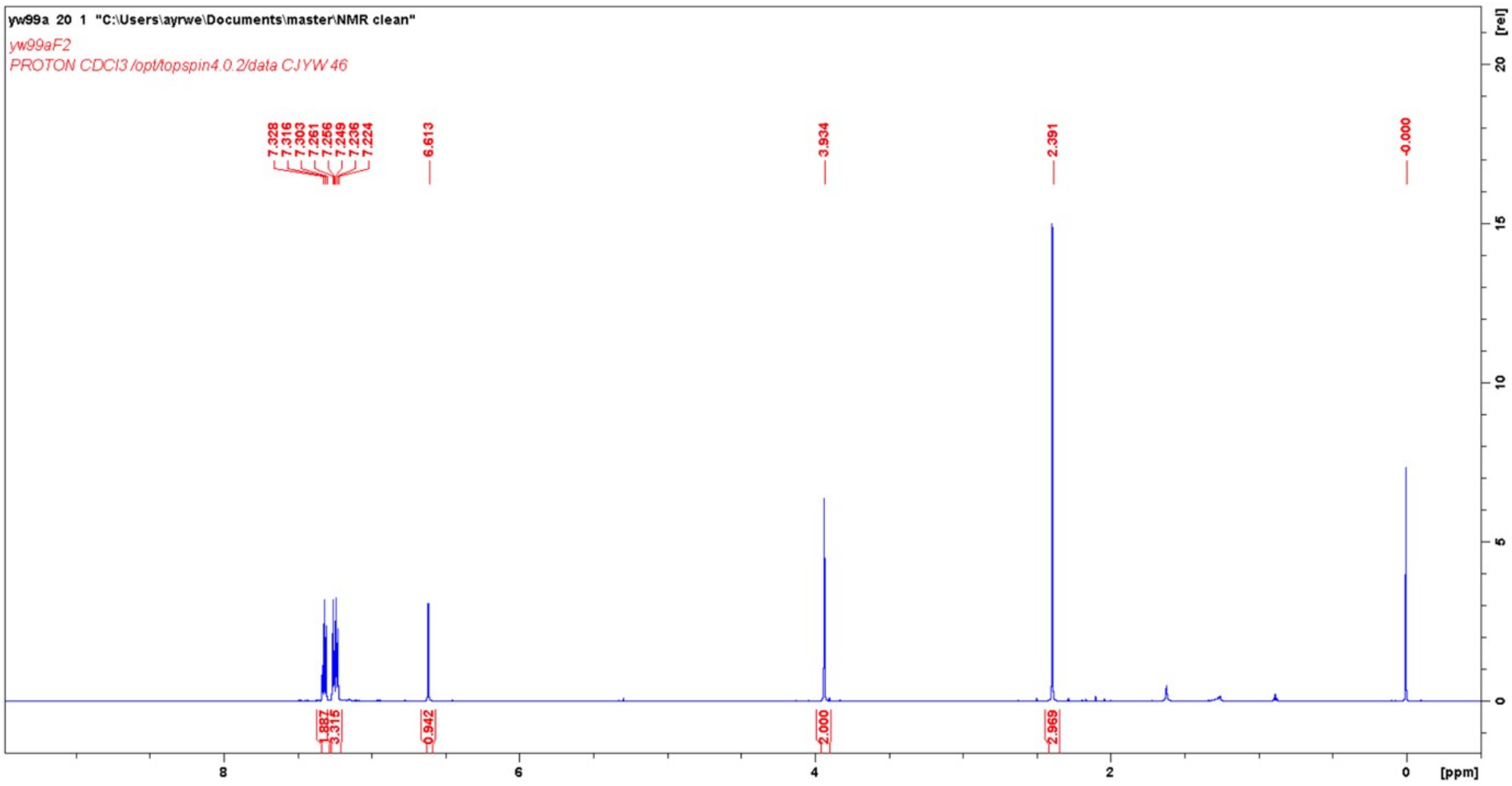

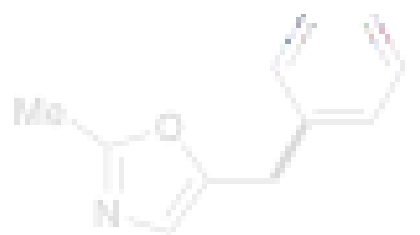

^{13}C NMR (101 MHz, $CDCl_3$), **21a**

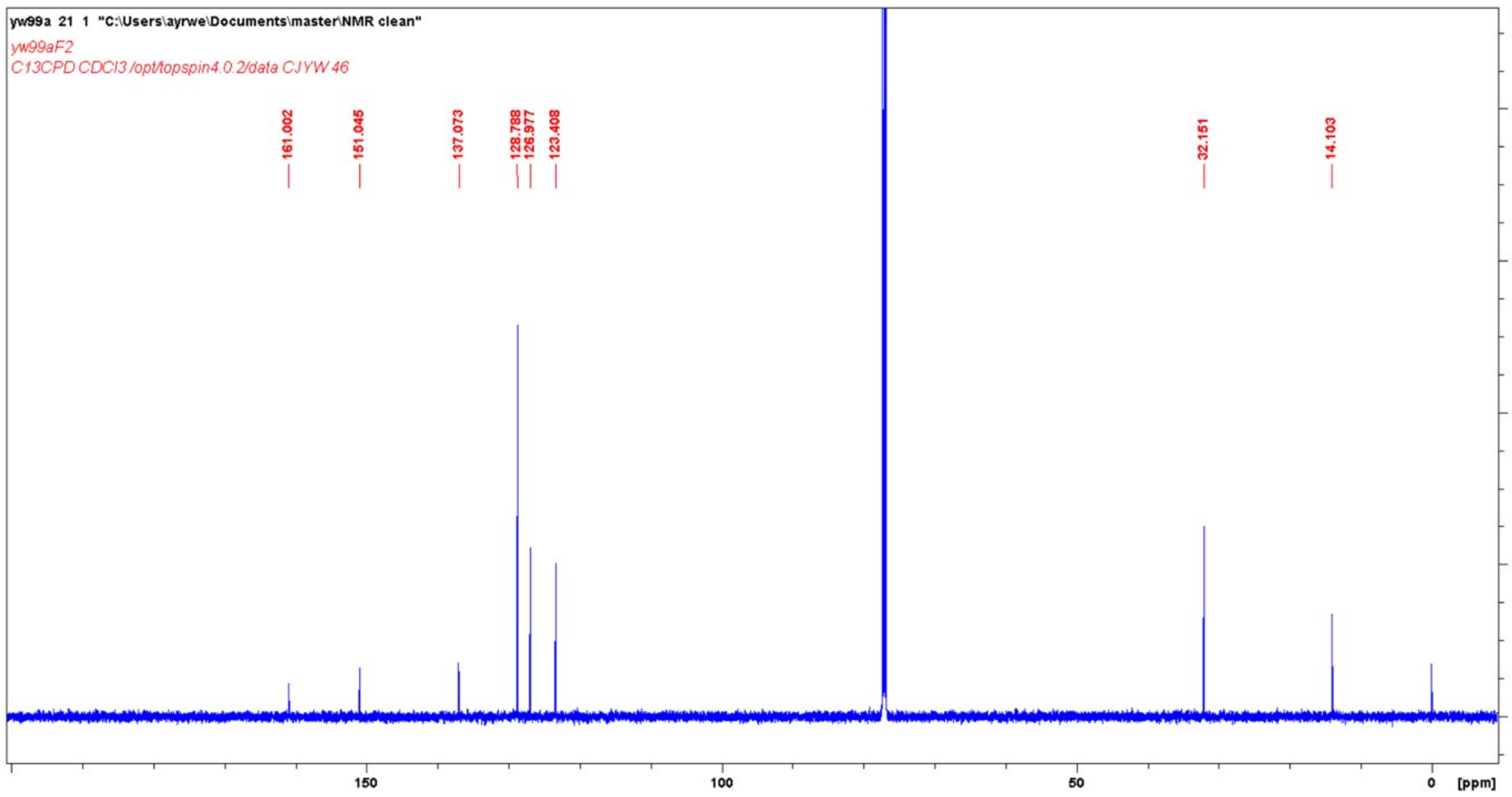

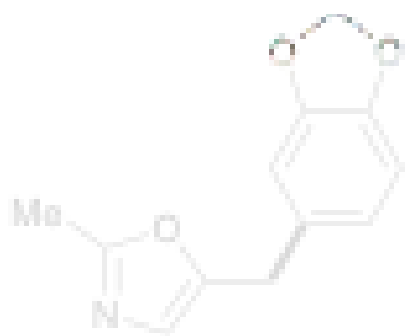

^{1}H NMR (400 MHz, $CDCl_3$), **21b**

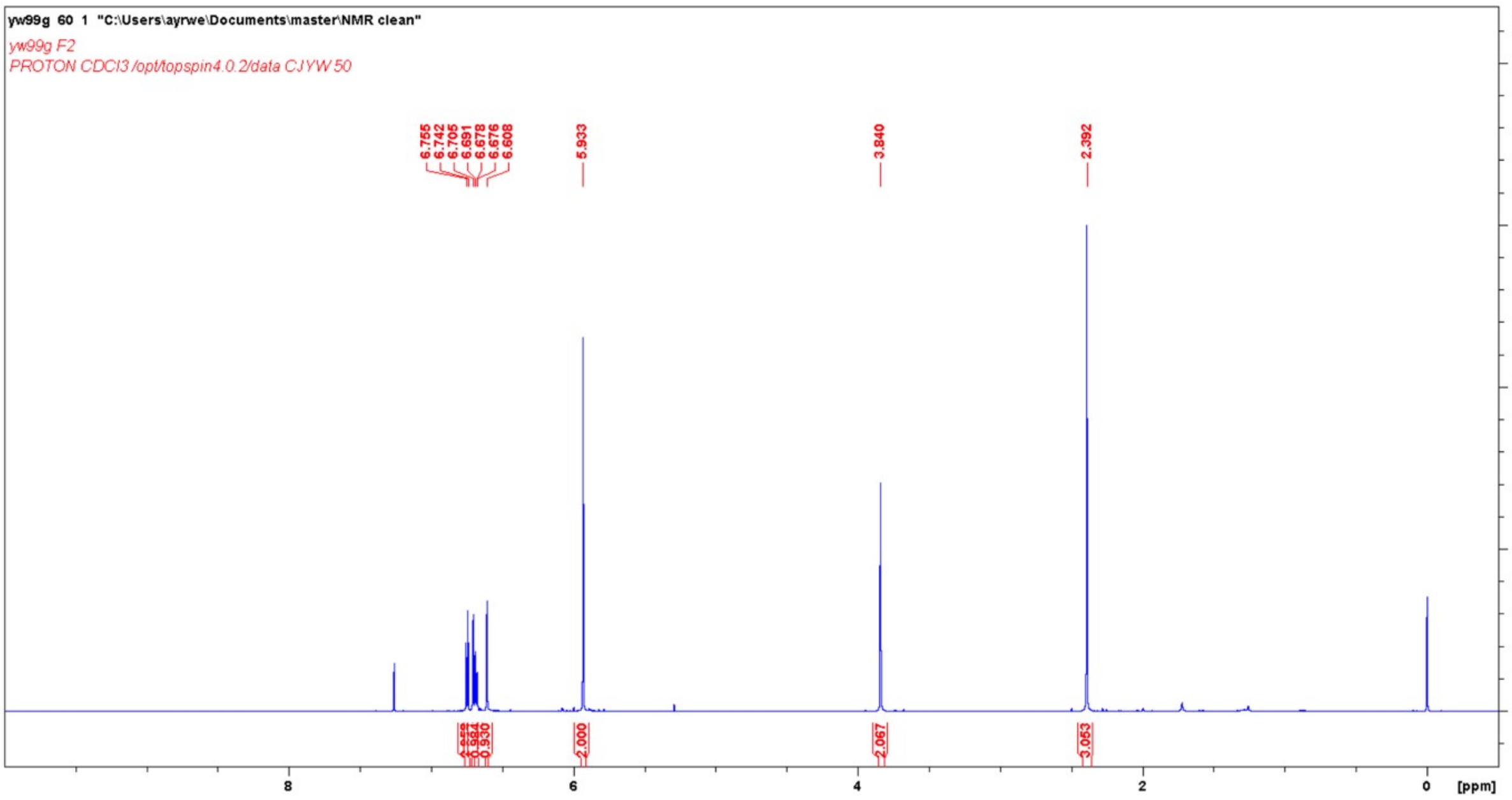

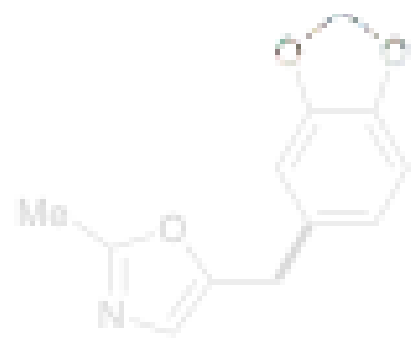

^{13}C NMR (101 MHz, $CDCl_3$), **21b**

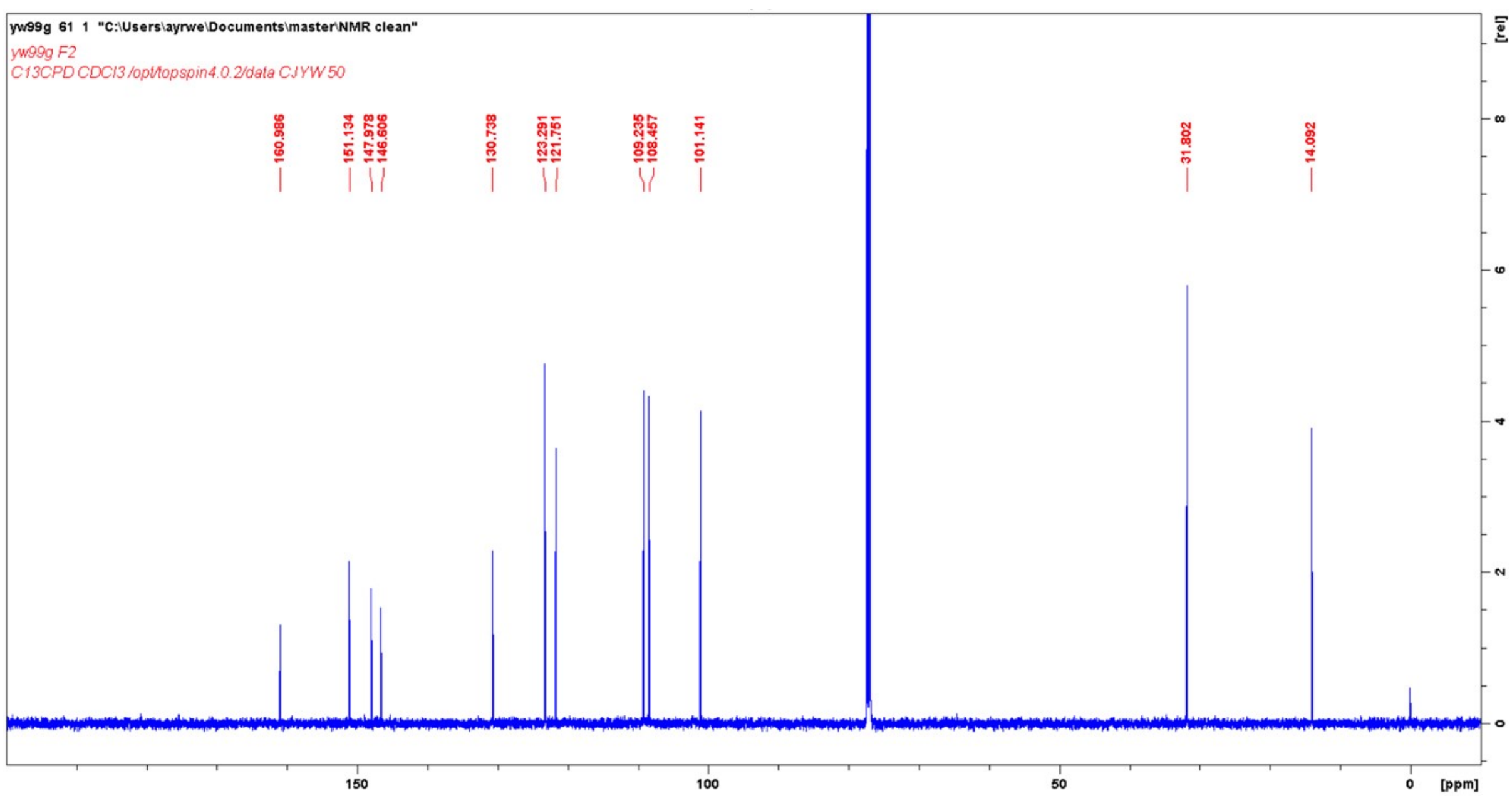

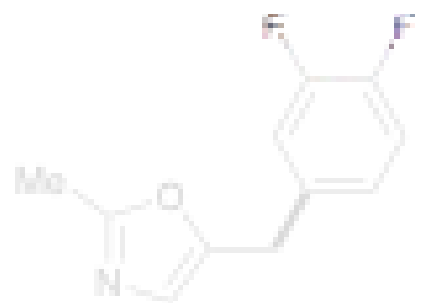

^{1}H NMR (400 MHz, $CDCl_3$), **21c**

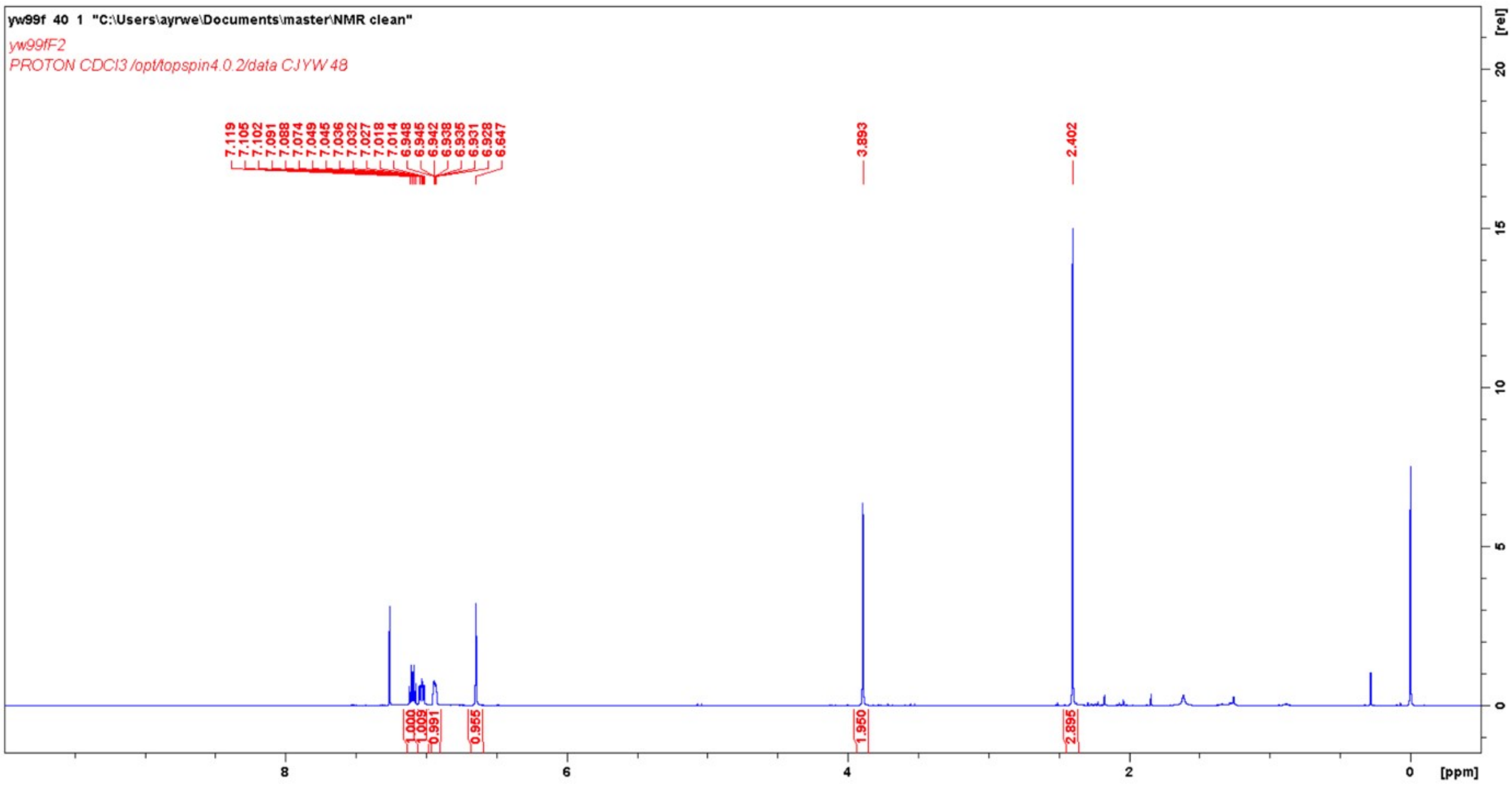

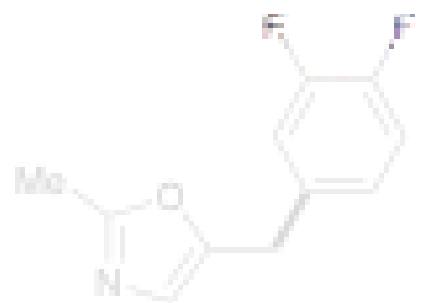

^{13}C NMR (101 MHz, $CDCl_3$), **21c**

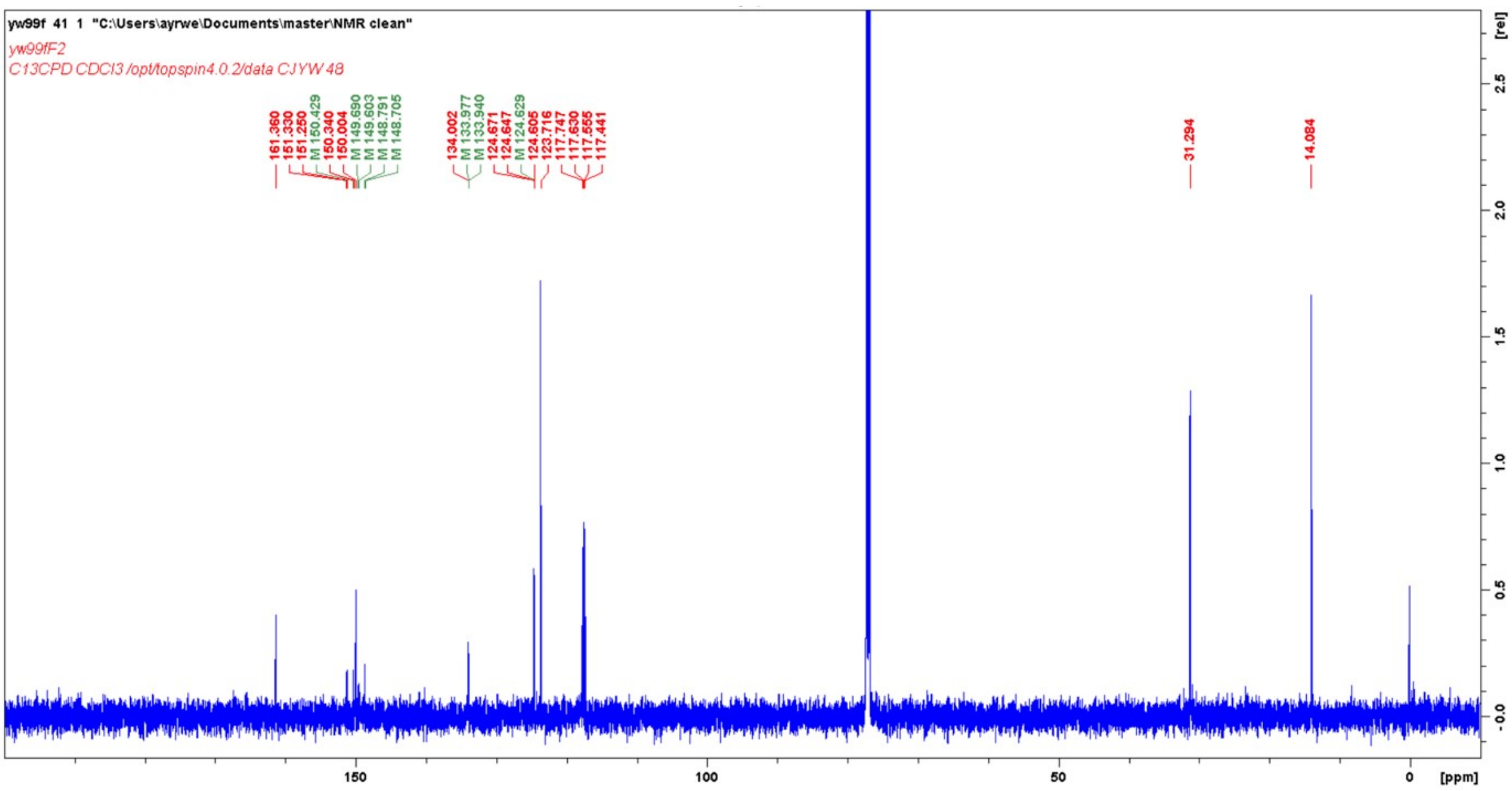

^1H NMR (400 MHz, $CDCl_3$), **21f**

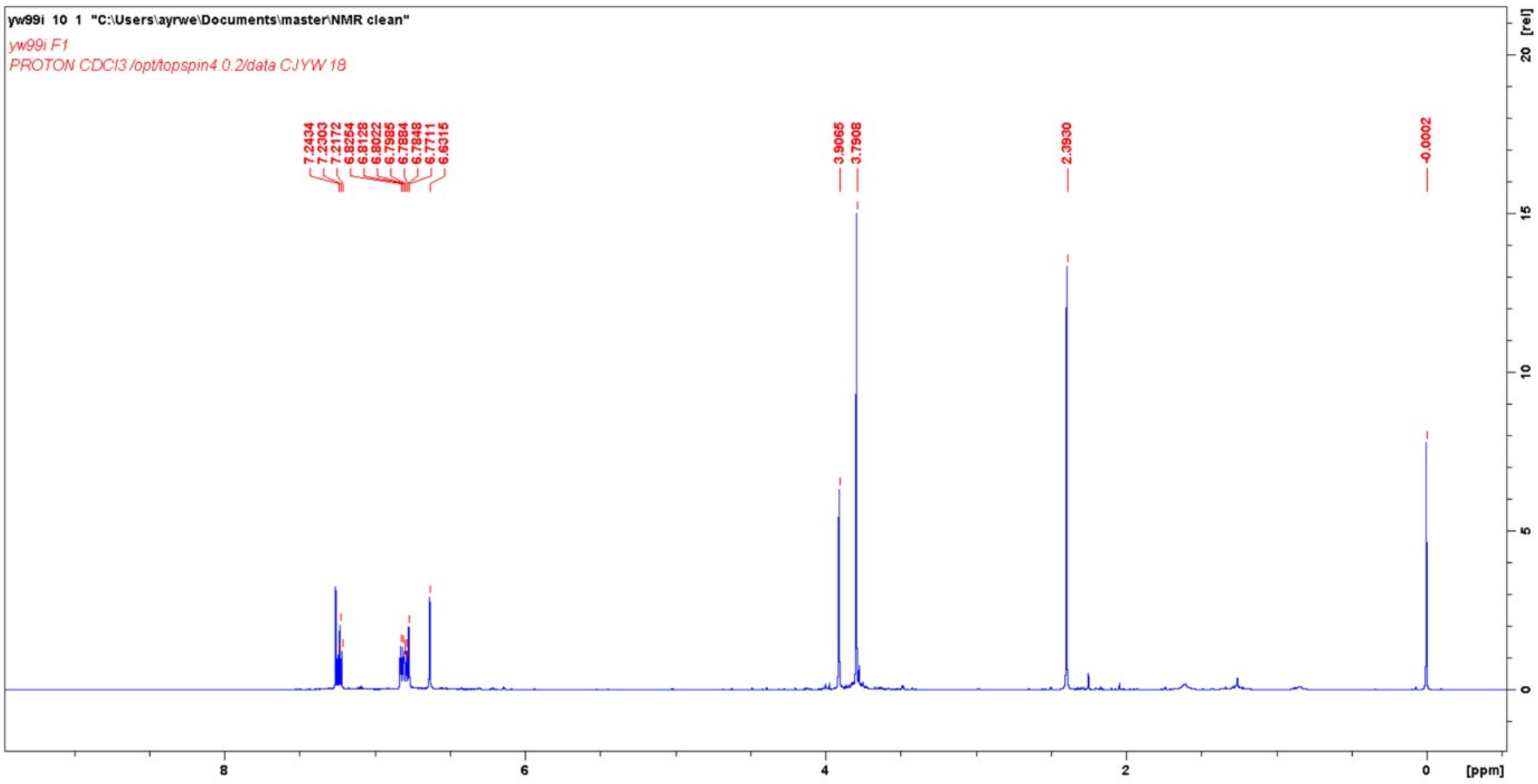

^{13}C NMR (101 MHz, $CDCl_3$), **21f**

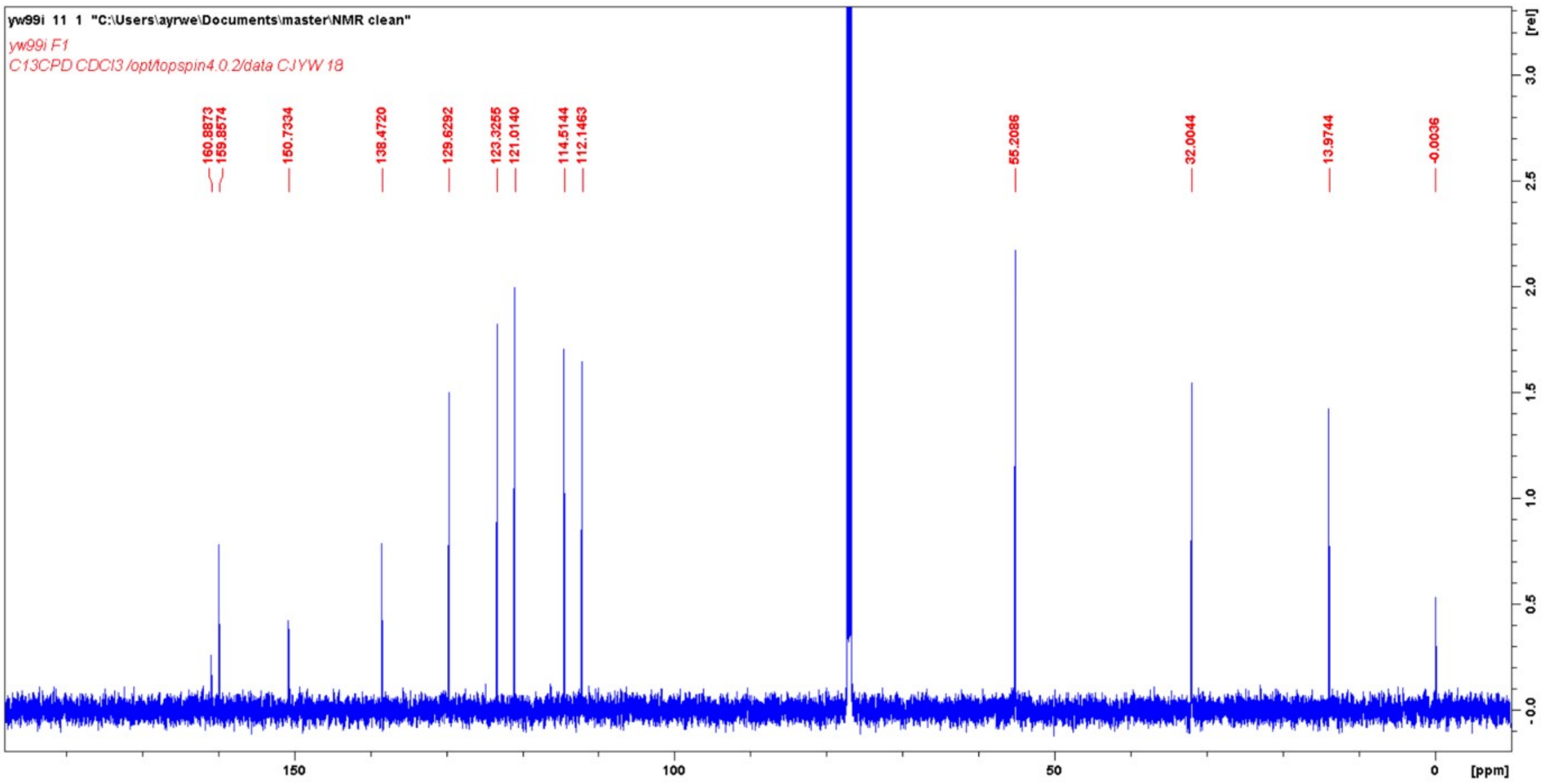

^{1}H NMR (400 MHz, $CDCl_3$), **21h & 21h’**

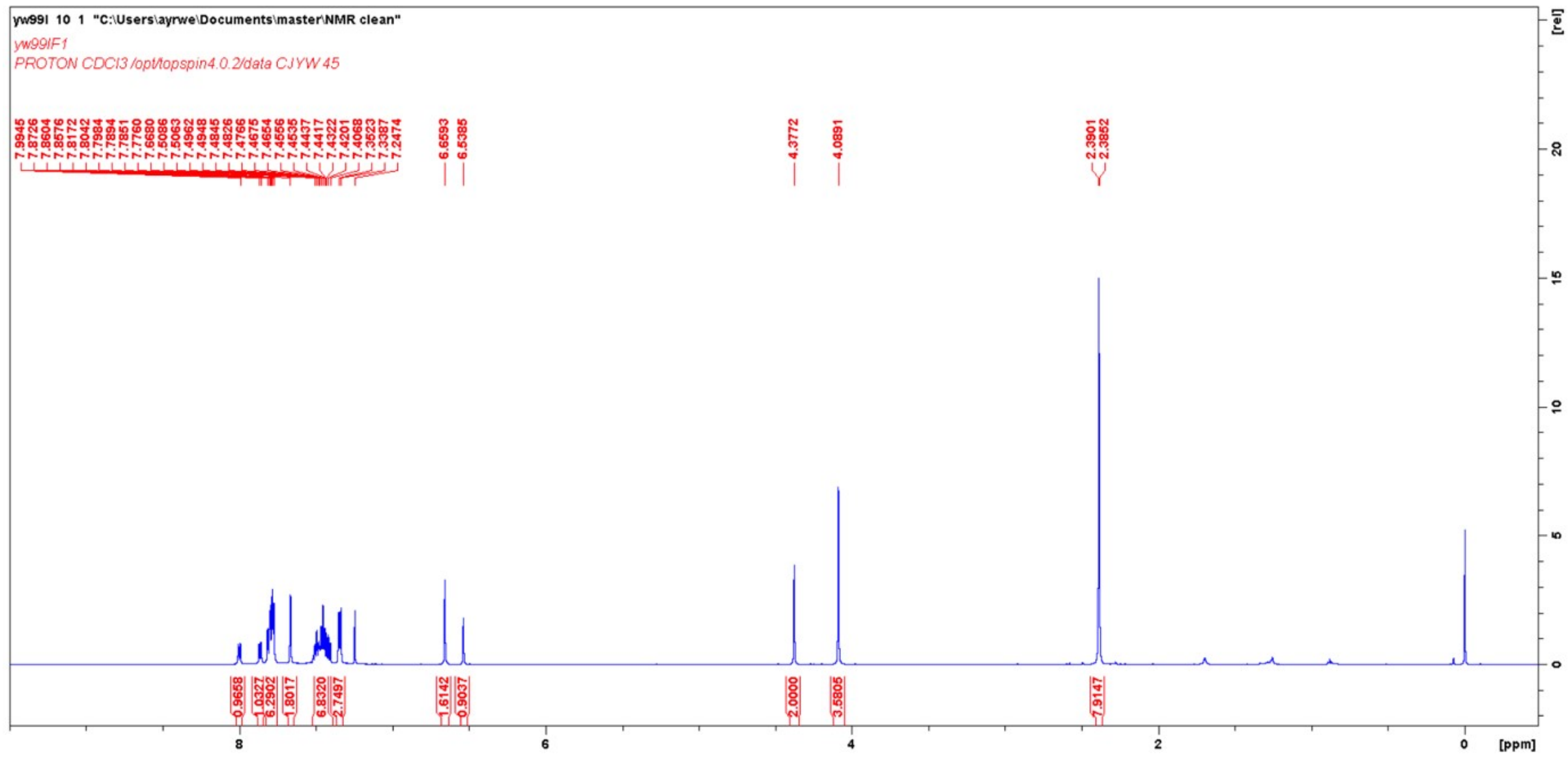

^{13}C NMR (101 MHz, $CDCl_3$), **21h & 21h'**

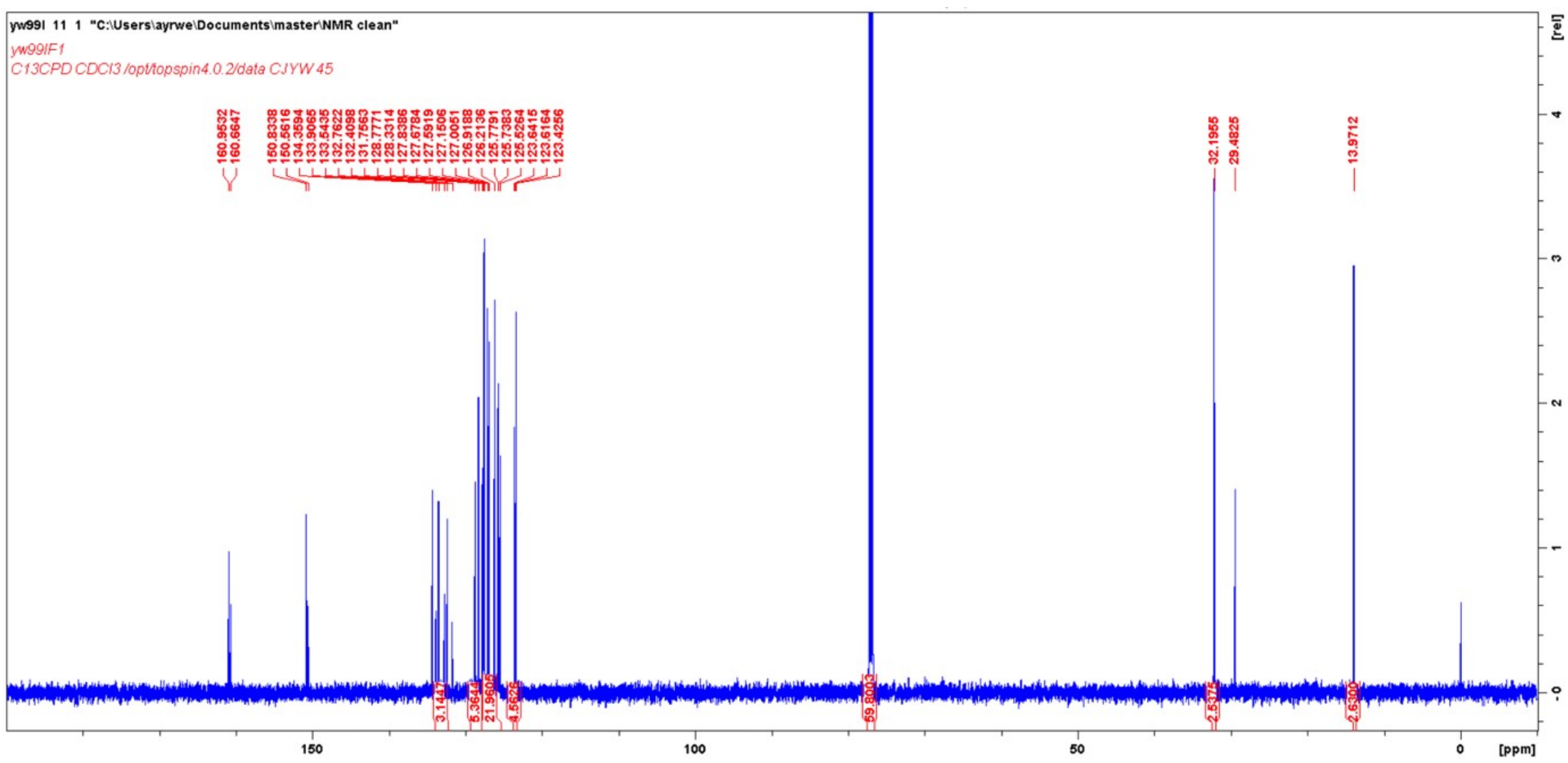

^{1}H NMR (400 MHz, $CDCl_3$), **21j & 21j'**

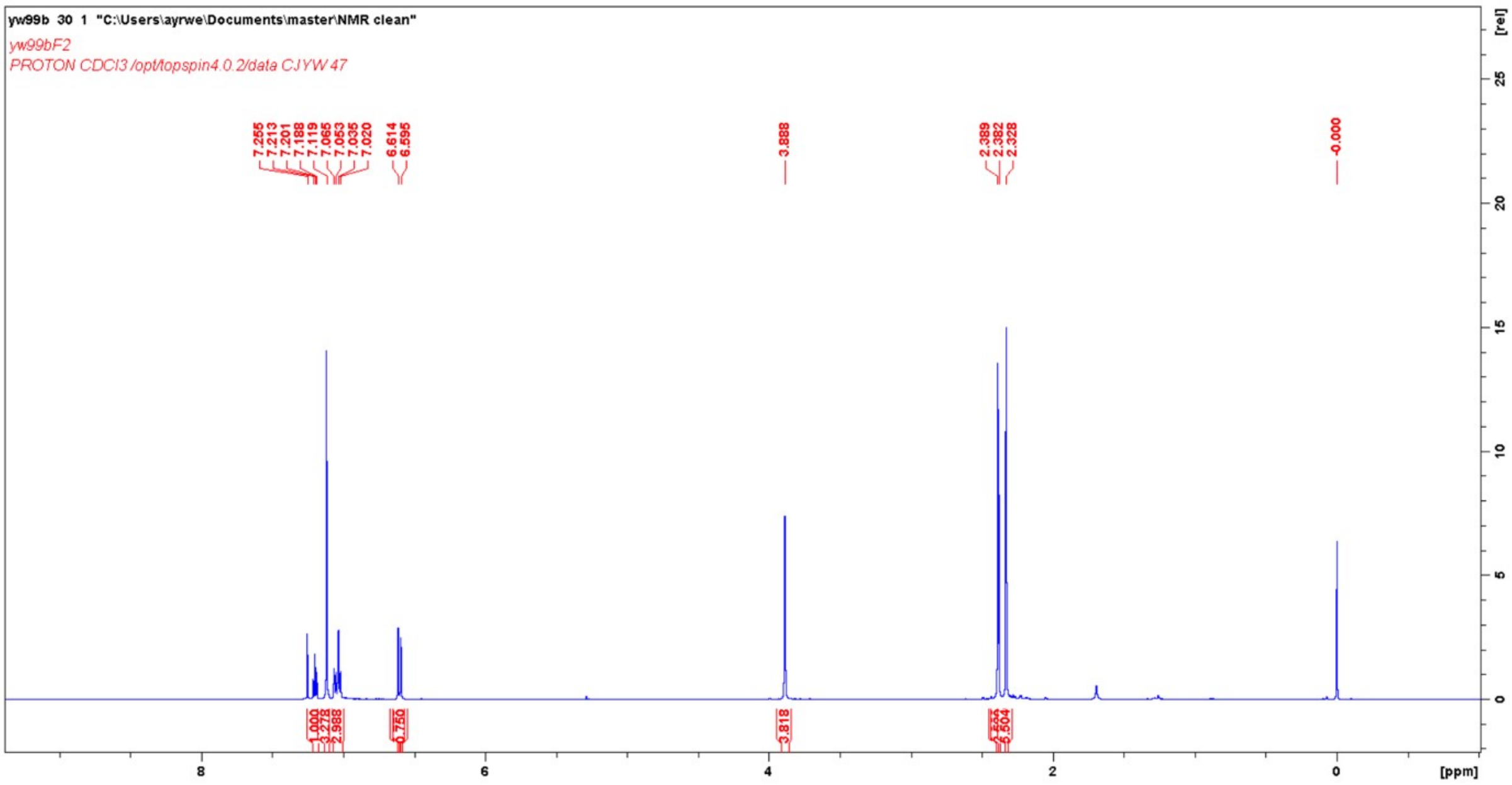

^{13}C NMR (101 MHz, $CDCl_3$), **21j & 21j'**

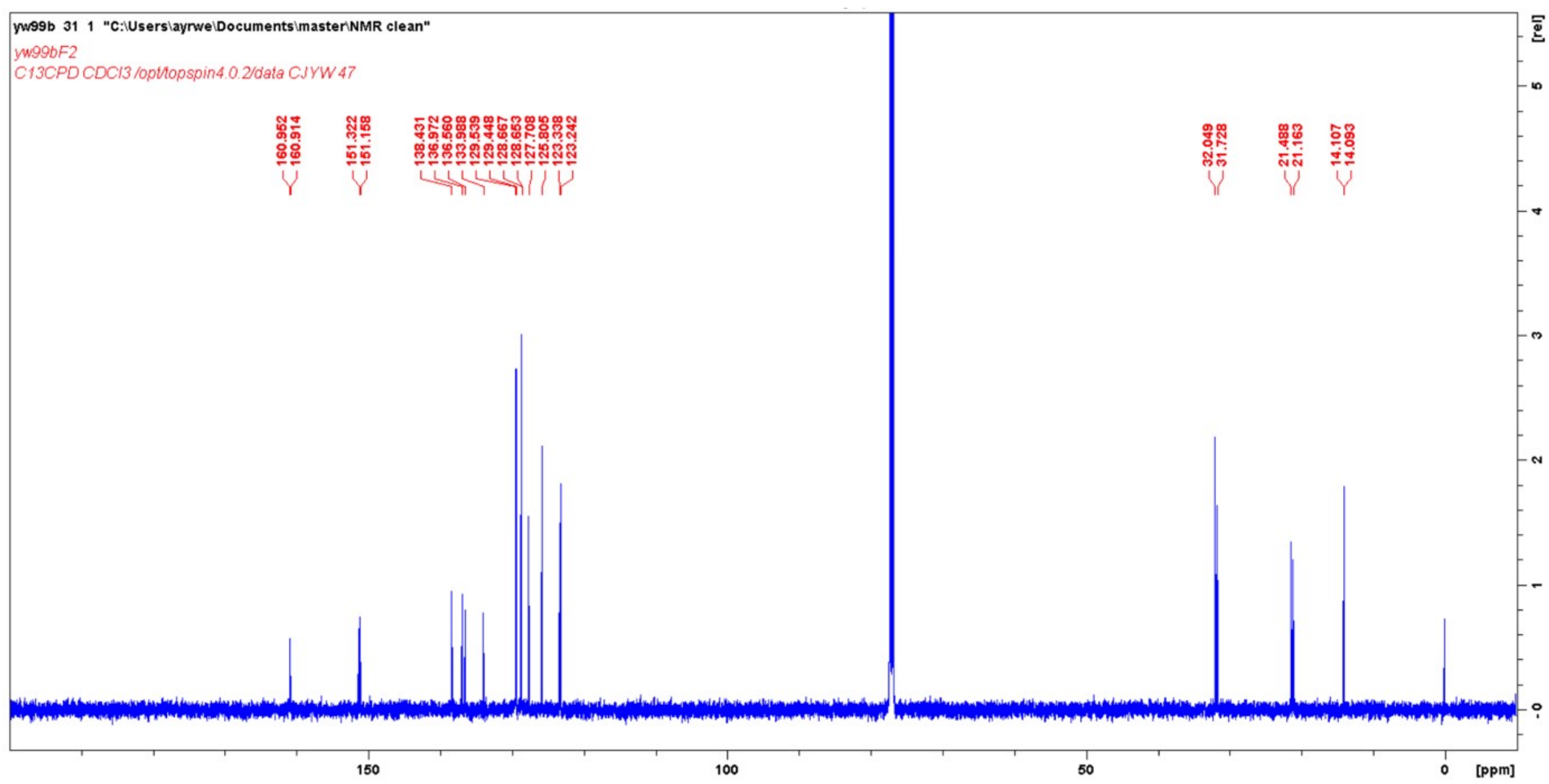

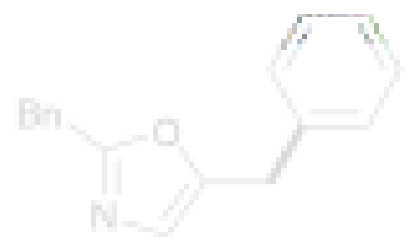

^{1}H NMR (400 MHz, $CDCl_3$), **22a**

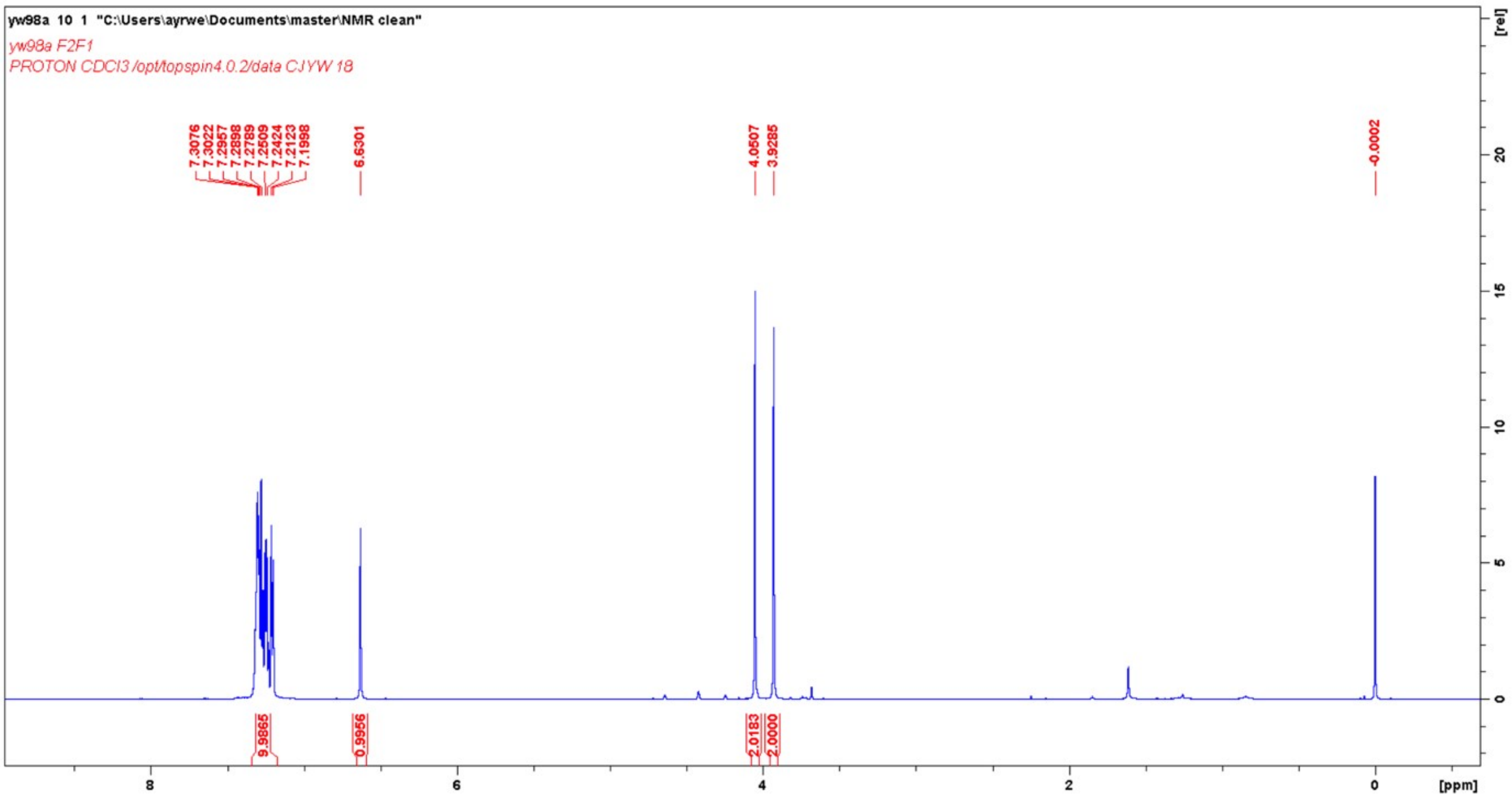

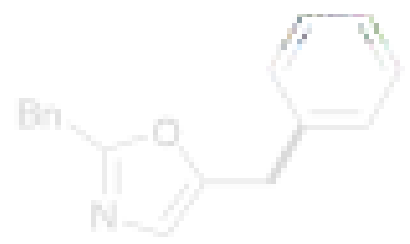

^{13}C NMR (101 MHz, $CDCl_3$), **22a**

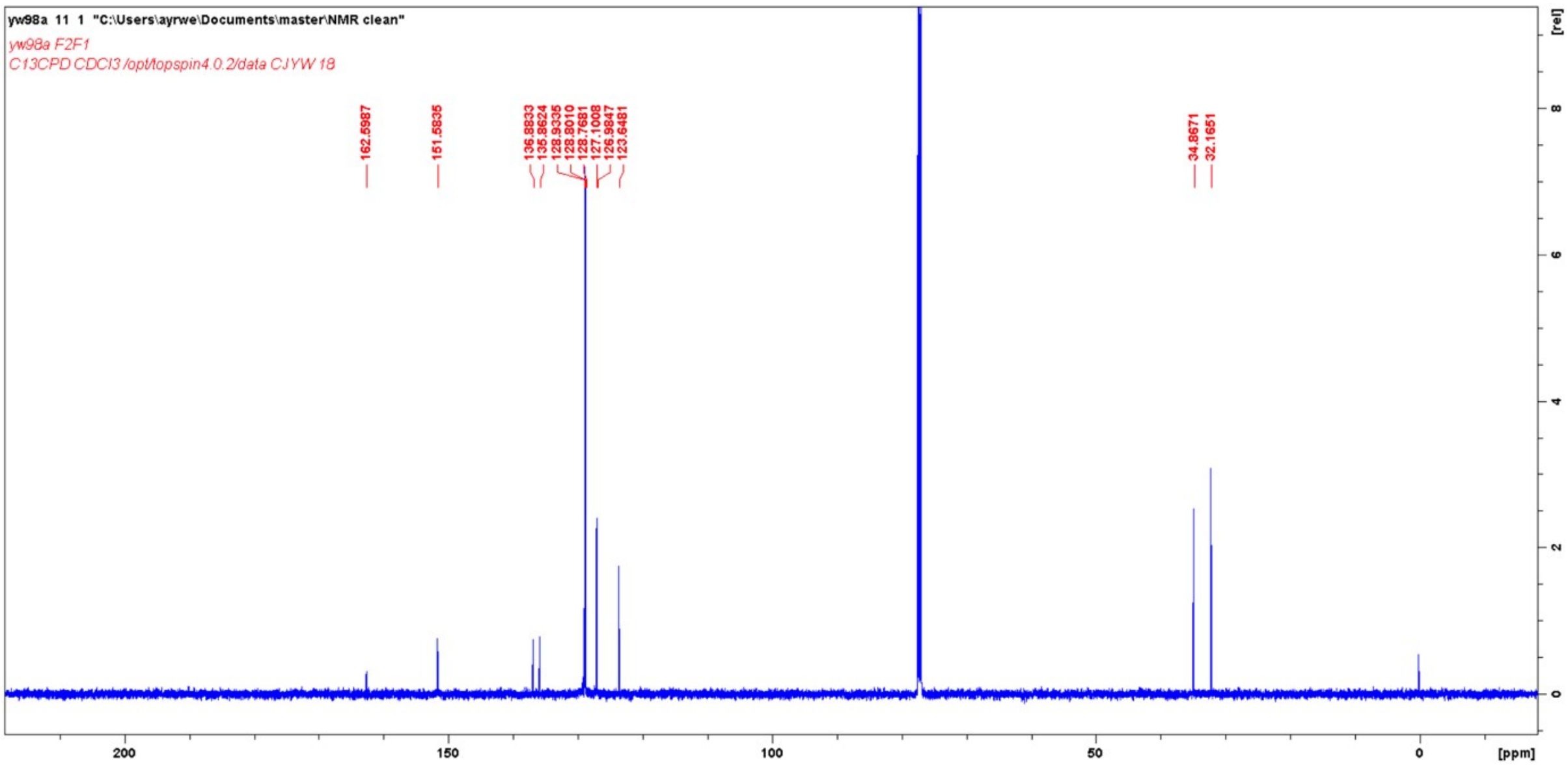

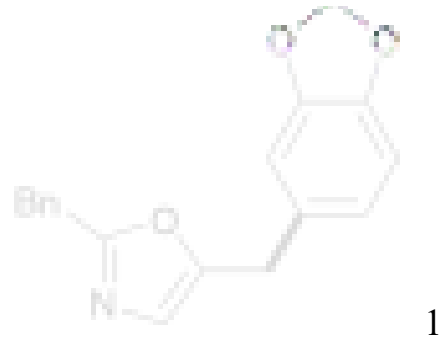

^{1}H NMR (400 MHz, $CDCl_3$), **22b**

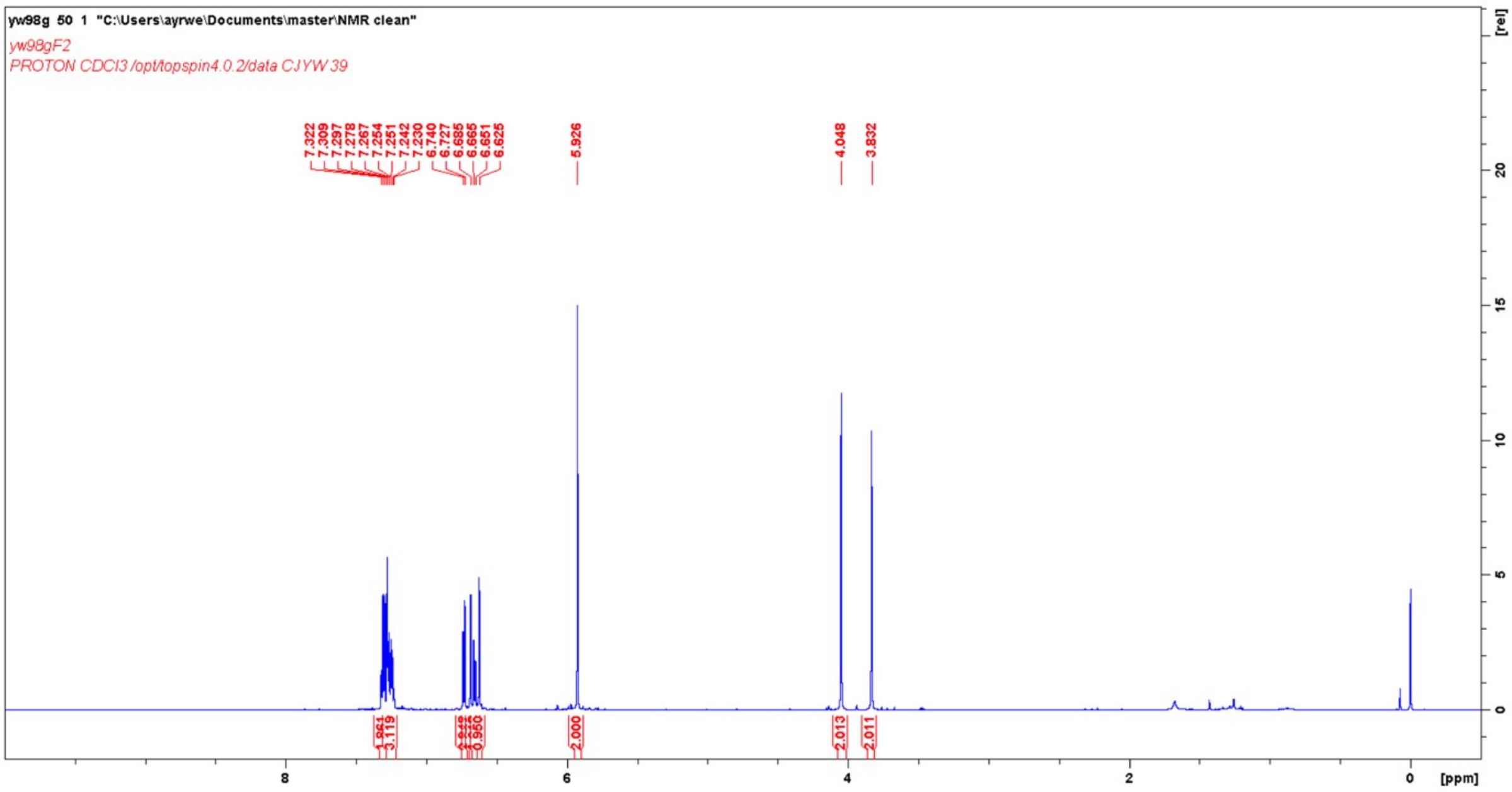

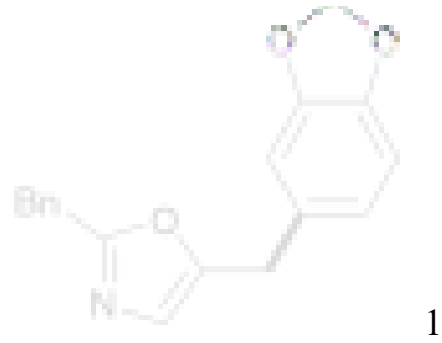

^{13}C NMR (101 MHz, $CDCl_3$), **22b**

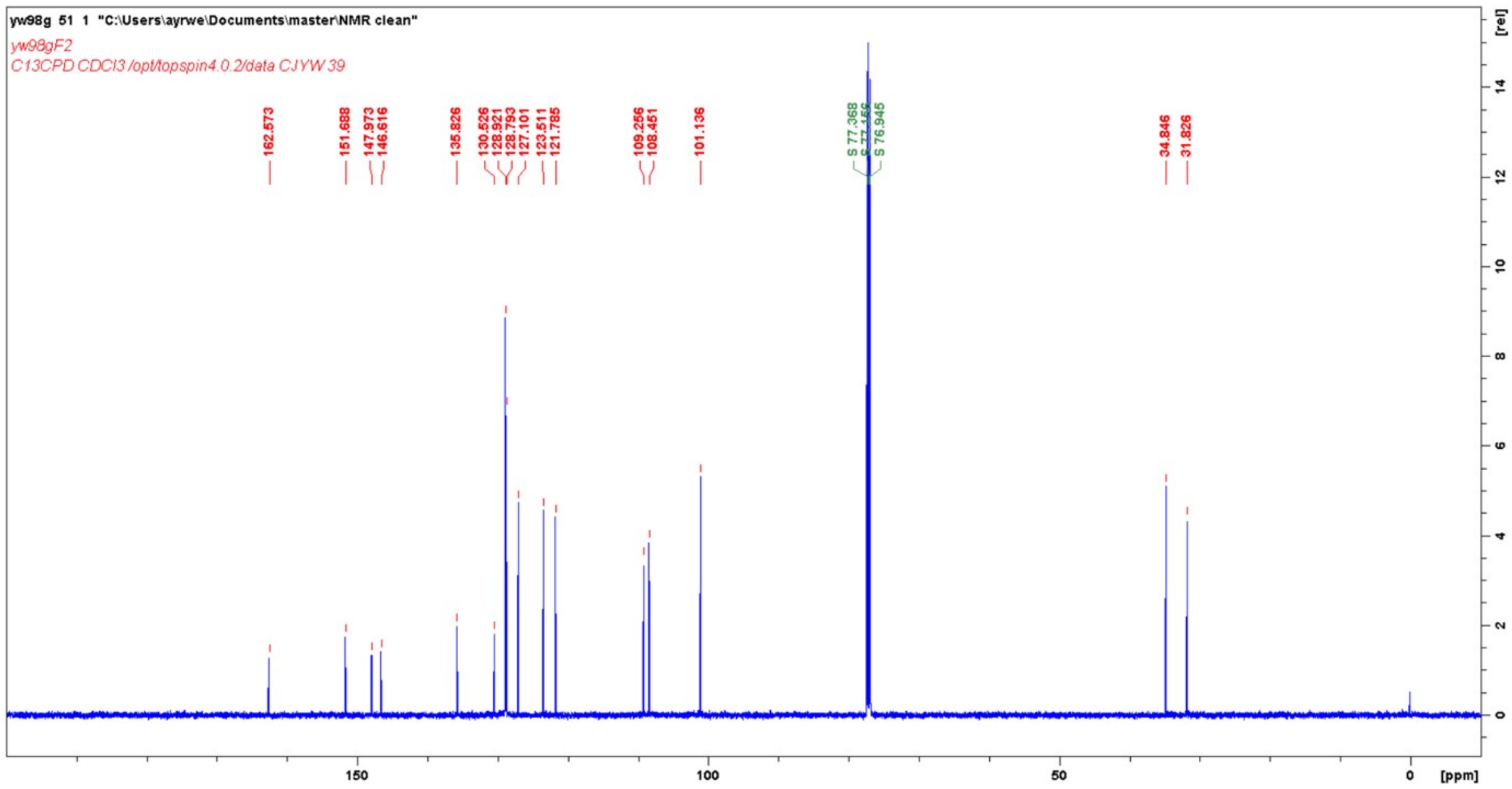

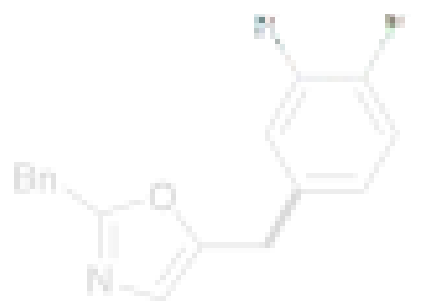

^{1}H NMR (400 MHz, $CDCl_3$), **22c**

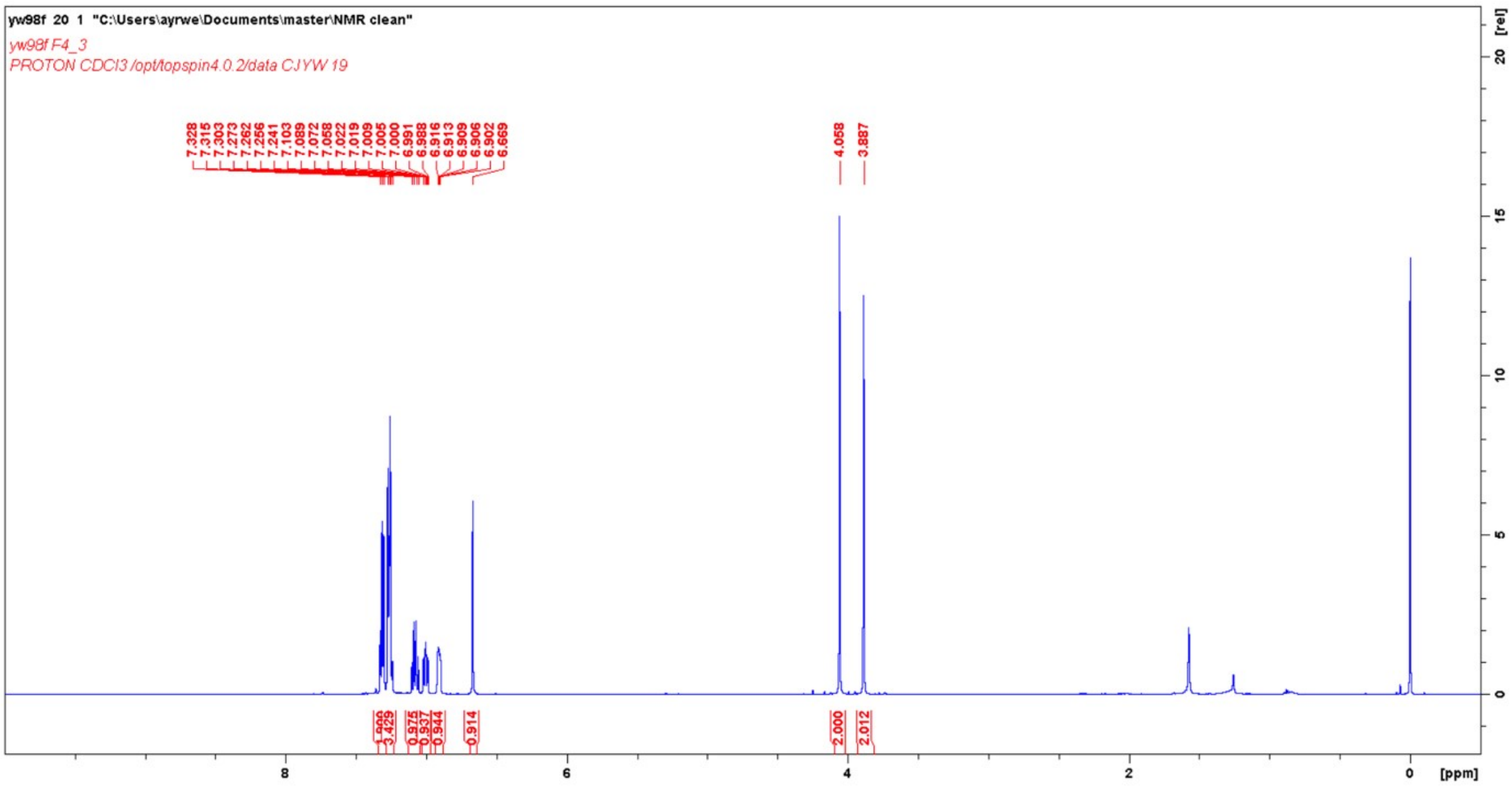

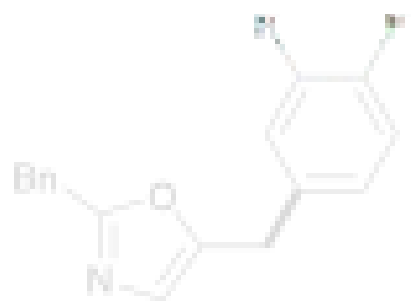

^{13}C NMR (101 MHz, $CDCl_3$), **22c**

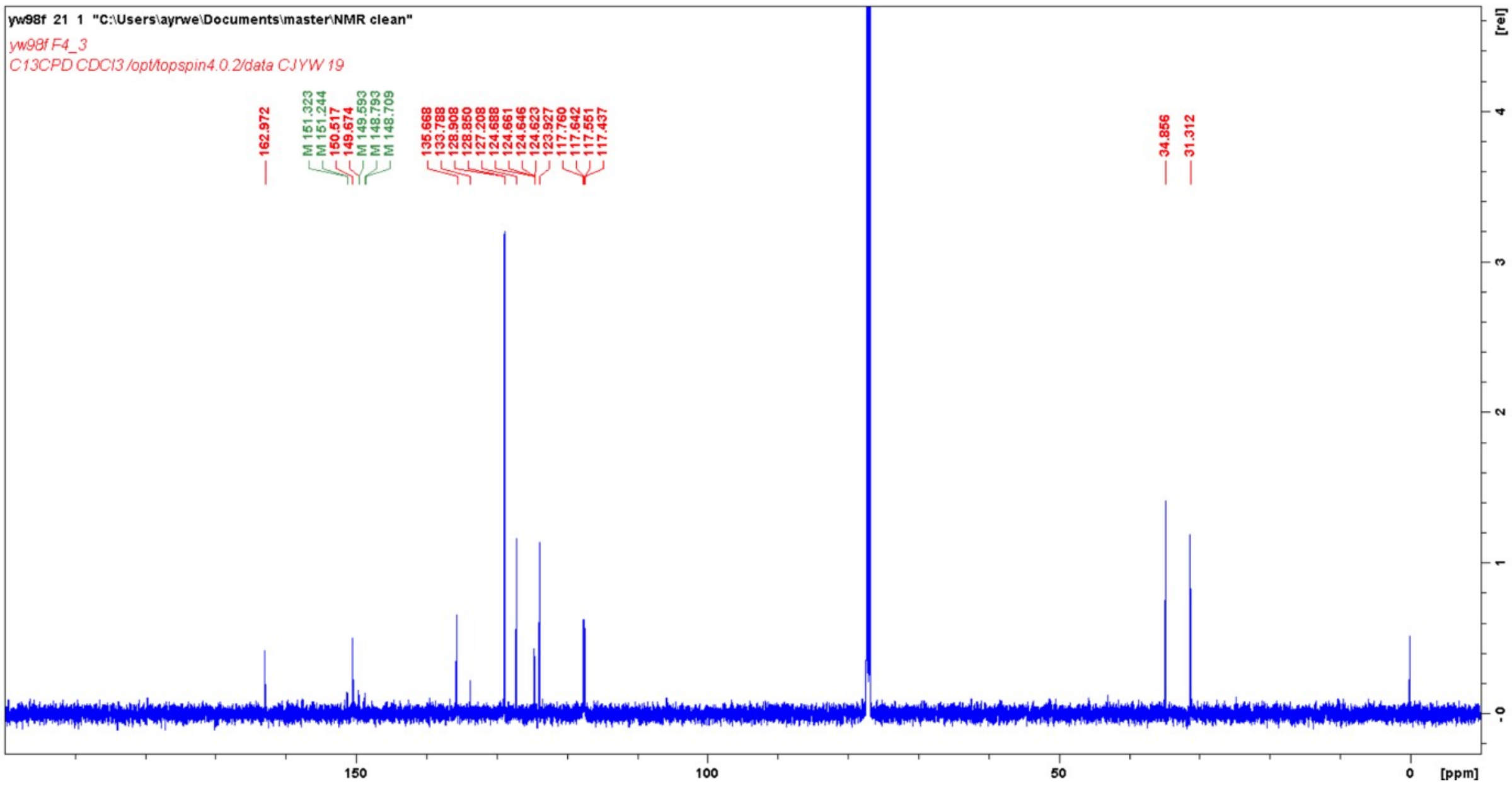

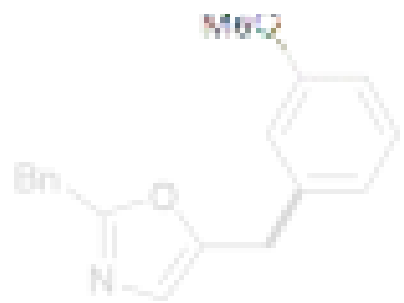

^{1}H NMR (400 MHz, $CDCl_3$), **22f**

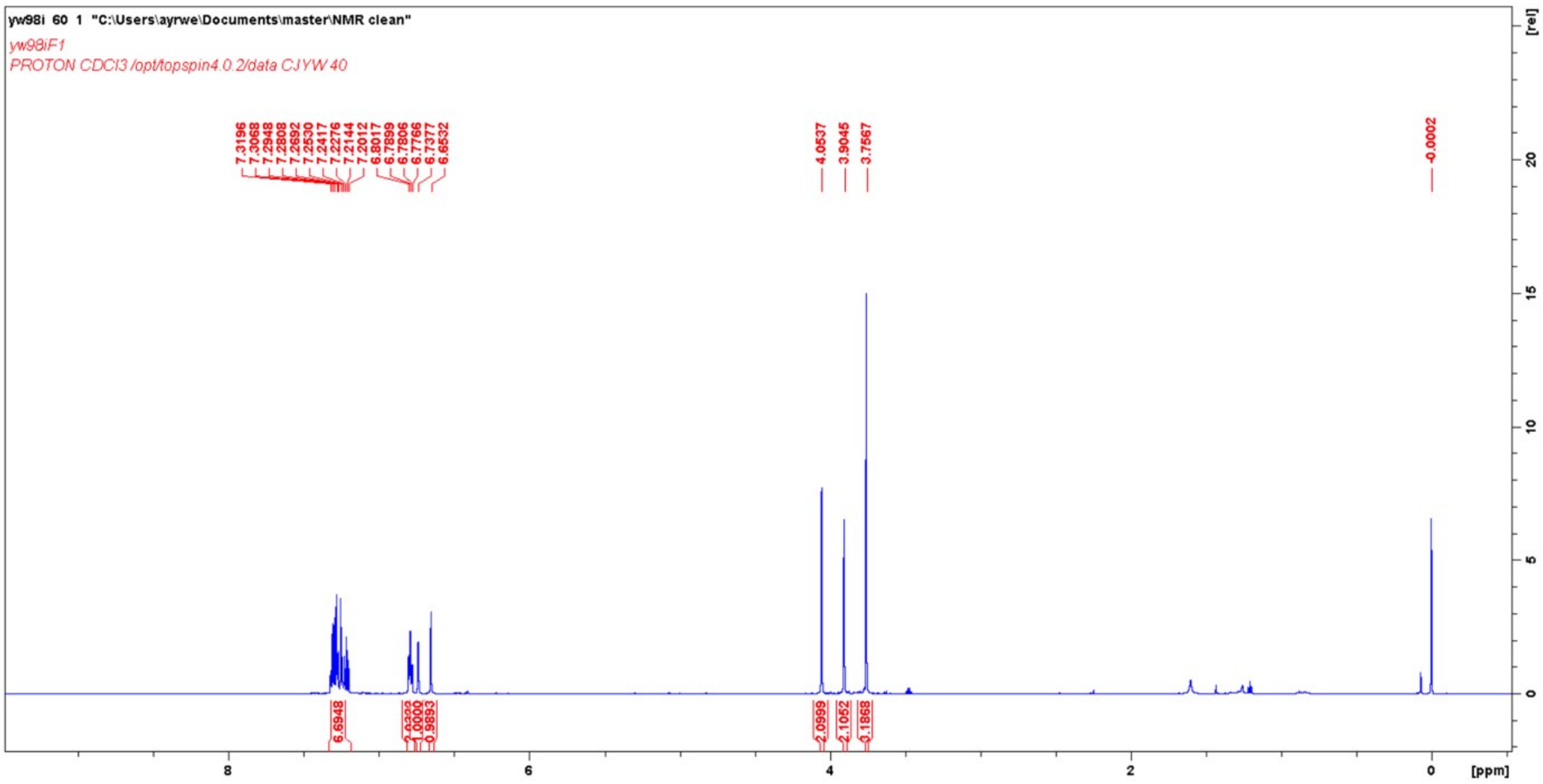

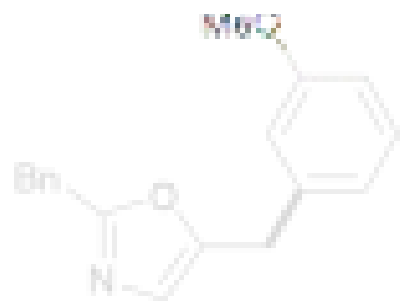

^{13}C NMR (101 MHz, $CDCl_3$), **22f**

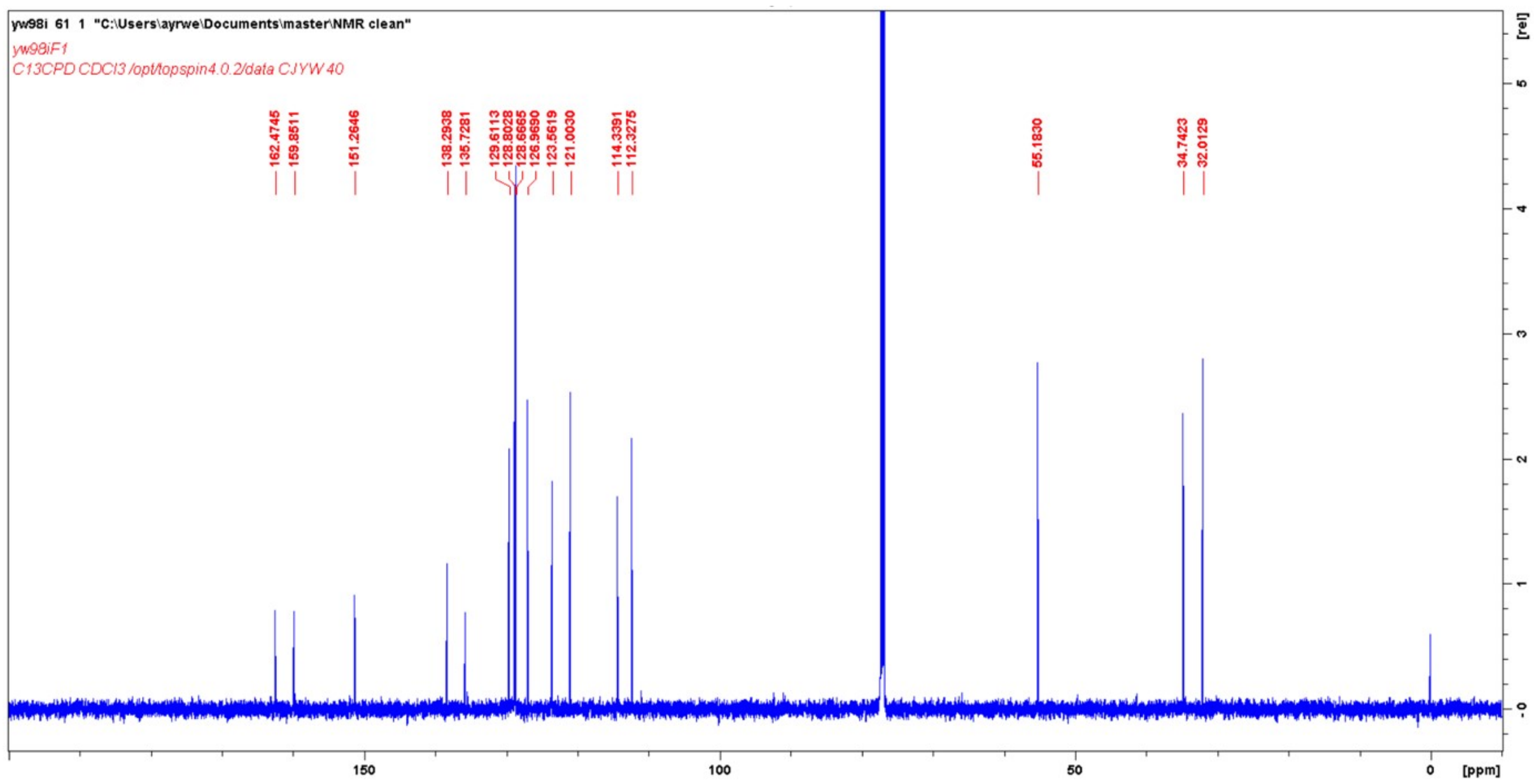

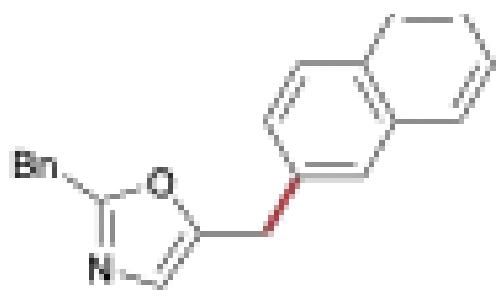
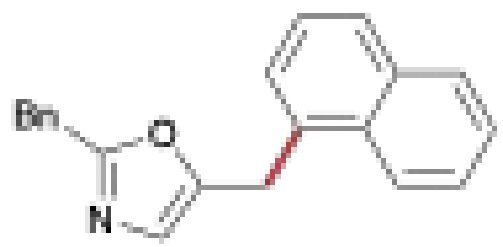

^{1}H NMR (400 MHz, $CDCl_3$), **22h & 22h'**

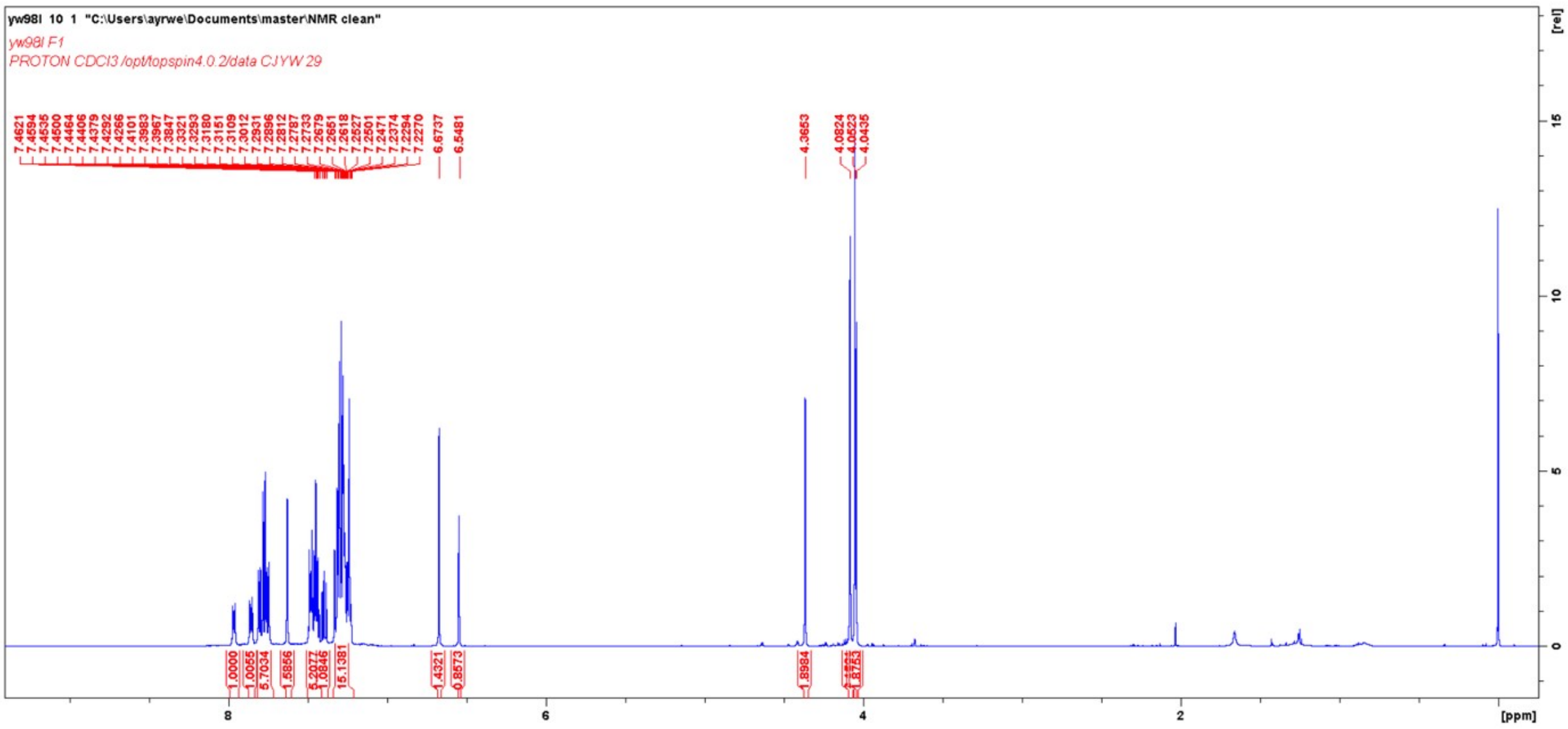

^{13}C NMR (101 MHz, $CDCl_3$), **22h & 22h'**

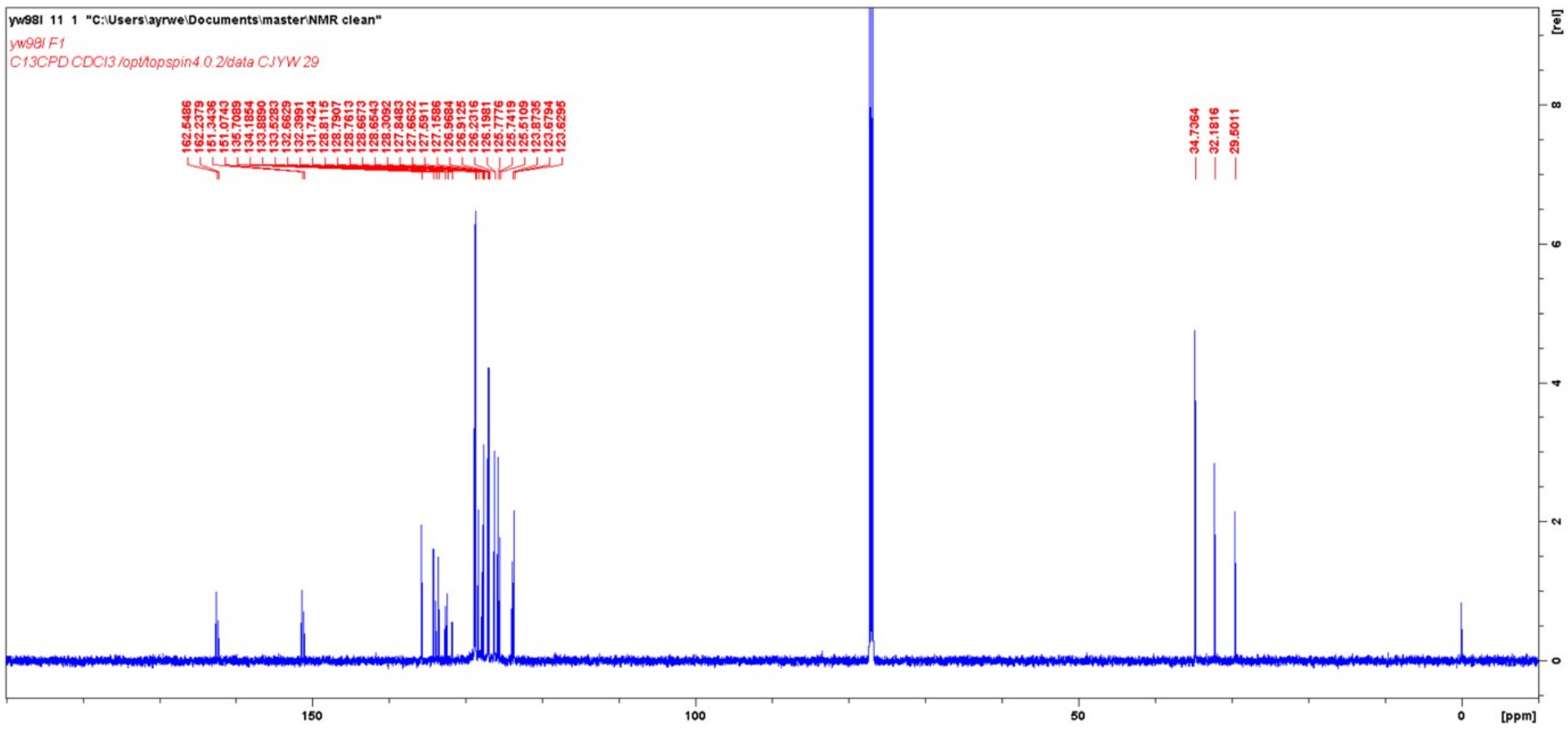

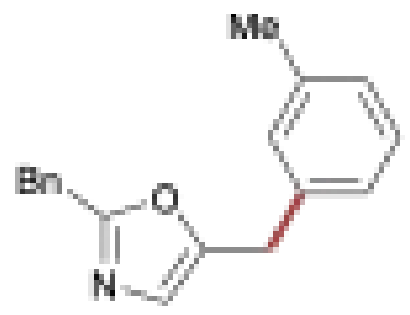

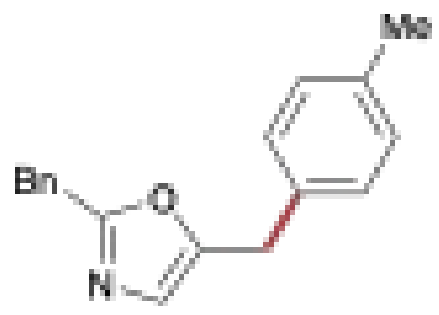

^{1}H NMR (400 MHz, $CDCl_3$), **22j & 22j'**

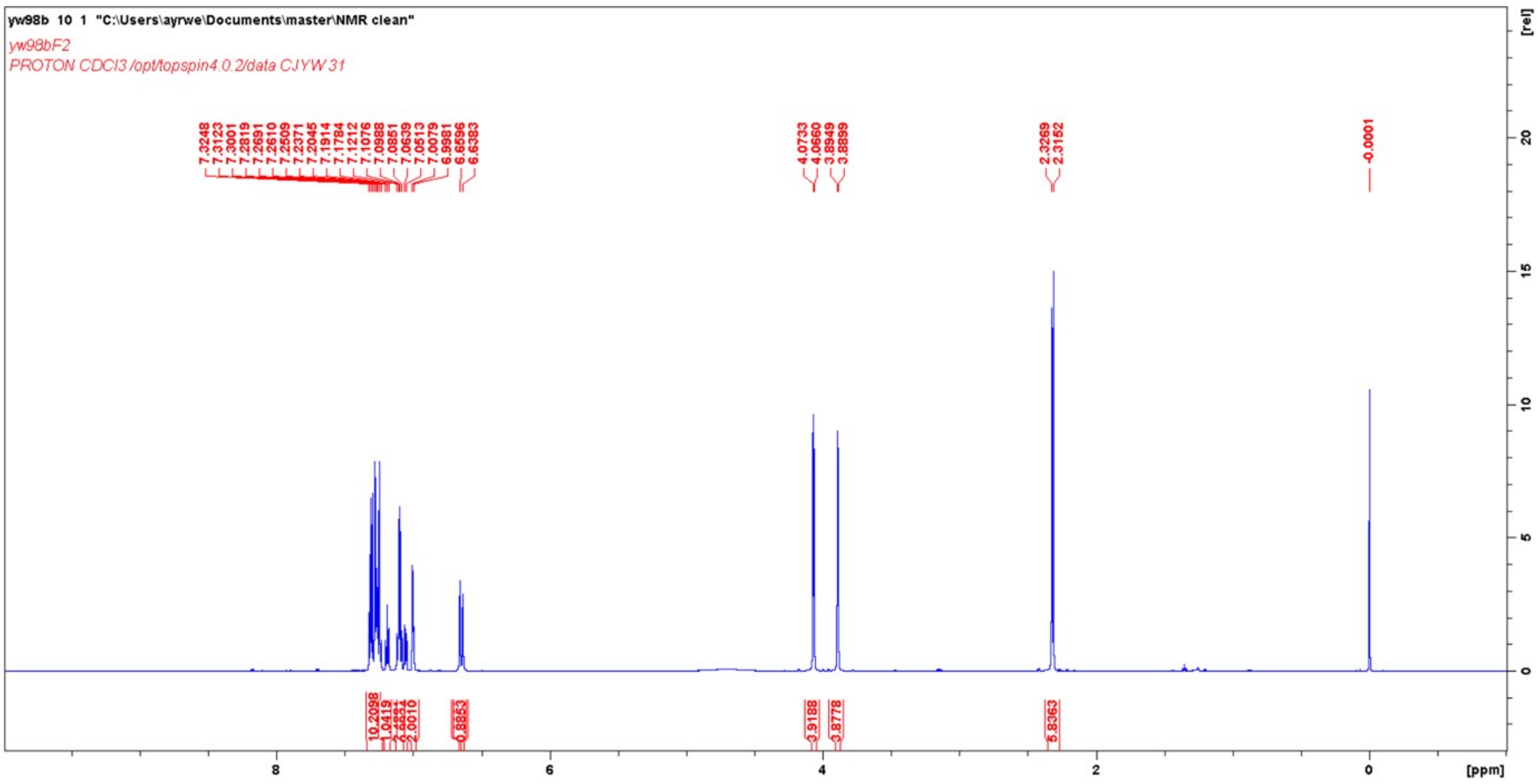

^{13}C NMR (101 MHz, $CDCl_3$), **22j & 22j'**

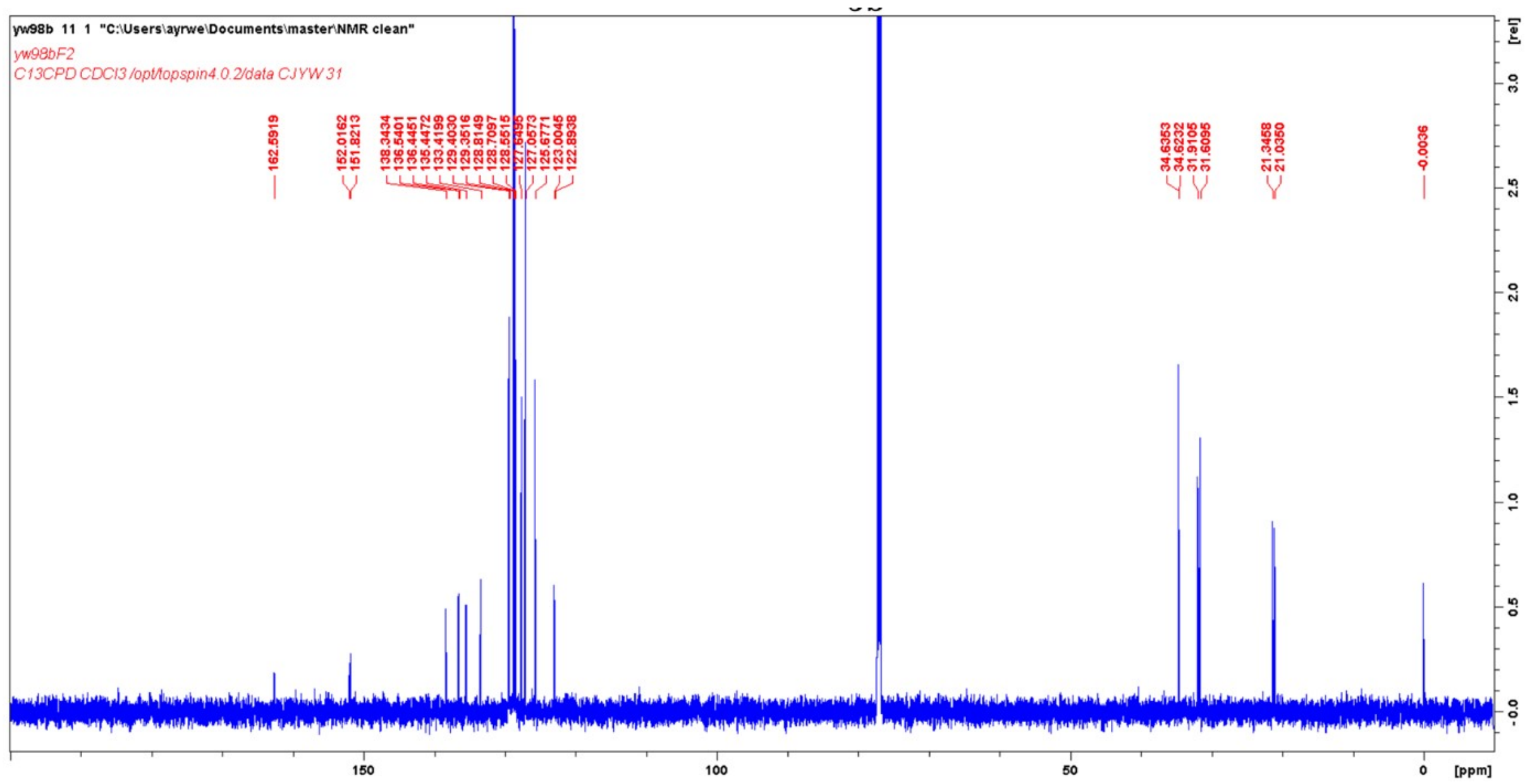

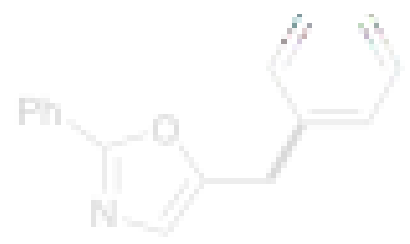

^{1}H NMR (400 MHz, $CDCl_3$), **23a**

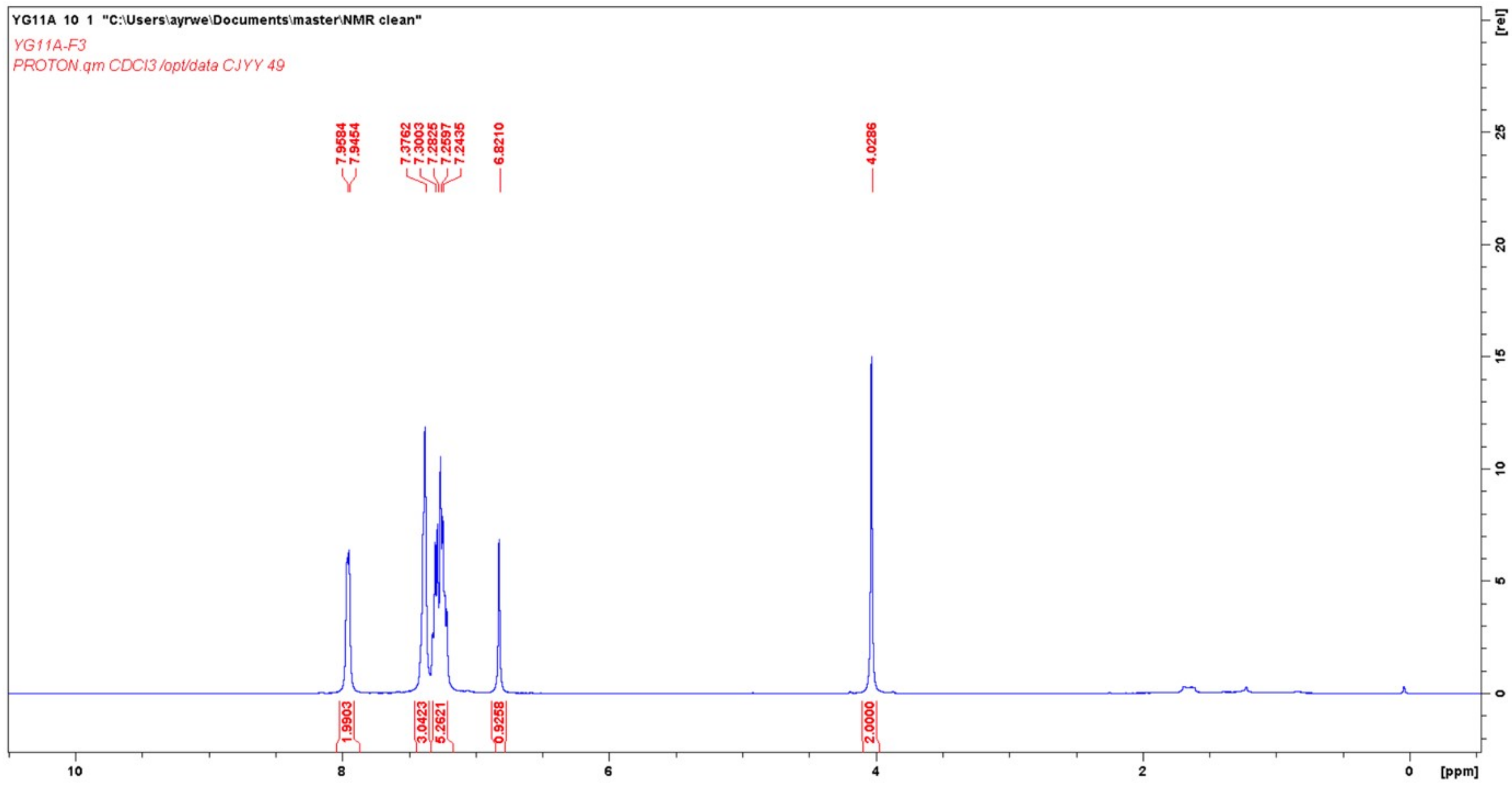

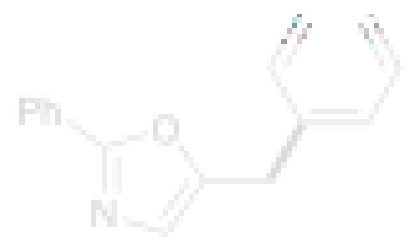

^{13}C NMR (101 MHz, $CDCl_3$), **23a**

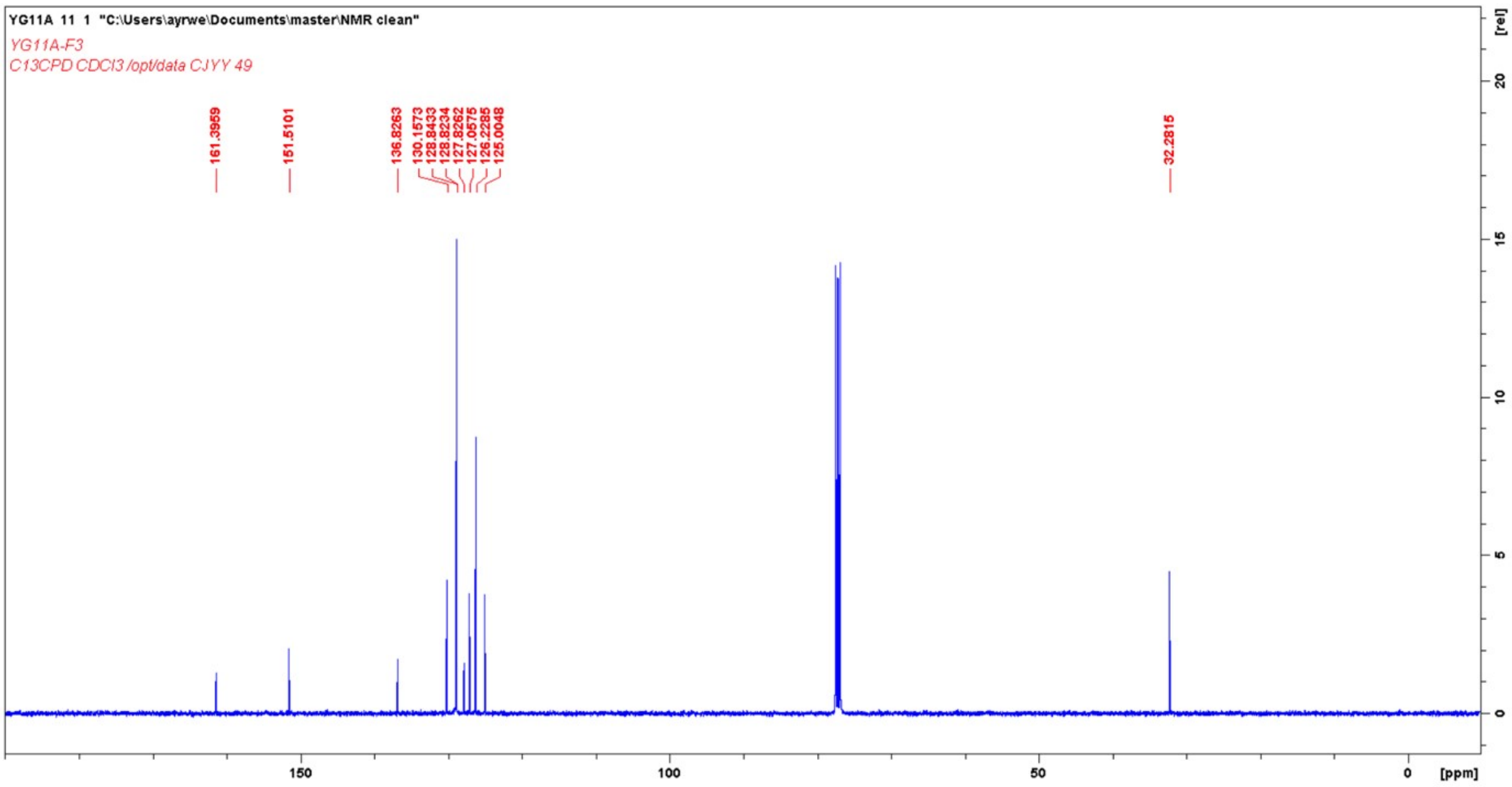

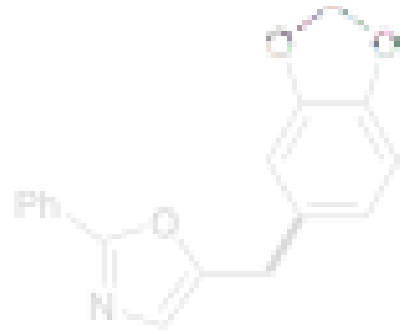

^{1}H NMR (400 MHz, $CDCl_3$), **23b**

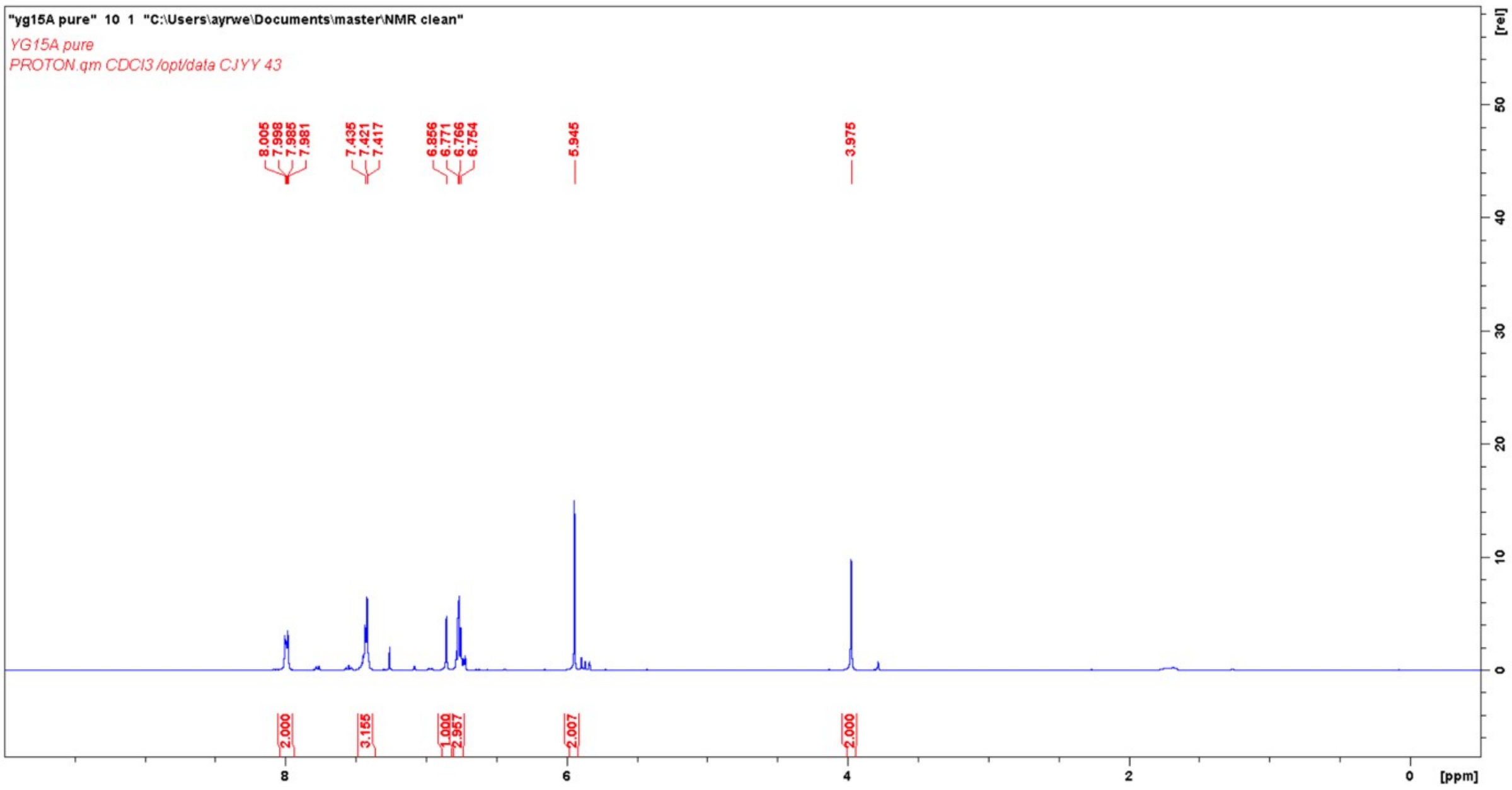

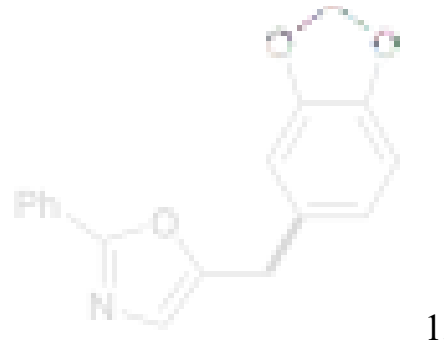

^{13}C NMR (101 MHz, $CDCl_3$), **23b**

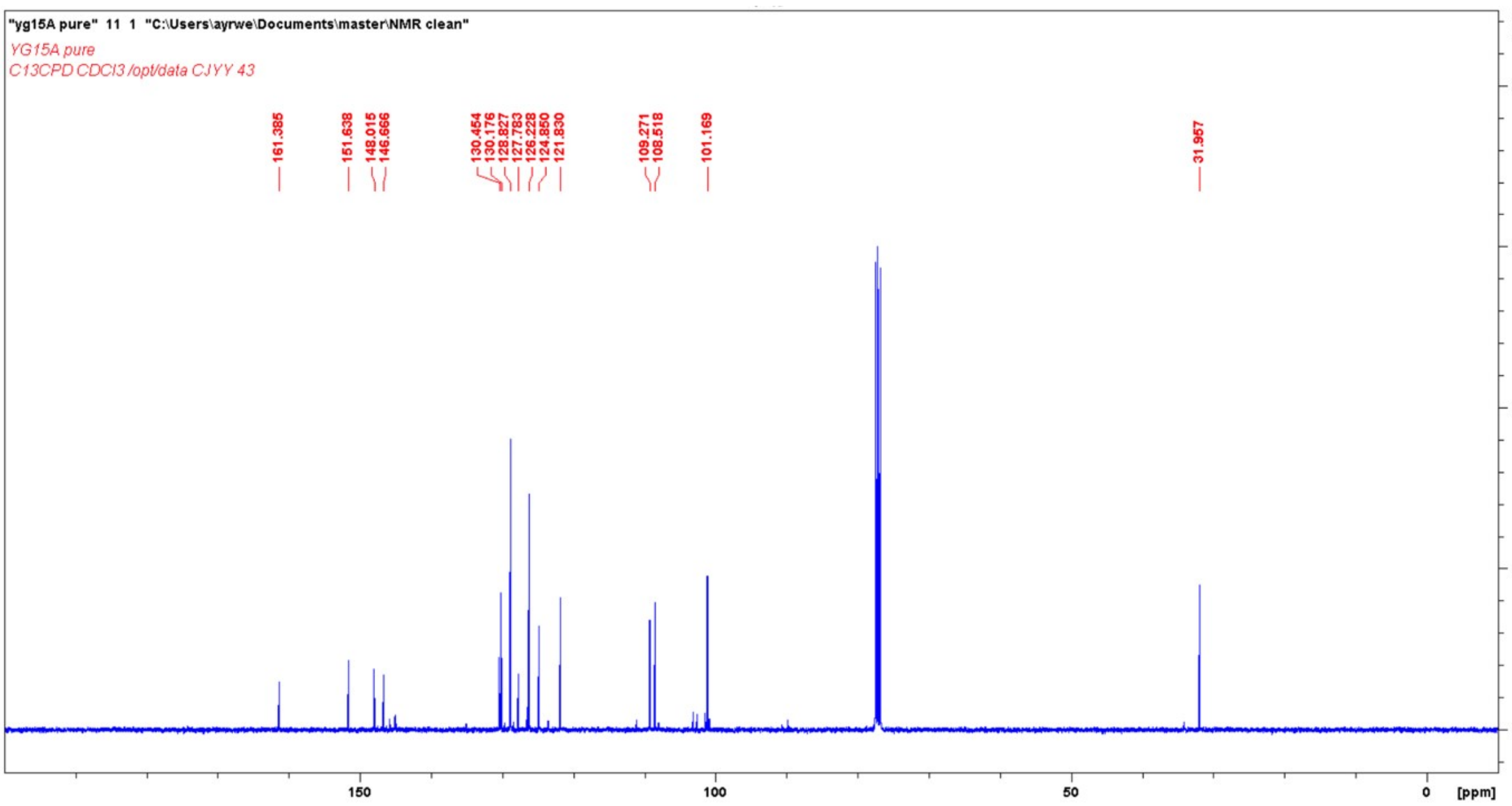

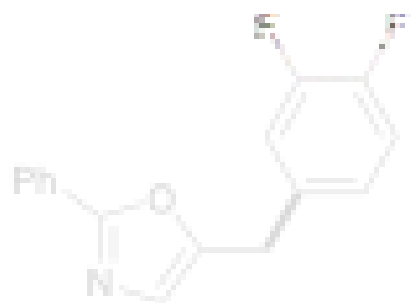

^{1}H NMR (400 MHz, $CDCl_3$), **23c**

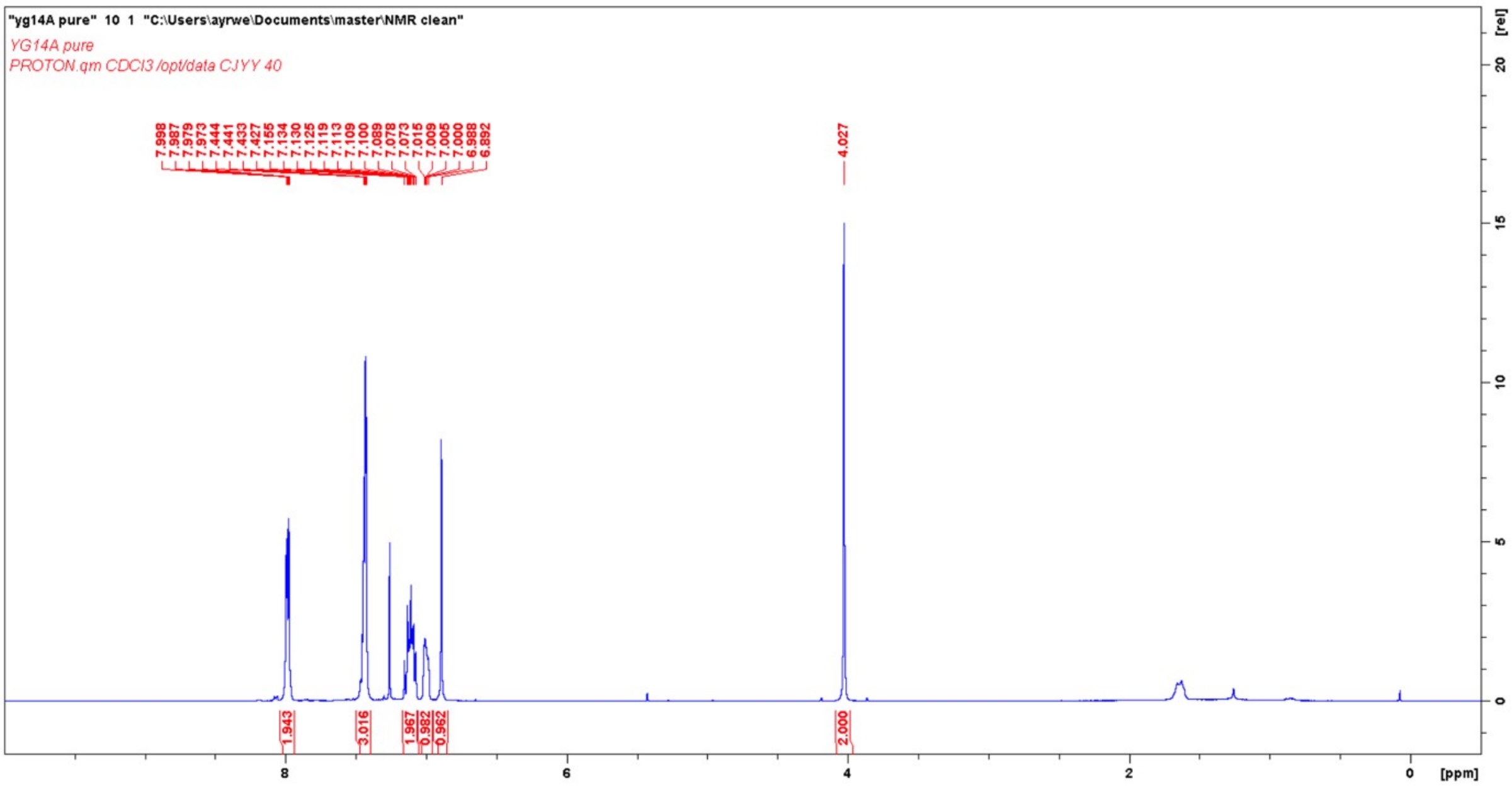

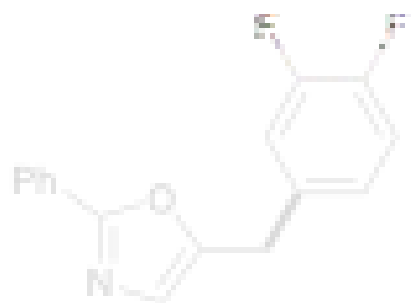

^{13}C NMR (101 MHz, $CDCl_3$), **23c**

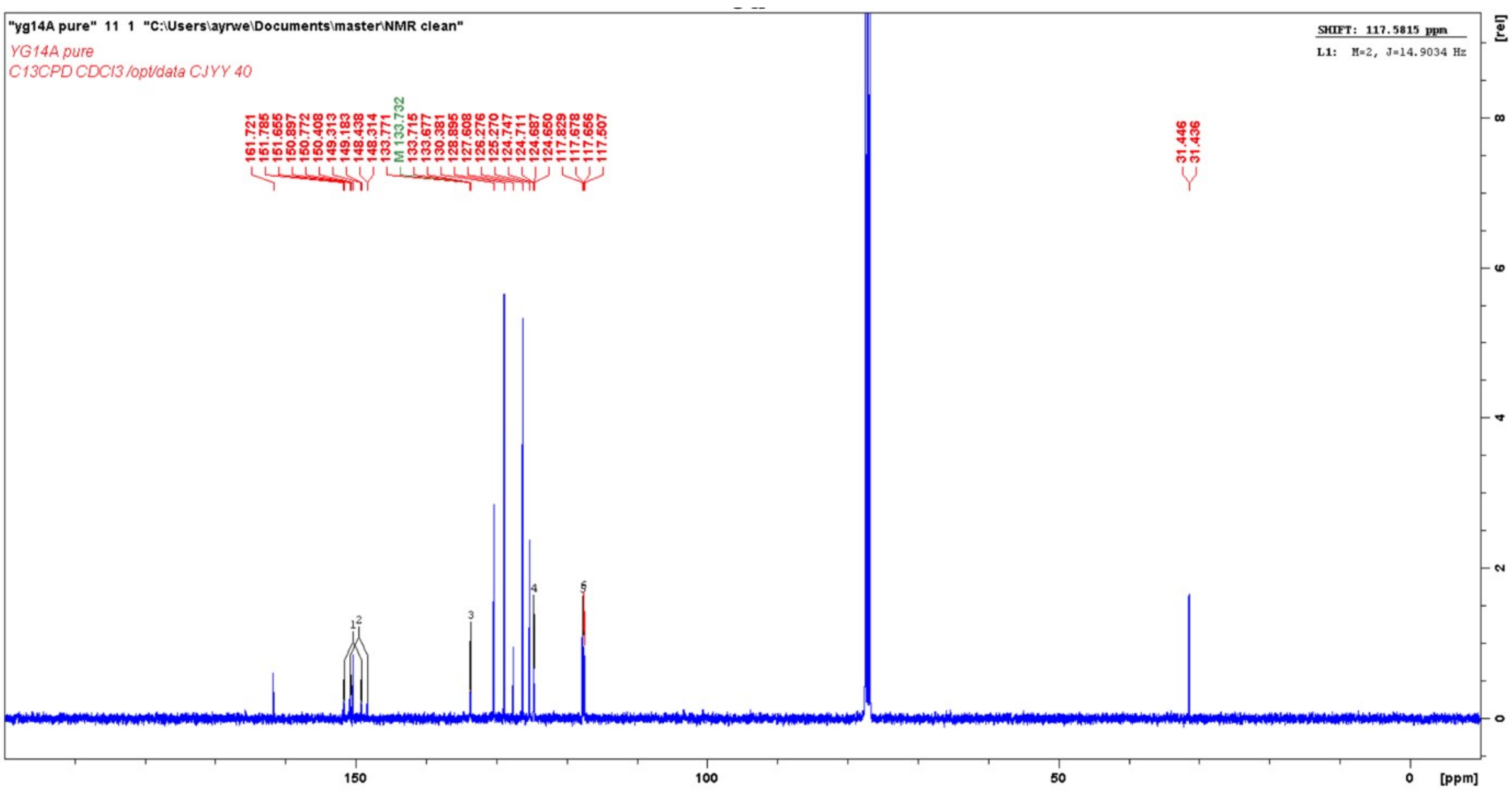

^{1}H NMR (400 MHz, $CDCl_3$), **23f**

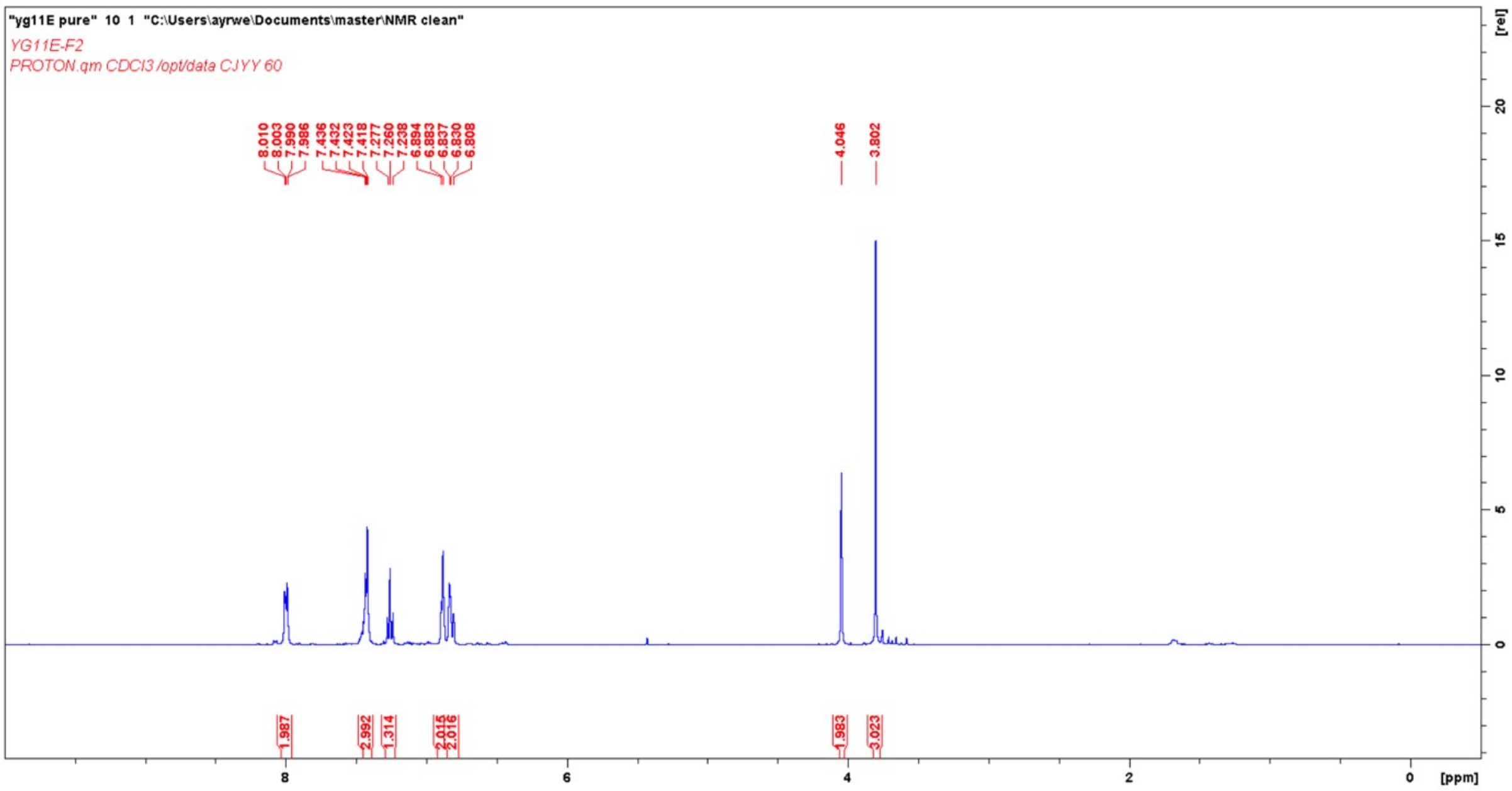

^{13}C NMR (101 MHz, $CDCl_3$), **23f**

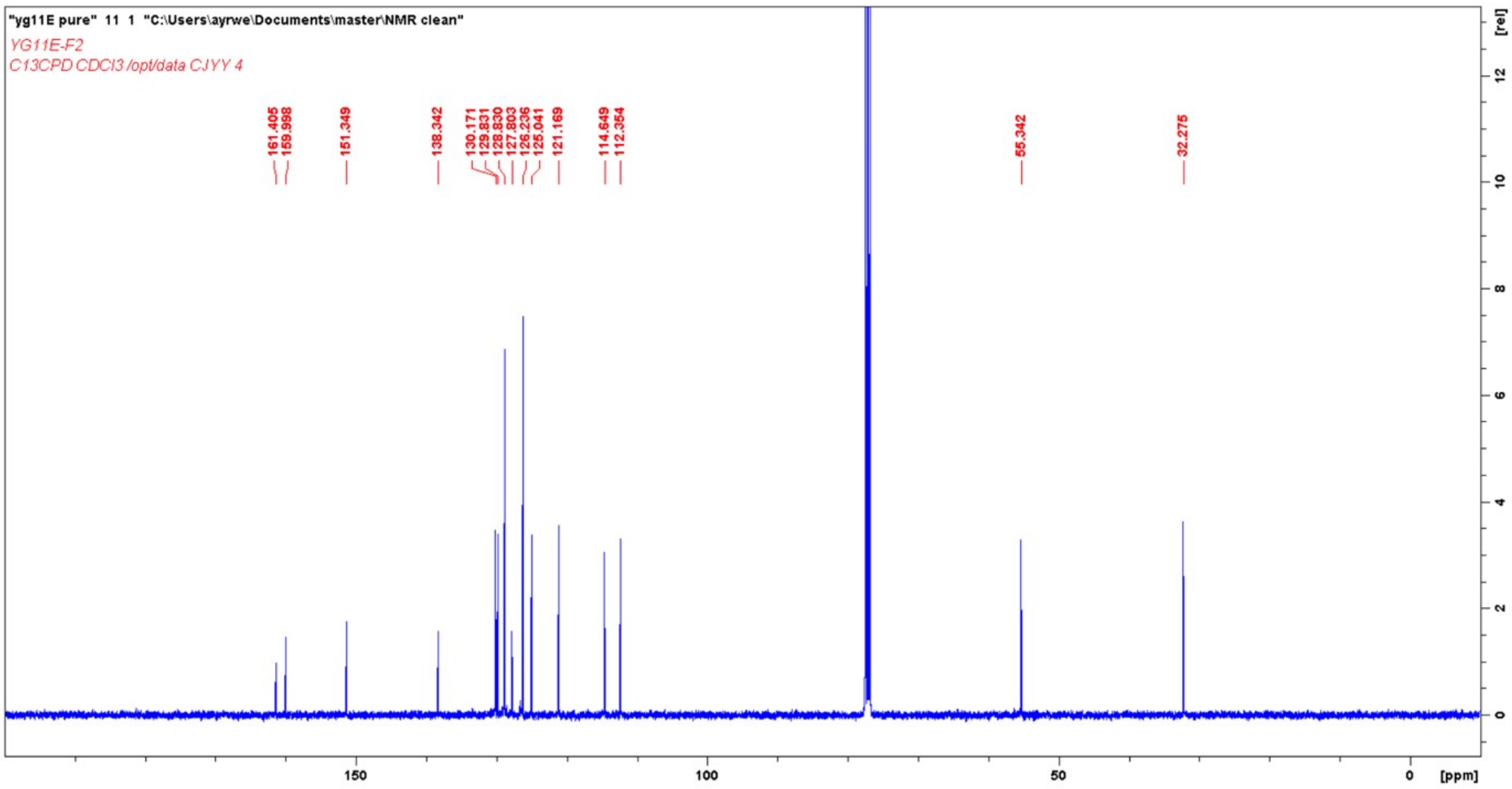

^{1}H NMR (400 MHz, $CDCl_3$), **23h & 23h'**

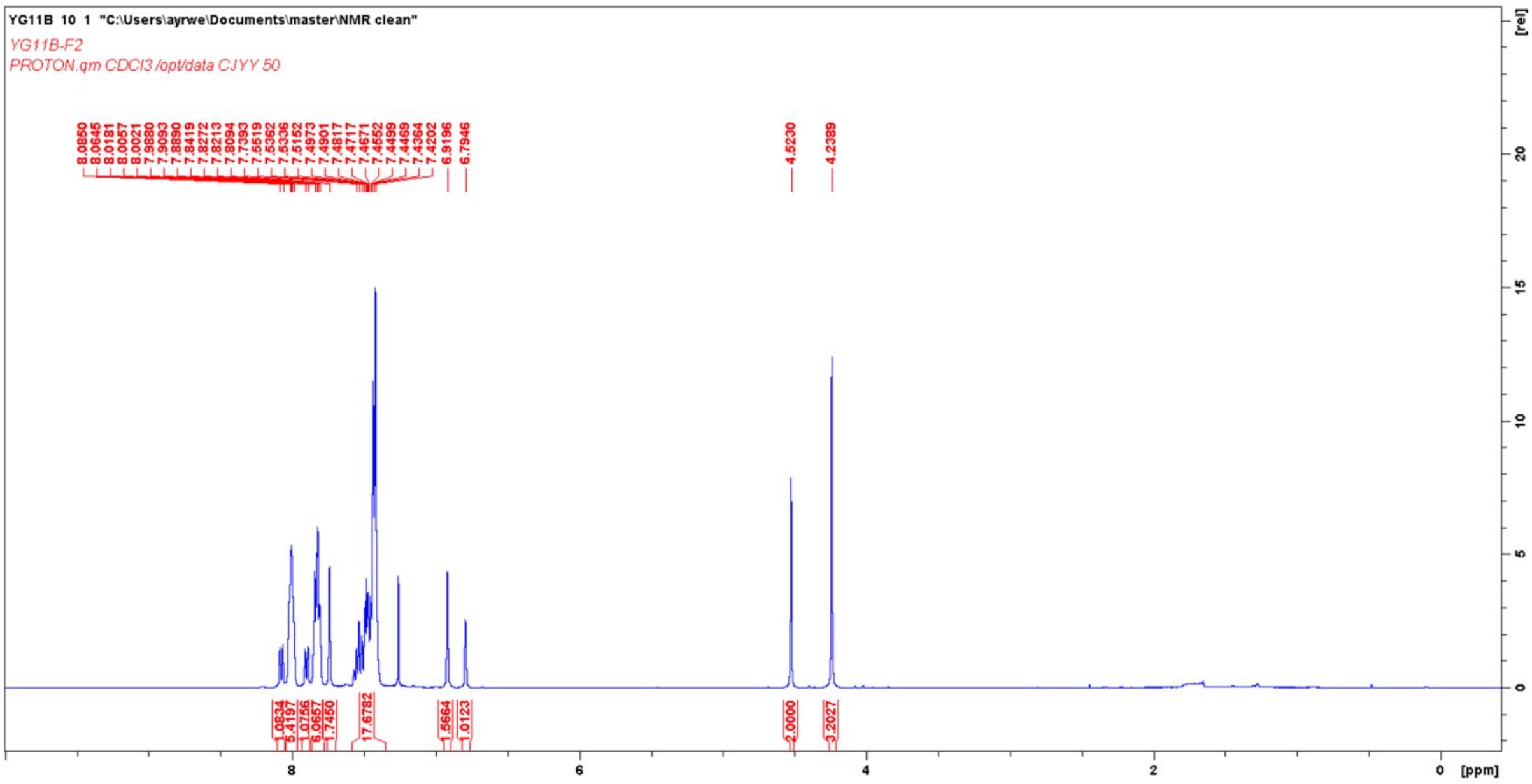

^{13}C NMR (101 MHz, $CDCl_3$), **23h & 23h'**

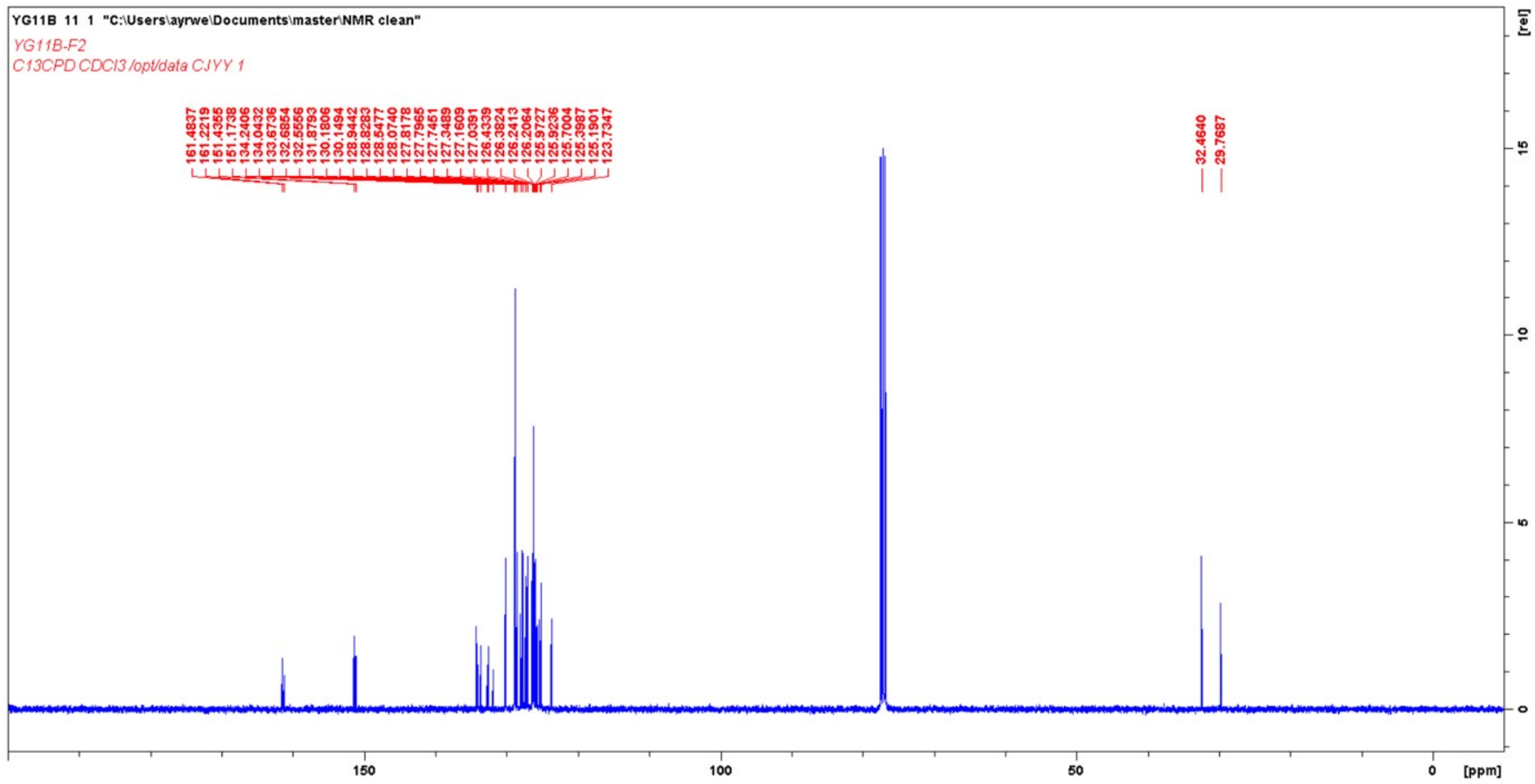

^{1}H NMR (400 MHz, $CDCl_3$), **23j & 23j’**

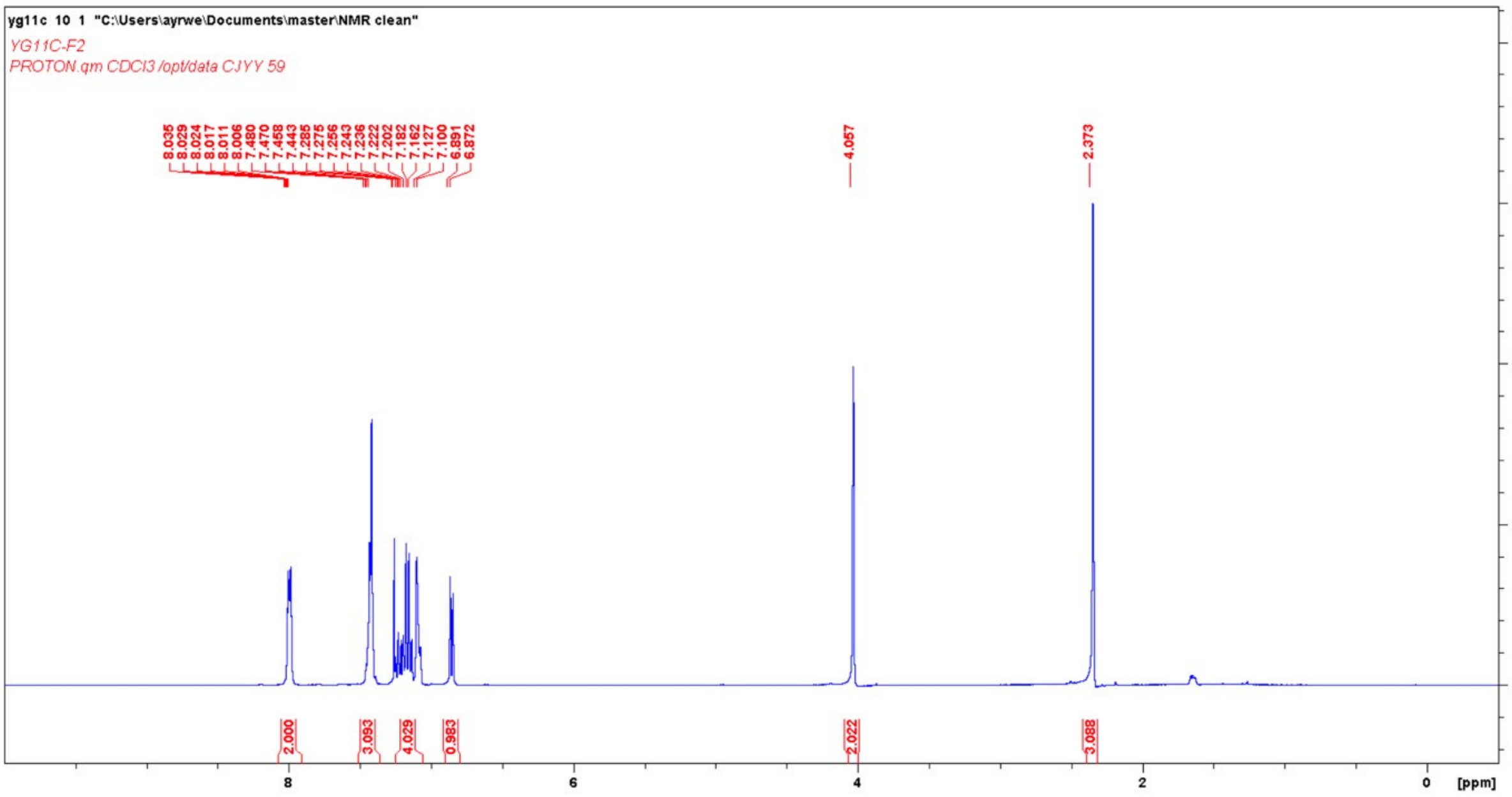

^{13}C NMR (101 MHz, $CDCl_3$), **23j & 23j'**

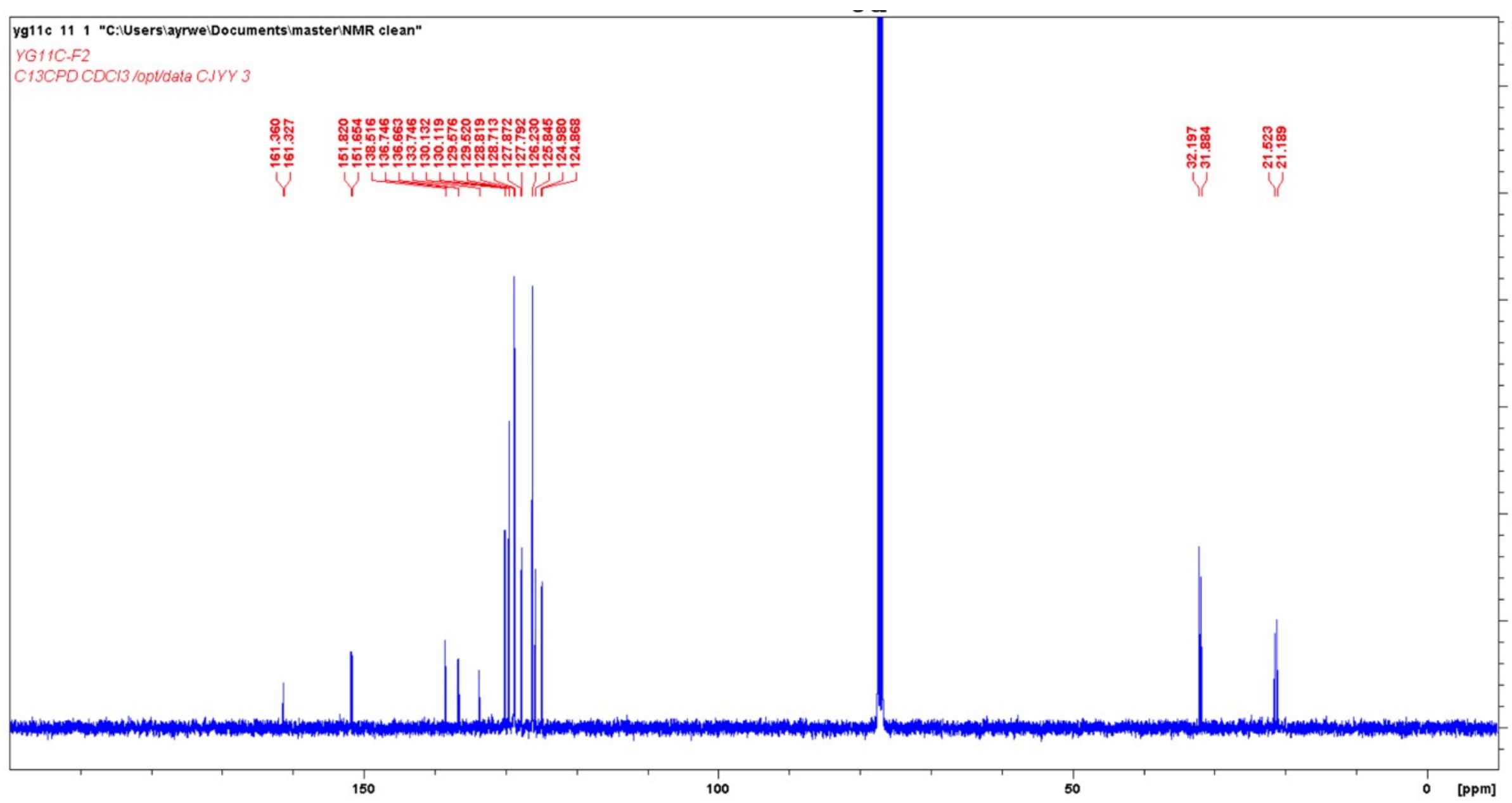

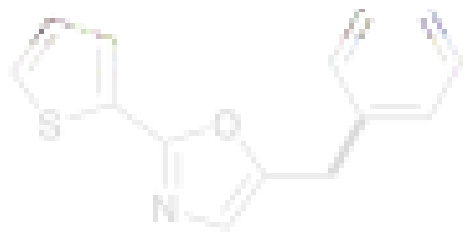

^{1}H NMR (400 MHz, $CDCl_3$), **24a**

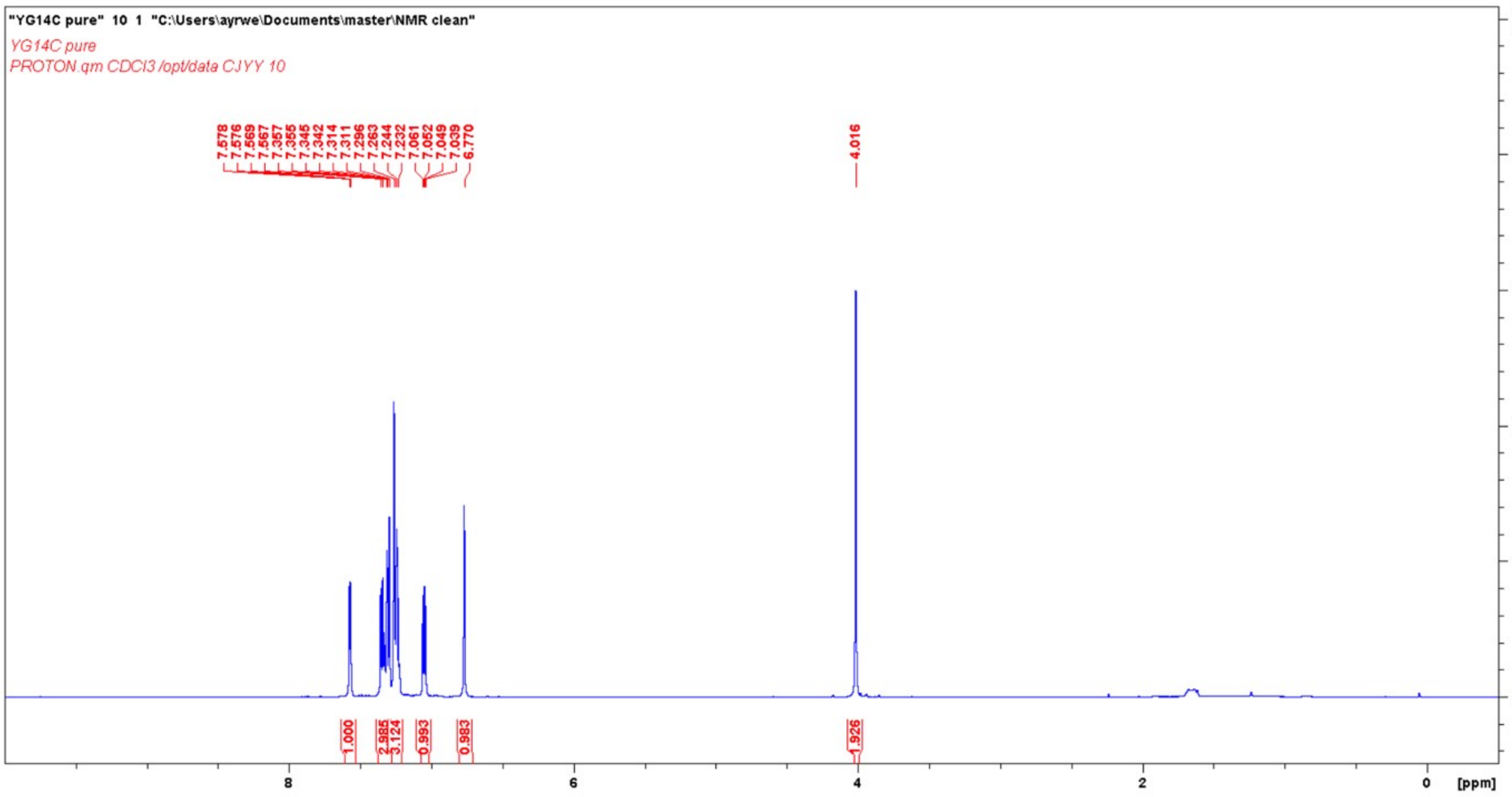

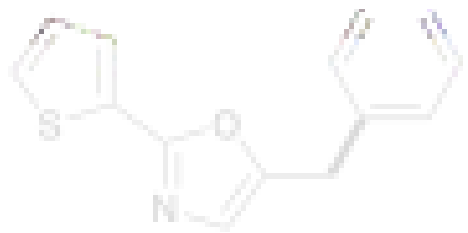

^{13}C NMR (101 MHz, $CDCl_3$), **24a**

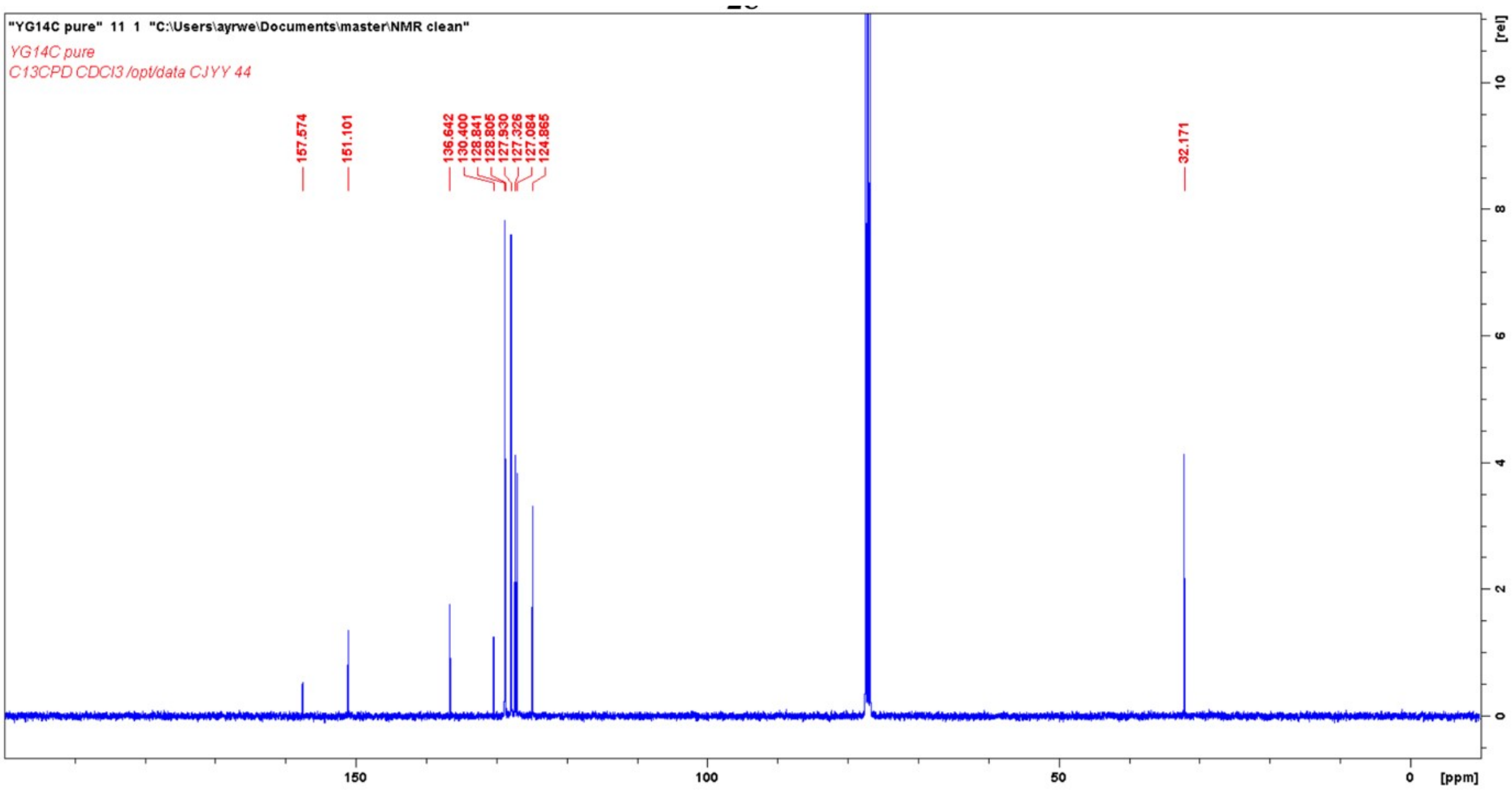

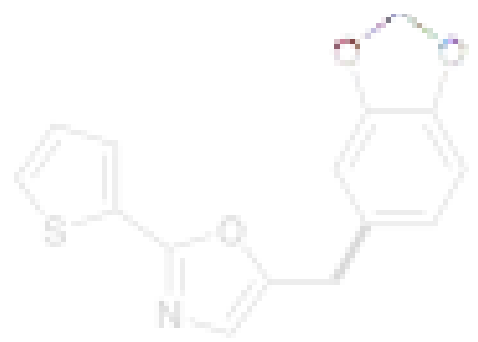

^1H NMR (400 MHz, $CDCl_3$), **24b**

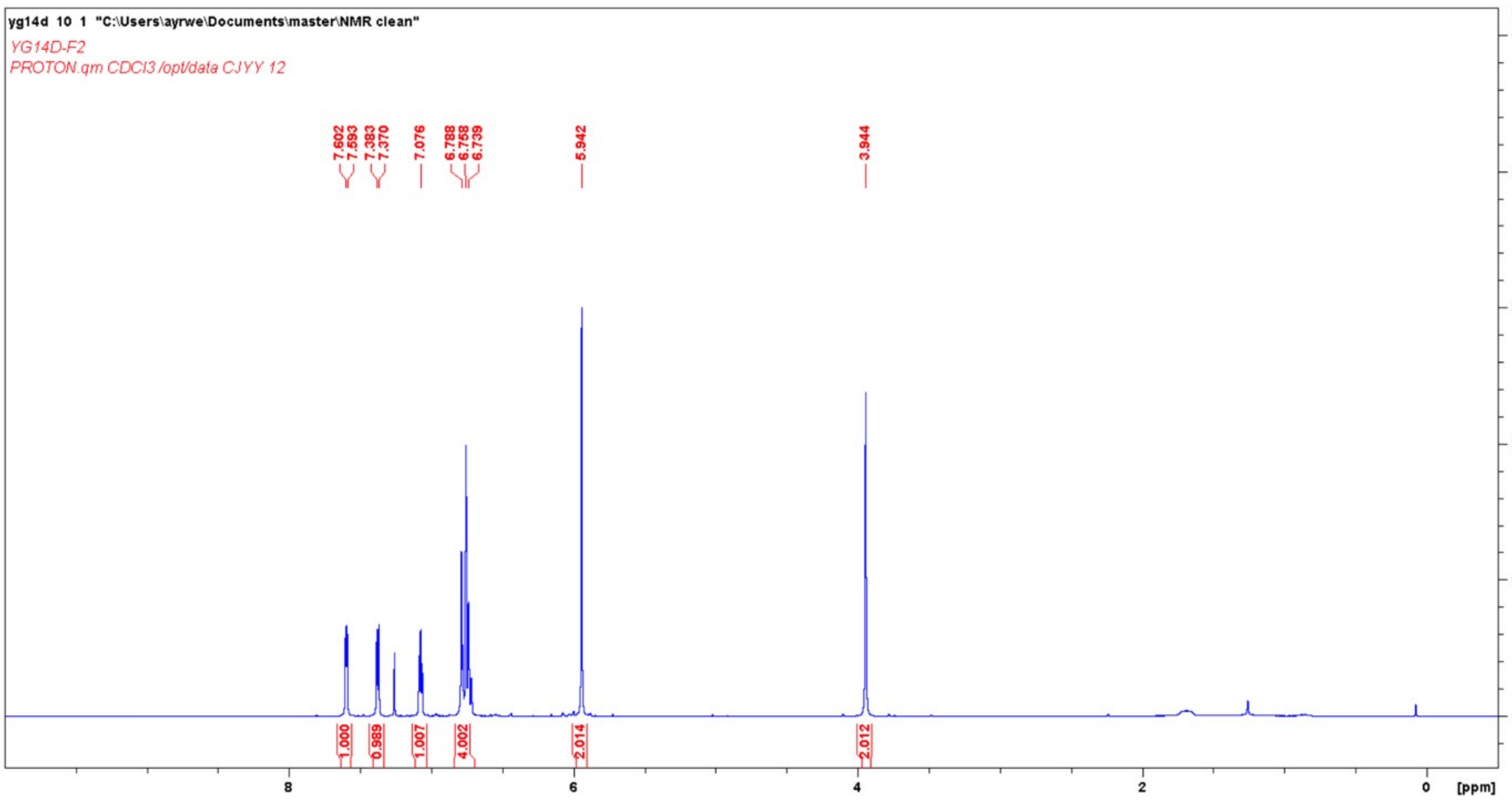

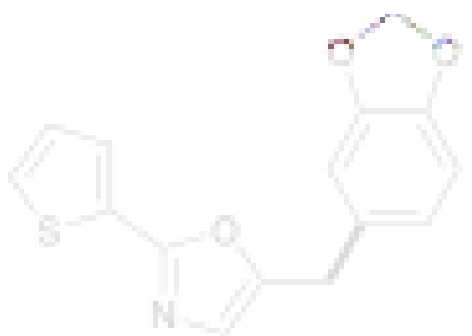

^{13}C NMR (101 MHz, $CDCl_3$), **24b**

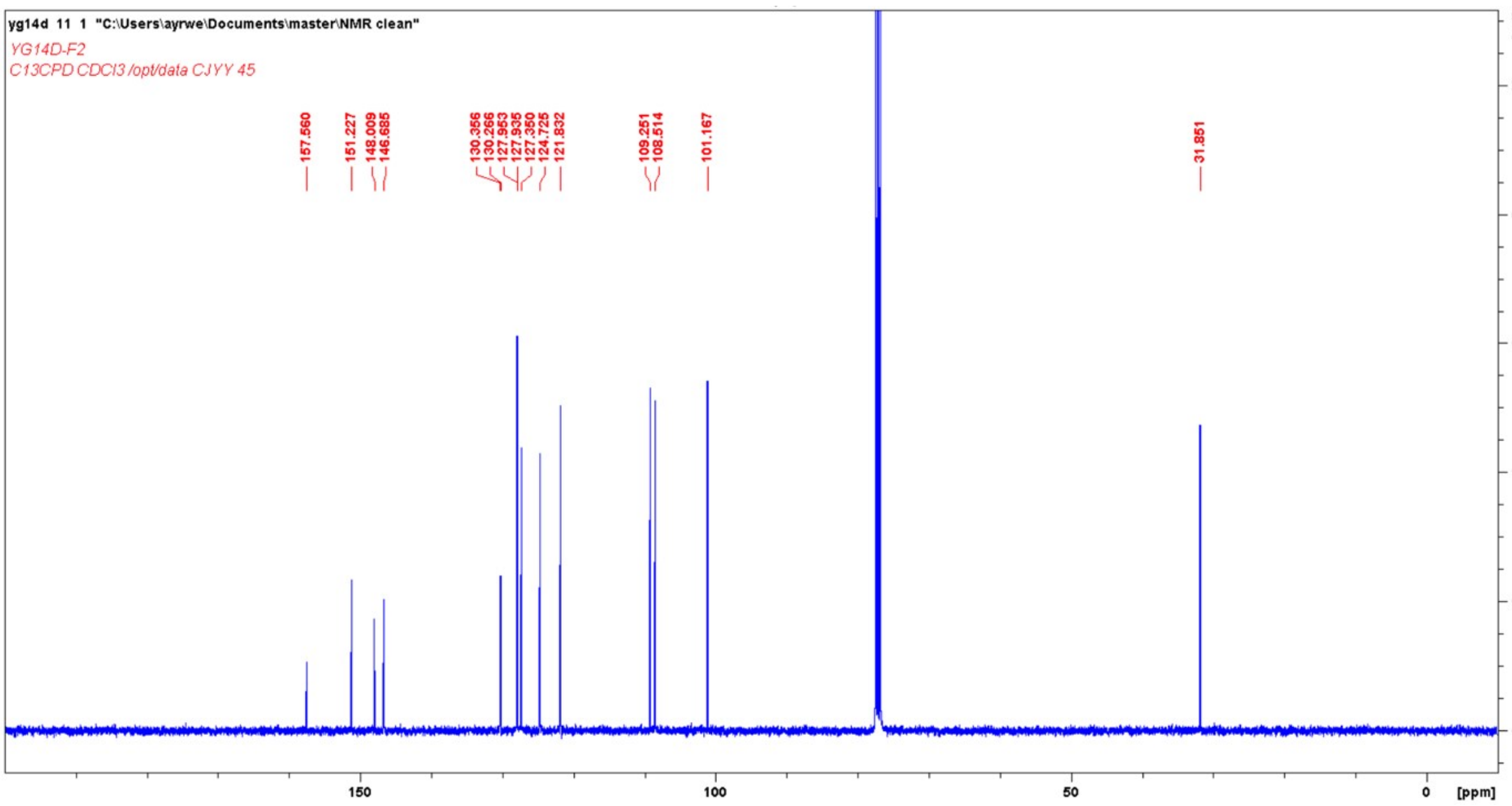

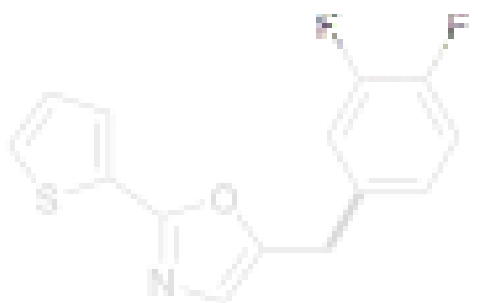

^{1}H NMR (400 MHz, $CDCl_3$), **24c**

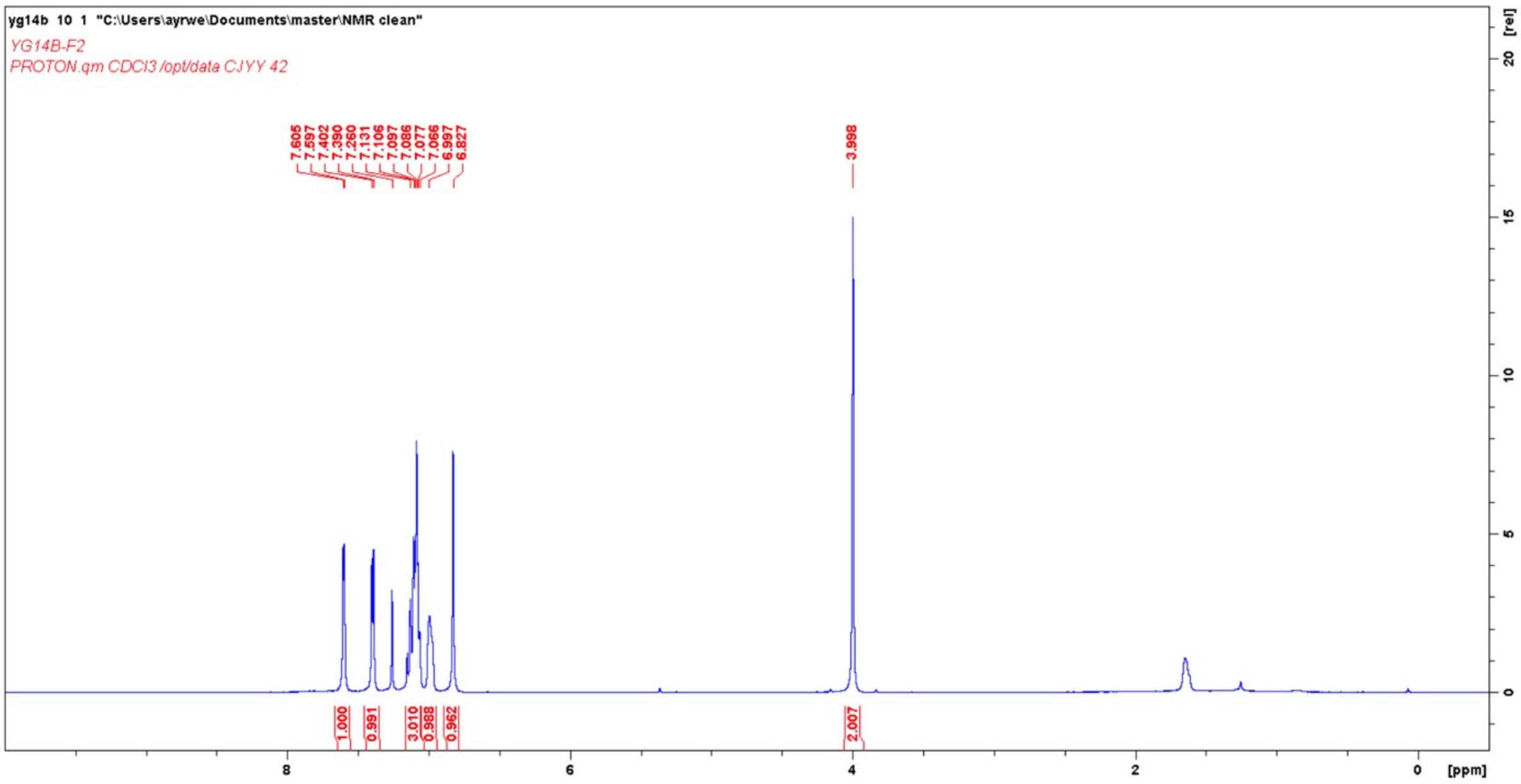

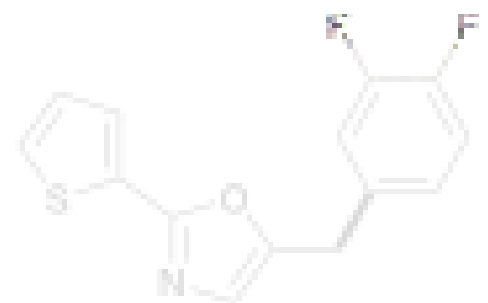

^{13}C NMR (101 MHz, $CDCl_3$), **24c**

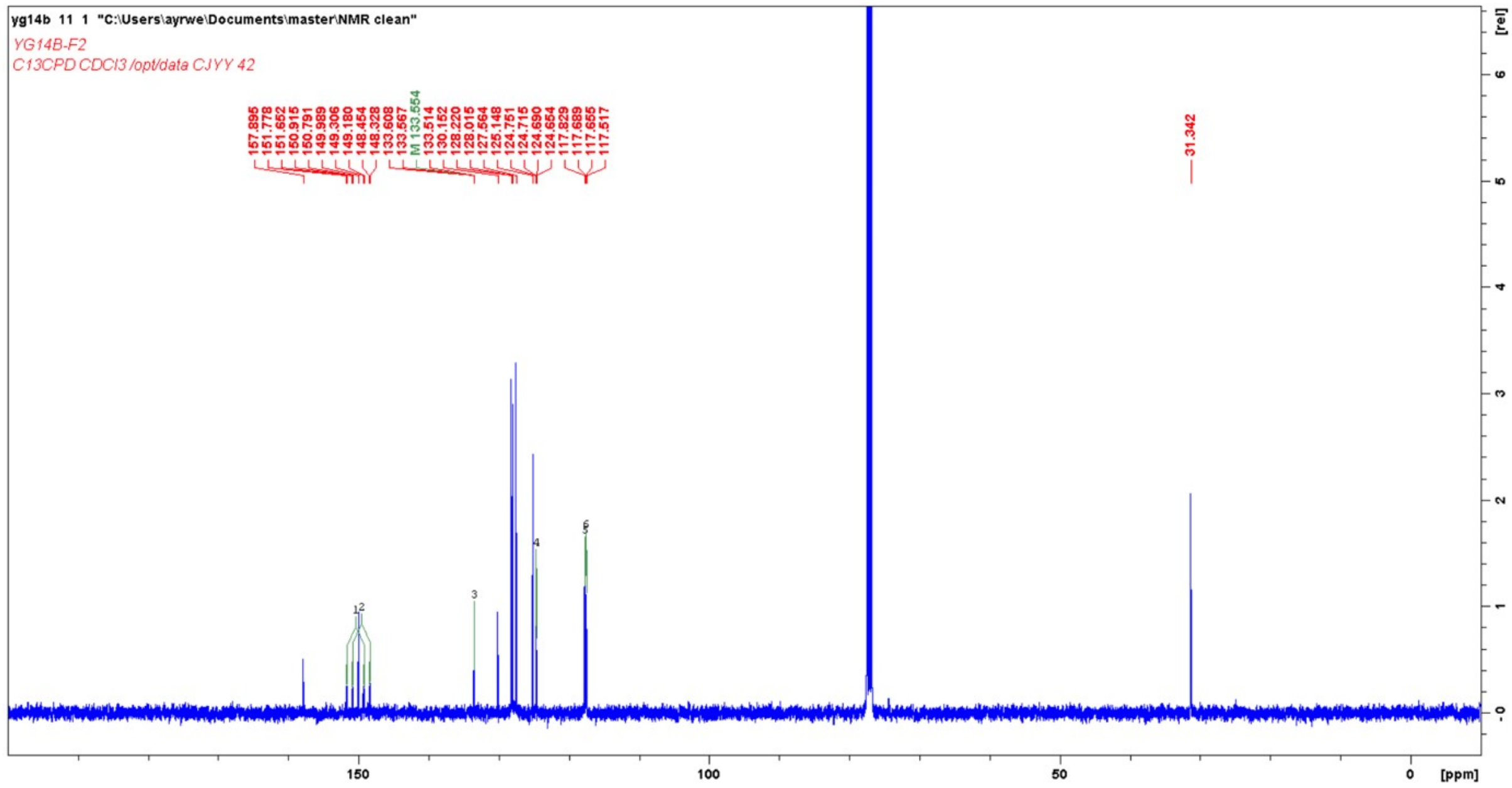

^1H NMR (400 MHz, $CDCl_3$), **24f**

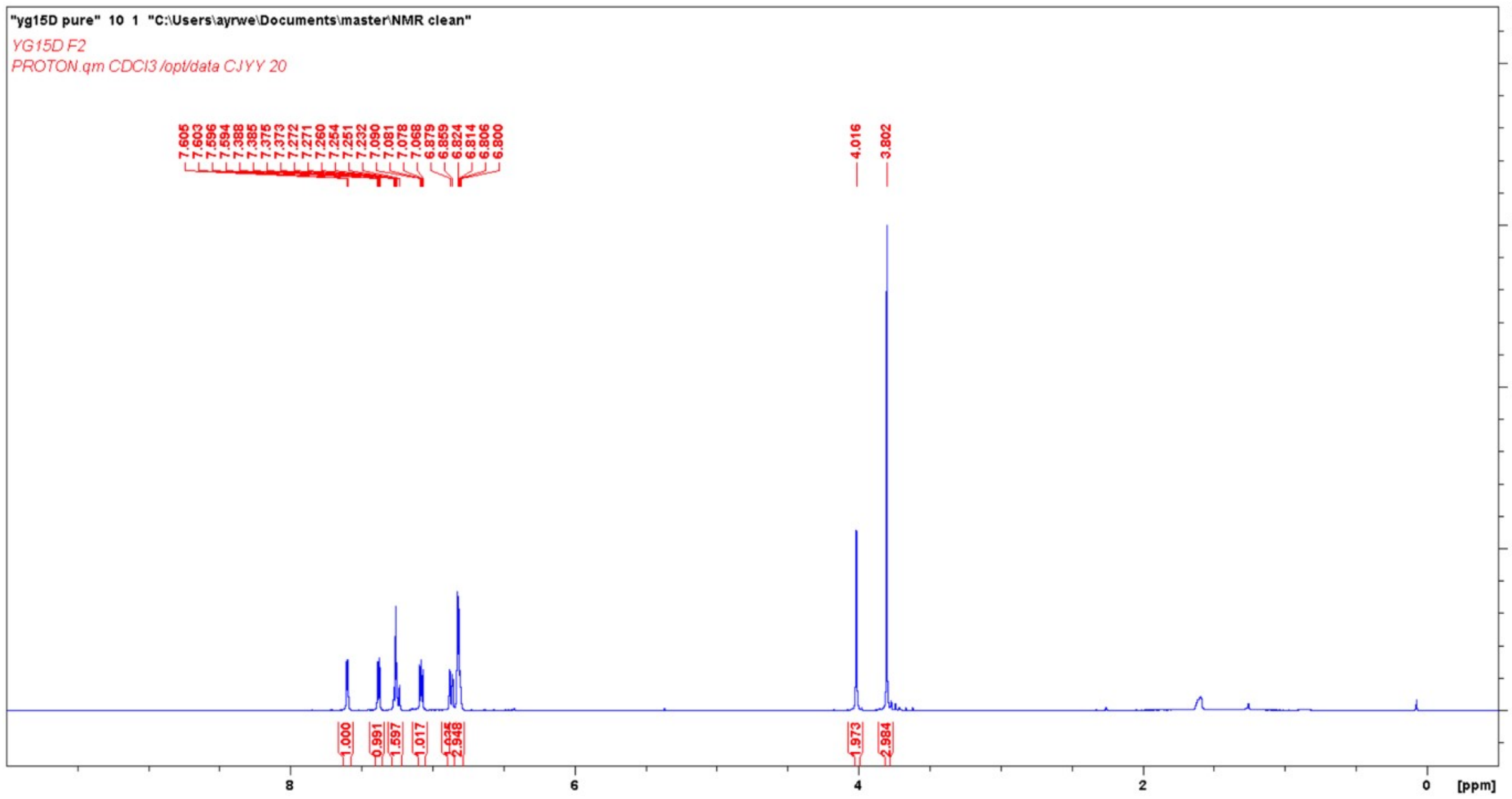

^{13}C NMR (101 MHz, $CDCl_3$), **24f**

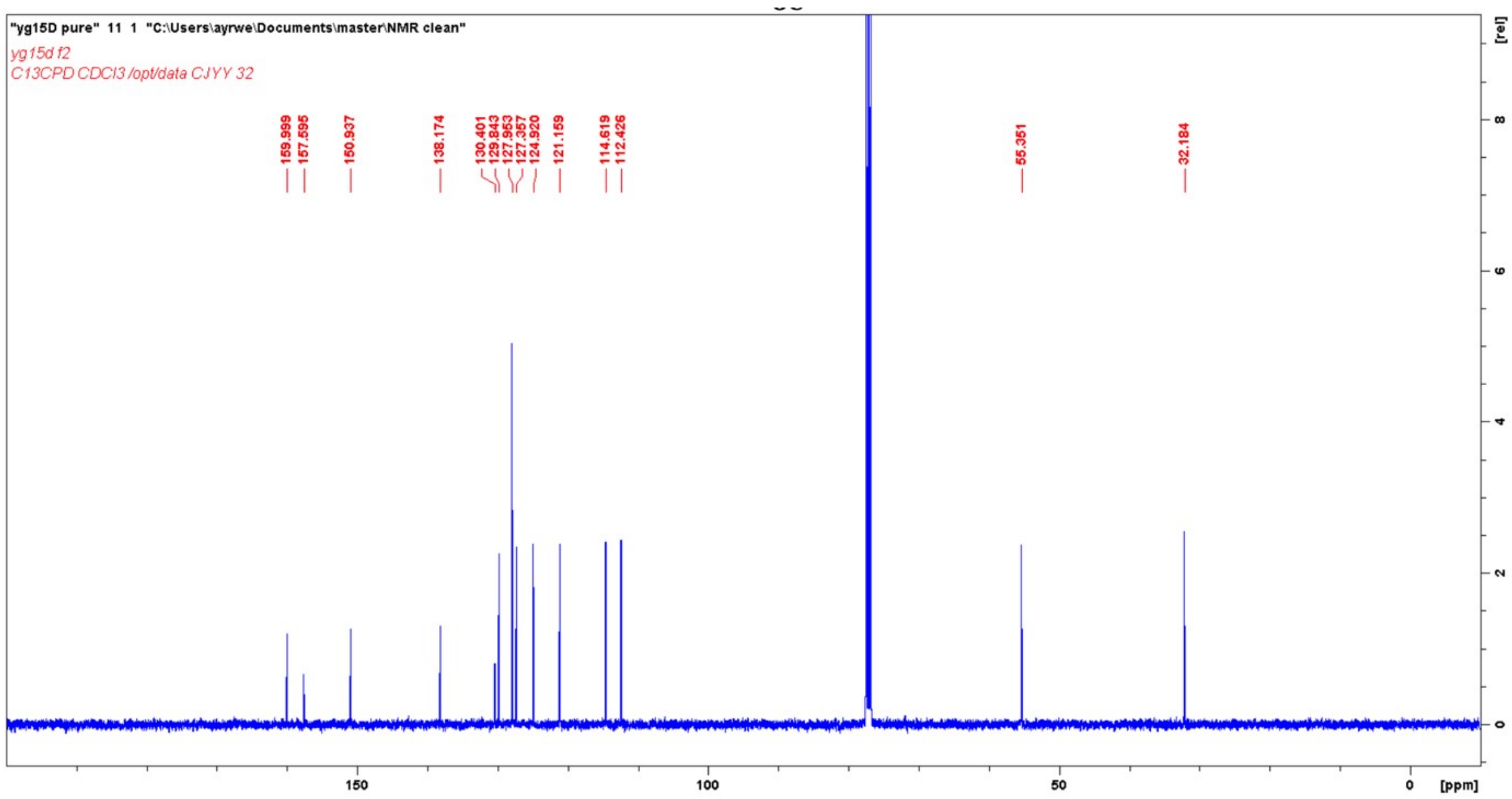

^{1}H NMR (400 MHz, $CDCl_3$), **24h & 24h’**

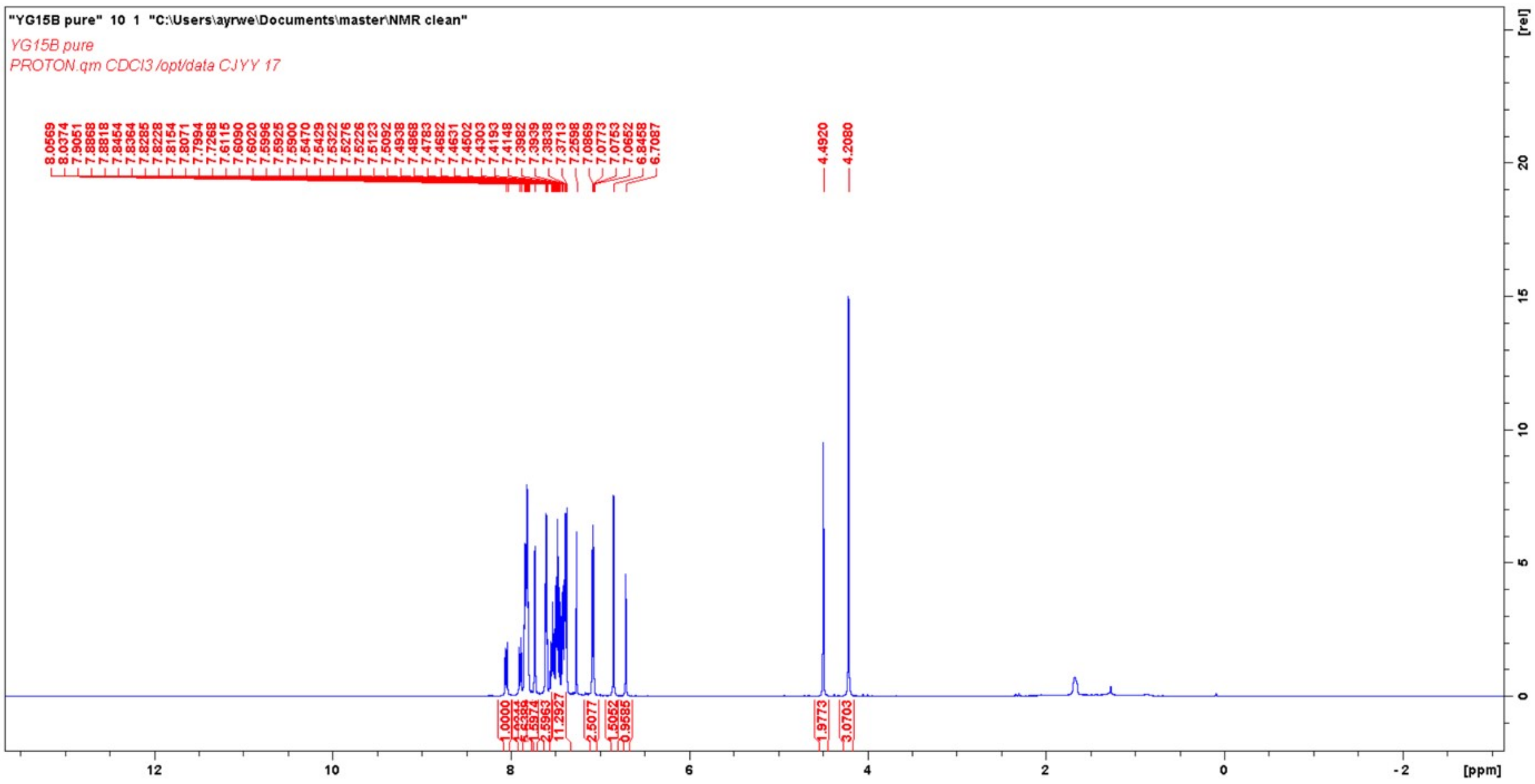

^{13}C NMR (101 MHz, $CDCl_3$), **24h & 24h'**

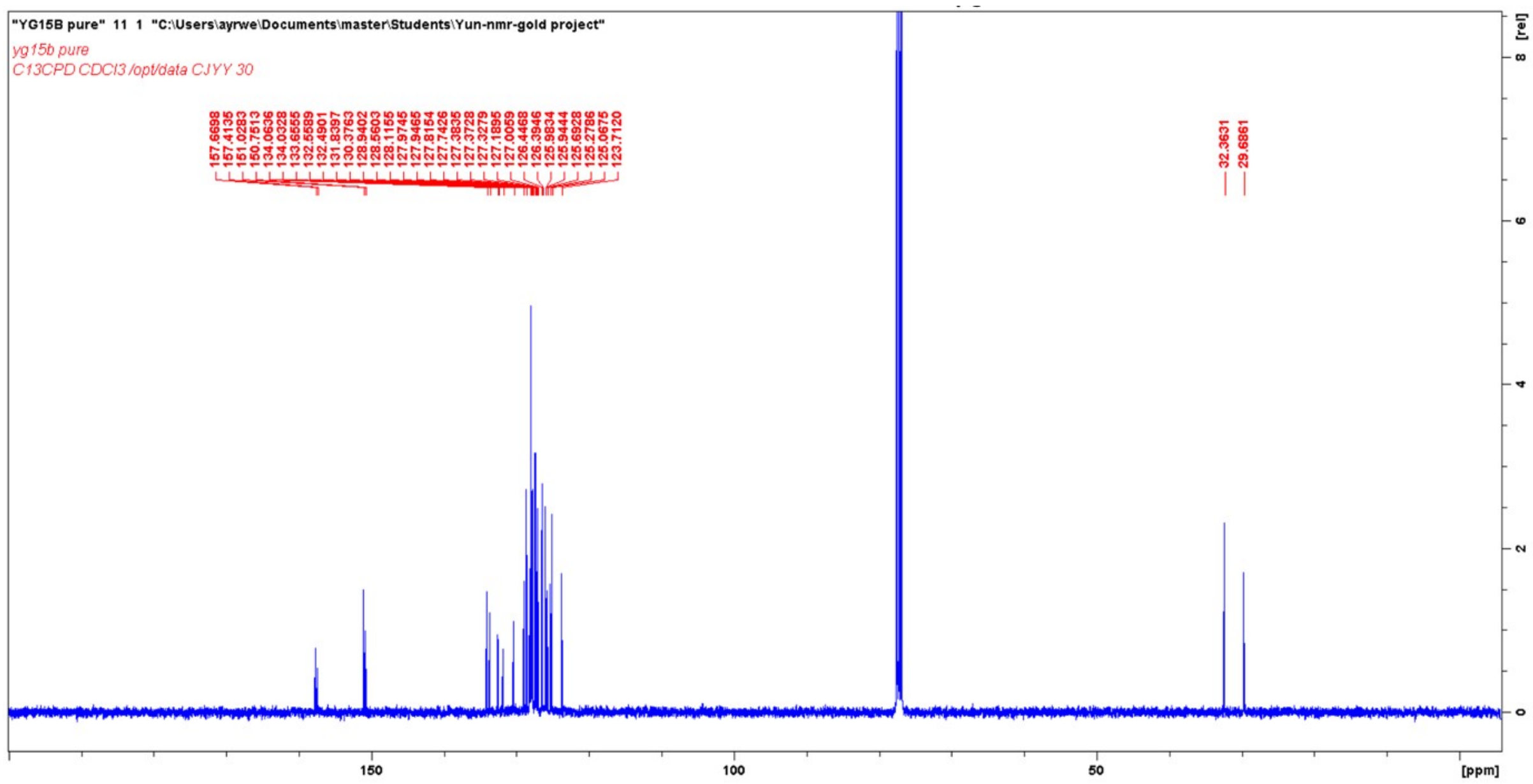

^1H NMR (400 MHz, $CDCl_3$), **24j & 24j'**

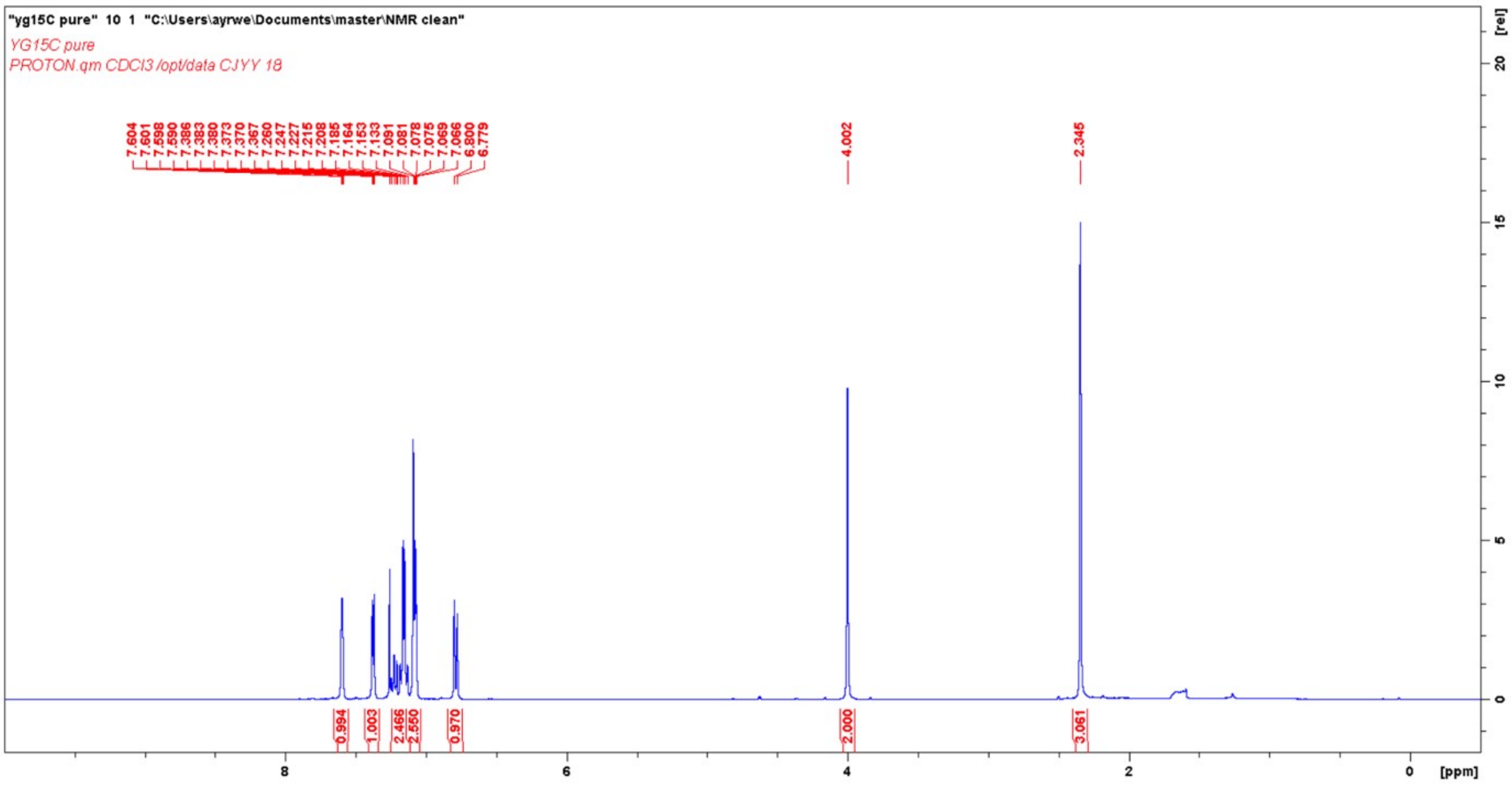

^{13}C NMR (101 MHz, $CDCl_3$), **24j & 24j'**

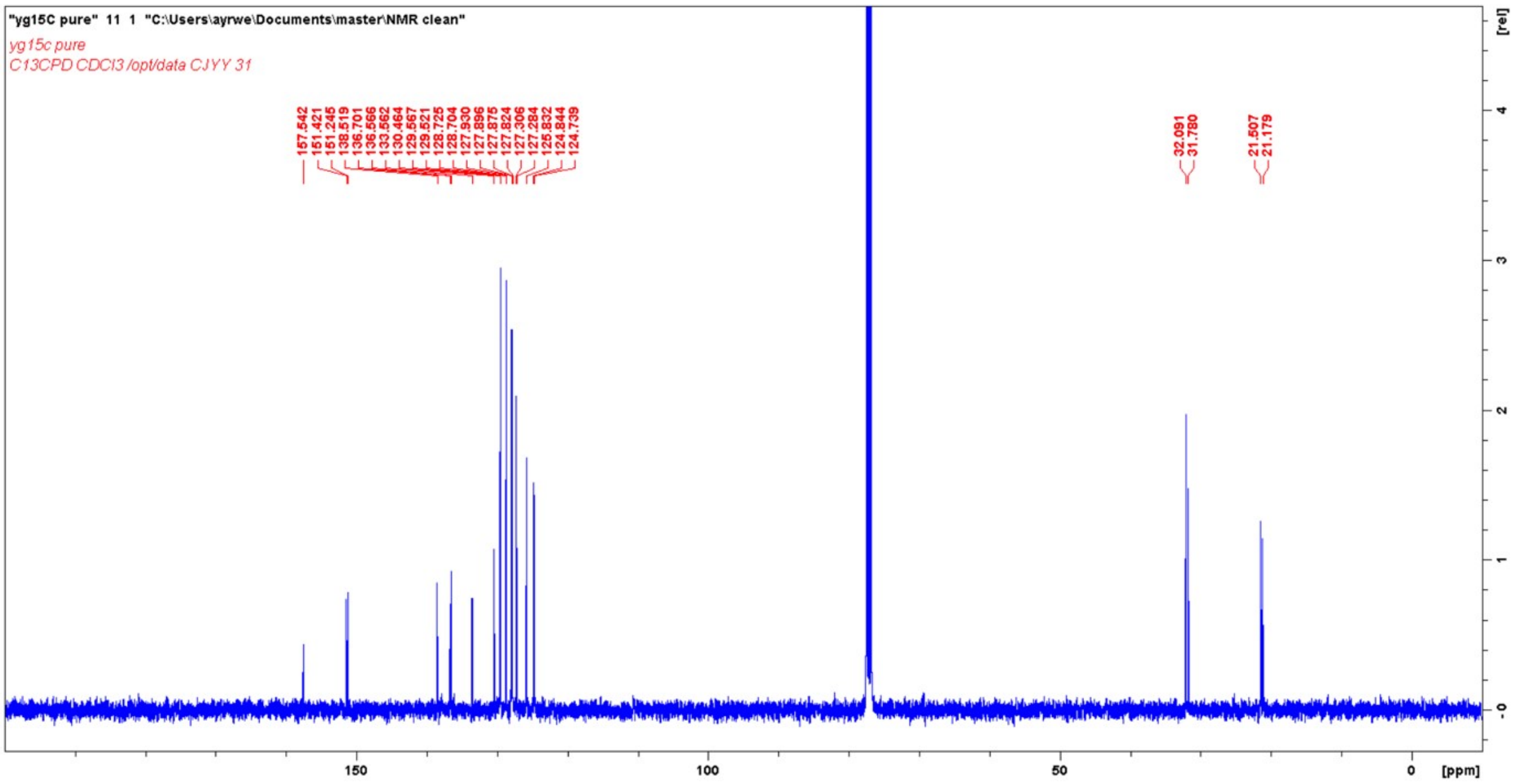

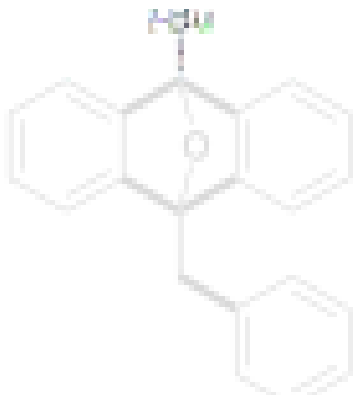

^{1}H NMR (400 MHz, $CDCl_3$), **16a**

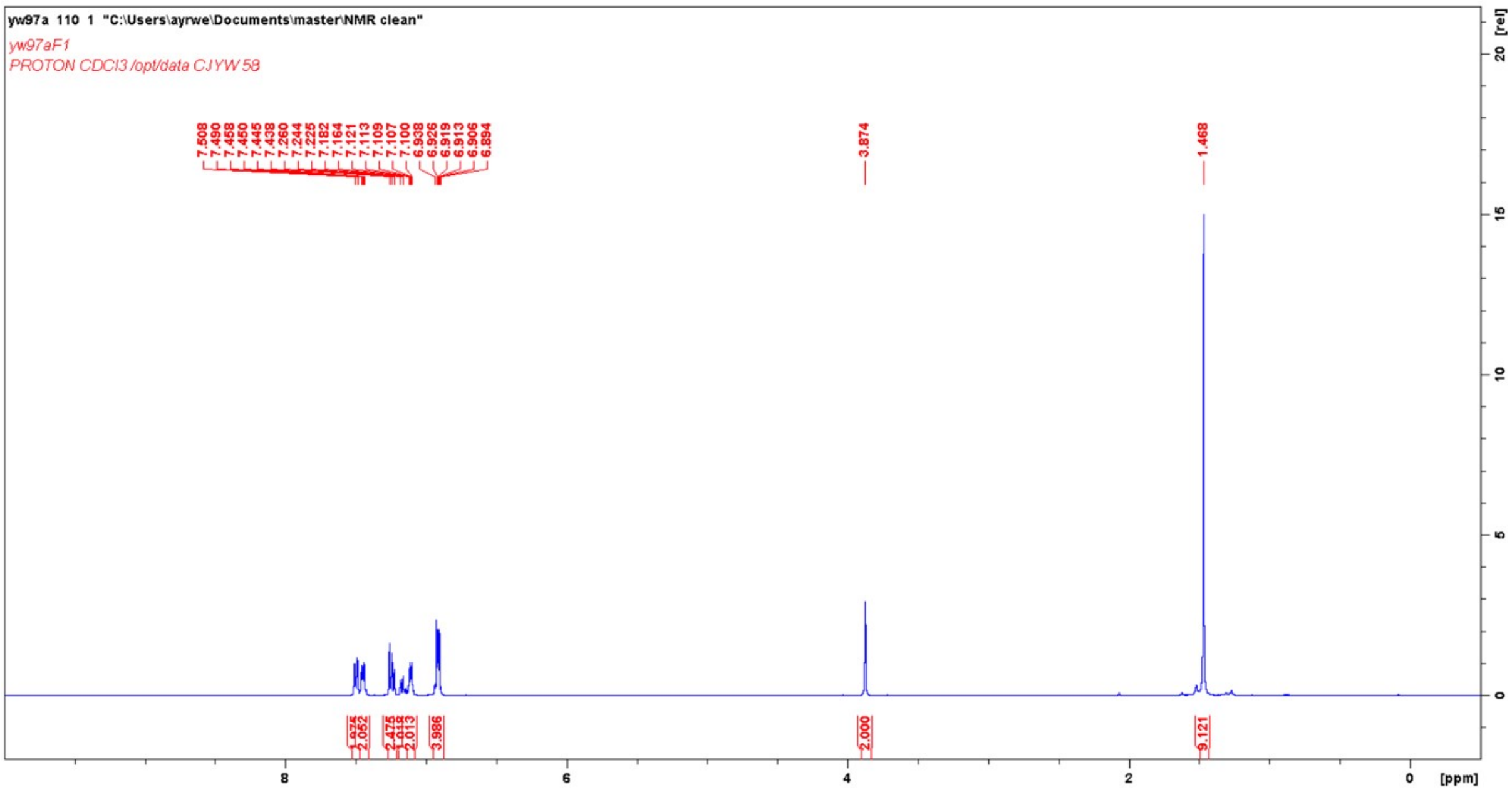

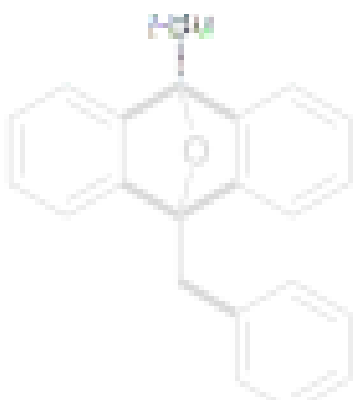

^{13}C NMR (101 MHz, $CDCl_3$), **16a**

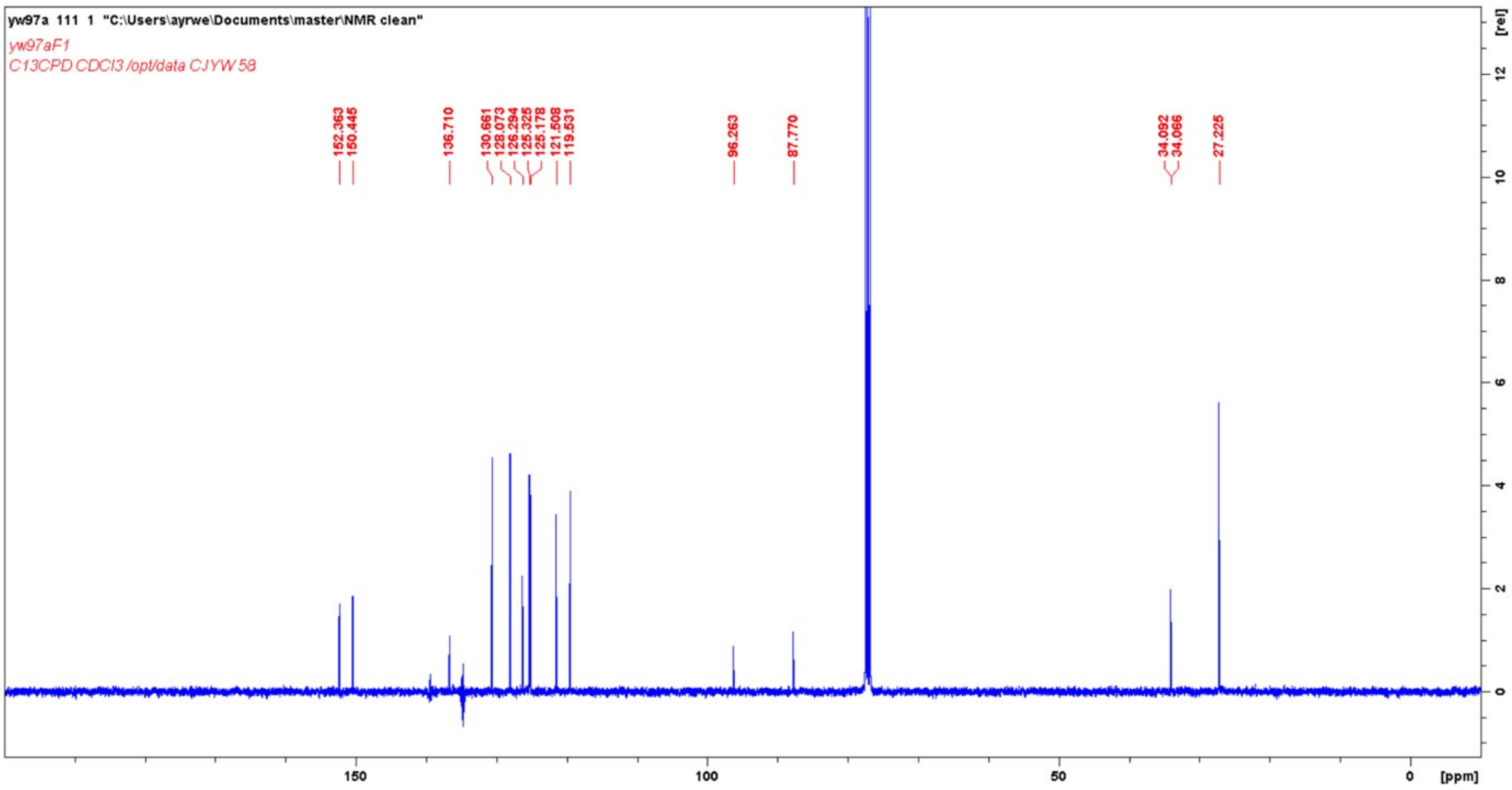

^1H NMR (400 MHz, $CDCl_3$), **16b**

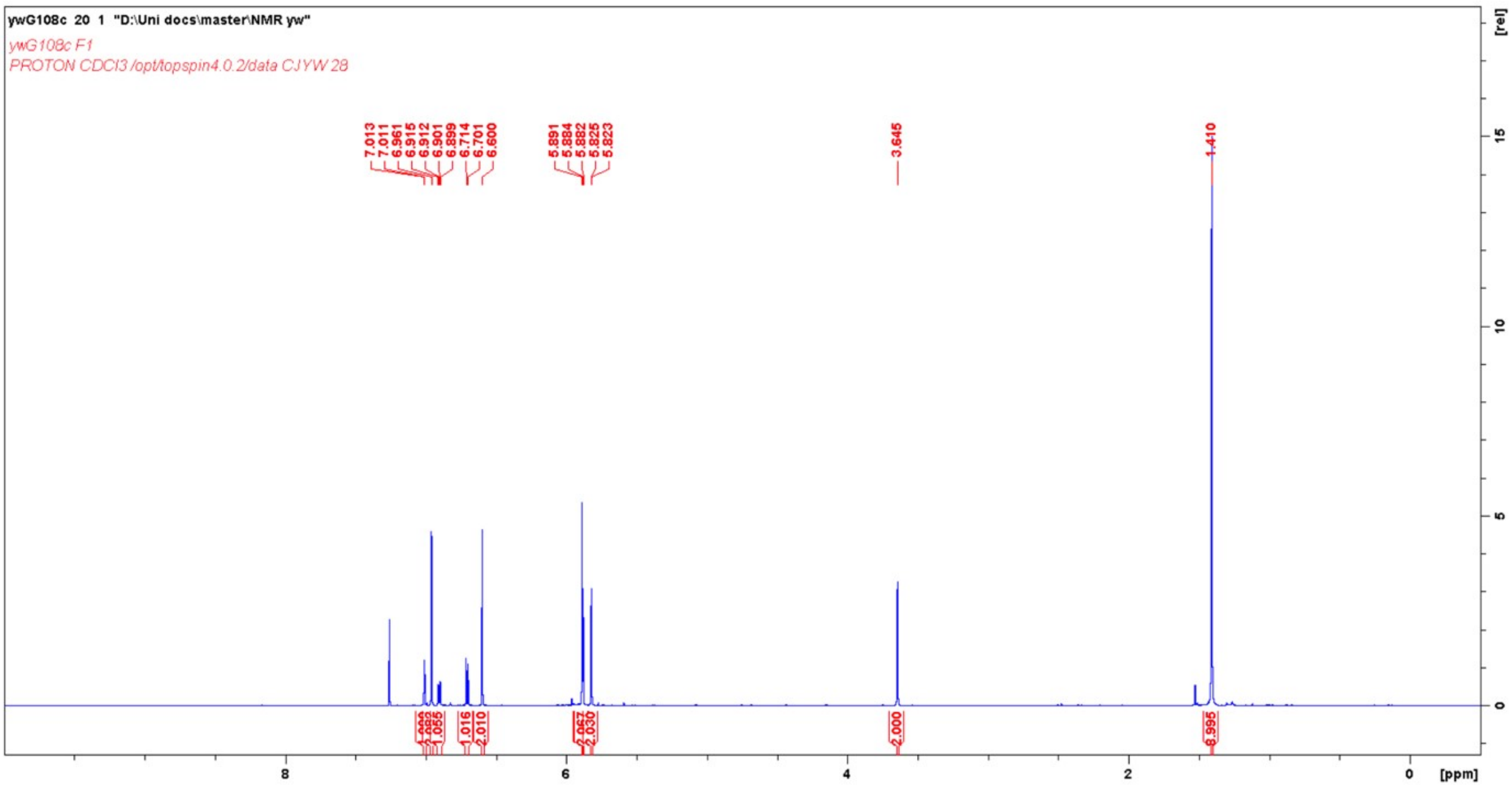

^{13}C NMR (101 MHz, $CDCl_3$), **16b**

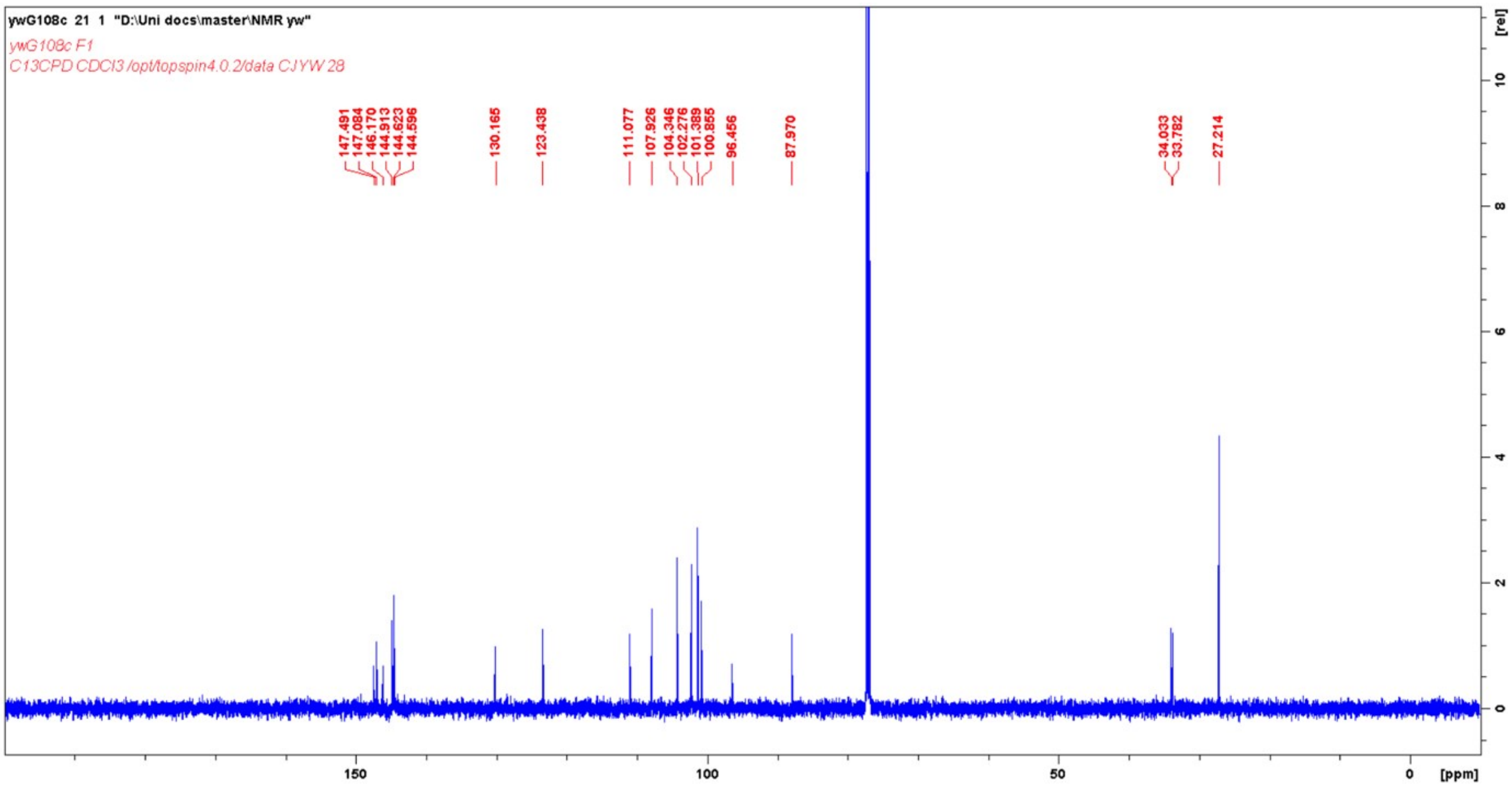

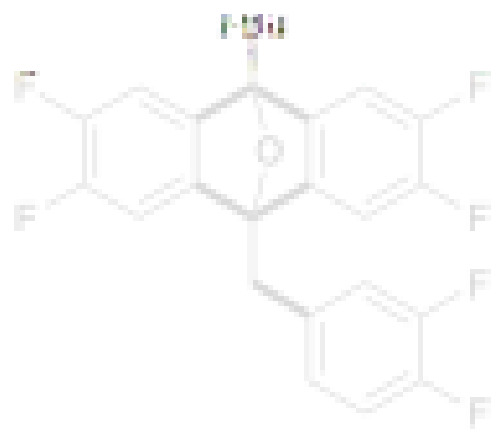

^{1}H NMR (400 MHz, $CDCl_3$), **16c**

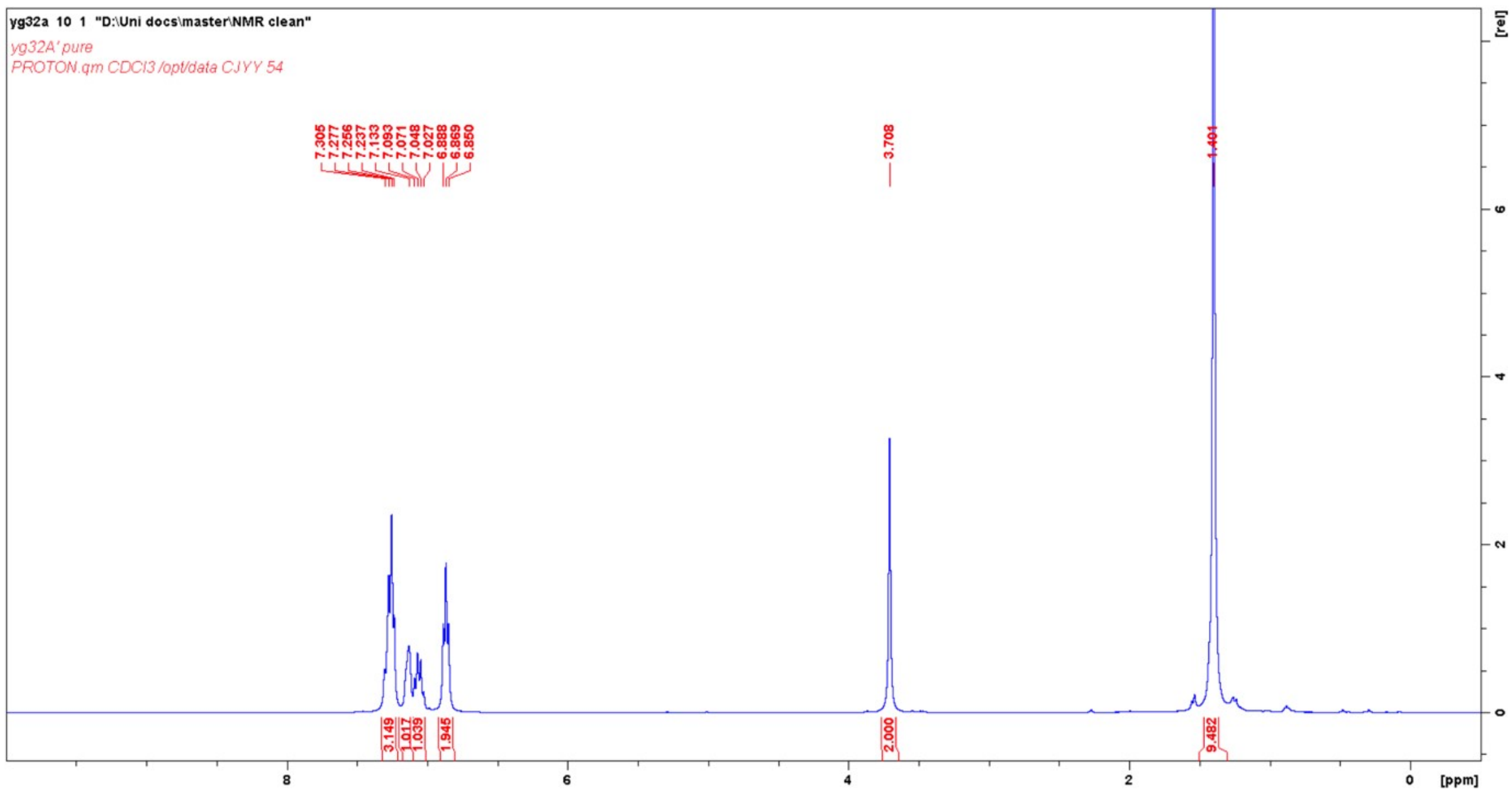

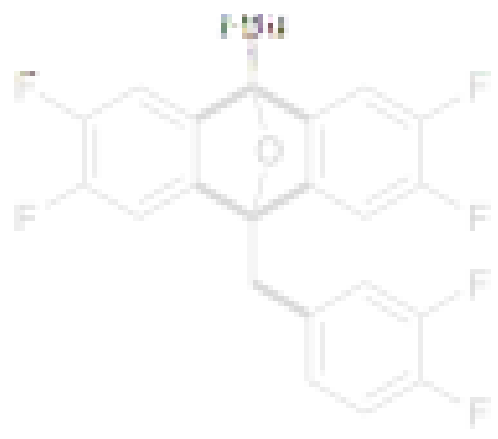

^{13}C NMR (101 MHz, $CDCl_3$), **16c**

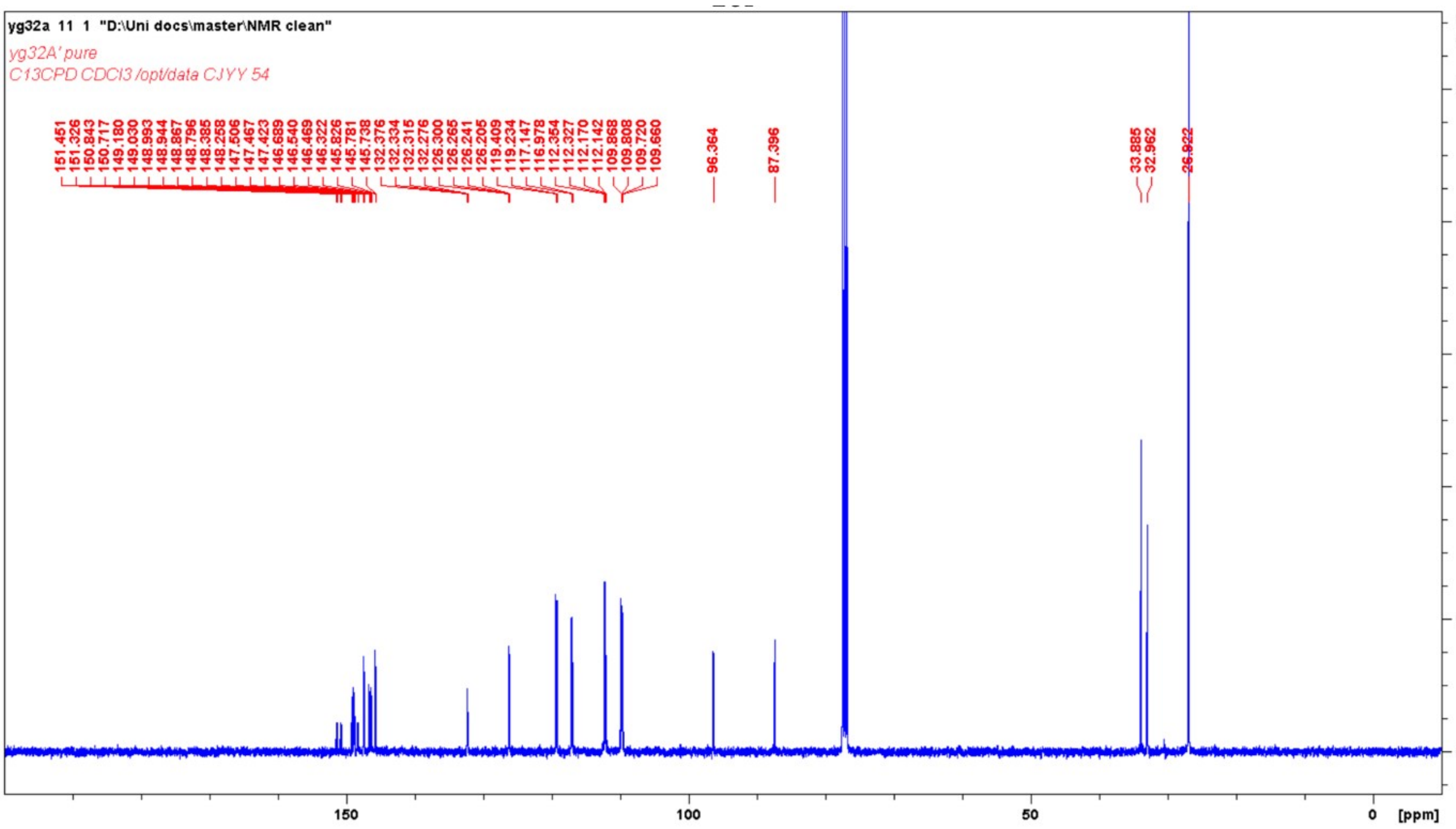

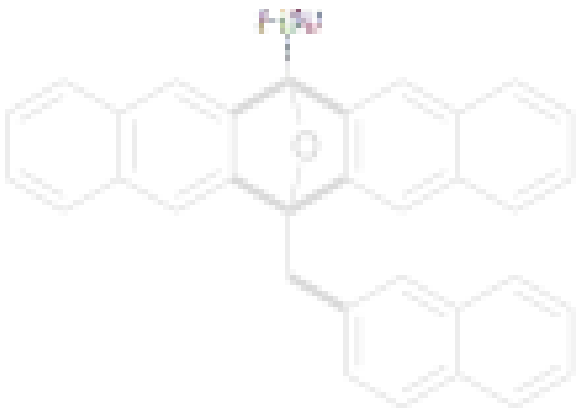

^{1}H NMR (400 MHz, $CDCl_3$), **16g**

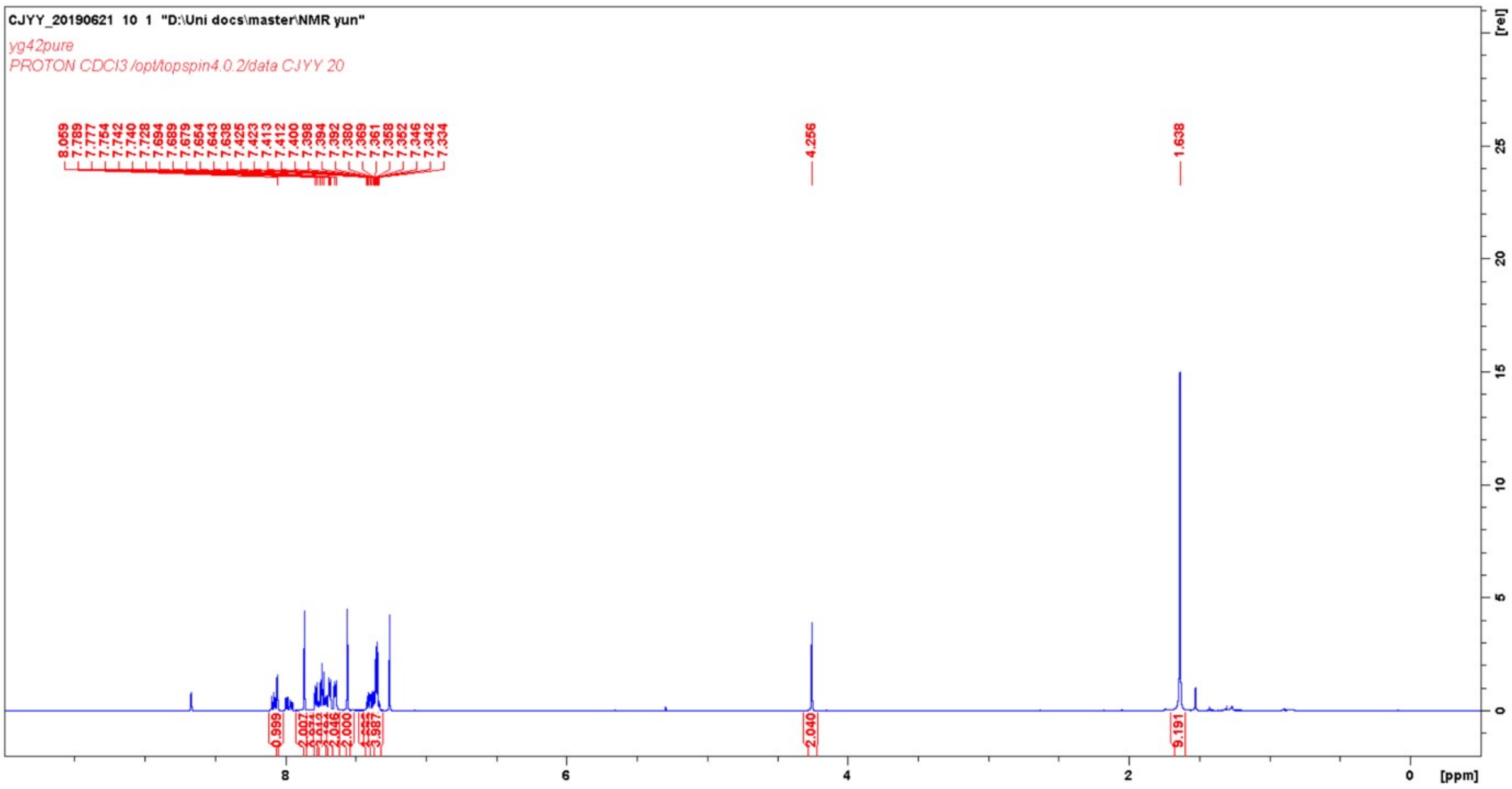

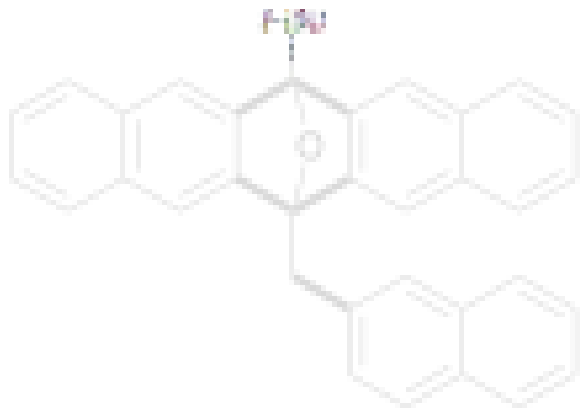

^{13}C NMR (101 MHz, $CDCl_3$), **16g**

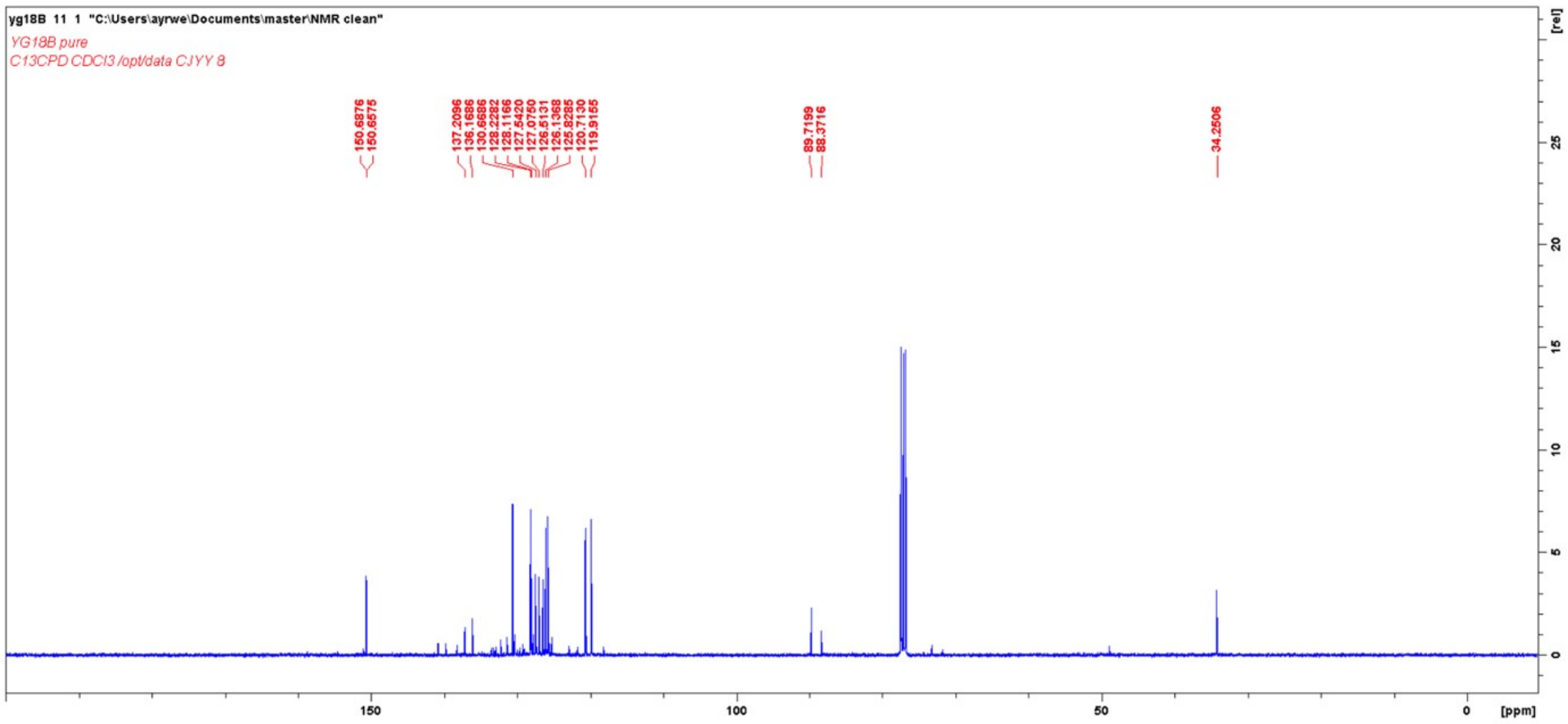

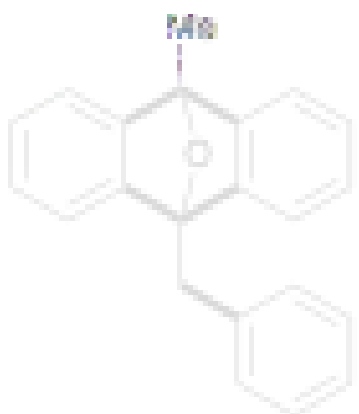

^{1}H NMR (400 MHz, $CDCl_3$), **25a**

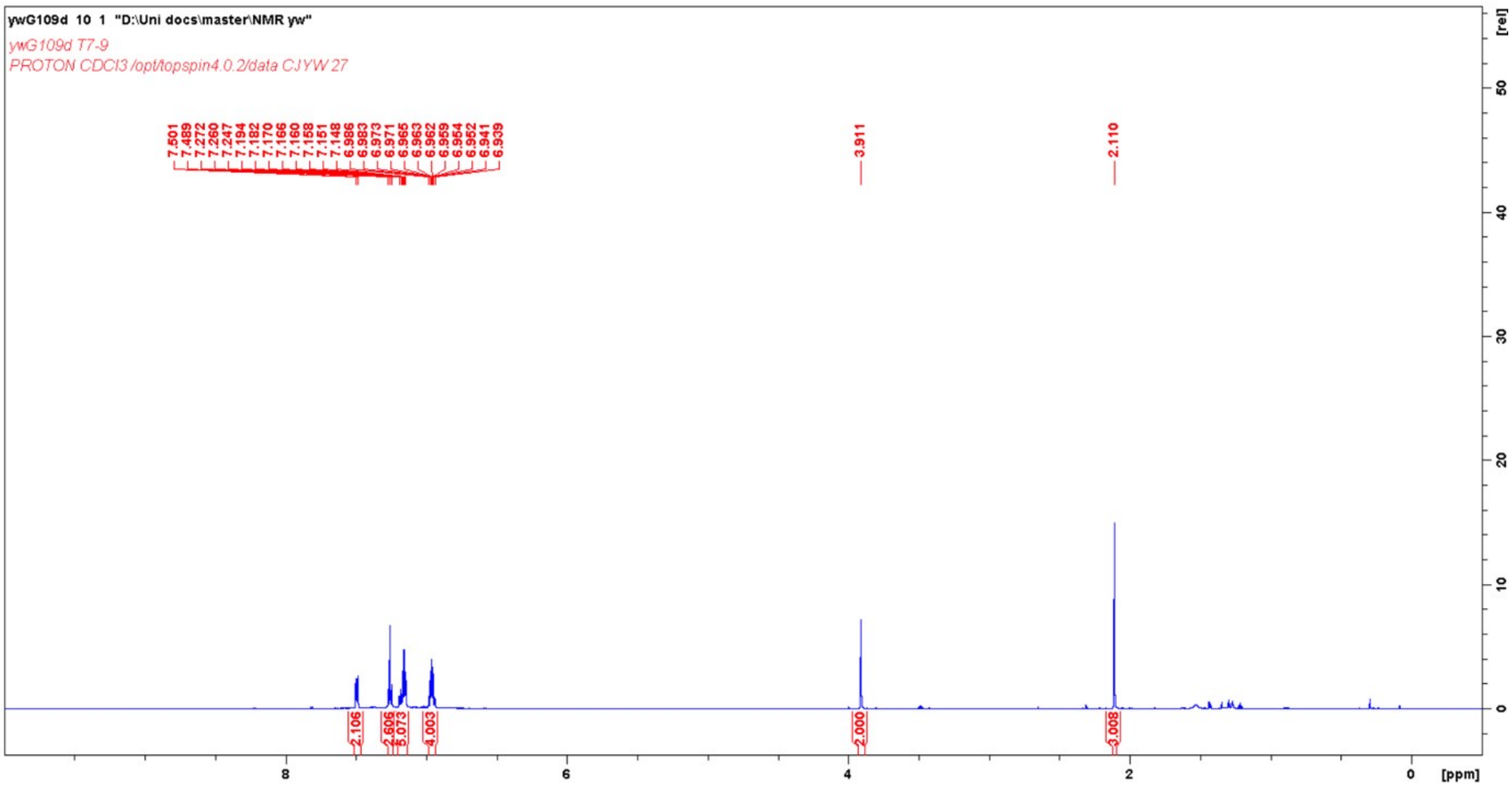

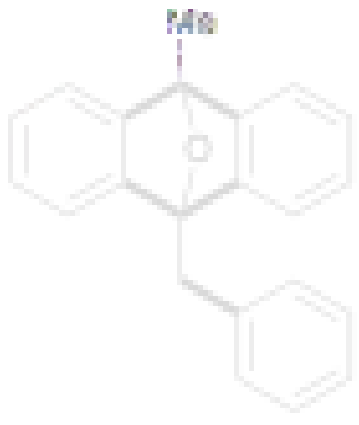

^{13}C NMR (101 MHz, $CDCl_3$), **25a**

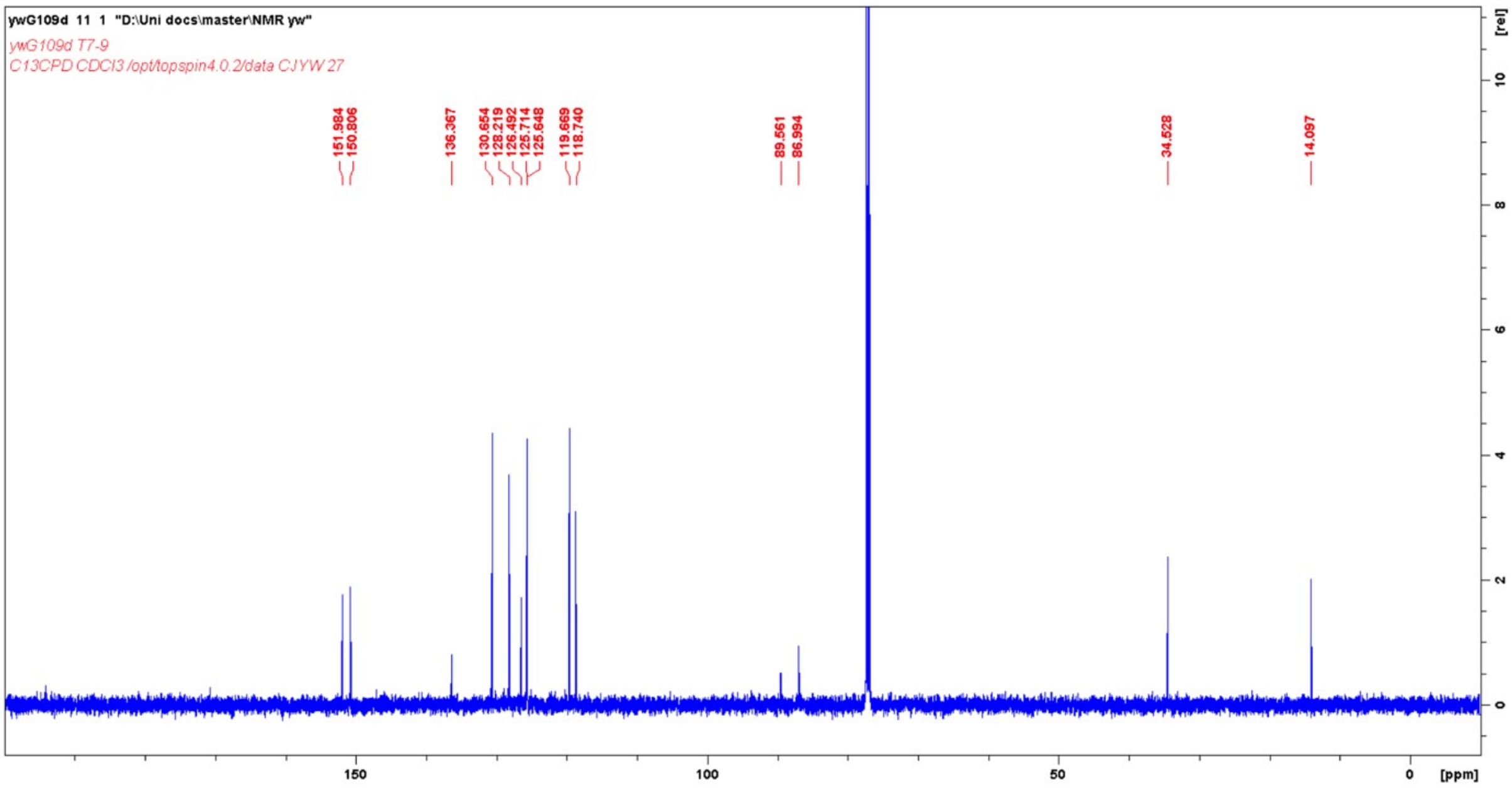

^1H NMR (400 MHz, $CDCl_3$), **25b**

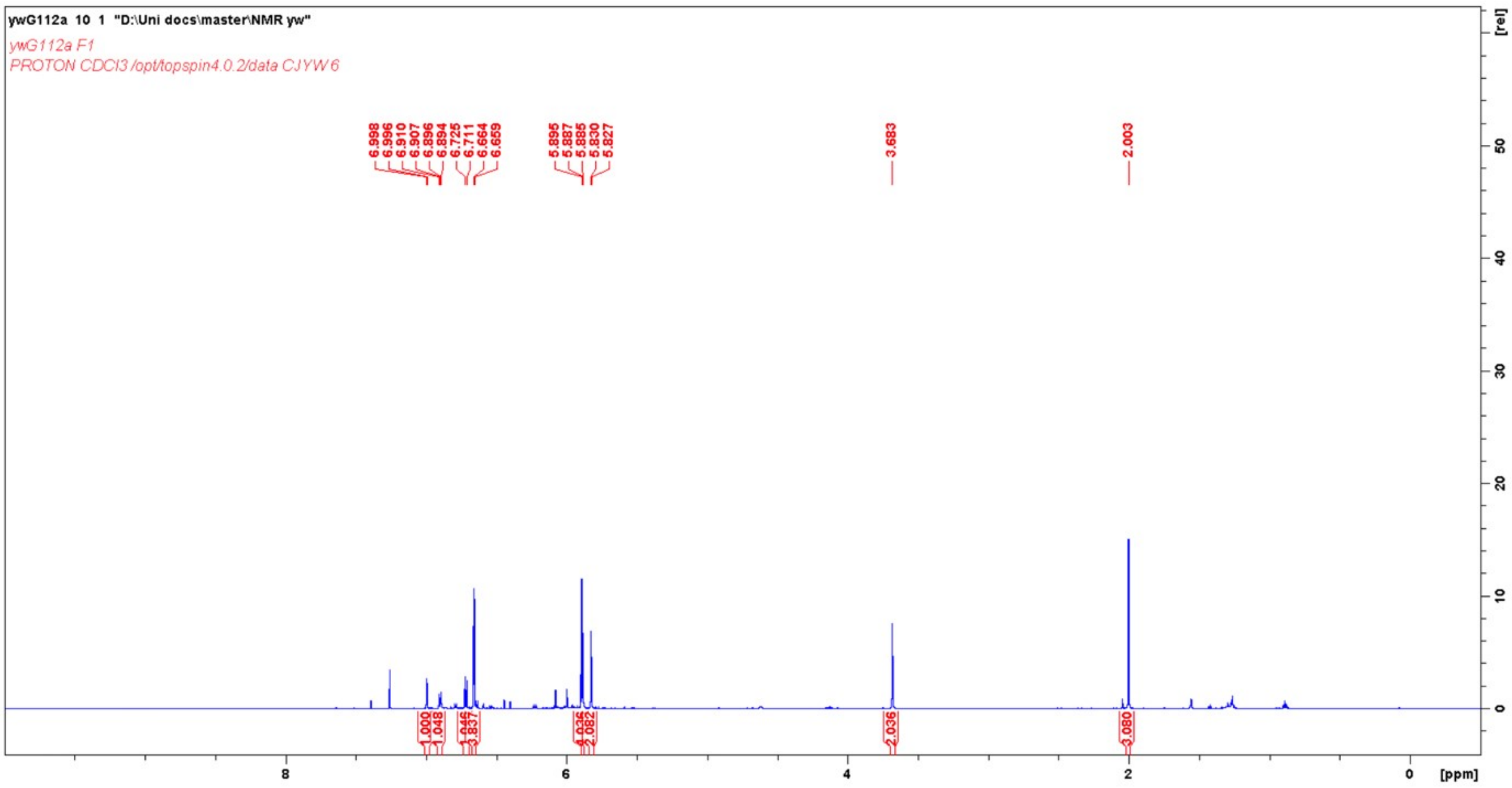

^{13}C NMR (101 MHz, $CDCl_3$), **25b**

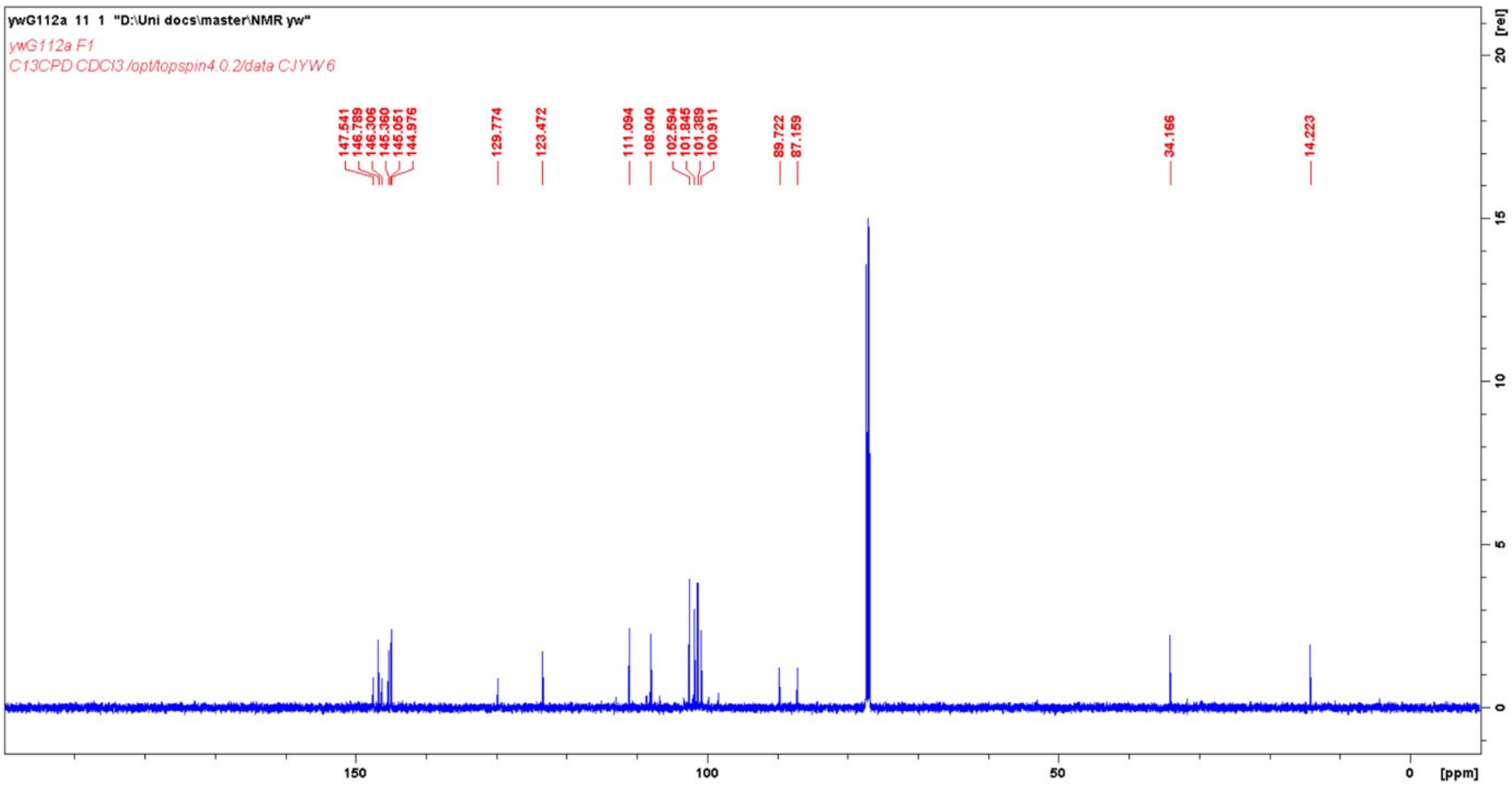

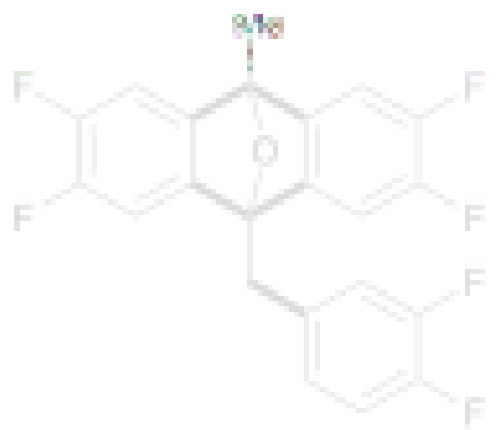

^{1}H NMR (400 MHz, $CDCl_3$), **25c**

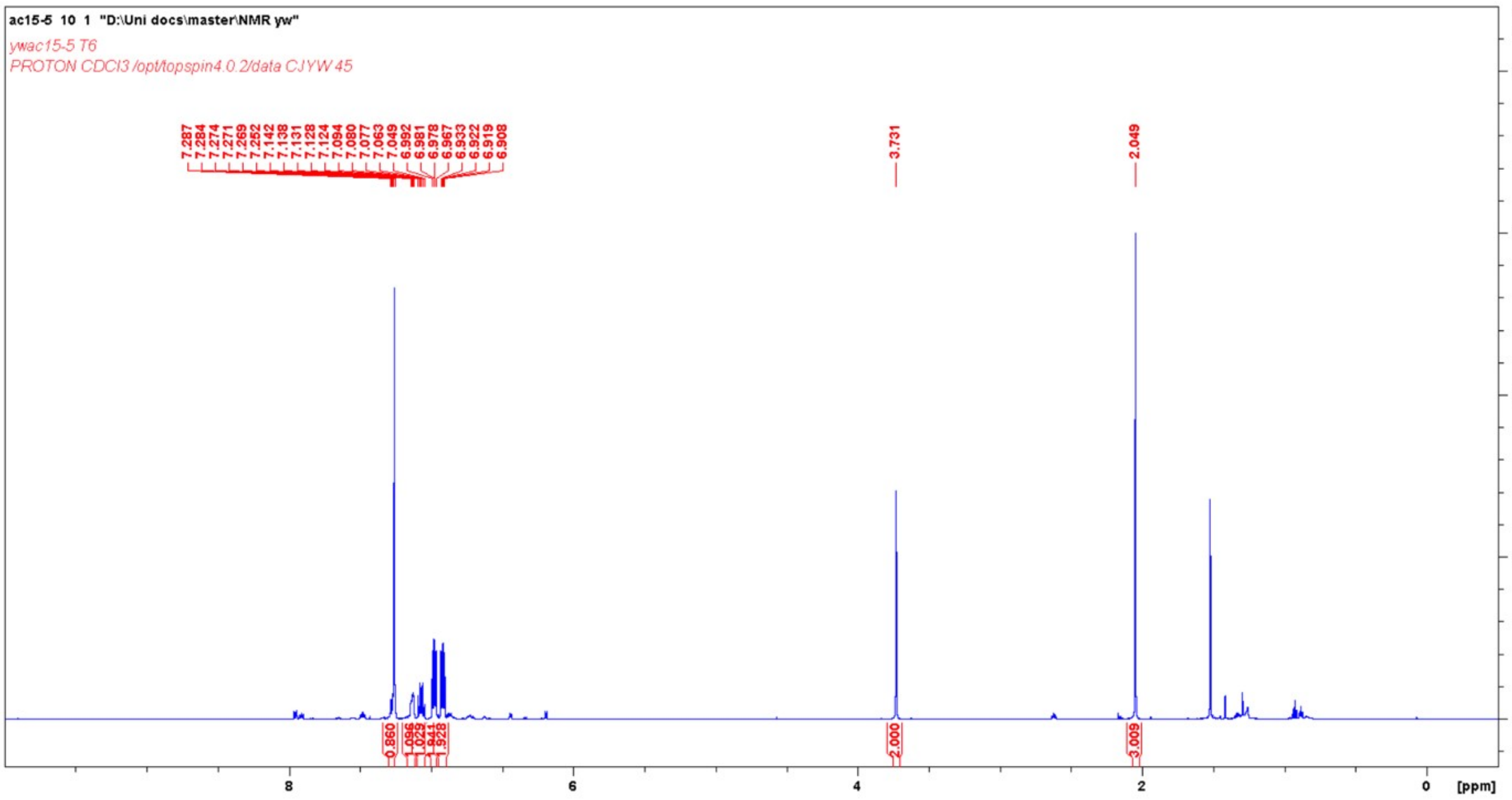

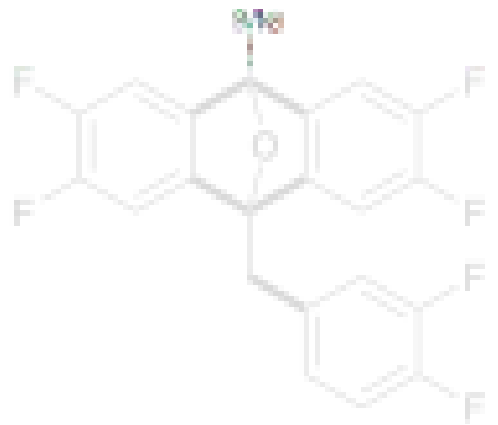

^{13}C NMR (101 MHz, $CDCl_3$), **25c**

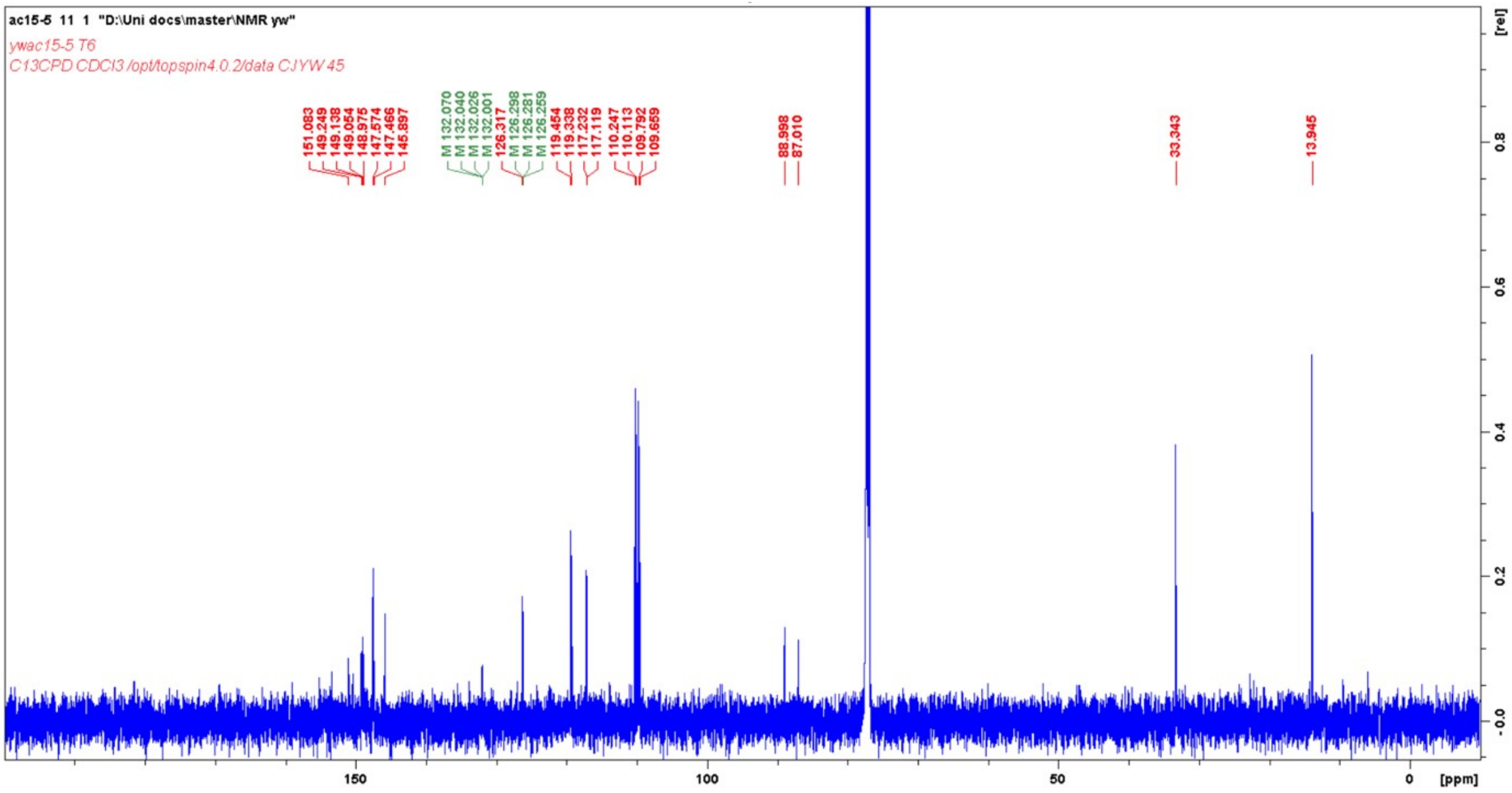

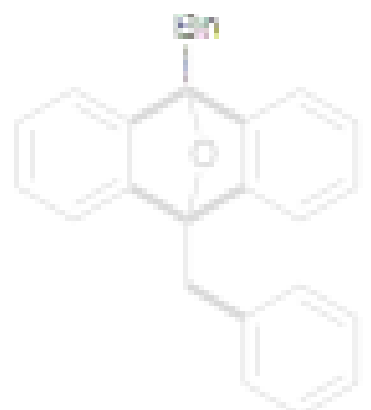

^{1}H NMR (400 MHz, $CDCl_3$), **26a**

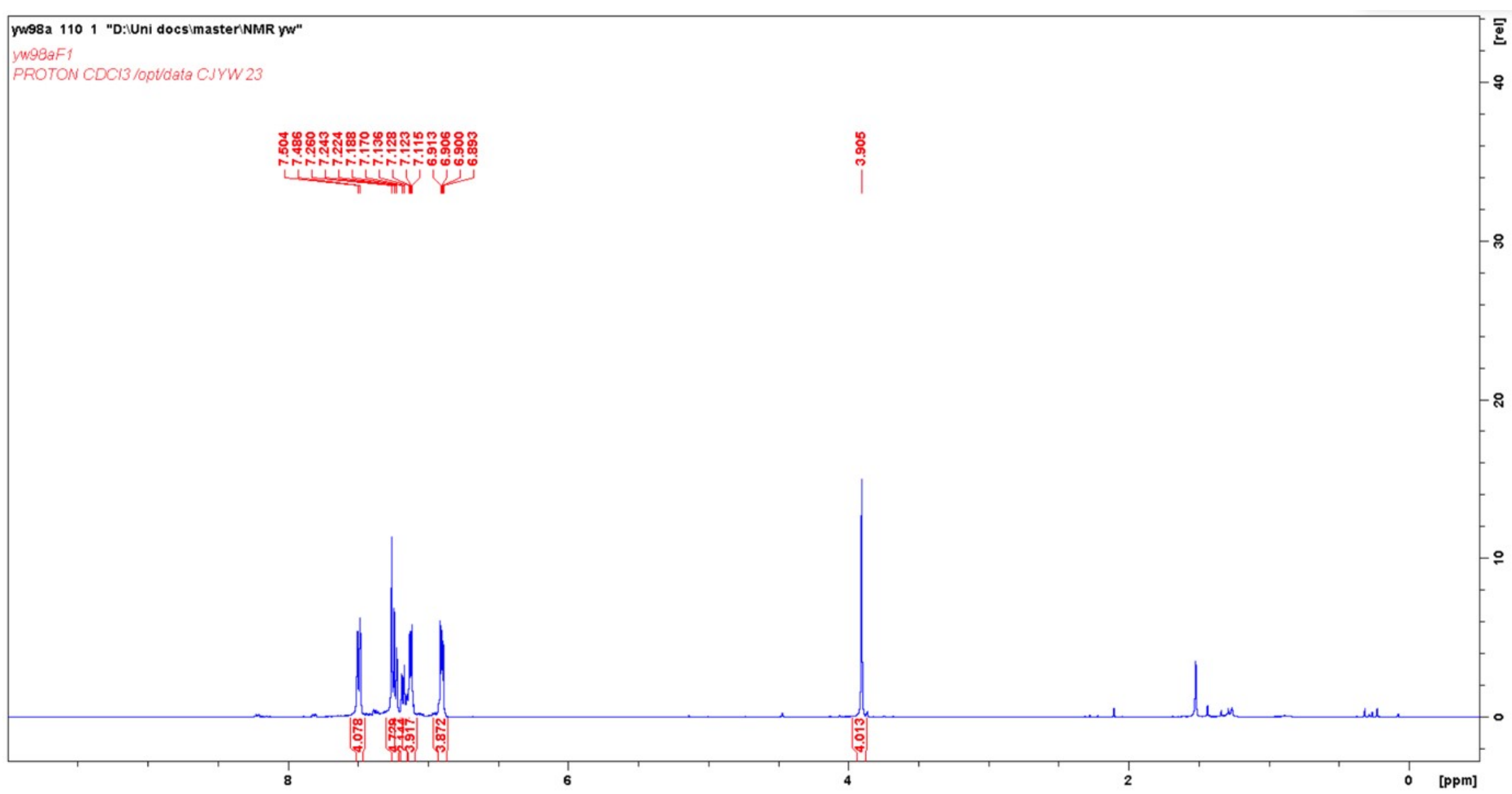

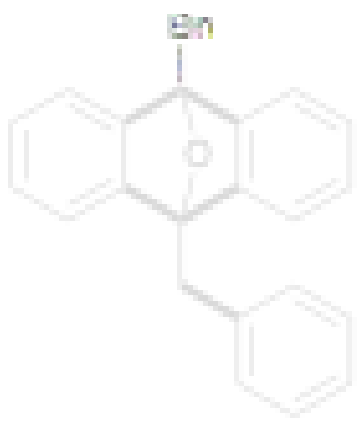

^{13}C NMR (101 MHz, $CDCl_3$), **26a**

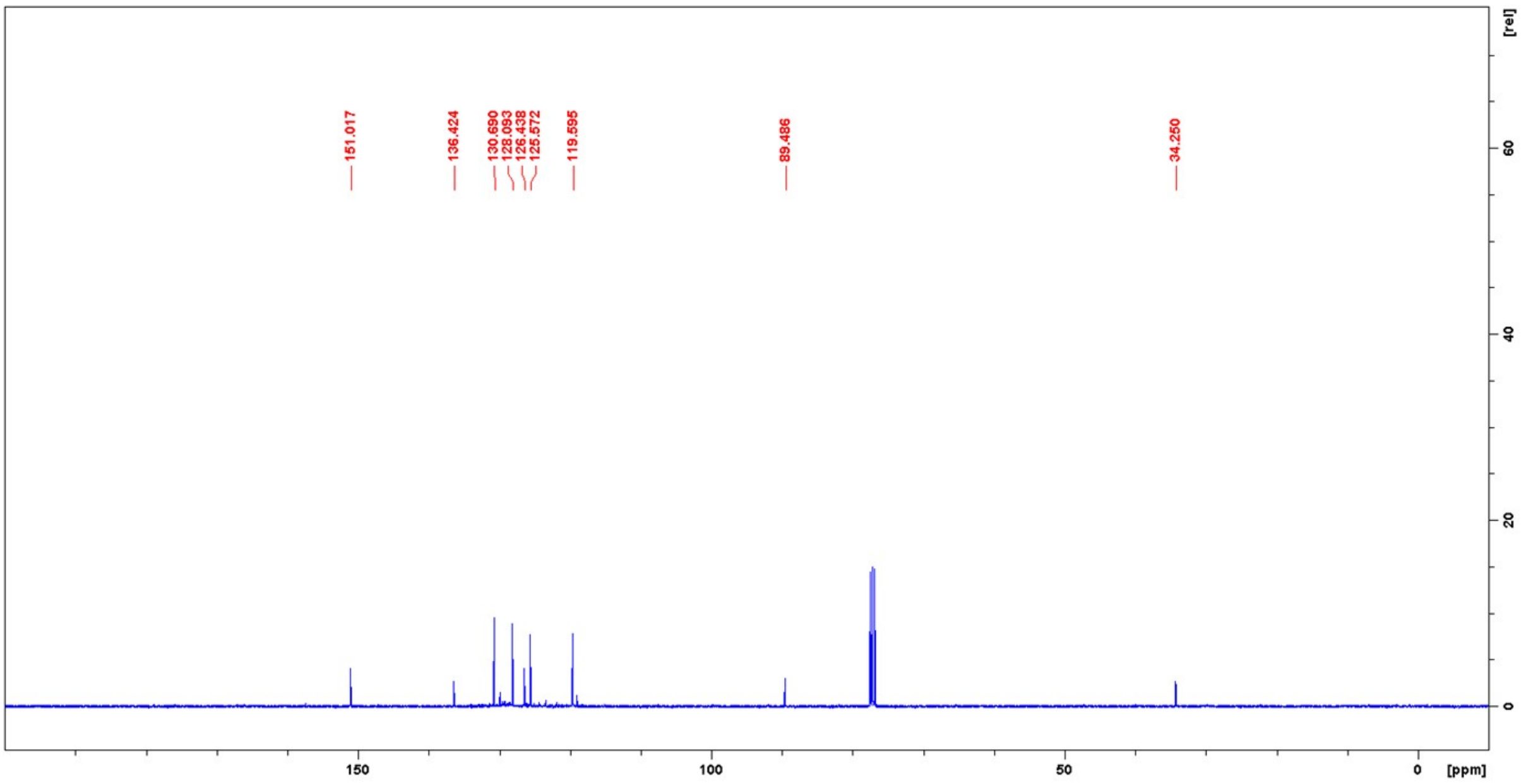

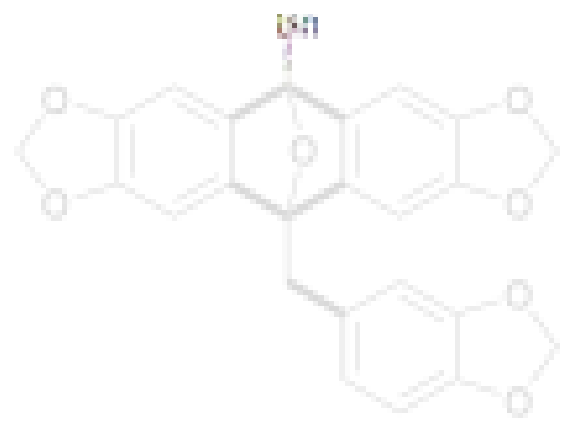

^1H NMR (400 MHz, $CDCl_3$), **26b**

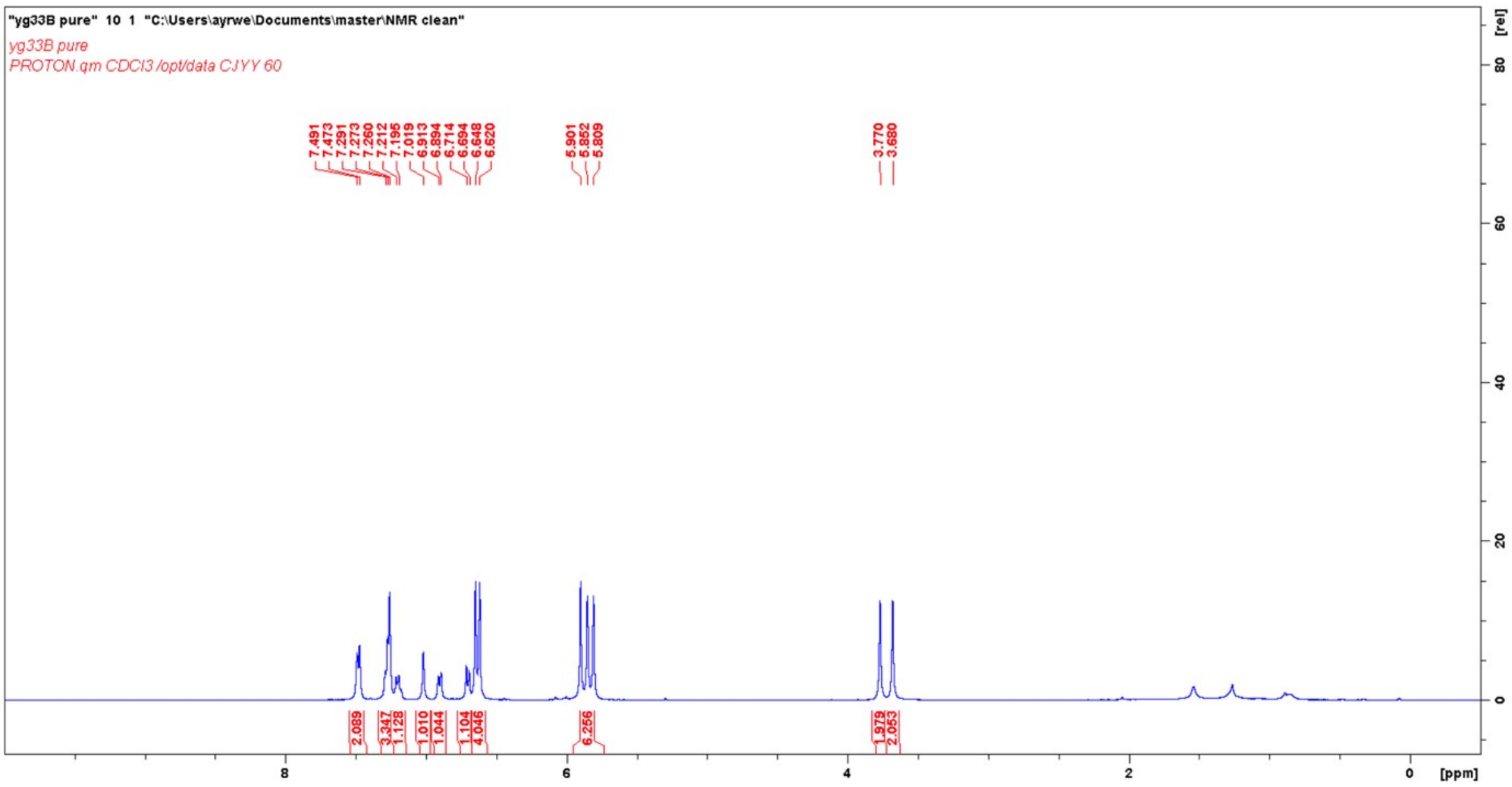

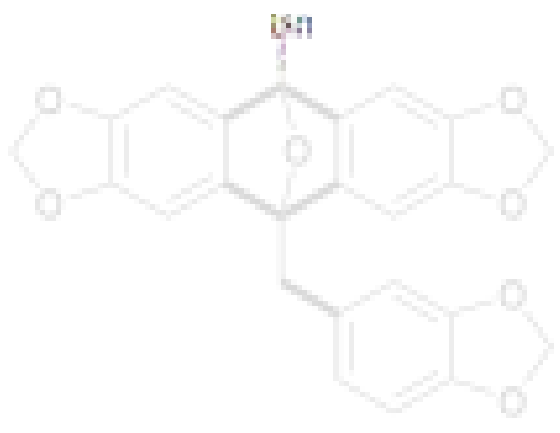

^{13}C NMR (101 MHz, $CDCl_3$), **26b**

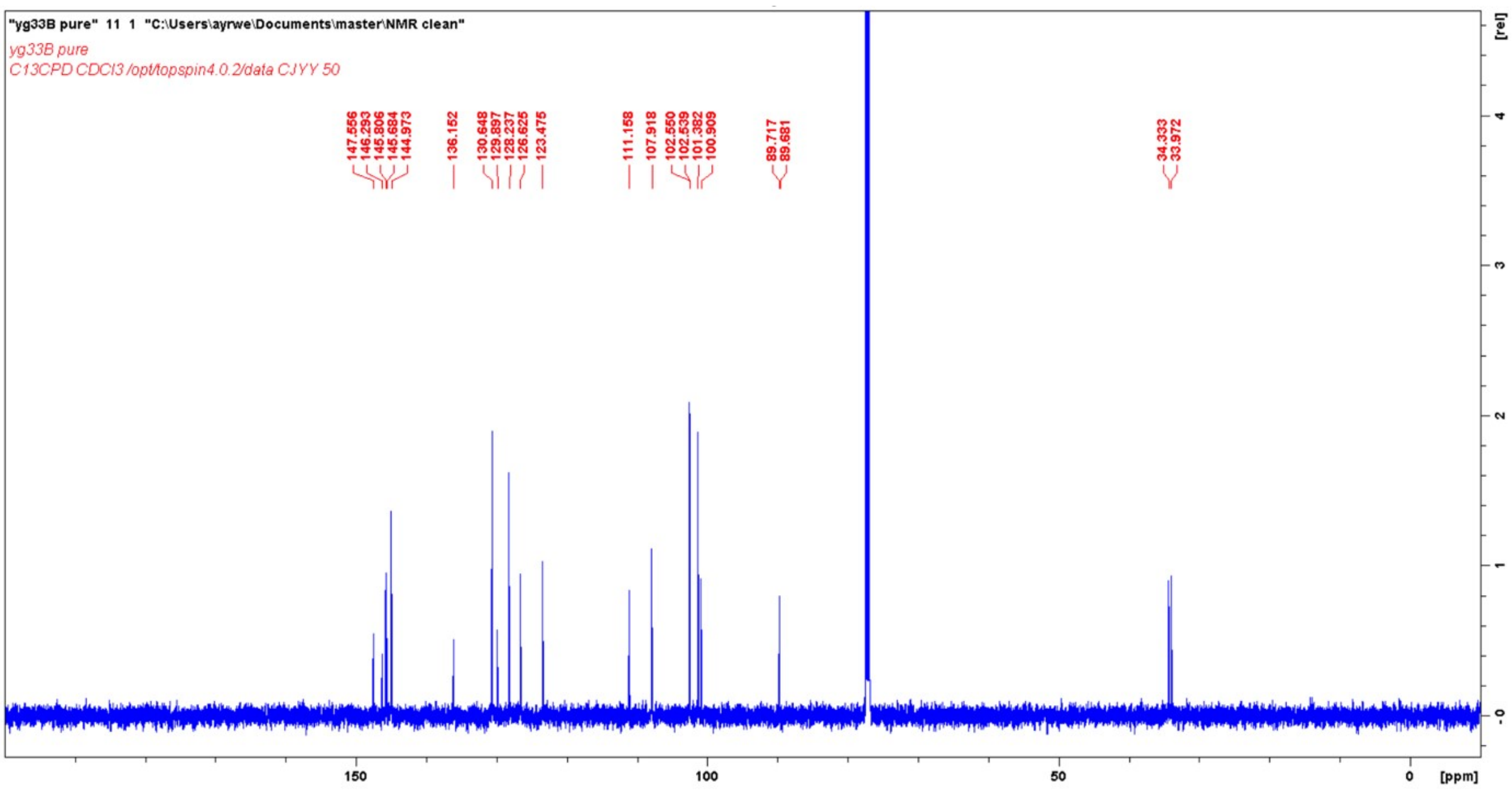

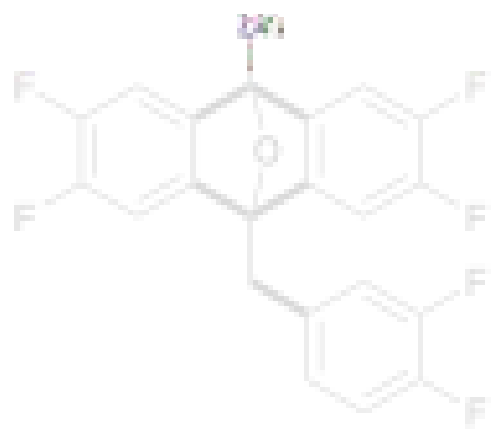

^1H NMR (400 MHz, $CDCl_3$), **26c**

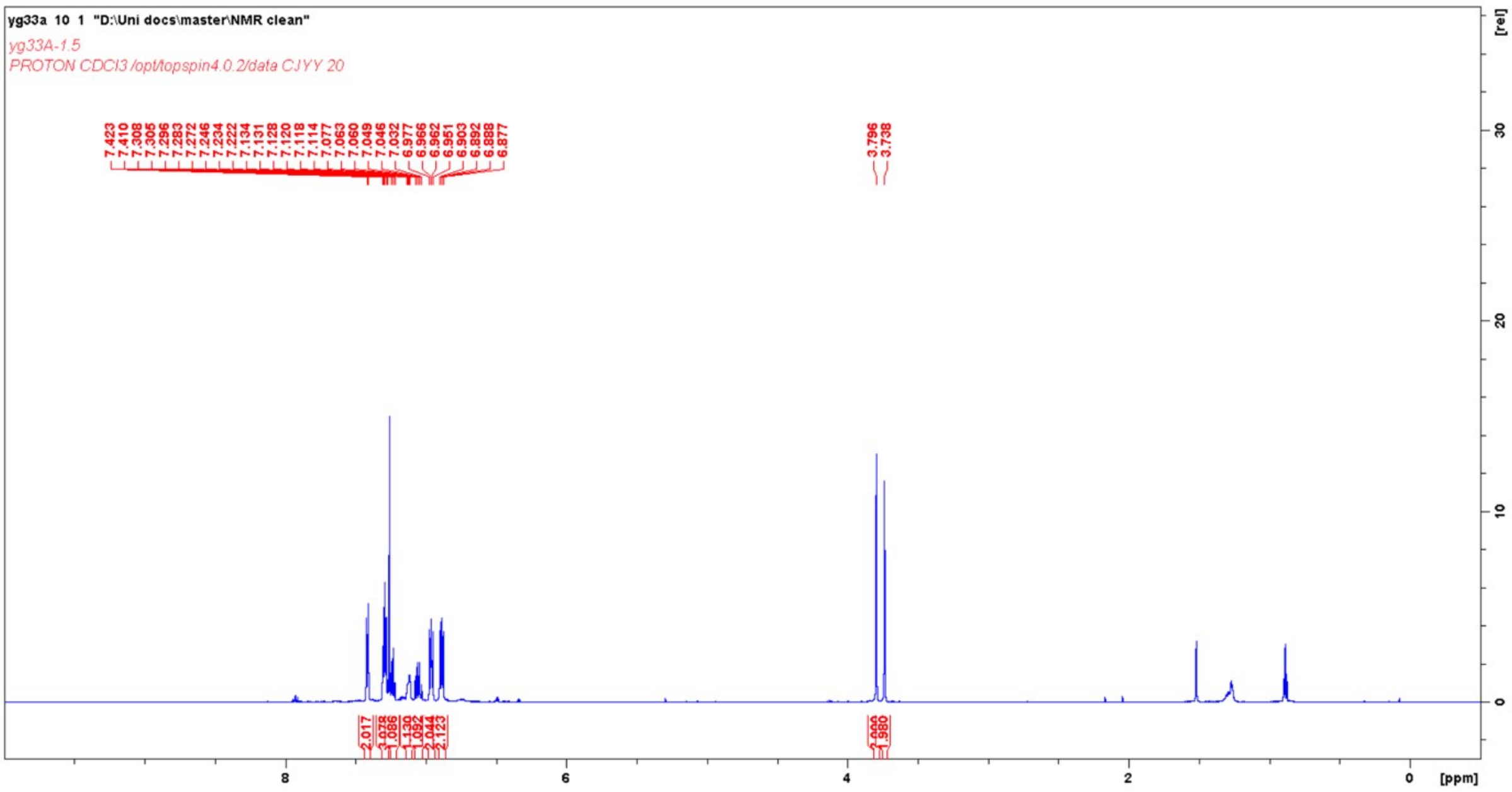

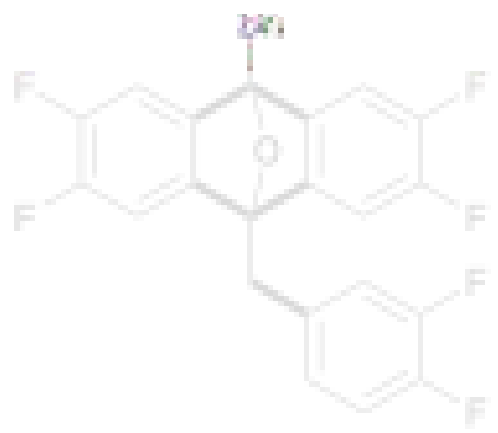

^{13}C NMR (101 MHz, $CDCl_3$), **26c**

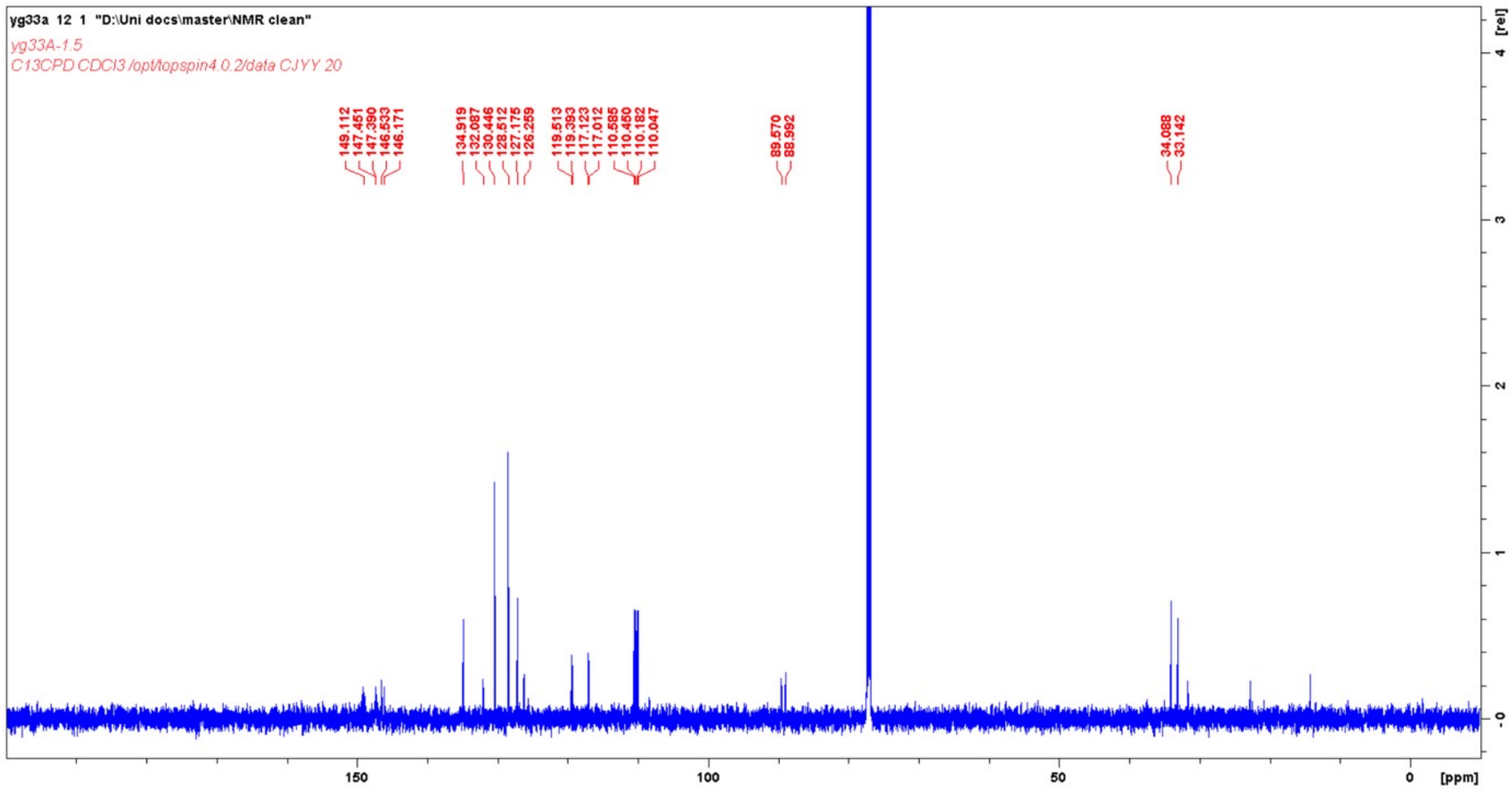

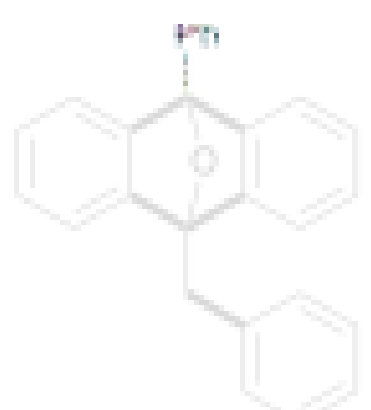

^1H NMR (400 MHz, $CDCl_3$), **27a**

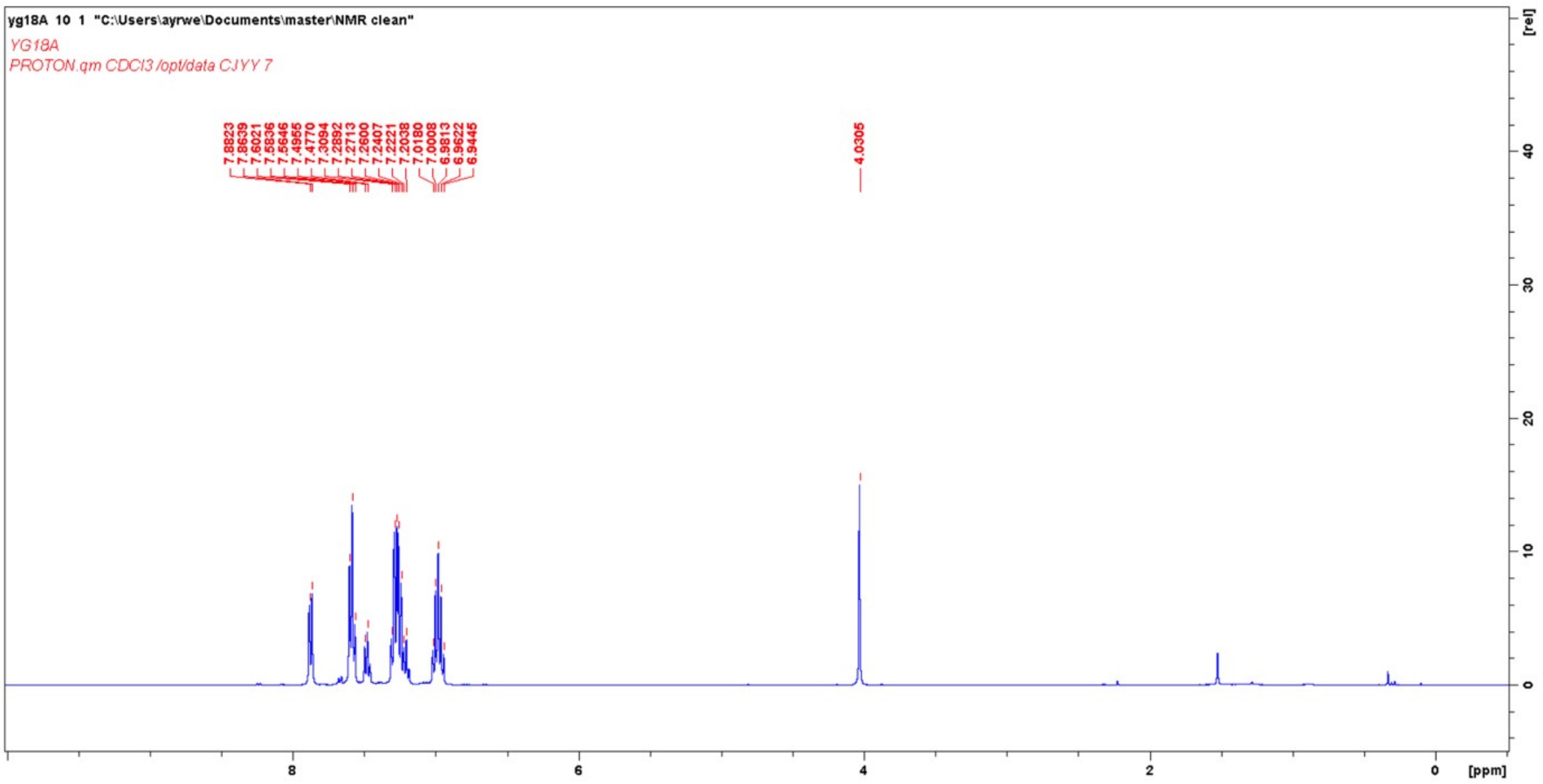

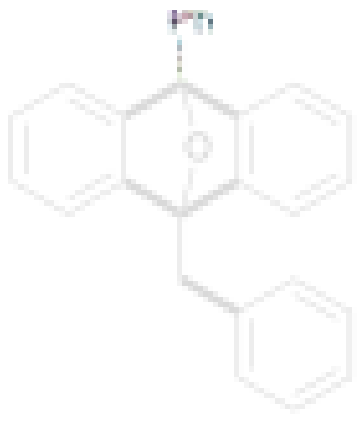

^{13}C NMR (101 MHz, $CDCl_3$), **27a**

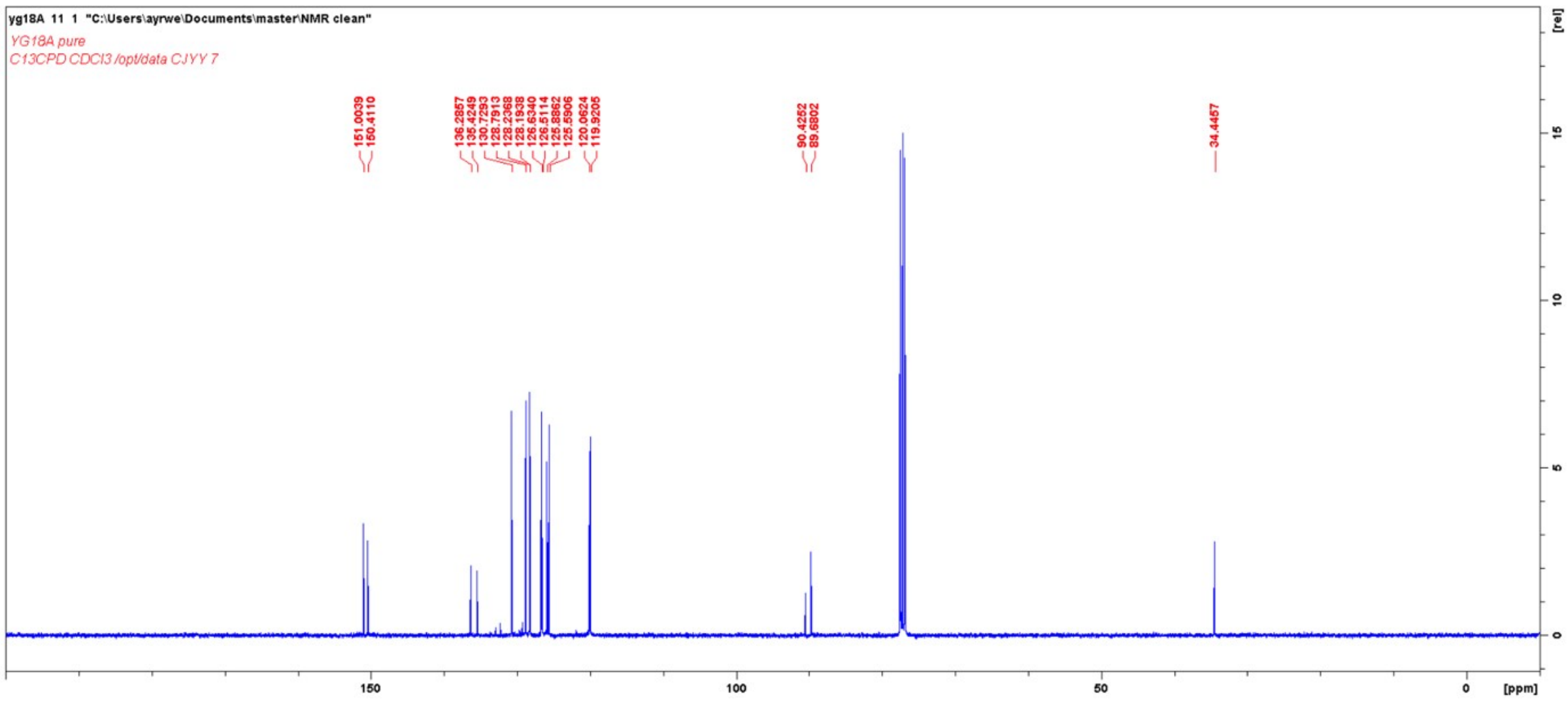

^{1}H NMR (400 MHz, $CDCl_3$), **27b**

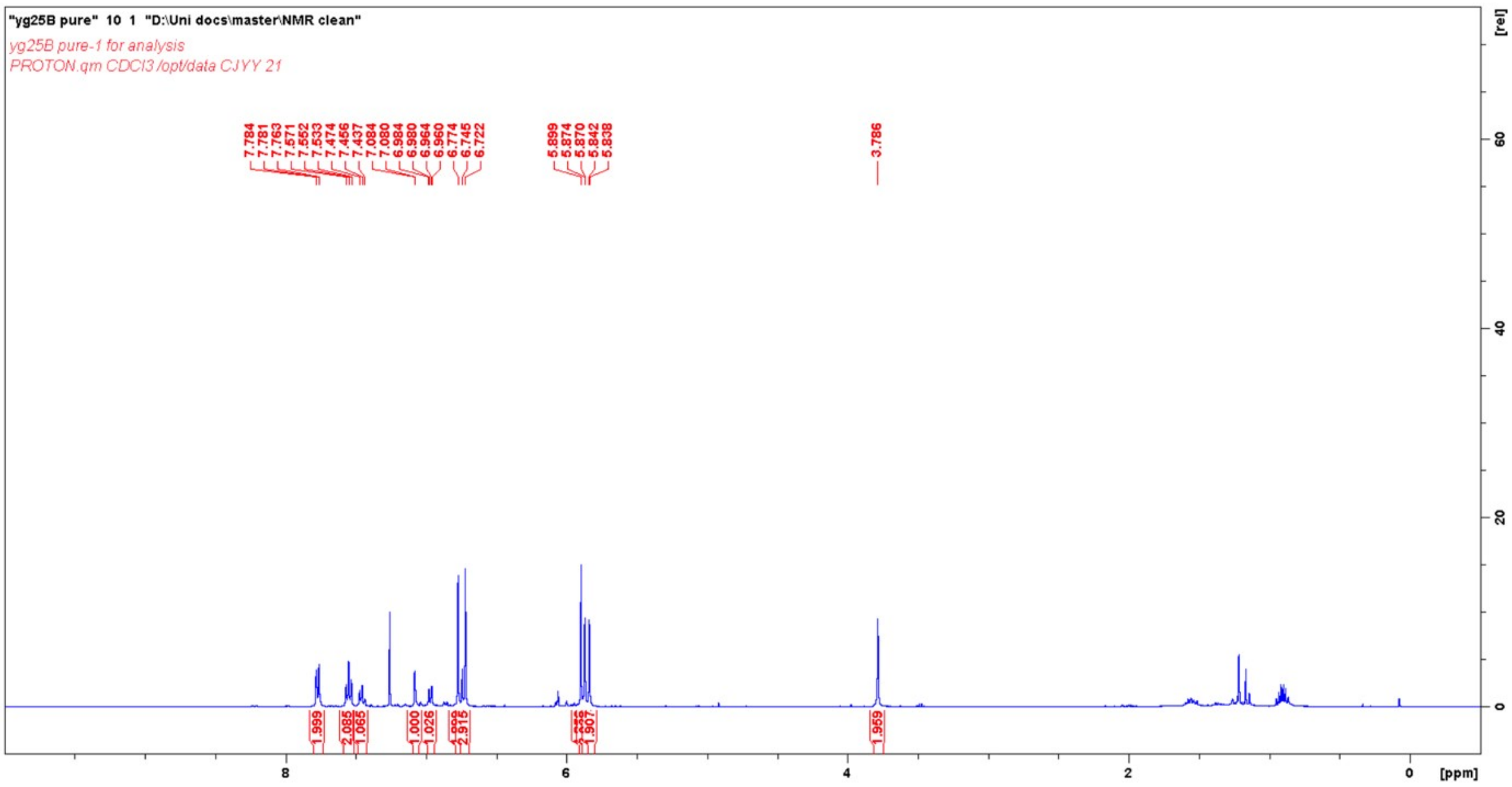

^{13}C NMR (101 MHz, $CDCl_3$), **27b**

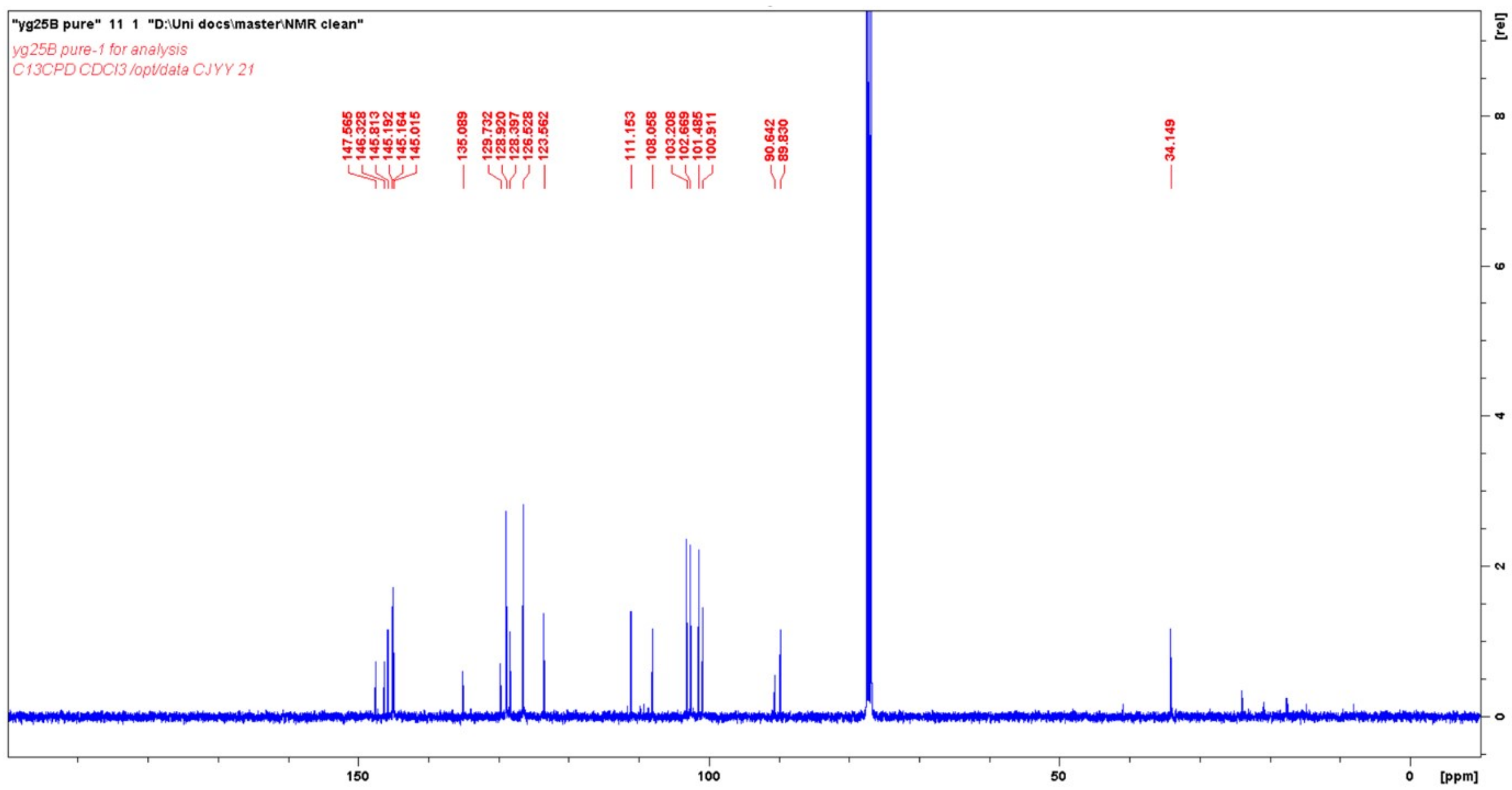

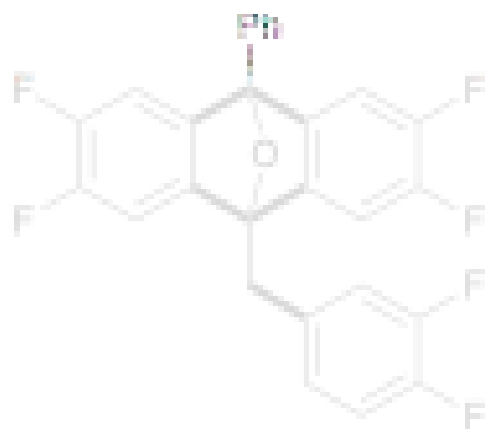

^1H NMR (400 MHz, $CDCl_3$), **27c**

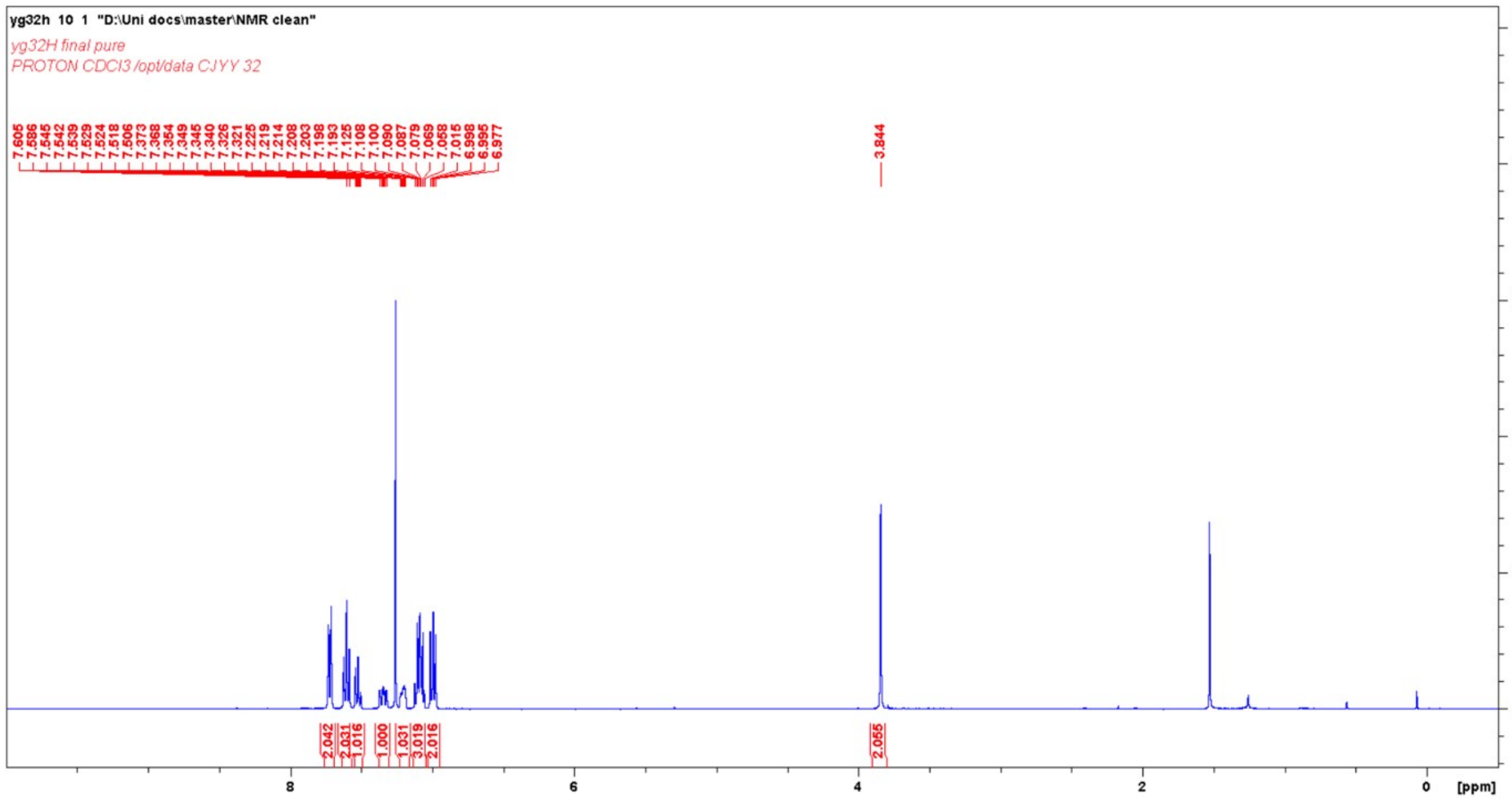

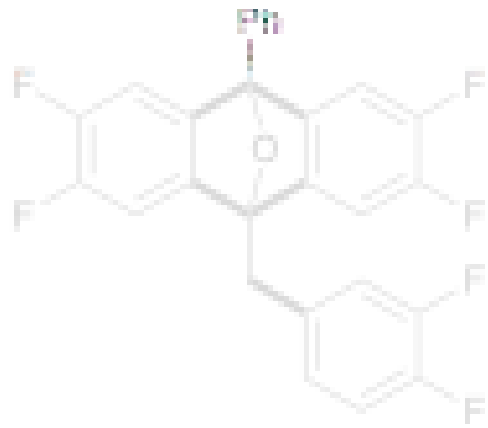

^{13}C NMR (101 MHz, $CDCl_3$), **27c**

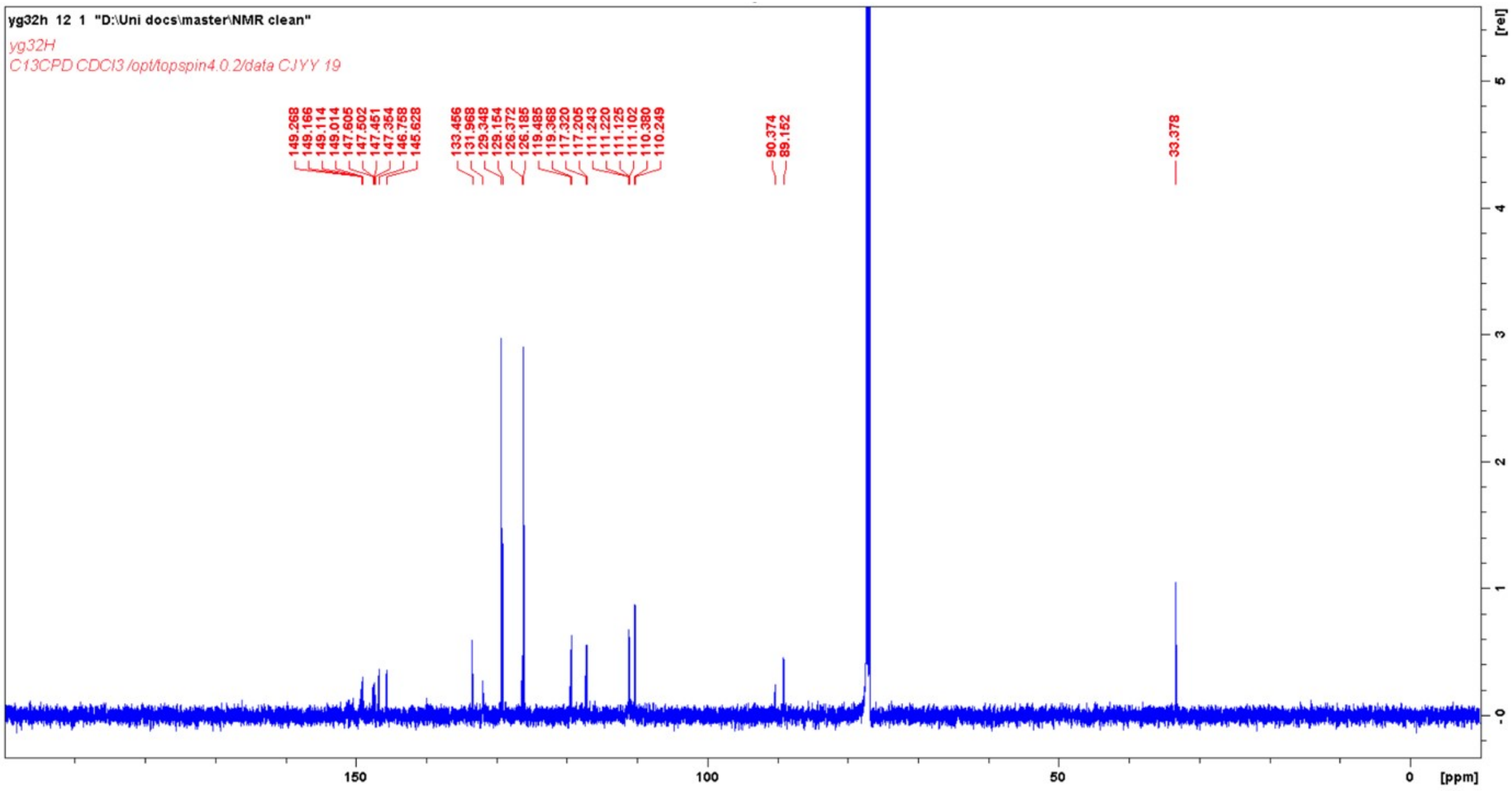

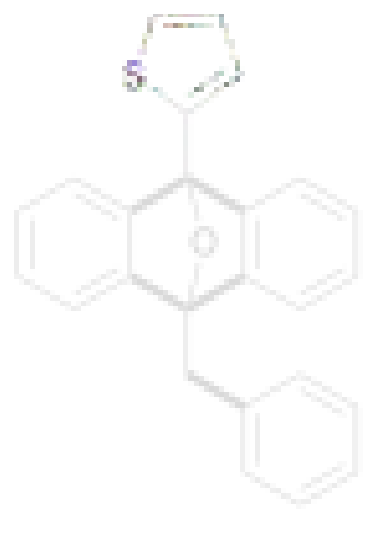

^{1}H NMR (400 MHz, $CDCl_3$), **28a**

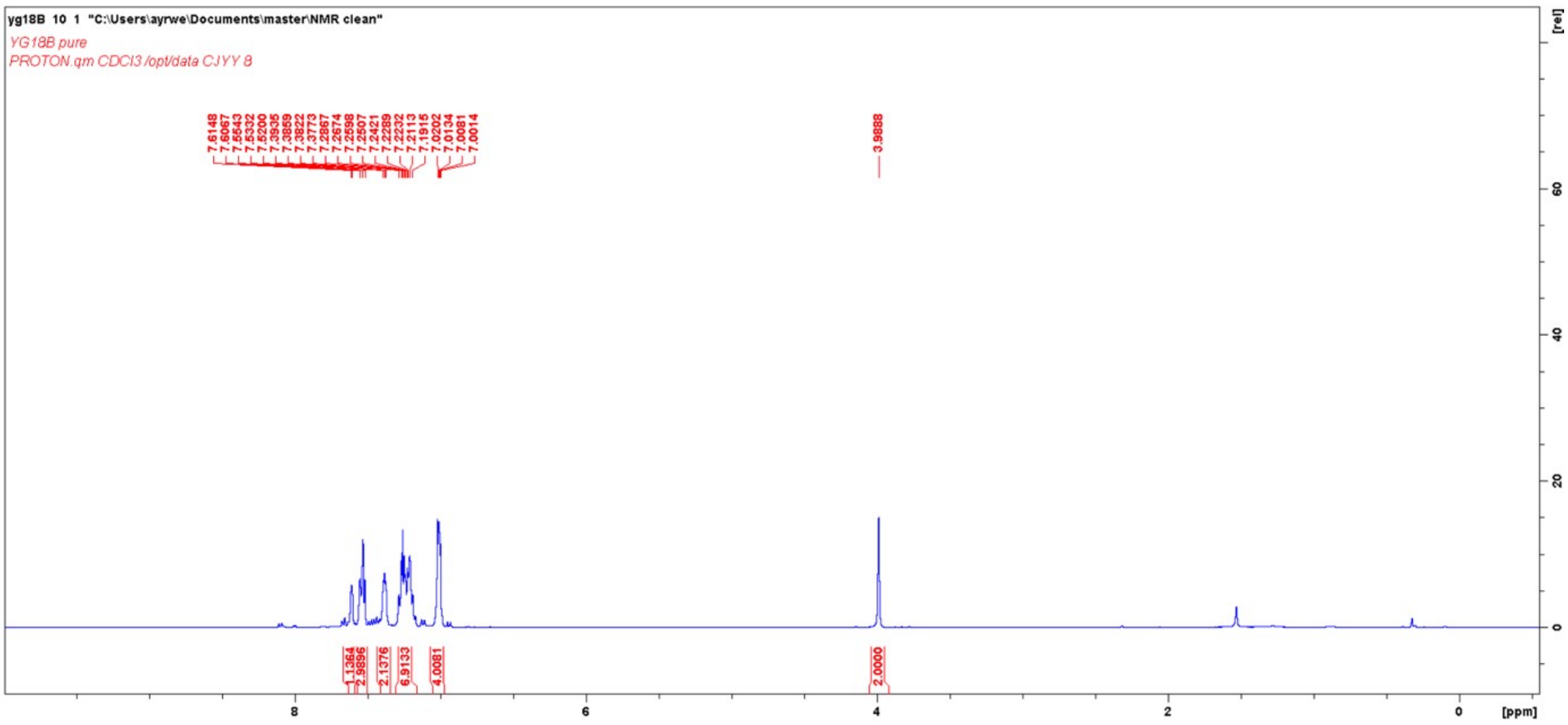

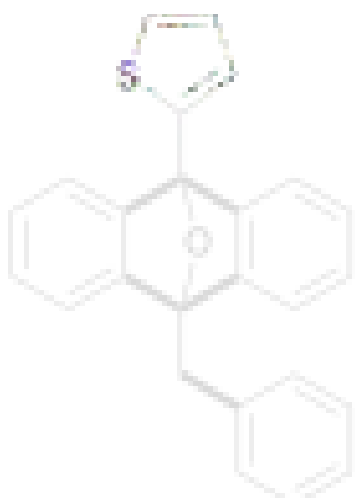

^{13}C NMR (101 MHz, $CDCl_3$), **28a**

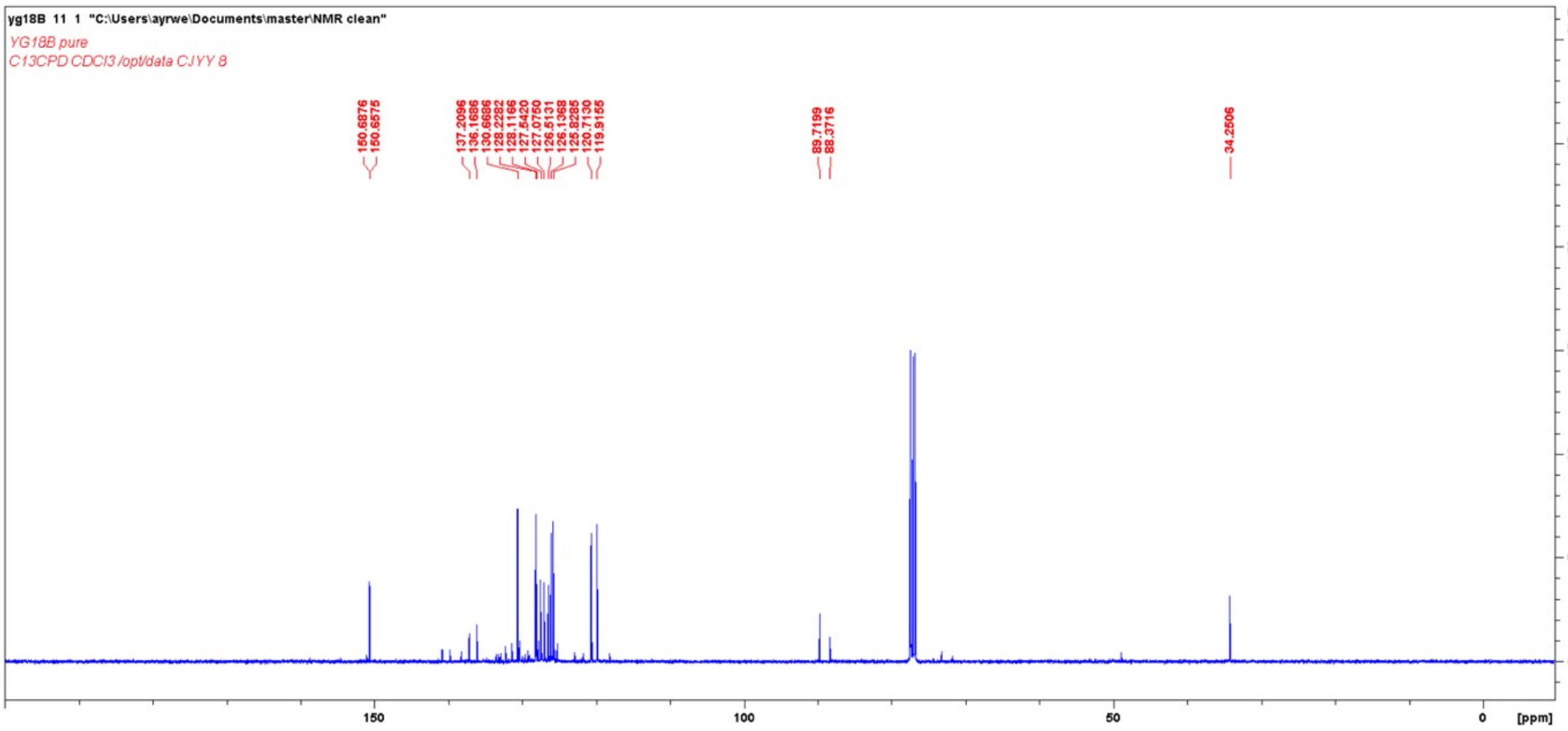

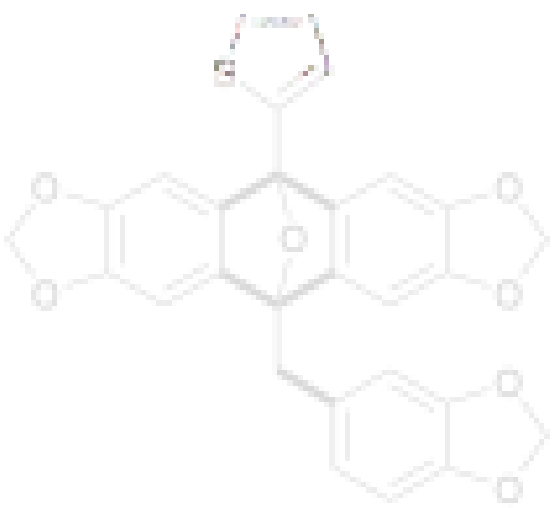

^1H NMR (400 MHz, $CDCl_3$), **28b**

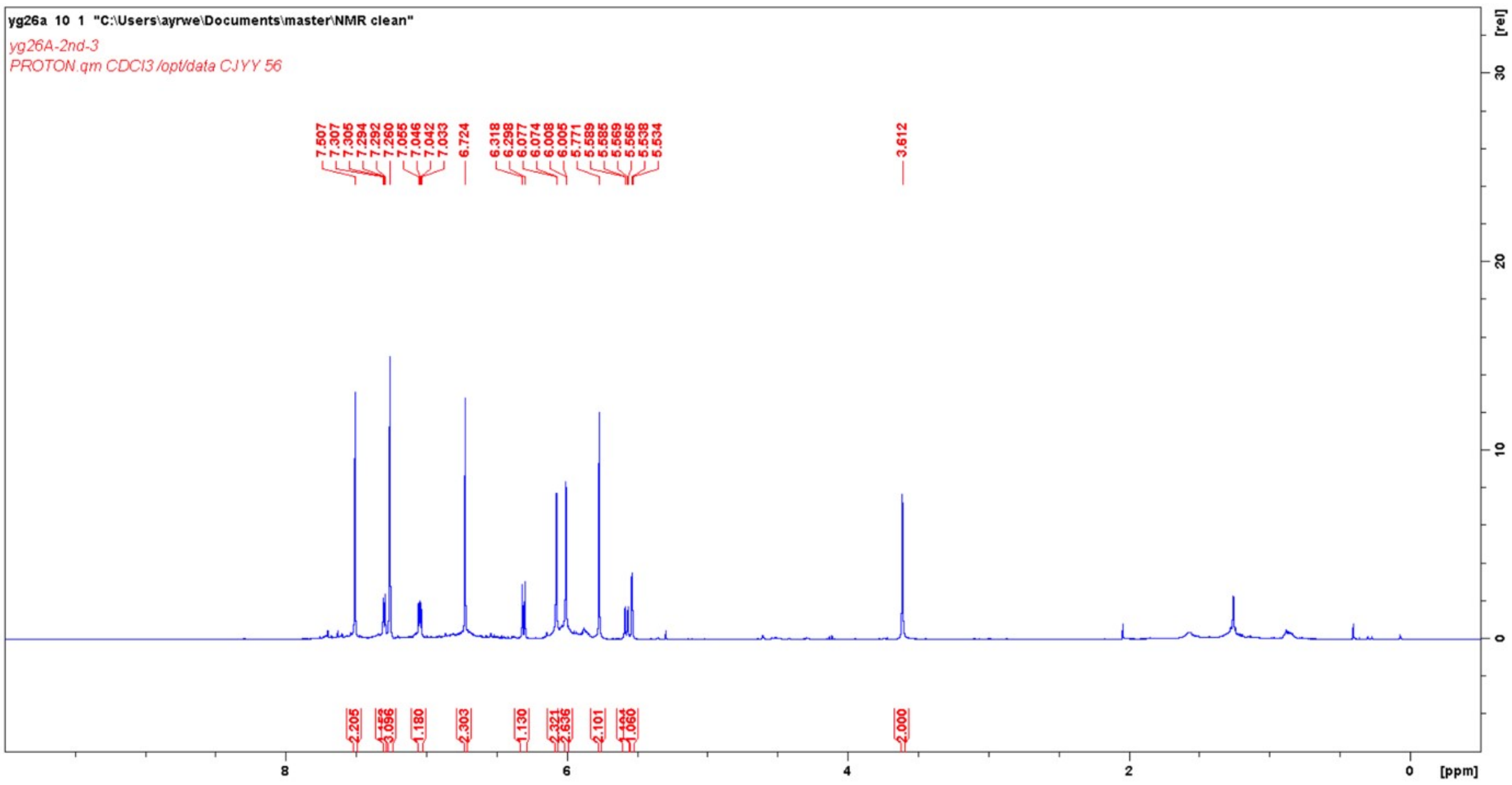

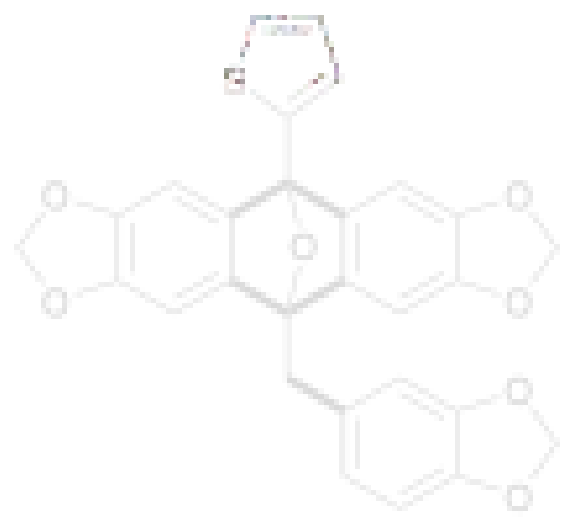

^{13}C NMR (101 MHz, $CDCl_3$), **28b**

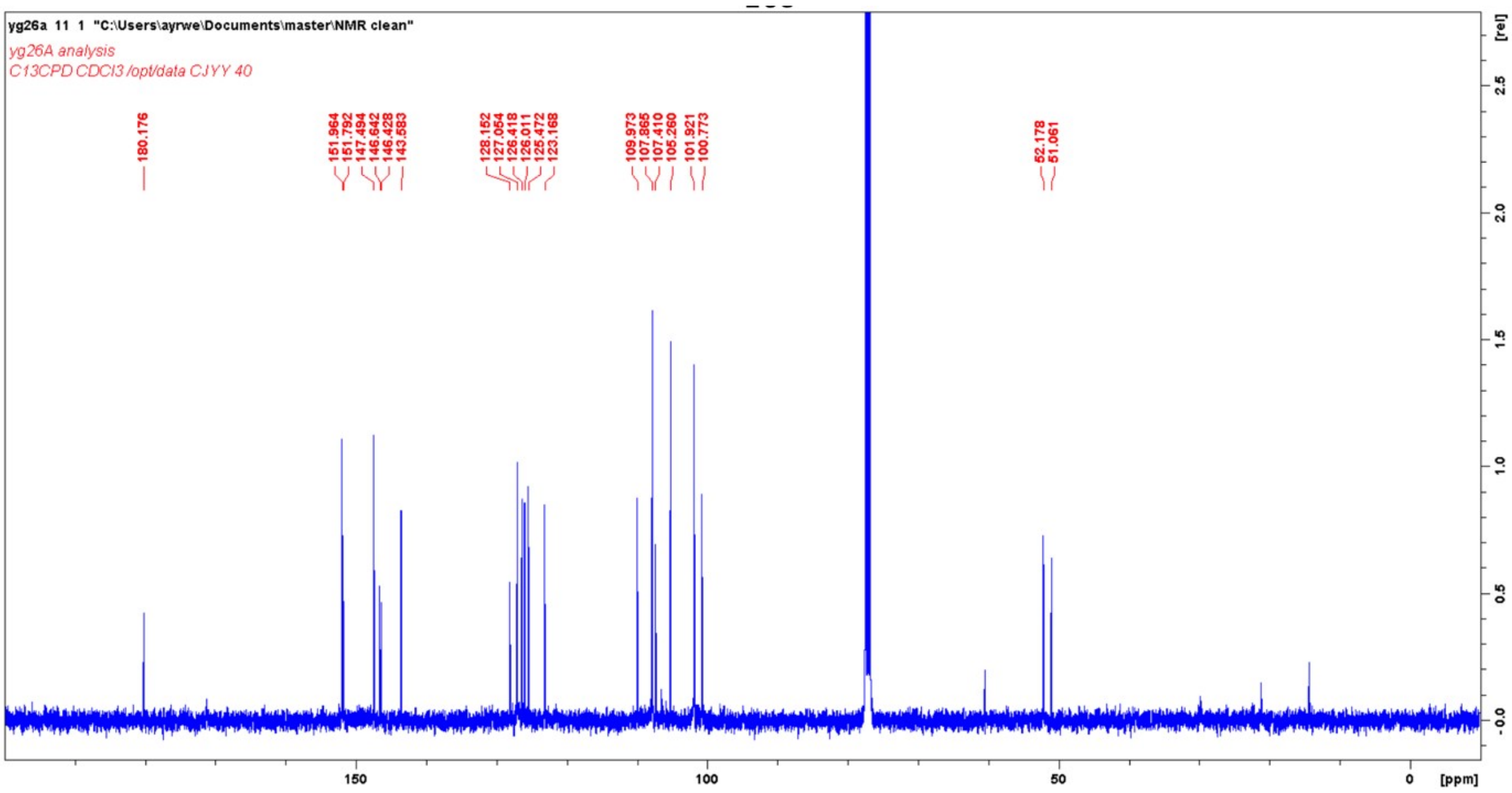

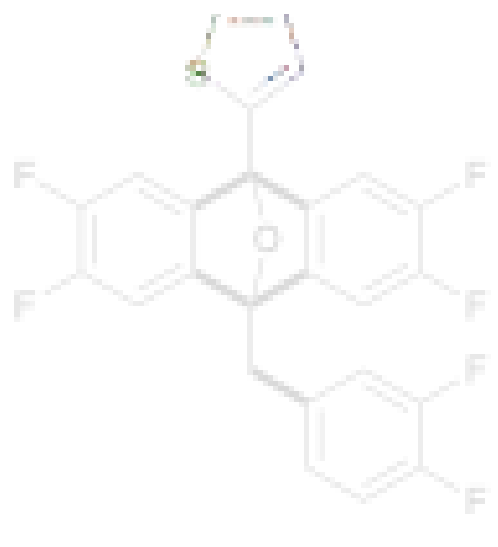

^{1}H NMR (400 MHz, $CDCl_3$), **28c**

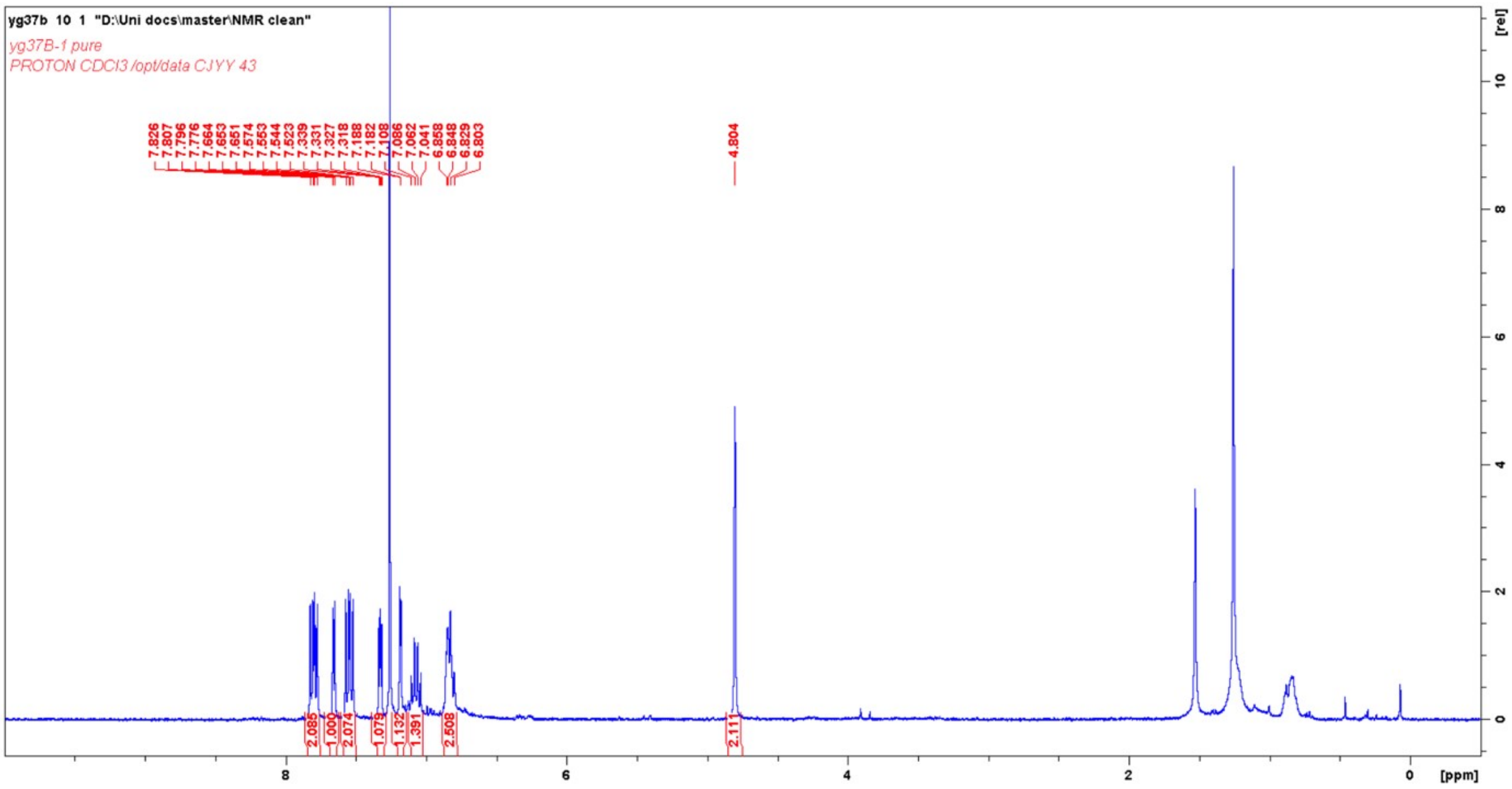

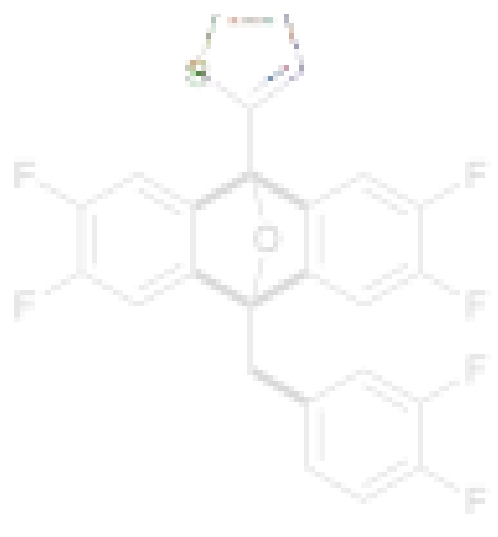

^{13}C NMR (101 MHz, $CDCl_3$), **28c**

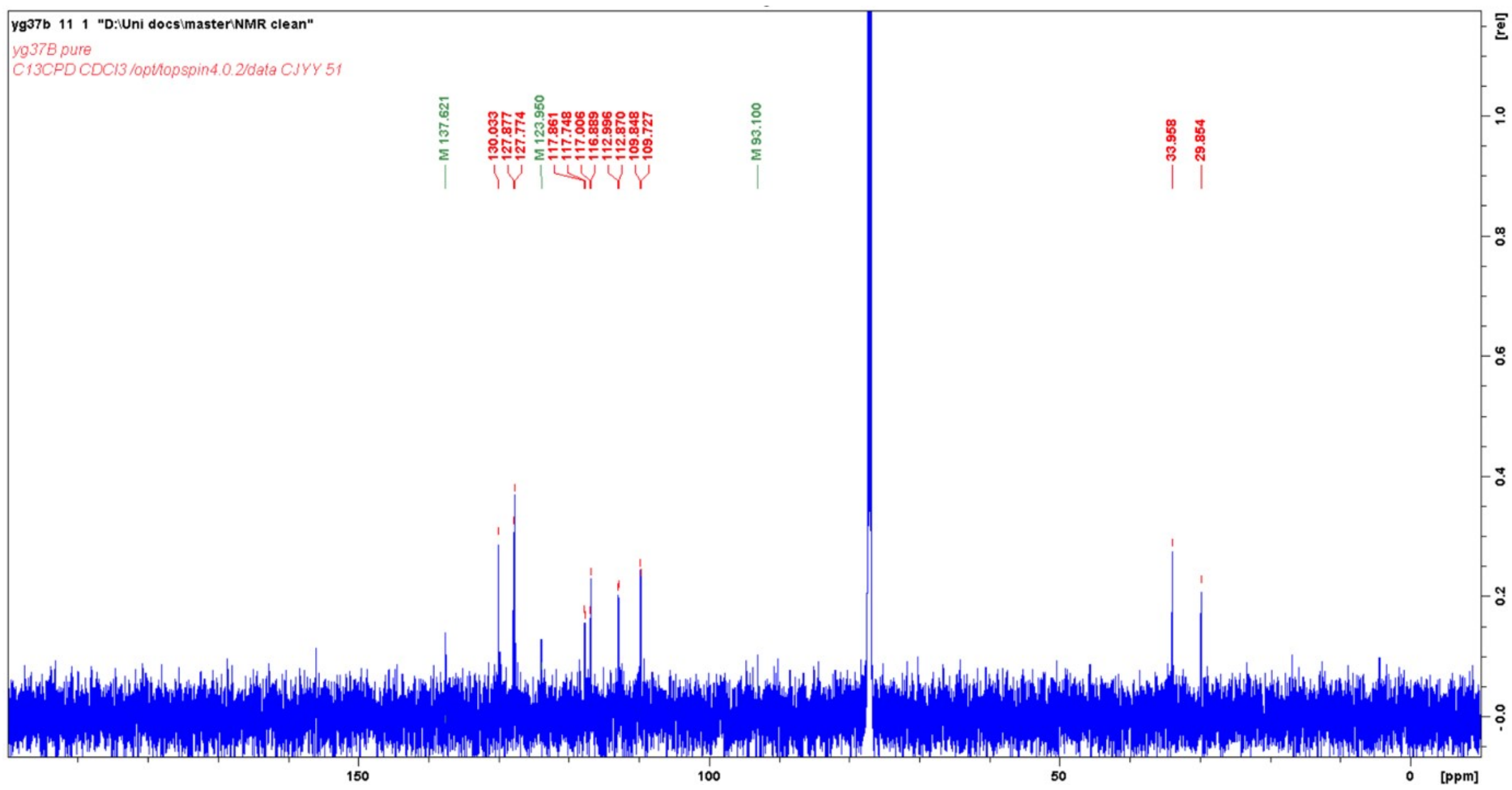

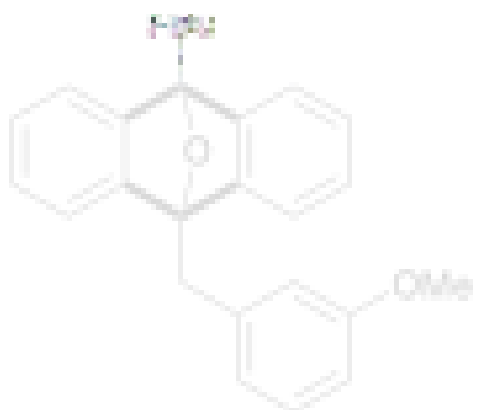

^{1}H NMR (400 MHz, $CDCl_3$), **16fa**

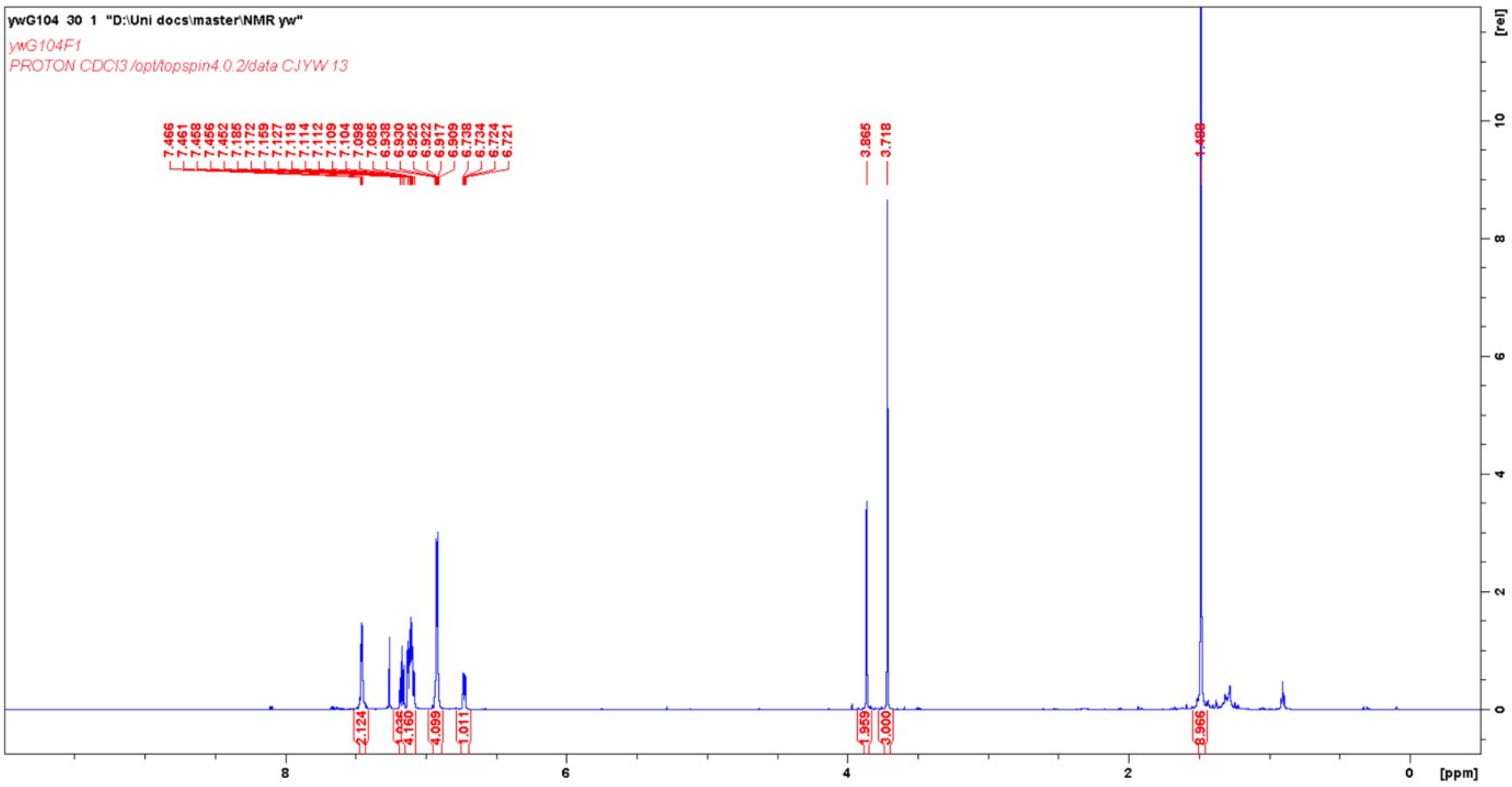

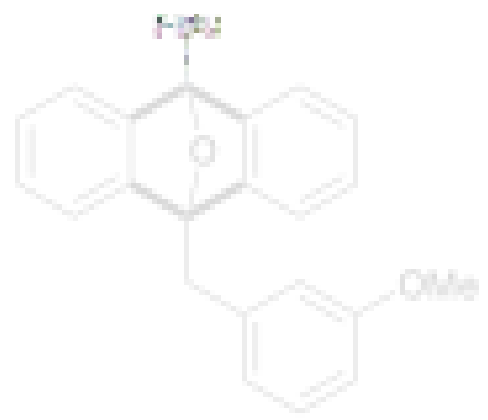

^{13}C NMR (101 MHz, $CDCl_3$), **16fa**

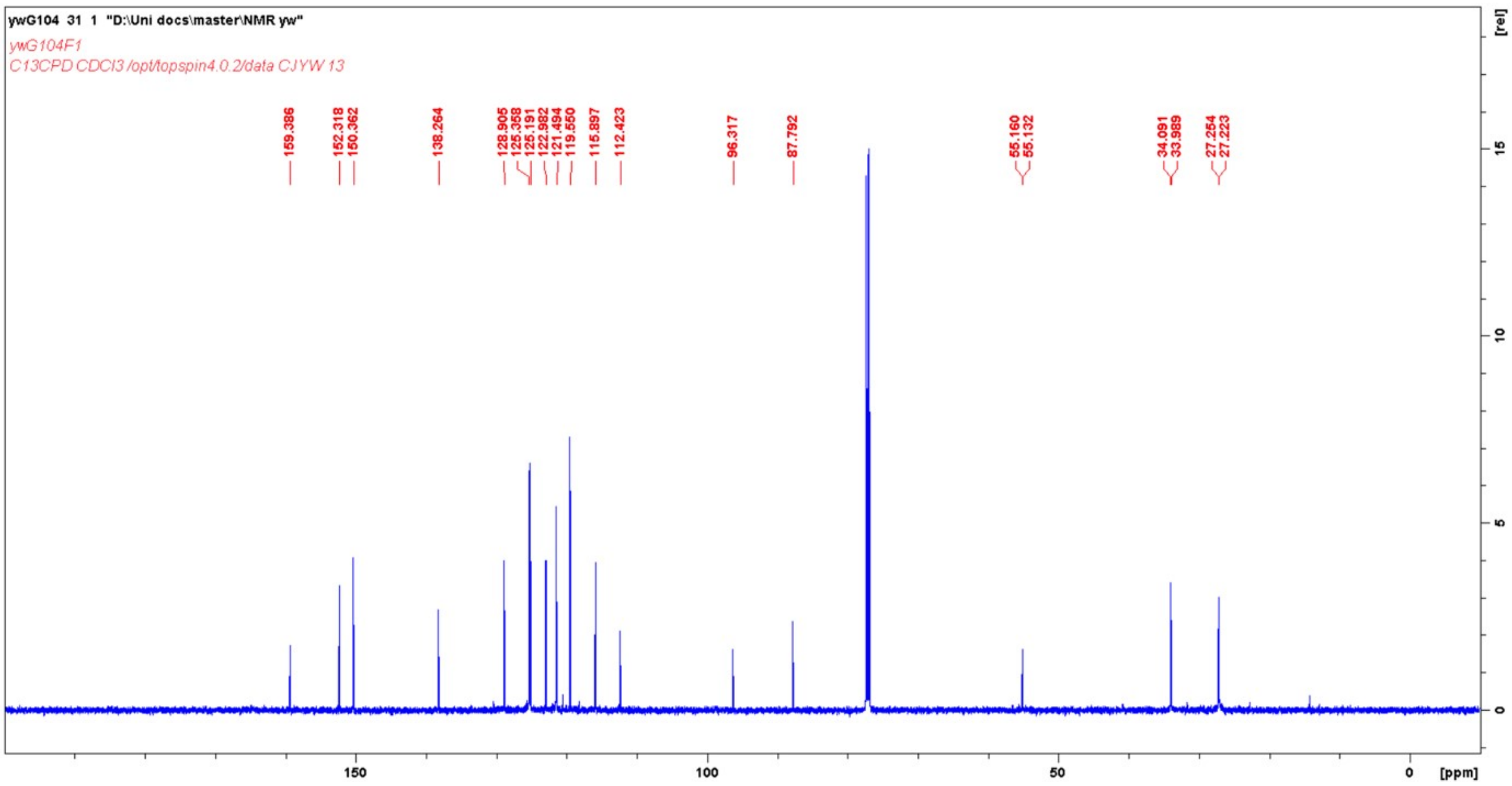

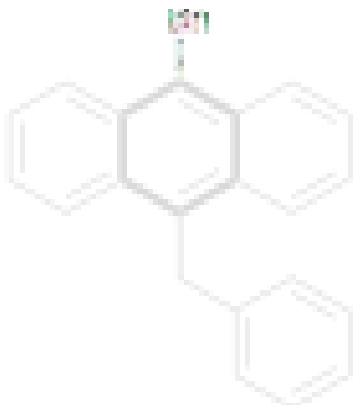

^1H NMR (400 MHz, $CDCl_3$), **29a**

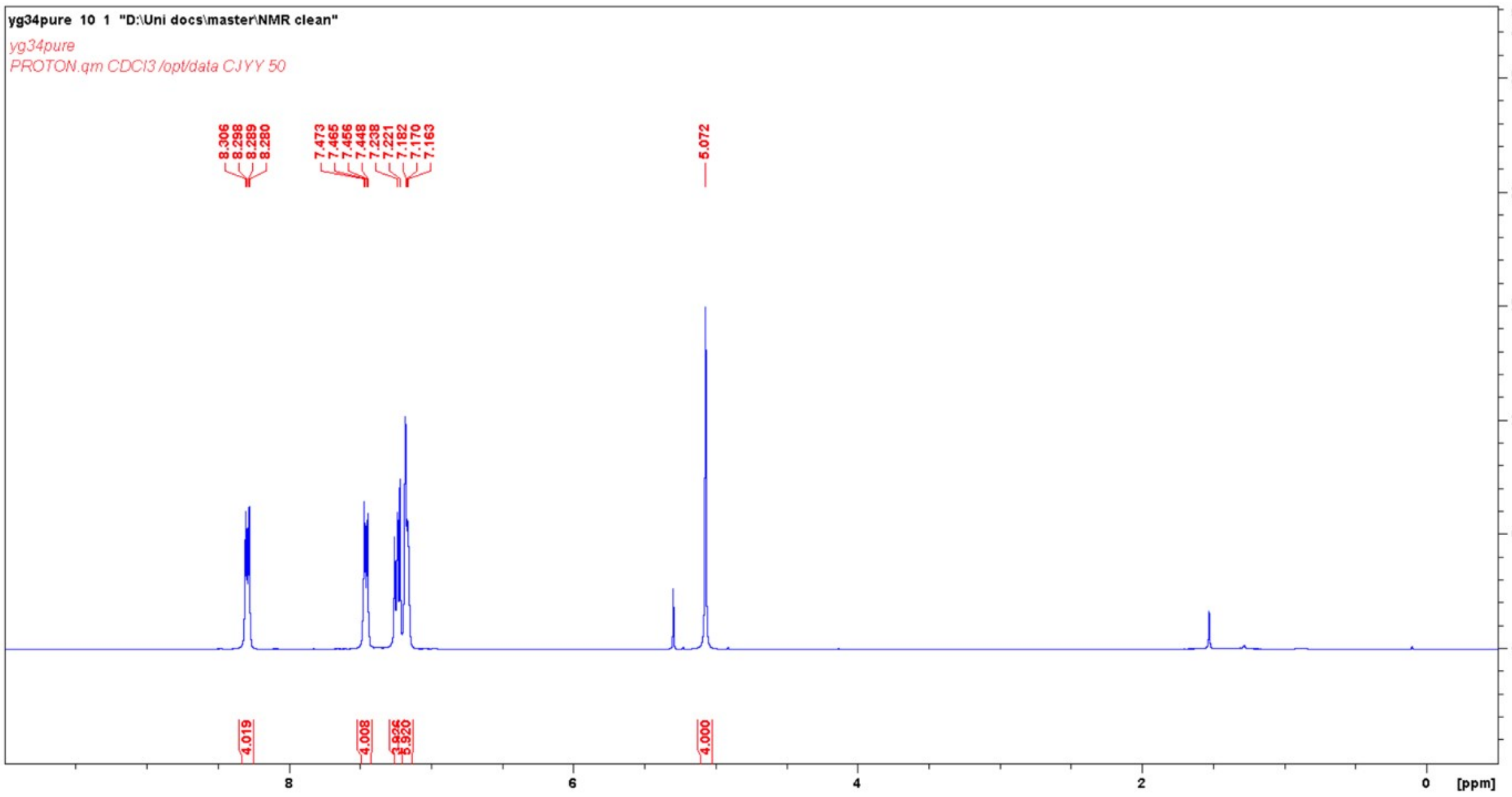

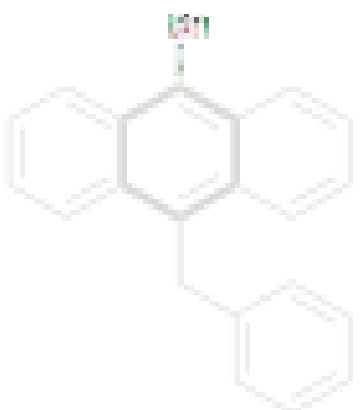

^{13}C NMR (101 MHz, $CDCl_3$), **29a**

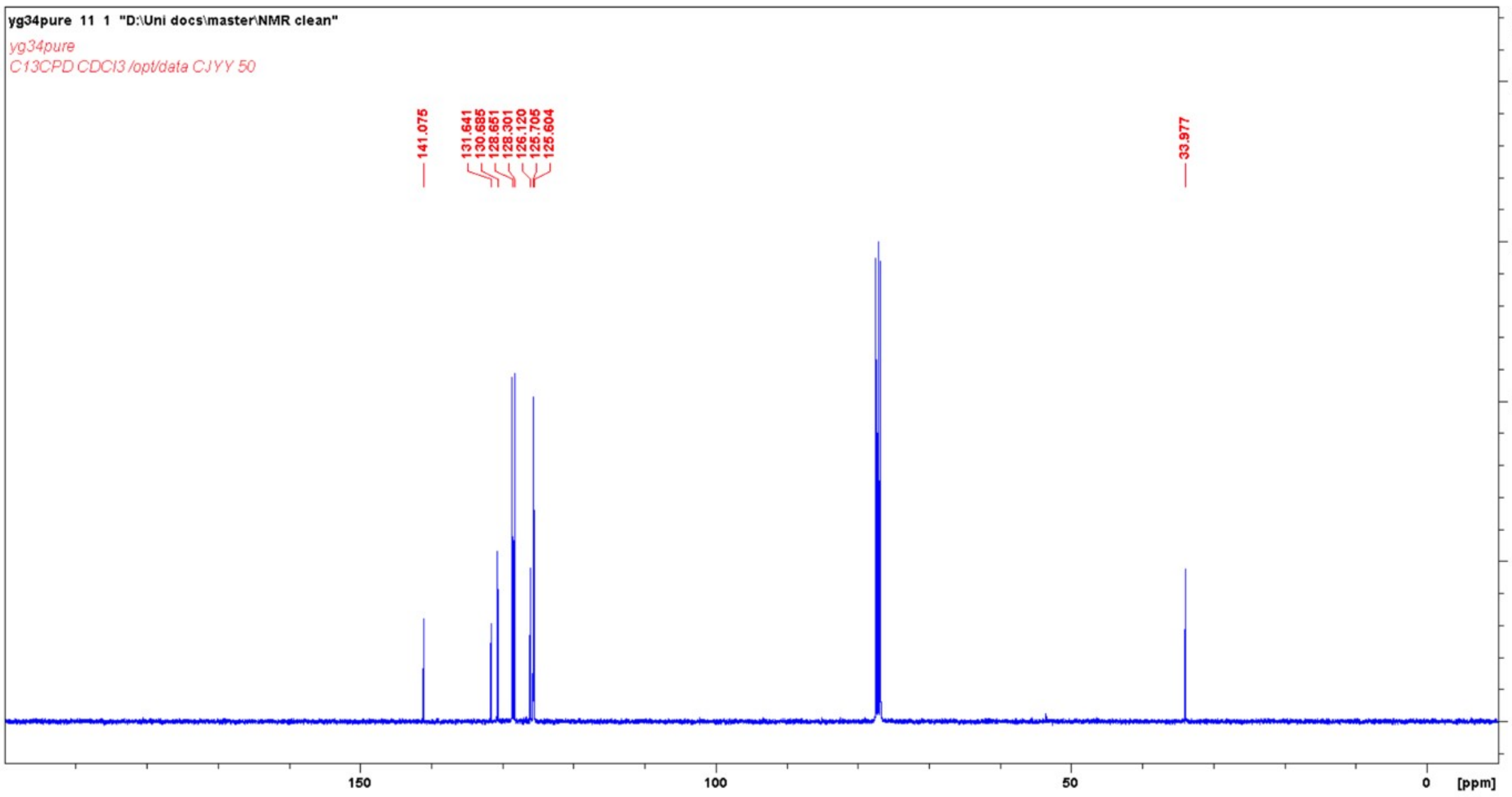

Supplement: Supplementary file 1 [file QO-013-D6QO01223J-s001.pdf]
